# Gene Ontology and KEGG Enrichment Analyses of Genes Related to Age-Related Macular Degeneration

**DOI:** 10.1155/2014/450386

**Published:** 2014-08-06

**Authors:** Jian Zhang, ZhiHao Xing, Mingming Ma, Ning Wang, Yu-Dong Cai, Lei Chen, Xun Xu

**Affiliations:** ^1^Department of Ophthalmology, Shanghai First People's Hospital, School of Medicine, Shanghai Jiaotong University, Shanghai 200080, China; ^2^Shanghai Key Laboratory of Ocular Fundus Diseases, Shanghai First People's Hospital, School of Medicine, Shanghai Jiaotong University, Shanghai 200080, China; ^3^The Key Laboratory of Stem Cell Biology, Institute of Health Sciences, Shanghai Jiaotong University School of Medicine and Shanghai Institutes for Biological Sciences, Chinese Academy of Sciences, Shanghai 200025, China; ^4^Institute of Systems Biology, Shanghai University, Shanghai 200444, China; ^5^College of Information Engineering, Shanghai Maritime University, Shanghai 201306, China

## Abstract

Identifying disease genes is one of the most important topics in biomedicine and may facilitate studies on the mechanisms underlying disease. Age-related macular degeneration (AMD) is a serious eye disease; it typically affects older adults and results in a loss of vision due to retina damage. In this study, we attempt to develop an effective method for distinguishing AMD-related genes. Gene ontology and KEGG enrichment analyses of known AMD-related genes were performed, and a classification system was established. In detail, each gene was encoded into a vector by extracting enrichment scores of the gene set, including it and its direct neighbors in STRING, and gene ontology terms or KEGG pathways. Then certain feature-selection methods, including minimum redundancy maximum relevance and incremental feature selection, were adopted to extract key features for the classification system. As a result, 720 GO terms and 11 KEGG pathways were deemed the most important factors for predicting AMD-related genes.

## 1. Introduction

Age-related macular degeneration (AMD or ARMD) is a chronic, progressive eye disorder that primarily occurs in elders (>50 years) and has become a major cause of blindness and visual impairment in developed countries as well as the third major cause globally [1, 2]. In an Asian population aged 40–79 years, the morbidities of early and late AMD were 6.8% and 0.56%, respectively [3]. Further, AMD is likely to increase with a longer life expectancy. Due to retina damage, AMD typically results in vision loss, which can render daily activities difficult, such as reading, watching TV, and recognizing faces [4]. There are two typical types of AMD: dry AMD and wet AMD. Dry AMD is the major type of AMD and accounts for approximately 80% of cases; no efficient surgical or medical treatments are available. It typically causes mild vision loss, which develops slowly. However, it can cause vision loss through retinal pigment epithelial layer atrophy, which results in photoreceptor loss (rods and cones) in the central portion of the eye. Wet AMD is caused by choroidal neovascularization (CNV), wherein new blood vessels grow in choriocapillaries through the Bruch's membrane. Leaking and bleeding of these vessels can damage the rods and cones, which lead to rapidly deteriorating vision. Thus, wet AMD accounts for 90% of AMD cases with severe visual impairment.

The AMD etiology is complex. AMD results from both genetic and environmental factors; however, the underlying mechanisms are unclear. Moreover, previous studies have demonstrated strong correlations between AMD and multiple environmental factors. In addition to age, many risk factors are correlated with AMD, such as cigarette smoking [5], oxidative stress [6–8], hypertension, previous cataract surgery, higher body mass index, a history of cardiovascular disease, and higher plasma fibrinogen [9].

AMD is characterized by complex traits. Moreover, mutant protein expression may begin early in AMD patients, and symptoms associated with AMD do not manifest until a long time thereafter. Often only clinical information for a single generation is available for studies; thus, it is difficult to detect AMD phenotypic heterogeneity and determine the underlying mechanisms. Initially, through early linkage studies on small families, several genetic loci at chromosomes 9p24, 10q26, and 15q21 [10] and 1q31, 10q26, and 17q25 [11] were identified and verified. A GWAS study greatly increased our understanding of AMD risk loci. Subsequently, more AMD-related genes have been identified, such as* C2* [12],* CFH* [13],* CFI* [14],* LIPC* [15],* CETP*,* TIMP3* [16], and* TNFRSF10A* [17]. Recently a large-scale GWAS analysis of more than 17,000 AMD cases indicated 19 other AMD loci, in which 7 loci were novel and near the genes* IER3-DDR1*,* COL8A1-FILIP1L*,* SLC16A8*,* TGFBR1*,* ADAMTS9*,* RAD51B,* and* B3GALTL* [18]. Several studies have evaluated the impact of susceptibility genes on AMD onset and progression. For instance,* CFH* gene mutations yield a high risk of AMD. Compared with the normal homozygous genotype, individuals with heterozygotic and homozygotic* CFH* exhibited a 4.6-fold or 7.4-fold increased AMD risk, respectively [19].

AMD is a disease with complex inheritance patterns, and it may be difficult to discover individual susceptibility genes due to multiple genetic and environmental effects and interactions. Identifying several genetic loci revealed that several important biological pathways are involved in AMD pathogenesis, such as the cholesterol, lipid metabolism pathway, complement pathway, extracellular matrix pathway, oxidative stress pathway, and angiogenesis signaling pathway in [20–22], which provides a foundation for systematically analyzing the biological processes underlying AMD. Gene ontology (GO) is a major bioinformatics tool that standardizes representation and the product attributes of genes across species [23]. The Kyoto Encyclopedia of Genes and Genomes (KEGG) [24, 25] pathway database is a collection of manually drawn diagrams and comprehensive inferences for pathway mapping. Based on the gene ontology and KEGG pathway materials, we analyzed the GO and KEGG enrichments for known AMD-related genes, which were retrieved from the Retina International website (http://www.retina-international.org/files/sci-news/remacdy.htm) or the published literature. To extract the distinctive features of these genes, certain genes, which were not reported as AMD-related genes, were randomly selected from Ensemble. Each investigated gene was encoded into numeric vectors consisting of enrichment scores of the gene set, including it and its direct neighbors in STRING, and the GO terms or KEGG pathways. Based on certain feature-selection methods and SMO as the prediction engine, certain important GO terms and KEGG pathways were discovered that were deemed important for identifying AMD-related genes. Analyses suggest that certain such genes relate directly or indirectly to AMD formation or development.

## 2. Materials and Methods

### 2.1. Dataset

The known AMD-related genes were retrieved from the Retina International website (http://www.retina-international.org/files/sci-news/remacdy.htm, recent update from March 24, 2010) and the literature. Specifically, 16 genes are from Retina International; three genes for the complement system proteins factor H (*CFH*), factor 3 (*C3*), and factor B (*CFB*), which are strongly related with a person's risk for developing AMD, are employed;* HTRA1* is from [26, 27];* ABCR* is from [28]; 2 genes are from [29, 30]; and 23 genes are from [18]. Finally, 39 known AMD-related genes were collected; these genes are referred to as “positive genes” and compose the gene set *S*_*p*_. To analyze the differences between the positive genes and other genes, we randomly selected 1,950 genes (50 times the number of positive genes) from Ensemble that were not in *S*_*p*_; these 1,950 genes are referred to as “negative genes” and compose the set *S*_*n*_. The Ensemble IDs for the positive and negative genes are in Supplementary Material I available online at http://dx.doi.org/10.1155/2014/450386.

The negative genes outnumbered the positive genes; thus, we confronted an imbalanced dataset. Encouraged by certain studies that have managed this type of data [31, 32], the following strategy was adopted. The negative genes were equally and randomly split into 10 portions *S*_*n*_^1^, *S*_*n*_^2^,…, *S*_*n*_^10^ (i.e., *S*_*n*_ = *S*_*n*_^1^ ∪ *S*_*n*_^2^ ∪ ⋯∪*S*_*n*_^10^ and *S*_*n*_^*i*^∩*S*_*n*_^*j*^ = *ϕ* for *i* ≠ *j*). For each *S*_*n*_^*i*^, we combined the genes in *S*_*p*_ and *S*_*n*_^*i*^ to comprise the *i*th datasets *D*_*i*_ (i.e., *D*_*i*_ = *S*_*n*_^*i*^ ∪ *S*_*p*_).

### 2.2. Feature Construction

To analyze the differences between the positive and negative genes, each gene must be represented by certain features that can then be processed by certain computer programs. Here, we adopted gene ontology (GO) and KEGG enrichment to compute numerical values that represent each gene.

GO enrichment indicates the relationship between genes and GO terms. For each gene *g* and each GO term GO_*j*_, a score is generated, which is typically referred to as the gene ontology enrichment score and defined as the −log_10_ of the hypergeometric test *P* value [33–35] for a gene set *G* consisting of *g*'s direct neighbors in STRING and the GO term GO_*j*_ that can be computed as follows:

(1) $$\begin{array}{c} \text{E}{\text{S}}_{\text{GO}}\left(g,\text{G}{\text{O}}_{j}\right)=-\text{lo}{\text{g}}_{10}\left(\overset{n}{\underset{k=m}{\sum }}\frac{\left(\begin{array}{c} M\\ m \end{array}\right)\left(\begin{array}{c} N-M\\ n-m \end{array}\right)}{\left(\begin{array}{c} N\\ n \end{array}\right)}\right), \end{array}$$

where *N* denotes the overall number of proteins in humans, *M* denotes the number of proteins annotated in the gene ontology term GO_*j*_, *n* denotes the number of proteins in *G*, and *m* denotes the number of proteins in *G* that are annotated in the gene ontology term GO_*j*_. If the score is large for one gene and one GO term, the gene and GO term likely have a strong relationship; there were 12,877 gene ontology enrichment scores.

Similarly, for each gene *g* and each KEGG pathway *P*_*j*_, the KEGG enrichment score is defined as the −log_10_ of the hypergeometric test *P* value [35, 36] for a gene set *G* that consists of *g*'s direct neighbors in STRING and the KEGG pathway *P*_*j*_, which can be calculated as follows:

(2) $$\begin{array}{c} \text{E}{\text{S}}_{\text{KEGG}}\left(g,P_{j}\right)=-\text{lo}{\text{g}}_{10}\left(\overset{n}{\underset{k=m}{\sum }}\frac{\left(\begin{array}{c} M\\ m \end{array}\right)\left(\begin{array}{c} N-M\\ n-m \end{array}\right)}{\left(\begin{array}{c} N\\ n \end{array}\right)}\right), \end{array}$$

where *N* denotes the overall number of proteins in humans, *M* denotes the number of proteins annotated in the KEGG pathway *P*_*j*_, *n* denotes the number of proteins in *G*, and *m* denotes the number of proteins in *G* that are annotated in the KEGG pathway *P*_*j*_. Additionally, a higher KEGG enrichment score between *g* and *P*_*j*_ indicates a stronger relationship; 239 features were KEGG enrichment scores.

Accordingly, each gene *g* can be represented by 12,877 gene ontology enrichment scores and 239 KEGG enrichment scores, which can be formulated as follows:

(3) v(g)=(ESGO(g,GO1),…,ESGO(g,GO12877),  ESKEGG(g,P1),…,ESKEGG(g,P239))T.

### 2.3. Prediction Method and Accuracy Measurement

Weka [37] is a collection of many state-of-the-art machine-learning algorithms and has been used to solve various biological problems [38–42]. One classifier, which is referred to as SMO, was adopted herein as the classification method; it implements John Platt's sequential minimal optimization algorithm to solve the optimization problem that should be settled during training of a support vector classifier. The kernel function can be polynomial or Gaussian [43, 44].

The predicted results for a two-class classification problem can be represented by a confusion matrix consisting of four entries: a true positive (TP), a true negative (TN), false positives (FP), and a false negative (FN) [45, 46]. Accordingly, the prediction accuracy (ACC), specificity (SP), and sensitivity (SN) can be computed as follows:

(4) $$\begin{array}{c} \text{ACC}=\frac{\text{TP}+\text{TN}}{\text{TP}+\text{TN}+\text{FP}+\text{FN}},\\ \text{SP}=\frac{\text{TN}}{\text{TN}+\text{FP}},\\ \text{SN}=\frac{\text{TP}}{\text{TP}+\text{FN}}. \end{array}$$

However, in each dataset *D*_*i*_, the number of negative genes was 5 times as many as the number of positive genes, which is still imbalanced. Thus, an additional measurement, Matthews's correlation coefficient (MCC) [47], was employed to solve the problem; the coefficient can be computed as follows:

(5) MCC =TP·TN−FP·FN(TN+FN)·(TN+FP)·(TP+FN)·(TP+FP).

### 2.4. 10-Fold Cross Validation

Ten-fold cross validation is often used to examine the performance of various classification models [48]. In 10-fold cross validation, the dataset is equally and randomly divided into ten portions. Each portion is used as testing data, and the samples in the remaining nine portions compose the training dataset. Each sample is tested once because each portion is tested once. Compared with the Jackknife test [49, 50], a 10-fold cross-validation test is more efficient and provides similar results for a given dataset. Thus, it was adopted herein to examine the classification model.

### 2.5. Feature Selection

As described in Section 2.2, each gene is represented by 12,877 + 239 = 13,116 enrichment scores. To analyze these features and extract key features that contribute the most to the positive and negative gene classification, certain feature-selection methods were employed. This procedure included two stages: (1) using Cramer's coefficient [51, 52] to exclude nonsignificant features and (2) using the minimum redundancy maximum relevance (mRMR) method as well as incremental feature selection (IFS) [53] for additional selection.

Cramer's coefficient [51, 52] is a statistical measure of two variables that was derived from the Pearson Chi-square test [54]; it ranges from 0 to 1. A high Cramer's coefficient for two variables indicates a strong association. Here, for each feature and samples' class labels, Cramer's coefficient was calculated, and features with a Cramer's coefficient lower than 0.1 were excluded.

The remaining features were further refined using the minimum redundancy maximum relevance (mRMR) method and incremental feature selection (IFS), which are feature selection methods that have been widely used in recent years [34, 55–58]. By evaluating a classification model, key features can be extracted from a complicated biological system. The mRMR method has two criteria: max-relevance and min-redundancy. Accordingly, two feature lists can be generated using this method: (1) the MaxRel feature list and (2) the mRMR feature list. Specifically, the former list sorts features according to their contributions to the classification (i.e., only considering the criterion of max-relevance), while the latter list sorts features by considering both the max-relevance and min-redundancy criteria. The MaxRel and mRMR features lists were formulated as follows:

(6) MaxRel  features  list:FM=[f1M,f2M,…,fNM],mRMR  features  list:Fm=[f1m,f2m,…,fNm],

where *N* denotes the total number of features. A detailed description of the mRMR method can be found in Peng et al.*'*s paper [53].

Only the mRMR features list was used to extract key features. The extraction procedure is described as follows.

1. For the mRMR features list *F*_*m*_, construct *N* feature set, say *F*_*m*_^1^, *F*_*m*_^2^,…, *F*_*m*_^*N*^, such that *F*_*m*_^*i*^ = [*f*_1_^*m*^, *f*_2_^*m*^,…, *f*_*i*_^*m*^] (1 ≤ *i* ≤ *N*) (i.e., *F*_*m*_^*i*^ contained the first *i* features in *F*_*m*_).
2. The classifier SMO was evaluated through 10-fold cross validation using features in *F*_*m*_^*i*^. As described in Section 2.3, ACC, SP, SN and MCC can be obtained.
3. The feature set with the maximum MCC is deemed the optimal feature set. For ease in observation, an IFS-curve can be plotted with MCC values as the *y*-axis and the superscript *i* of *F*_*m*_^*i*^ as the *x*-axis.

## 3. Results and Discussion

### 3.1. Results of the First Stage of Feature Selection

For each of the 10 datasets *D*_1_, *D*_2_,…, *D*_10_, Cramer's coefficients of the features and samples' class labels were calculated. Accordingly, features with Cramer's coefficients less than 0.1 were excluded, while the remaining features were processed further. The number of remaining features in each dataset is listed in Table 1.

### 3.2. Results of the Second Stage of Feature Selection

For each dataset *D*_*i*_, the mRMR, IFS, and SMO methods were used to process the remaining features. The mRMR program was retrieved from http://research.janelia.org/peng/proj/mRMR/ and was executed with its default parameters. As a result, we generated two feature lists: the MaxRel and mRMR features lists. To reduce the computation time, only the first 500 features in each of the two feature lists were obtained, and they are available in Supplementary Material II.

**Figure 1:**
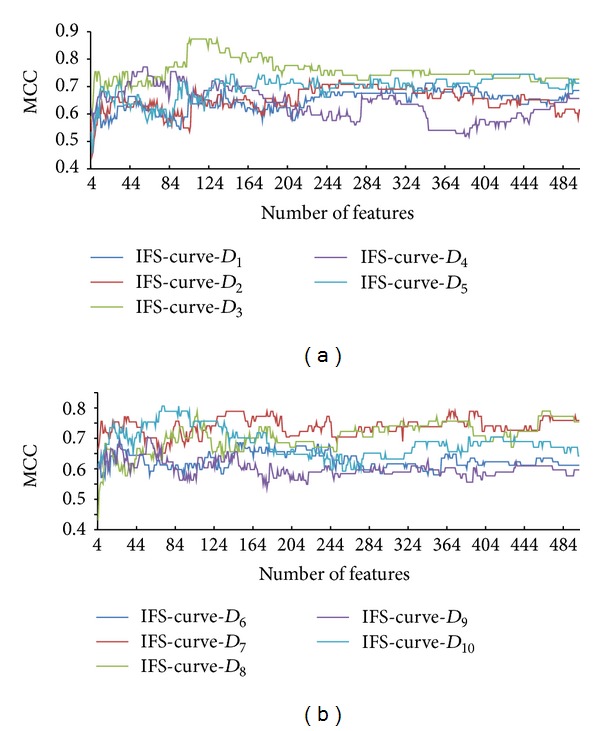
IFS-curve for each dataset. Specifically, (a) shows the IFS-curves for the datasets *D*_1_, *D*_2_, *D*_3_, *D*_4_, and *D*_5_, while (b) shows the IFS-curves for the datasets *D*_6_, *D*_7_, *D*_8_, *D*_9_, and *D*_10_. The *y*-axis represents Matthews's correlation coefficient (MCC), and the *x*-axis represents the number of features involved in the classification model.

The IFS and SMO methods were used in accordance with the mRMR features list for each dataset *D*_*i*_ evaluated using 10-fold cross validation. The SNs, SPs, ACCs, and MCCs obtained for each dataset *D*_*i*_ are available in Supplementary Material III. For clarity, we plotted an IFS-curve for each dataset *D*_*i*_, which is referred to as IFS-curve-*D*_*i*_. The five IFS-curves for *D*_1_, *D*_2_, *D*_3_, *D*_4_, and *D*_5_ are shown in Figure 1(a), while the other five IFS-curves for *D*_6_, *D*_7_, *D*_8_, *D*_9_, and *D*_10_ are shown in Figure 1(b); the ten IFS-curves that are plotted in separate coordinates are available in Supplementary Material IV. Generating the maximum MCC for each dataset from Supplementary Material III and IV (listed in column 3 of Table 2) was a straightforward process. Clearly, most MCCs are in the range 0.7 to 0.8, and the mean value was 0.76139. As mentioned in Section 2.5, the features used to obtain the maximum MCC compose the optimal feature set. The number of features in the optimal feature set for each dataset is listed in column 2 of Table 2. The results for dataset *D*_1_ are described as follows. The maximum MCC for the dataset *D*_1_ is 0.712699 (listed in row 2 and column 3 of Table 2) using the first 344 (listed in row 2 and column 2 of Table 2) features in the mRMR features list of dataset *D*_1_ (see Supplementary Material II).

### 3.3. Analysis of the Optimal Feature Set

**Figure 2:**
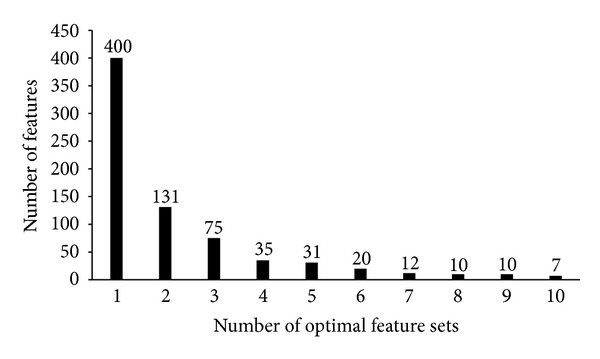
The number of features against the number of optimal feature sets.

As mentioned in Section 3.2, we generated an optimal feature set for each dataset, thereby obtaining 10 optimal feature sets. We combined these optimal feature sets to compose the final optimal feature set, which includes 720 GO terms and 11 KEGG pathways that are available in Supplementary Material V. To discern the distribution of these 731 optimal features, we counted the number of optimal feature sets containing each of 731 features.  Figure 2 shows the number of features against the number of optimal feature sets, from which we can see that 400 features were exactly contained in one optimal feature set, 131 features were exactly contained in two optimal feature sets, while others were contained in at least three optimal feature sets. Accordingly, 45.28% (331/731) features were contained in at least two optimal feature sets, indicating that different datasets may induce some common features. It also suggested that some important features for distinguishing AMD-related genes were contained in the final optimal feature set. In the following sections, features in the final optimal feature set were discussed.

#### 3.3.1. GO Number and Percentage

**Figure 3:**
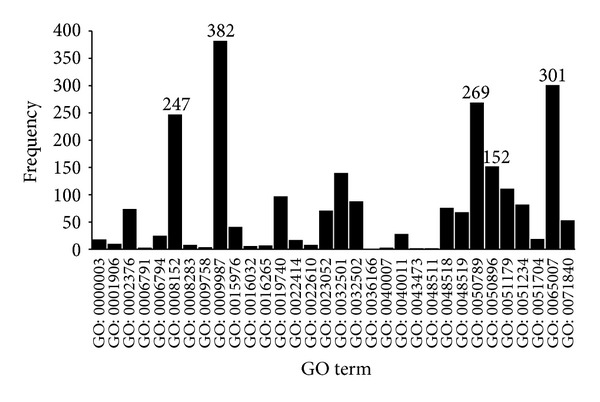
Frequency of children terms of biological process GO terms in the final optimal feature set.

**Figure 4:**
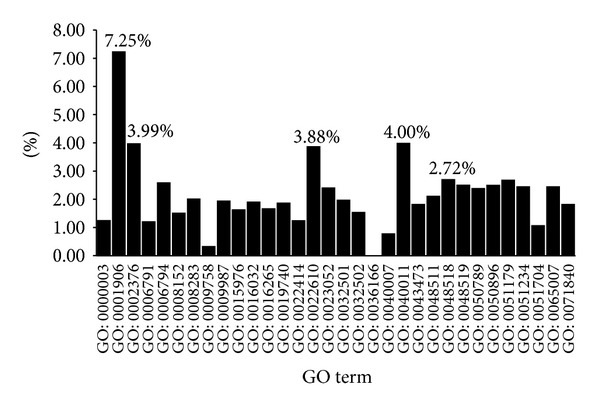
Percentage of children terms of biological process GO terms in the final optimal feature set.

**Figure 5:**
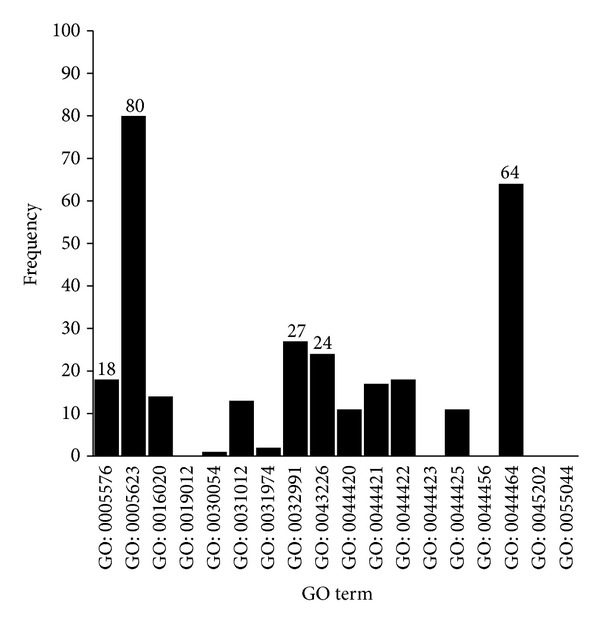
Frequency of children terms of cellular component GO terms in the final optimal feature set.

**Figure 6:**
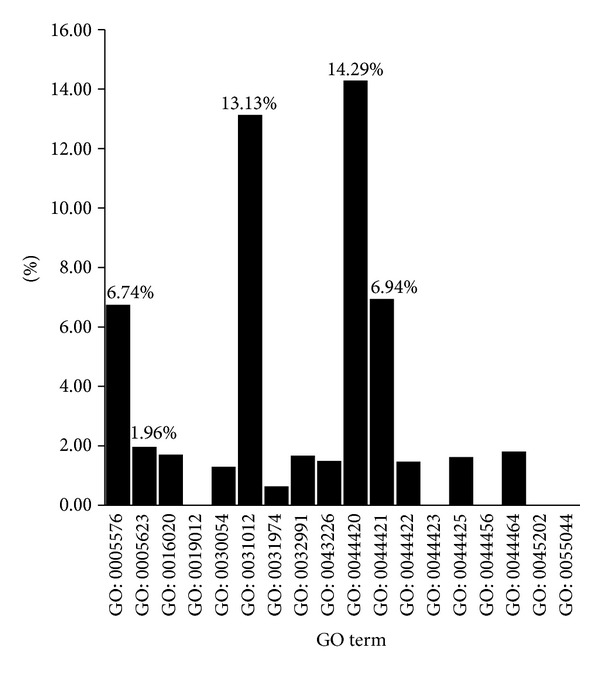
Percentage of children terms of cellular component GO terms in the final optimal feature set.

**Figure 7:**
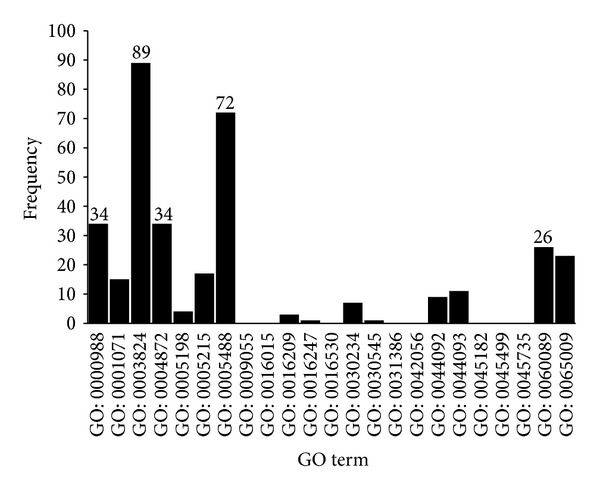
Frequency of children terms of molecular function GO terms in the final optimal feature set.

**Figure 8:**
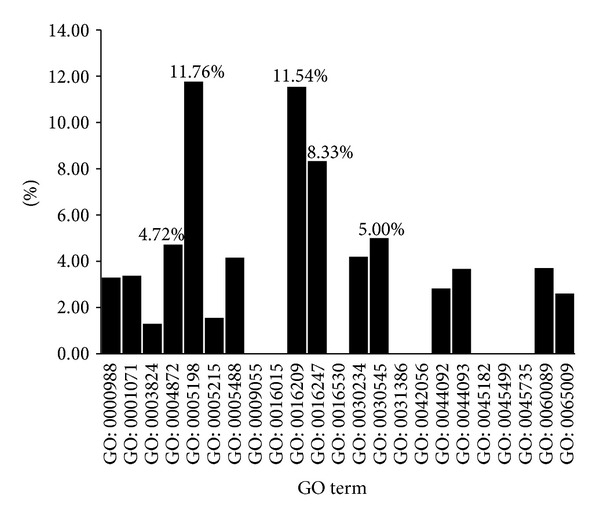
Percentage of children terms of molecular function GO terms in the final optimal feature set.

It is known that GO terms can be divided into the following three types: (1) biological process (BP) GO term, (2) cellular component (CC) GO term, and (3) molecular function (MF) GO term. To efficiently discern the biological meanings and characterize the functional essentiality of the GO terms in the final optimal feature set, we considered the children terms of the aforementioned three types. For clarity, let *S*_*o*_ be the 720 GO terms in the final optimal feature set and *S* be the children terms of any children term of BP GO term, CC GO term, or MF GO term. To display the distribution of the GO terms in *S*_*o*_, we calculated the frequency and percentage for each children term of BP GO term, CC GO term, or MF GO term which were defined as |*S*_*o*_∩*S*| and |*S*_*o*_∩*S*|/|*S*|, respectively. Figures 3–8 display the frequency and percentage of children terms of BP GO term, CC GO term, or MF GO term in the final optimal feature set.

(1)* BP GO Terms.* In Figure 3, based on the BP term frequencies, the top five biological process terms are (I) GO: 0009987: cellular process (382); (II) GO: 0065007: biological regulation (301); (III) GO: 0050789: regulation of biological process (269); (IV) GO: 0008152: metabolic process (247); and (V) GO: 0050896: response to stimulus (152).

The top four BP terms may indicate that these biological processes are necessary to maintain normal cellular functions and may lead to AMD due to aberrant behavior in relevant cells.

“Response to stimulus” refers to any process that results from a stimulus, which leads to a change in a state or activity, such as movement and secretion.

For the BP term percentages, as shown in Figure 4, the top five biological process terms are (I) GO: 0001906: cell killing (7.25%); (II) GO: 0040011: locomotion (4.00%); (III) GO: 0002376: immune system process (3.99%); (IV) GO: 0022610: biological adhesion (3.88%), and (V) GO: 0048518: positive regulation of a biological process (2.72%).

Biological adhesion between substrate and cells modulates several critical cellular processes, such as cell locomotion and gene expression [59]. Biological adhesion- and locomotion-related gene dysfunction may result in AMD. Previous research has shown that the immune system, particularly the complement system, is relevant to AMD. Genetic studies also indicate that several complement-related genes, including* CFH*,* complement component 2*,* complement component 3*,* CFHR1,* and* CFHR3*, are highly associated with AMD [60]. Further, complement can enhance the generation of VEGF (vascular endothelial growth factor), which may strongly facilitate AMD development [61]. Histological studies show the presence of macrophages, lymphocytes, mast cells, and fibroblasts in both atrophic lesions and with retinal neovascularization [61]. 

(2)* CC GO Terms.* In Figure 5, for the cellular component GO term frequency, the top five CC terms are (I) GO: 0005623: cell (80); (II) GO: 0044464: cell part (64); (III) GO: 0032991: macromolecular complex (27); (IV) GO: 0043226: organelle (24); and (V) GO: 0005576: extracellular region (18). Cell, cell part, organelle, and macromolecular complex inclusion may be attributed to large base numbers of these GO terms.

For the percentage of cellular component terms, as shown in Figure 6, the top five CC terms include (I) GO: 0044420: extracellular matrix part (14.29%); (II) GO: 0031012: extracellular matrix (13.13%); (III) GO: 0044421: extracellular region part (6.94%); (IV) GO: 0005576: extracellular region (6.74%); and (V) GO: 0005623: cell (1.96%).

From the distribution of CC terms, except for the cell term (GO: 0005623), the top four CC terms are associated with the extracellular matrix. Moreover, the extracellular region is relevant to cell adhesion and locomotion, which were mentioned in the biological process GO terms.

The results are also consistent with a recent GWAS study, which identified several new loci with enrichment for genes involved in the extracellular matrix and other activities [18]. Structural damage of extracellular matrix in retinal cells may lead to break point of AMD [62]. Matrix metalloproteinases result in extracellular matrix degradation and are highly related to AMD pathogenesis [63]. Therefore, taken together, these facts suggest that the extracellular matrix plays an important role in AMD.

(3)* MF GO Terms.* In Figure 7, based on the frequency of molecular function terms, the top five MF terms are (I) GO: 0003824: catalytic activity (89); (II) GO: 0005488: binding (72); (III) GO: 0000988: protein binding transcription factor activity (34); (IV) GO: 0004872: receptor activity (34); and (V) GO: 0060089: molecular transducer activity (26).

MF terms related to catalytic activity and binding were highlighted partly due to the large base numbers of these terms. However, this finding may suggest that genes assigned to these two terms are essential to maintain normal function. For example, matrix metalloproteinases, which can degrade extracellular matrix proteins, play an important role in AMD [63]. In addition, highlighting receptor activity and molecular transducer activity indicates that abnormal cellular signal pathway behaviors are involved in AMD patients. For example, the Aryl hydrocarbon receptor, which is responsible for clearing cellular debris and for toxin metabolism, is essential to maintaining normal function in RPE cells, and deficiency of this receptor causes AMD in mice [64].

For the percentage of molecular function terms, as shown in Figure 8, the top five MF terms are (I) GO: 0005198: structural molecule activity (11.76%); (II) GO: 0016209: antioxidant activity (11.54%); (III) GO: 0016247: channel regulator activity (8.33%); (IV) GO: 0030545: receptor regulator activity (5.00%); and (V) GO: 0004872: receptor activity (4.72%). To our surprise, receptor activity was highlighted in both the frequency and percentage of molecular function terms, which is further evidence of the important role that receptor activity plays in AMD. Antioxidant activity is also highlighted, and oxidative stress [6] is a risk factor correlated with AMD. Channel regulator activity and structural molecule activity may also be involved in AMD.

#### 3.3.2. The KEGG Pathways in the Final Optimal Set

Based on the final optimal set, we obtained 11 KEGG pathways, which are (I) hsa00290 (valine, leucine, and isoleucine biosynthesis); (II) has00450 (selenocompound metabolism); (III) hsa00512 (mucin-type O-glycan biosynthesis); (IV) hsa03013 (RNA transport); (V) hsa04145 (phagosome); (VI) hsa04610 (complement and coagulation cascades); (VII) hsa04962 (vasopressin-regulated water reabsorption); (VIII) hsa05133 (pertussis); (IX) hsa05146 (viral myocarditis); and (X) hsa05150 (*Staphylococcus aureus* infection); and (XI) hsa05416 (viral myocarditis).

Valine, leucine, and isoleucine biosynthesis (hsa00290) and selenocompound metabolism (hsa00450) are related to amino acid metabolism. Mucin-type O-glycan biosynthesis is associated with modifications of serine or threonine residues of certain proteins. RNA transport from nucleus to cytoplasm is also essential for gene expression. These terms may not be the key factors in AMD, but they may give us suggestions about the AMD development. Phagosome (hsa04145) is also associated with AMD. There are various forms of cell death and phagocytosis in the retina [65]. But failure of retinal pigment epithelial cells and macrophages to phagocytize dying retinal pigment epithelial cells may result in drusen formation and development of AMD [66]. The underlying mechanism of AMD is still unclear, but many studies have highlighted the essential role of the immune system in the development and progression of AMD [67]. Previous studies have revealed a strong association between complement pathway and AMD [20]. Several complement genes including complement 2 (*C2*) and complement 3 (*C3*) have been strongly associated with AMD [12, 68]. Except vasopressin-regulated water reabsorption, viral myocarditis (hsa05146) and* Staphylococcus aureus* infection (hsa05150) are all correlated with immunity, which further emphasizes the effect of immunity in AMD.

## 4. Conclusions

In this study, we performed GO and KEGG enrichment analyses of AMD-related genes. The results suggest that 720 GO terms and 11 KEGG pathways are important factors that contribute to identifying AMD-related genes.

## Supplementary Material

The Supplementary Material contains five files. In detail, Supplementary Material I lists 39 known AMD related genes and 1,950 randomly selected genes; Supplementary Material II lists the output of mRMR program on each dataset; Supplementary Material III lists the accuracies obtained by IFS and SMO on each dataset; Supplementary Material IV lists the IFS curve on each dataset; Supplementary Material V lists the features in the final optimal feature set.

## Figures and Tables

**Table 1:** The number of remaining features for each dataset after the first stage of feature selection.

Dataset	Number of remaining features
*D*_1_	4,288
*D*_2_	3,919
*D*_3_	4,549
*D*_4_	4,663
*D*_5_	4,371
*D*_6_	5,012
*D*_7_	4,877
*D*_8_	3,787
*D*_9_	4,701
*D*_10_	4,473

**Table 2:** The number of features in the optimal feature set for each dataset and the MCC value obtained using these features.

Dataset	Number of features in the optimal feature set	Maximum MCC value
*D*_1_	344	0.712699
*D*_2_	226	0.723116
*D*_3_	104	0.873086
*D*_4_	57	0.77142
*D*_5_	146	0.744851
*D*_6_	26	0.699118
*D*_7_	136	0.788893
*D*_8_	462	0.789865
*D*_9_	55	0.704687
*D*_10_	70	0.806162

Mean		0.76139
