# Supplementary material for: Gene Ontology and KEGG Enrichment Analyses of Genes Related to Age-Related Macular Degeneration
Source: Biomed Res Int. 2014 Aug 6;2014:450386. doi: 10.1155/2014/450386 (PMC4140130; doi:10.1155/2014/450386)
Supplement: Supplementary file 1 — The Supplementary Material contains five files. In detail, Supplementary Material I lists 39 known AMD related genes and 1,950 randomly selected genes; Supplementary Material II lists the output of mRMR program on each dataset; Supplementary Material III lists the accuracies obtained by IFS and SMO on each dataset; Supplementary Material IV lists the IFS curve on each dataset; Supplementary Material V lists the features in the final optimal feature set. [file 450386.f1.zip › Supp-I.pdf]

Supplementary Material I. 39 known AMD related genes, termed as 'positive genes', and 1,950 randomly selected genes from Ensemble, termed as 'negative genes'.

(1) Positive genes

ENSP00000200676	ENSP00000221132	ENSP00000243222
ENSP00000245907	ENSP00000252486	ENSP00000261037
ENSP00000266085	ENSP00000271588	ENSP00000296795
ENSP00000299022	ENSP00000299367	ENSP00000314299
ENSP00000321735	ENSP00000343002	ENSP00000345008
ENSP00000346560	ENSP00000348089	ENSP00000356395
ENSP00000356399	ENSP00000357980	ENSP00000361125
ENSP00000363089	ENSP00000364133	ENSP00000364660
ENSP00000372853	ENSP00000378130	ENSP00000392835
ENSP00000395405	ENSP00000397956	ENSP00000398139
ENSP00000401397	ENSP00000402278	ENSP00000405800
ENSP00000406091	ENSP00000407961	ENSP00000410815
ENSP00000412283	ENSP00000416561	ENSP00000418735

(2) Negative genes

ENSP00000005284	ENSP00000006015	ENSP00000006526
ENSP00000007722	ENSP00000013034	ENSP00000028008
ENSP00000053468	ENSP00000075503	ENSP00000085068
ENSP00000155926	ENSP00000156825	ENSP00000159087
ENSP00000159111	ENSP00000164227	ENSP00000164640
ENSP00000171111	ENSP00000171757	ENSP00000172229
ENSP00000173229	ENSP00000173898	ENSP00000175091
ENSP00000176183	ENSP00000177694	ENSP00000181839
ENSP00000183605	ENSP00000188790	ENSP00000192314
ENSP00000194871	ENSP00000196489	ENSP00000196548
ENSP00000198765	ENSP00000201031	ENSP00000201979
ENSP00000202017	ENSP00000202788	ENSP00000203629
ENSP00000204549	ENSP00000204961	ENSP00000205061
ENSP00000205143	ENSP00000205194	ENSP00000205214
ENSP00000207549	ENSP00000215587	ENSP00000215727
ENSP00000215862	ENSP00000215939	ENSP00000216044
ENSP00000216133	ENSP00000216200	ENSP00000216338
ENSP00000216463	ENSP00000216520	ENSP00000216639
ENSP00000216797	ENSP00000216911	ENSP00000217188
ENSP00000217372	ENSP00000217652	ENSP00000217901
ENSP00000218006	ENSP00000218197	ENSP00000218432
ENSP00000218721	ENSP00000219070	ENSP00000219150
ENSP00000219169	ENSP00000219406	ENSP00000219481
ENSP00000220507	ENSP00000220584	ENSP00000220616

ENSP00000220931	ENSP00000221452	ENSP00000221466
ENSP00000221856	ENSP00000221859	ENSP00000221891
ENSP00000221996	ENSP00000222120	ENSP00000222254
ENSP00000222284	ENSP00000222482	ENSP00000222598
ENSP00000222728	ENSP00000222823	ENSP00000223190
ENSP00000223368	ENSP00000223428	ENSP00000223795
ENSP00000224600	ENSP00000225328	ENSP00000225519
ENSP00000225525	ENSP00000225603	ENSP00000225729
ENSP00000225737	ENSP00000225873	ENSP00000225941
ENSP00000225972	ENSP00000226091	ENSP00000226230
ENSP00000226522	ENSP00000226840	ENSP00000227135
ENSP00000227378	ENSP00000227638	ENSP00000227918
ENSP00000228245	ENSP00000228284	ENSP00000228850
ENSP00000229416	ENSP00000229769	ENSP00000229829
ENSP00000230056	ENSP00000230256	ENSP00000230510
ENSP00000230640	ENSP00000231004	ENSP00000231228
ENSP00000231751	ENSP00000231790	ENSP00000232375
ENSP00000232424	ENSP00000233114	ENSP00000233190
ENSP00000233379	ENSP00000233468	ENSP00000233545
ENSP00000233630	ENSP00000233809	ENSP00000233826
ENSP00000234420	ENSP00000234454	ENSP00000235310
ENSP00000236130	ENSP00000236273	ENSP00000237186
ENSP00000237696	ENSP00000238081	ENSP00000238256
ENSP00000238341	ENSP00000238651	ENSP00000239032
ENSP00000239223	ENSP00000239243	ENSP00000239830
ENSP00000239891	ENSP00000239906	ENSP00000240159
ENSP00000240304	ENSP00000240335	ENSP00000240652
ENSP00000241051	ENSP00000241312	ENSP00000241436
ENSP00000242159	ENSP00000242776	ENSP00000243077
ENSP00000243673	ENSP00000243776	ENSP00000243911
ENSP00000243918	ENSP00000244007	ENSP00000244096
ENSP00000244221	ENSP00000244333	ENSP00000244534
ENSP00000244728	ENSP00000244745	ENSP00000244799
ENSP00000245222	ENSP00000245312	ENSP00000245541
ENSP00000245912	ENSP00000246000	ENSP00000246090
ENSP00000246166	ENSP00000246533	ENSP00000246912
ENSP00000247020	ENSP00000247087	ENSP00000247933
ENSP00000247986	ENSP00000248072	ENSP00000248089
ENSP00000248150	ENSP00000248846	ENSP00000248984
ENSP00000249344	ENSP00000249636	ENSP00000249822
ENSP00000250066	ENSP00000250151	ENSP00000250156
ENSP00000250244	ENSP00000250495	ENSP00000250823
ENSP00000251343	ENSP00000251535	ENSP00000251588
ENSP00000251808	ENSP00000251973	ENSP00000252087

ENSP00000252137	ENSP00000252268	ENSP00000252599
ENSP00000252603	ENSP00000252655	ENSP00000252675
ENSP00000252816	ENSP00000252891	ENSP00000252898
ENSP00000252996	ENSP00000253054	ENSP00000253099
ENSP00000253335	ENSP00000253354	ENSP00000253457
ENSP00000253934	ENSP00000254035	ENSP00000254101
ENSP00000254227	ENSP00000254323	ENSP00000254605
ENSP00000254719	ENSP00000254765	ENSP00000254816
ENSP00000254928	ENSP00000254998	ENSP00000255078
ENSP00000255082	ENSP00000255129	ENSP00000255262
ENSP00000255380	ENSP00000255409	ENSP00000255531
ENSP00000255559	ENSP00000255688	ENSP00000256151
ENSP00000256178	ENSP00000256196	ENSP00000256216
ENSP00000256367	ENSP00000256398	ENSP00000256441
ENSP00000256442	ENSP00000256447	ENSP00000256458
ENSP00000256474	ENSP00000256592	ENSP00000256785
ENSP00000256797	ENSP00000256861	ENSP00000257034
ENSP00000257248	ENSP00000257572	ENSP00000257626
ENSP00000257765	ENSP00000257879	ENSP00000257974
ENSP00000258062	ENSP00000258145	ENSP00000258214
ENSP00000258385	ENSP00000258390	ENSP00000258436
ENSP00000258443	ENSP00000258457	ENSP00000258526
ENSP00000258530	ENSP00000258739	ENSP00000258930
ENSP00000258969	ENSP00000259008	ENSP00000259154
ENSP00000259206	ENSP00000259211	ENSP00000259213
ENSP00000259365	ENSP00000259569	ENSP00000259698
ENSP00000259727	ENSP00000259750	ENSP00000259895
ENSP00000260049	ENSP00000260128	ENSP00000260129
ENSP00000260130	ENSP00000260184	ENSP00000260364
ENSP00000260403	ENSP00000260447	ENSP00000260723
ENSP00000260731	ENSP00000260795	ENSP00000260818
ENSP00000261017	ENSP00000261023	ENSP00000261206
ENSP00000261233	ENSP00000261247	ENSP00000261349
ENSP00000261396	ENSP00000261435	ENSP00000261458
ENSP00000261590	ENSP00000261623	ENSP00000261681
ENSP00000261712	ENSP00000261713	ENSP00000261716
ENSP00000261739	ENSP00000261745	ENSP00000261778
ENSP00000261799	ENSP00000261826	ENSP00000261973
ENSP00000261991	ENSP00000262053	ENSP00000262055
ENSP00000262133	ENSP00000262144	ENSP00000262238
ENSP00000262300	ENSP00000262302	ENSP00000262315
ENSP00000262319	ENSP00000262383	ENSP00000262395
ENSP00000262502	ENSP00000262510	ENSP00000262519
ENSP00000262633	ENSP00000262659	ENSP00000262738

ENSP00000262820	ENSP00000262839	ENSP00000262861
ENSP00000262887	ENSP00000262919	ENSP00000263083
ENSP00000263093	ENSP00000263257	ENSP00000263273
ENSP00000263331	ENSP00000263346	ENSP00000263377
ENSP00000263401	ENSP00000263437	ENSP00000263461
ENSP00000263498	ENSP00000263576	ENSP00000263577
ENSP00000263579	ENSP00000263620	ENSP00000263657
ENSP00000263773	ENSP00000263795	ENSP00000263800
ENSP00000263921	ENSP00000263934	ENSP00000263985
ENSP00000264005	ENSP00000264029	ENSP00000264144
ENSP00000264218	ENSP00000264234	ENSP00000264245
ENSP00000264313	ENSP00000264344	ENSP00000264363
ENSP00000264399	ENSP00000264409	ENSP00000264499
ENSP00000264597	ENSP00000264668	ENSP00000264748
ENSP00000264784	ENSP00000264834	ENSP00000264893
ENSP00000264926	ENSP00000264972	ENSP00000264995
ENSP00000264998	ENSP00000265007	ENSP00000265029
ENSP00000265056	ENSP00000265071	ENSP00000265073
ENSP00000265093	ENSP00000265150	ENSP00000265162
ENSP00000265260	ENSP00000265294	ENSP00000265299
ENSP00000265310	ENSP00000265334	ENSP00000265382
ENSP00000265395	ENSP00000265428	ENSP00000265440
ENSP00000265627	ENSP00000265634	ENSP00000265689
ENSP00000265717	ENSP00000265723	ENSP00000265742
ENSP00000265773	ENSP00000265800	ENSP00000265801
ENSP00000265814	ENSP00000265840	ENSP00000265849
ENSP00000265960	ENSP00000266079	ENSP00000266524
ENSP00000266682	ENSP00000267102	ENSP00000267415
ENSP00000267422	ENSP00000267430	ENSP00000267803
ENSP00000267938	ENSP00000267973	ENSP00000268043
ENSP00000268281	ENSP00000268482	ENSP00000268595
ENSP00000268607	ENSP00000268613	ENSP00000268695
ENSP00000268763	ENSP00000268981	ENSP00000269122
ENSP00000269141	ENSP00000269221	ENSP00000269878
ENSP00000270538	ENSP00000270570	ENSP00000271532
ENSP00000271732	ENSP00000272065	ENSP00000272102
ENSP00000272133	ENSP00000272164	ENSP00000272342
ENSP00000272427	ENSP00000272647	ENSP00000273077
ENSP00000273130	ENSP00000273308	ENSP00000273317
ENSP00000273375	ENSP00000273905	ENSP00000273986
ENSP00000274008	ENSP00000274026	ENSP00000274137
ENSP00000274181	ENSP00000274345	ENSP00000274487
ENSP00000274496	ENSP00000274498	ENSP00000274625
ENSP00000274711	ENSP00000274712	ENSP00000274764

ENSP00000275300	ENSP00000275493	ENSP00000275605
ENSP00000275874	ENSP00000276110	ENSP00000276218
ENSP00000276373	ENSP00000276390	ENSP00000276594
ENSP00000276603	ENSP00000276816	ENSP00000276914
ENSP00000276943	ENSP00000277458	ENSP00000277575
ENSP00000278207	ENSP00000278360	ENSP00000278559
ENSP00000278612	ENSP00000278836	ENSP00000278865
ENSP00000278937	ENSP00000279022	ENSP00000279178
ENSP00000279230	ENSP00000279441	ENSP00000280155
ENSP00000280606	ENSP00000280614	ENSP00000281141
ENSP00000281156	ENSP00000281171	ENSP00000281474
ENSP00000282030	ENSP00000282223	ENSP00000282391
ENSP00000282516	ENSP00000282549	ENSP00000282606
ENSP00000283256	ENSP00000283303	ENSP00000283645
ENSP00000283752	ENSP00000283916	ENSP00000284031
ENSP00000284259	ENSP00000284425	ENSP00000284669
ENSP00000284719	ENSP00000284885	ENSP00000285208
ENSP00000285311	ENSP00000285402	ENSP00000285419
ENSP00000285735	ENSP00000285805	ENSP00000286031
ENSP00000286190	ENSP00000286614	ENSP00000287038
ENSP00000287380	ENSP00000287437	ENSP00000287667
ENSP00000287907	ENSP00000288139	ENSP00000289272
ENSP00000289805	ENSP00000289893	ENSP00000290075
ENSP00000290401	ENSP00000290552	ENSP00000290795
ENSP00000290866	ENSP00000291182	ENSP00000291560
ENSP00000291592	ENSP00000291700	ENSP00000291759
ENSP00000292180	ENSP00000292301	ENSP00000292363
ENSP00000292433	ENSP00000292778	ENSP00000292896
ENSP00000293405	ENSP00000293745	ENSP00000293826
ENSP00000293883	ENSP00000294119	ENSP00000294360
ENSP00000294753	ENSP00000295006	ENSP00000295206
ENSP00000295297	ENSP00000295321	ENSP00000295453
ENSP00000295566	ENSP00000295571	ENSP00000295624
ENSP00000295682	ENSP00000295683	ENSP00000295704
ENSP00000295727	ENSP00000295736	ENSP00000295757
ENSP00000295771	ENSP00000295901	ENSP00000295937
ENSP00000295962	ENSP00000295971	ENSP00000295974
ENSP00000296043	ENSP00000296051	ENSP00000296096
ENSP00000296266	ENSP00000296350	ENSP00000296412
ENSP00000296417	ENSP00000296420	ENSP00000296424
ENSP00000296452	ENSP00000296518	ENSP00000296589
ENSP00000296597	ENSP00000296733	ENSP00000296794
ENSP00000296824	ENSP00000296882	ENSP00000297107
ENSP00000297130	ENSP00000297135	ENSP00000297151

ENSP00000297163	ENSP00000297273	ENSP00000297354
ENSP00000297469	ENSP00000297512	ENSP00000297623
ENSP00000297625	ENSP00000297689	ENSP00000297784
ENSP00000297785	ENSP00000297792	ENSP00000297977
ENSP00000297988	ENSP00000298050	ENSP00000298129
ENSP00000298428	ENSP00000298527	ENSP00000298585
ENSP00000298694	ENSP00000298784	ENSP00000298832
ENSP00000299157	ENSP00000299424	ENSP00000299601
ENSP00000299626	ENSP00000299667	ENSP00000299687
ENSP00000299727	ENSP00000299759	ENSP00000299766
ENSP00000299824	ENSP00000299847	ENSP00000299866
ENSP00000300093	ENSP00000300134	ENSP00000300215
ENSP00000300289	ENSP00000300589	ENSP00000300738
ENSP00000300811	ENSP00000300900	ENSP00000300917
ENSP00000300961	ENSP00000301011	ENSP00000301030
ENSP00000301037	ENSP00000301050	ENSP00000301061
ENSP00000301263	ENSP00000301272	ENSP00000301420
ENSP00000301547	ENSP00000301656	ENSP00000301765
ENSP00000301819	ENSP00000301905	ENSP00000301917
ENSP00000301995	ENSP00000302037	ENSP00000302046
ENSP00000302120	ENSP00000302139	ENSP00000302239
ENSP00000302276	ENSP00000302289	ENSP00000302393
ENSP00000302569	ENSP00000302586	ENSP00000302665
ENSP00000302924	ENSP00000302936	ENSP00000302938
ENSP00000302994	ENSP00000303015	ENSP00000303434
ENSP00000303686	ENSP00000303709	ENSP00000303766
ENSP00000303844	ENSP00000303939	ENSP00000304006
ENSP00000304060	ENSP00000304283	ENSP00000304290
ENSP00000304308	ENSP00000304353	ENSP00000304376
ENSP00000304408	ENSP00000304593	ENSP00000304701
ENSP00000304875	ENSP00000304891	ENSP00000304945
ENSP00000305263	ENSP00000305288	ENSP00000305403
ENSP00000305502	ENSP00000305596	ENSP00000305682
ENSP00000305804	ENSP00000305839	ENSP00000305852
ENSP00000305906	ENSP00000305964	ENSP00000305973
ENSP00000306105	ENSP00000306220	ENSP00000306344
ENSP00000306397	ENSP00000306752	ENSP00000306894
ENSP00000306918	ENSP00000306974	ENSP00000307071
ENSP00000307096	ENSP00000307134	ENSP00000307183
ENSP00000307206	ENSP00000307387	ENSP00000307423
ENSP00000307525	ENSP00000307567	ENSP00000307666
ENSP00000307674	ENSP00000307889	ENSP00000307954
ENSP00000308208	ENSP00000308226	ENSP00000308270
ENSP00000308430	ENSP00000308461	ENSP00000308727

ENSP00000308741	ENSP00000308893	ENSP00000308901
ENSP00000308938	ENSP00000308957	ENSP00000309052
ENSP00000309096	ENSP00000309103	ENSP00000309186
ENSP00000309259	ENSP00000309365	ENSP00000309402
ENSP00000309432	ENSP00000309433	ENSP00000309463
ENSP00000309542	ENSP00000309606	ENSP00000309714
ENSP00000309751	ENSP00000309913	ENSP00000309953
ENSP00000309968	ENSP00000310006	ENSP00000310088
ENSP00000310275	ENSP00000310309	ENSP00000310321
ENSP00000310448	ENSP00000310547	ENSP00000310551
ENSP00000310568	ENSP00000310573	ENSP00000310623
ENSP00000310668	ENSP00000310770	ENSP00000310788
ENSP00000310796	ENSP00000310814	ENSP00000310966
ENSP00000310978	ENSP00000310998	ENSP00000311032
ENSP00000311121	ENSP00000311200	ENSP00000311202
ENSP00000311360	ENSP00000311479	ENSP00000311712
ENSP00000311713	ENSP00000311747	ENSP00000311833
ENSP00000311837	ENSP00000312042	ENSP00000312050
ENSP00000312066	ENSP00000312143	ENSP00000312150
ENSP00000312326	ENSP00000312370	ENSP00000312415
ENSP00000312624	ENSP00000312649	ENSP00000312664
ENSP00000312767	ENSP00000312988	ENSP00000313021
ENSP00000313046	ENSP00000313172	ENSP00000313199
ENSP00000313377	ENSP00000313506	ENSP00000313875
ENSP00000313890	ENSP00000314036	ENSP00000314343
ENSP00000314414	ENSP00000314508	ENSP00000314528
ENSP00000314606	ENSP00000314827	ENSP00000314901
ENSP00000315035	ENSP00000315182	ENSP00000315212
ENSP00000315614	ENSP00000315630	ENSP00000315644
ENSP00000315654	ENSP00000315674	ENSP00000315693
ENSP00000315774	ENSP00000315835	ENSP00000315931
ENSP00000316092	ENSP00000316222	ENSP00000316454
ENSP00000316905	ENSP00000316950	ENSP00000317121
ENSP00000317141	ENSP00000317144	ENSP00000317224
ENSP00000317232	ENSP00000317334	ENSP00000317357
ENSP00000317445	ENSP00000317564	ENSP00000317595
ENSP00000317790	ENSP00000317895	ENSP00000317905
ENSP00000318057	ENSP00000318115	ENSP00000318128
ENSP00000318147	ENSP00000318182	ENSP00000318629
ENSP00000318716	ENSP00000318770	ENSP00000318799
ENSP00000318869	ENSP00000318999	ENSP00000319170
ENSP00000319254	ENSP00000319388	ENSP00000319511
ENSP00000319531	ENSP00000319610	ENSP00000319622
ENSP00000319636	ENSP00000319984	ENSP00000319991

ENSP00000320038	ENSP00000320043	ENSP00000320081
ENSP00000320083	ENSP00000320246	ENSP00000320247
ENSP00000320303	ENSP00000320346	ENSP00000320349
ENSP00000320431	ENSP00000320503	ENSP00000320563
ENSP00000320604	ENSP00000320672	ENSP00000320709
ENSP00000320848	ENSP00000320886	ENSP00000321108
ENSP00000321133	ENSP00000321196	ENSP00000321346
ENSP00000321406	ENSP00000321449	ENSP00000321584
ENSP00000321594	ENSP00000321679	ENSP00000321951
ENSP00000322088	ENSP00000322191	ENSP00000322192
ENSP00000322234	ENSP00000322524	ENSP00000322568
ENSP00000322582	ENSP00000322617	ENSP00000322730
ENSP00000322915	ENSP00000322924	ENSP00000322991
ENSP00000323050	ENSP00000323099	ENSP00000323300
ENSP00000323328	ENSP00000323387	ENSP00000323424
ENSP00000323479	ENSP00000323568	ENSP00000323584
ENSP00000323663	ENSP00000323696	ENSP00000323720
ENSP00000323777	ENSP00000323811	ENSP00000323822
ENSP00000323837	ENSP00000323982	ENSP00000324438
ENSP00000324534	ENSP00000324549	ENSP00000324570
ENSP00000324651	ENSP00000324769	ENSP00000324857
ENSP00000324870	ENSP00000325296	ENSP00000325312
ENSP00000325506	ENSP00000325508	ENSP00000325526
ENSP00000325634	ENSP00000325738	ENSP00000326022
ENSP00000326031	ENSP00000326070	ENSP00000326238
ENSP00000326340	ENSP00000326519	ENSP00000326737
ENSP00000326817	ENSP00000326888	ENSP00000327070
ENSP00000327075	ENSP00000327133	ENSP00000327179
ENSP00000327197	ENSP00000327315	ENSP00000327336
ENSP00000327704	ENSP00000328103	ENSP00000328207
ENSP00000328287	ENSP00000328397	ENSP00000328472
ENSP00000328484	ENSP00000328511	ENSP00000328690
ENSP00000328747	ENSP00000328800	ENSP00000328818
ENSP00000329167	ENSP00000329219	ENSP00000329243
ENSP00000329357	ENSP00000329482	ENSP00000329499
ENSP00000329568	ENSP00000329715	ENSP00000329797
ENSP00000329930	ENSP00000329964	ENSP00000329991
ENSP00000330005	ENSP00000330031	ENSP00000330276
ENSP00000330284	ENSP00000330374	ENSP00000330382
ENSP00000330523	ENSP00000330572	ENSP00000330658
ENSP00000330694	ENSP00000330732	ENSP00000330813
ENSP00000330965	ENSP00000331106	ENSP00000331209
ENSP00000331242	ENSP00000331258	ENSP00000331572
ENSP00000331681	ENSP00000331746	ENSP00000331867

ENSP00000331938	ENSP00000332018	ENSP00000332052
ENSP00000332062	ENSP00000332123	ENSP00000332139
ENSP00000332163	ENSP00000332407	ENSP00000332444
ENSP00000332455	ENSP00000332576	ENSP00000332591
ENSP00000332613	ENSP00000332756	ENSP00000332818
ENSP00000332823	ENSP00000333018	ENSP00000333203
ENSP00000333537	ENSP00000333553	ENSP00000333638
ENSP00000333697	ENSP00000333744	ENSP00000334153
ENSP00000334216	ENSP00000334229	ENSP00000334280
ENSP00000334300	ENSP00000334308	ENSP00000334415
ENSP00000334441	ENSP00000334448	ENSP00000334474
ENSP00000334657	ENSP00000334681	ENSP00000334714
ENSP00000334910	ENSP00000334962	ENSP00000335083
ENSP00000335246	ENSP00000335306	ENSP00000335357
ENSP00000335486	ENSP00000335511	ENSP00000336655
ENSP00000336673	ENSP00000336801	ENSP00000337040
ENSP00000337056	ENSP00000337159	ENSP00000337194
ENSP00000337209	ENSP00000337265	ENSP00000337289
ENSP00000337313	ENSP00000337432	ENSP00000337452
ENSP00000337463	ENSP00000337733	ENSP00000337757
ENSP00000337838	ENSP00000337972	ENSP00000338050
ENSP00000338127	ENSP00000338141	ENSP00000338217
ENSP00000338293	ENSP00000338487	ENSP00000338510
ENSP00000338524	ENSP00000338533	ENSP00000338562
ENSP00000338613	ENSP00000338707	ENSP00000338727
ENSP00000339057	ENSP00000339115	ENSP00000339161
ENSP00000339208	ENSP00000339390	ENSP00000339449
ENSP00000339503	ENSP00000339769	ENSP00000339844
ENSP00000339861	ENSP00000339958	ENSP00000339960
ENSP00000340271	ENSP00000340278	ENSP00000340296
ENSP00000340328	ENSP00000340330	ENSP00000340347
ENSP00000340396	ENSP00000340409	ENSP00000340466
ENSP00000340474	ENSP00000340684	ENSP00000340691
ENSP00000340748	ENSP00000340761	ENSP00000340766
ENSP00000340811	ENSP00000340982	ENSP00000341083
ENSP00000341094	ENSP00000341213	ENSP00000341422
ENSP00000341489	ENSP00000341524	ENSP00000341539
ENSP00000341581	ENSP00000341698	ENSP00000341743
ENSP00000341756	ENSP00000341838	ENSP00000341942
ENSP00000342059	ENSP00000342071	ENSP00000342267
ENSP00000342374	ENSP00000342564	ENSP00000342623
ENSP00000342656	ENSP00000342812	ENSP00000342818
ENSP00000342952	ENSP00000343023	ENSP00000343054
ENSP00000343164	ENSP00000343244	ENSP00000343246

ENSP00000343248	ENSP00000343282	ENSP00000343325
ENSP00000343348	ENSP00000343430	ENSP00000343435
ENSP00000343464	ENSP00000343479	ENSP00000343557
ENSP00000343593	ENSP00000343617	ENSP00000343645
ENSP00000343706	ENSP00000343709	ENSP00000343742
ENSP00000343763	ENSP00000343765	ENSP00000343891
ENSP00000344223	ENSP00000344260	ENSP00000344352
ENSP00000344402	ENSP00000344446	ENSP00000344489
ENSP00000344545	ENSP00000344587	ENSP00000344749
ENSP00000344829	ENSP00000344976	ENSP00000344989
ENSP00000345023	ENSP00000345133	ENSP00000345156
ENSP00000345161	ENSP00000345193	ENSP00000345270
ENSP00000345333	ENSP00000345341	ENSP00000345344
ENSP00000345492	ENSP00000345532	ENSP00000345633
ENSP00000345681	ENSP00000345689	ENSP00000345772
ENSP00000345972	ENSP00000345974	ENSP00000346103
ENSP00000346127	ENSP00000346139	ENSP00000346206
ENSP00000346255	ENSP00000346256	ENSP00000346300
ENSP00000346316	ENSP00000346340	ENSP00000346453
ENSP00000346508	ENSP00000346566	ENSP00000346599
ENSP00000346644	ENSP00000346697	ENSP00000346800
ENSP00000346886	ENSP00000346913	ENSP00000346986
ENSP00000347045	ENSP00000347117	ENSP00000347152
ENSP00000347161	ENSP00000347314	ENSP00000347338
ENSP00000347339	ENSP00000347409	ENSP00000347444
ENSP00000347581	ENSP00000347710	ENSP00000347810
ENSP00000347872	ENSP00000348206	ENSP00000348215
ENSP00000348234	ENSP00000348258	ENSP00000348300
ENSP00000348302	ENSP00000348381	ENSP00000348394
ENSP00000348395	ENSP00000348401	ENSP00000348429
ENSP00000348449	ENSP00000348593	ENSP00000348634
ENSP00000348877	ENSP00000349078	ENSP00000349145
ENSP00000349205	ENSP00000349238	ENSP00000349270
ENSP00000349391	ENSP00000349393	ENSP00000349562
ENSP00000349595	ENSP00000349629	ENSP00000349709
ENSP00000349877	ENSP00000349929	ENSP00000349970
ENSP00000350003	ENSP00000350005	ENSP00000350017
ENSP00000350132	ENSP00000350195	ENSP00000350199
ENSP00000350263	ENSP00000350332	ENSP00000350716
ENSP00000350961	ENSP00000350976	ENSP00000351132
ENSP00000351325	ENSP00000351416	ENSP00000351605
ENSP00000351608	ENSP00000351684	ENSP00000351717
ENSP00000351727	ENSP00000351790	ENSP00000351813
ENSP00000351939	ENSP00000352021	ENSP00000352097

ENSP00000352101	ENSP00000352305	ENSP00000352665
ENSP00000352712	ENSP00000352770	ENSP00000352839
ENSP00000352842	ENSP00000352849	ENSP00000352989
ENSP00000353013	ENSP00000353078	ENSP00000353093
ENSP00000353094	ENSP00000353099	ENSP00000353142
ENSP00000353259	ENSP00000353491	ENSP00000353557
ENSP00000353575	ENSP00000353582	ENSP00000353652
ENSP00000353655	ENSP00000353656	ENSP00000353677
ENSP00000353731	ENSP00000353735	ENSP00000353940
ENSP00000354270	ENSP00000354293	ENSP00000354332
ENSP00000354340	ENSP00000354458	ENSP00000354522
ENSP00000354536	ENSP00000354541	ENSP00000354580
ENSP00000354689	ENSP00000354717	ENSP00000354826
ENSP00000354927	ENSP00000355010	ENSP00000355031
ENSP00000355050	ENSP00000355173	ENSP00000355177
ENSP00000355192	ENSP00000355195	ENSP00000355228
ENSP00000355396	ENSP00000355431	ENSP00000355556
ENSP00000355583	ENSP00000355799	ENSP00000355870
ENSP00000355920	ENSP00000355924	ENSP00000355958
ENSP00000356016	ENSP00000356105	ENSP00000356170
ENSP00000356251	ENSP00000356263	ENSP00000356370
ENSP00000356468	ENSP00000356473	ENSP00000356476
ENSP00000356536	ENSP00000356552	ENSP00000356563
ENSP00000356590	ENSP00000356674	ENSP00000356700
ENSP00000356737	ENSP00000356744	ENSP00000356811
ENSP00000356908	ENSP00000356918	ENSP00000356969
ENSP00000356975	ENSP00000356982	ENSP00000357033
ENSP00000357058	ENSP00000357060	ENSP00000357079
ENSP00000357086	ENSP00000357103	ENSP00000357152
ENSP00000357156	ENSP00000357189	ENSP00000357206
ENSP00000357278	ENSP00000357387	ENSP00000357461
ENSP00000357494	ENSP00000357555	ENSP00000357650
ENSP00000357674	ENSP00000357682	ENSP00000357683
ENSP00000357721	ENSP00000357726	ENSP00000357748
ENSP00000357775	ENSP00000357794	ENSP00000357796
ENSP00000357844	ENSP00000357920	ENSP00000358031
ENSP00000358033	ENSP00000358071	ENSP00000358147
ENSP00000358232	ENSP00000358242	ENSP00000358391
ENSP00000358417	ENSP00000358430	ENSP00000358470
ENSP00000358490	ENSP00000358510	ENSP00000358714
ENSP00000358719	ENSP00000358730	ENSP00000358777
ENSP00000358857	ENSP00000358888	ENSP00000358925
ENSP00000358966	ENSP00000359024	ENSP00000359070
ENSP00000359221	ENSP00000359234	ENSP00000359240

ENSP00000359258	ENSP00000359321	ENSP00000359329
ENSP00000359334	ENSP00000359356	ENSP00000359368
ENSP00000359370	ENSP00000359376	ENSP00000359380
ENSP00000359425	ENSP00000359490	ENSP00000359504
ENSP00000359520	ENSP00000359549	ENSP00000359594
ENSP00000359663	ENSP00000359685	ENSP00000359699
ENSP00000359719	ENSP00000359772	ENSP00000359795
ENSP00000359848	ENSP00000359899	ENSP00000359925
ENSP00000359942	ENSP00000359998	ENSP00000360147
ENSP00000360167	ENSP00000360217	ENSP00000360235
ENSP00000360269	ENSP00000360310	ENSP00000360360
ENSP00000360381	ENSP00000360412	ENSP00000360437
ENSP00000360441	ENSP00000360472	ENSP00000360488
ENSP00000360498	ENSP00000360519	ENSP00000360530
ENSP00000360806	ENSP00000360829	ENSP00000360905
ENSP00000360966	ENSP00000361005	ENSP00000361027
ENSP00000361095	ENSP00000361186	ENSP00000361331
ENSP00000361359	ENSP00000361373	ENSP00000361381
ENSP00000361423	ENSP00000361536	ENSP00000361544
ENSP00000361548	ENSP00000361672	ENSP00000361746
ENSP00000361843	ENSP00000361935	ENSP00000362095
ENSP00000362122	ENSP00000362207	ENSP00000362255
ENSP00000362283	ENSP00000362298	ENSP00000362399
ENSP00000362424	ENSP00000362465	ENSP00000362508
ENSP00000362524	ENSP00000362551	ENSP00000362590
ENSP00000362638	ENSP00000362748	ENSP00000362781
ENSP00000362978	ENSP00000362994	ENSP00000363019
ENSP00000363055	ENSP00000363071	ENSP00000363081
ENSP00000363108	ENSP00000363207	ENSP00000363329
ENSP00000363391	ENSP00000363392	ENSP00000363459
ENSP00000363590	ENSP00000363603	ENSP00000363647
ENSP00000363747	ENSP00000363787	ENSP00000363799
ENSP00000363970	ENSP00000364094	ENSP00000364246
ENSP00000364260	ENSP00000364270	ENSP00000364287
ENSP00000364501	ENSP00000364737	ENSP00000364749
ENSP00000364847	ENSP00000364858	ENSP00000364864
ENSP00000364883	ENSP00000364965	ENSP00000365081
ENSP00000365350	ENSP00000365465	ENSP00000365528
ENSP00000365624	ENSP00000365663	ENSP00000365757
ENSP00000365775	ENSP00000365844	ENSP00000365943
ENSP00000365962	ENSP00000366032	ENSP00000366234
ENSP00000366244	ENSP00000366273	ENSP00000366299
ENSP00000366487	ENSP00000366522	ENSP00000366525
ENSP00000366534	ENSP00000366563	ENSP00000366586

ENSP00000366593	ENSP00000366608	ENSP00000366679
ENSP00000366899	ENSP00000366939	ENSP00000366948
ENSP00000366999	ENSP00000367034	ENSP00000367172
ENSP00000367369	ENSP00000367453	ENSP00000367528
ENSP00000367570	ENSP00000367638	ENSP00000367664
ENSP00000367700	ENSP00000367766	ENSP00000367841
ENSP00000367891	ENSP00000367992	ENSP00000368062
ENSP00000368144	ENSP00000368169	ENSP00000368174
ENSP00000368237	ENSP00000368305	ENSP00000368350
ENSP00000368351	ENSP00000368391	ENSP00000368402
ENSP00000368459	ENSP00000368547	ENSP00000368589
ENSP00000368684	ENSP00000368748	ENSP00000368856
ENSP00000369129	ENSP00000369135	ENSP00000369146
ENSP00000369198	ENSP00000369251	ENSP00000369257
ENSP00000369380	ENSP00000369442	ENSP00000369473
ENSP00000369558	ENSP00000369568	ENSP00000369695
ENSP00000369725	ENSP00000369727	ENSP00000369739
ENSP00000369757	ENSP00000369784	ENSP00000369823
ENSP00000369895	ENSP00000370021	ENSP00000370031
ENSP00000370047	ENSP00000370150	ENSP00000370151
ENSP00000370191	ENSP00000370259	ENSP00000370316
ENSP00000370343	ENSP00000370391	ENSP00000370508
ENSP00000370639	ENSP00000370648	ENSP00000370766
ENSP00000370808	ENSP00000370826	ENSP00000370883
ENSP00000370990	ENSP00000371037	ENSP00000371070
ENSP00000371085	ENSP00000371232	ENSP00000371308
ENSP00000371432	ENSP00000371434	ENSP00000371471
ENSP00000371514	ENSP00000371886	ENSP00000371994
ENSP00000372155	ENSP00000372160	ENSP00000372199
ENSP00000372210	ENSP00000372292	ENSP00000372459
ENSP00000372599	ENSP00000372703	ENSP00000372723
ENSP00000372734	ENSP00000372746	ENSP00000372827
ENSP00000372857	ENSP00000372914	ENSP00000372930
ENSP00000372980	ENSP00000372991	ENSP00000373004
ENSP00000373031	ENSP00000373114	ENSP00000373586
ENSP00000373694	ENSP00000373698	ENSP00000373715
ENSP00000373730	ENSP00000373918	ENSP00000374205
ENSP00000374332	ENSP00000374448	ENSP00000374484
ENSP00000374813	ENSP00000374904	ENSP00000374944
ENSP00000374979	ENSP00000374989	ENSP00000375045
ENSP00000375086	ENSP00000375181	ENSP00000375598
ENSP00000375629	ENSP00000375783	ENSP00000375829
ENSP00000375844	ENSP00000375855	ENSP00000376188
ENSP00000376309	ENSP00000376333	ENSP00000376436

ENSP00000376553	ENSP00000376623	ENSP00000376652
ENSP00000376865	ENSP00000376871	ENSP00000377218
ENSP00000377233	ENSP00000377344	ENSP00000377558
ENSP00000377717	ENSP00000378161	ENSP00000378326
ENSP00000378517	ENSP00000378529	ENSP00000378786
ENSP00000378788	ENSP00000378792	ENSP00000379213
ENSP00000379616	ENSP00000379625	ENSP00000379669
ENSP00000379678	ENSP00000379865	ENSP00000379884
ENSP00000380025	ENSP00000380109	ENSP00000380413
ENSP00000380557	ENSP00000380635	ENSP00000380982
ENSP00000381086	ENSP00000381129	ENSP00000381148
ENSP00000381177	ENSP00000381227	ENSP00000381272
ENSP00000381504	ENSP00000381526	ENSP00000381553
ENSP00000381631	ENSP00000381840	ENSP00000381950
ENSP00000381951	ENSP00000382004	ENSP00000382026
ENSP00000382104	ENSP00000382166	ENSP00000382218
ENSP00000382274	ENSP00000382281	ENSP00000382373
ENSP00000382790	ENSP00000382875	ENSP00000383155
ENSP00000383360	ENSP00000383506	ENSP00000383521
ENSP00000383549	ENSP00000383611	ENSP00000383628
ENSP00000383900	ENSP00000383901	ENSP00000384169
ENSP00000384179	ENSP00000384313	ENSP00000384383
ENSP00000384398	ENSP00000384597	ENSP00000384675
ENSP00000384690	ENSP00000384708	ENSP00000384903
ENSP00000384979	ENSP00000385143	ENSP00000385215
ENSP00000385347	ENSP00000385899	ENSP00000385939
ENSP00000385995	ENSP00000386069	ENSP00000386092
ENSP00000386121	ENSP00000386161	ENSP00000386167
ENSP00000386174	ENSP00000386184	ENSP00000386222
ENSP00000386239	ENSP00000386502	ENSP00000386621
ENSP00000386717	ENSP00000386992	ENSP00000387040
ENSP00000387170	ENSP00000387252	ENSP00000387266
ENSP00000387278	ENSP00000387310	ENSP00000387412
ENSP00000388356	ENSP00000388631	ENSP00000388662
ENSP00000388779	ENSP00000388842	ENSP00000388987
ENSP00000389244	ENSP00000389427	ENSP00000389490
ENSP00000389630	ENSP00000389709	ENSP00000389792
ENSP00000389817	ENSP00000389913	ENSP00000390070
ENSP00000390134	ENSP00000390590	ENSP00000391069
ENSP00000391386	ENSP00000391402	ENSP00000391404
ENSP00000391457	ENSP00000391564	ENSP00000391638
ENSP00000391735	ENSP00000392466	ENSP00000392678
ENSP00000393198	ENSP00000393324	ENSP00000393335
ENSP00000393381	ENSP00000393393	ENSP00000393549

ENSP00000393631	ENSP00000393642	ENSP00000393751
ENSP00000393889	ENSP00000393958	ENSP00000394842
ENSP00000394863	ENSP00000395015	ENSP00000395225
ENSP00000395497	ENSP00000395656	ENSP00000395699
ENSP00000395780	ENSP00000396045	ENSP00000396152
ENSP00000396163	ENSP00000396732	ENSP00000397118
ENSP00000397140	ENSP00000397405	ENSP00000397636
ENSP00000397705	ENSP00000397759	ENSP00000397872
ENSP00000397971	ENSP00000398064	ENSP00000398103
ENSP00000398163	ENSP00000398342	ENSP00000398462
ENSP00000398671	ENSP00000398971	ENSP00000399011
ENSP00000399013	ENSP00000399078	ENSP00000399255
ENSP00000399565	ENSP00000400046	ENSP00000400157
ENSP00000400476	ENSP00000400867	ENSP00000401087
ENSP00000401177	ENSP00000401371	ENSP00000401513
ENSP00000401596	ENSP00000401786	ENSP00000401980
ENSP00000402038	ENSP00000402181	ENSP00000402343
ENSP00000402527	ENSP00000403246	ENSP00000403698
ENSP00000403721	ENSP00000403780	ENSP00000403802
ENSP00000404231	ENSP00000404306	ENSP00000404381
ENSP00000404416	ENSP00000404438	ENSP00000404539
ENSP00000405108	ENSP00000405176	ENSP00000405636
ENSP00000405738	ENSP00000405950	ENSP00000406271
ENSP00000406288	ENSP00000406378	ENSP00000406547
ENSP00000406706	ENSP00000406832	ENSP00000406933
ENSP00000407233	ENSP00000407375	ENSP00000407452
ENSP00000407460	ENSP00000407779	ENSP00000407885
ENSP00000407952	ENSP00000407978	ENSP00000407981
ENSP00000408017	ENSP00000408207	ENSP00000408395
ENSP00000408398	ENSP00000408451	ENSP00000408453
ENSP00000408581	ENSP00000408860	ENSP00000409285
ENSP00000409382	ENSP00000409403	ENSP00000409493
ENSP00000409912	ENSP00000410088	ENSP00000410098
ENSP00000410420	ENSP00000410758	ENSP00000411099
ENSP00000411115	ENSP00000411397	ENSP00000411471
ENSP00000411847	ENSP00000411949	ENSP00000411990
ENSP00000412031	ENSP00000412060	ENSP00000412309
ENSP00000412553	ENSP00000412800	ENSP00000413007
ENSP00000413009	ENSP00000413404	ENSP00000413405
ENSP00000413575	ENSP00000413625	ENSP00000413706
ENSP00000413949	ENSP00000413961	ENSP00000414302
ENSP00000414667	ENSP00000414777	ENSP00000414780
ENSP00000415106	ENSP00000415222	ENSP00000415464
ENSP00000415477	ENSP00000415769	ENSP00000415941

ENSP00000416095	ENSP00000416141	ENSP00000416293
ENSP00000416320	ENSP00000416753	ENSP00000417052
ENSP00000417147	ENSP00000417164	ENSP00000417257
ENSP00000417300	ENSP00000417492	ENSP00000417587
ENSP00000417601	ENSP00000417628	ENSP00000417748
ENSP00000417764	ENSP00000418001	ENSP00000418348
ENSP00000418447	ENSP00000418575	ENSP00000418649
ENSP00000418768	ENSP00000418823	ENSP00000418994
ENSP00000419371	ENSP00000419449	ENSP00000419923
ENSP00000420405	ENSP00000420517	ENSP00000420820
