# Supplementary material for: Gene Ontology and KEGG Enrichment Analyses of Genes Related to Age-Related Macular Degeneration
Source: Biomed Res Int. 2014 Aug 6;2014:450386. doi: 10.1155/2014/450386 (PMC4140130; doi:10.1155/2014/450386)
Supplement: Supplementary file 1 — The Supplementary Material contains five files. In detail, Supplementary Material I lists 39 known AMD related genes and 1,950 randomly selected genes; Supplementary Material II lists the output of mRMR program on each dataset; Supplementary Material III lists the accuracies obtained by IFS and SMO on each dataset; Supplementary Material IV lists the IFS curve on each dataset; Supplementary Material V lists the features in the final optimal feature set. [file 450386.f1.zip › Supp-II.pdf]

Supplementary Material II. The MaxRel features list and mRMR features list obtained by mRMR method for each dataset.

(1) Dataset D_1

a) MaxRel features list

Rank	Feature name
1	GO:0030449
2	hsa05150
3	GO:0001970
4	GO:0006956
5	GO:0043183
6	GO:0016495
7	GO:0071953
8	GO:0034436
9	GO:0034437
10	GO:0010001
11	GO:0072563
12	GO:0035645
13	GO:0034189
14	hsa04610
15	GO:0071482
16	GO:0001798
17	GO:1900006
18	GO:0048739
19	GO:0043537
20	GO:0043536
21	GO:0010711
22	GO:0010703
23	GO:0034103
24	GO:0060311
25	GO:0060313
26	GO:0021762
27	GO:0005172
28	GO:0051400
29	GO:0038025
30	GO:0004944
31	GO:0043117
32	GO:0002575
33	GO:0010764
34	GO:0006957
35	GO:0003273
36	GO:0014043

37	GO:0002462
38	GO:0004943
39	GO:0004876
40	GO:0001850
41	GO:0060319
42	GO:1900086
43	GO:0001938
44	GO:0060342
45	GO:0042253
46	GO:2000256
47	GO:0042231
48	GO:0031109
49	GO:0034346
50	GO:0042033
51	GO:0043331
52	GO:0060312
53	GO:0032930
54	GO:0038091
55	GO:0002042
56	GO:0046629
57	GO:0002429
58	GO:0034405
59	GO:0032958
60	GO:0000302
61	GO:0043184
62	GO:0042095
63	GO:0001300
64	GO:0042660
65	GO:0009409
66	GO:0006958
67	GO:0051651
68	GO:0032805
69	GO:0046911
70	GO:0060754
71	GO:0033627
72	GO:0034447
73	GO:0004866
74	GO:0000189
75	GO:0043498
76	GO:0032855
77	GO:0045766
78	GO:0051918
79	GO:0001540

80	GO:0032432
81	GO:0002551
82	GO:0034021
83	GO:0048844
84	GO:0001315
85	GO:0003069
86	GO:0048773
87	GO:0006940
88	GO:0002457
89	GO:0090037
90	GO:0030949
91	GO:0001910
92	GO:0002693
93	GO:0010477
94	GO:0048845
95	GO:0032809
96	GO:0008228
97	GO:0010863
98	GO:0051856
99	GO:0033364
100	GO:0004962
101	GO:0002282
102	GO:0021940
103	GO:0007497
104	GO:0008209
105	GO:0043171
106	GO:0045541
107	GO:0017158
108	GO:0045429
109	GO:0046813
110	GO:0060346
111	GO:0045103
112	GO:0042088
113	GO:0001968
114	GO:0000303
115	hsa05323
116	GO:0004784
117	GO:0019836
118	GO:0010716
119	GO:0038026
120	GO:0032463
121	GO:0050665
122	GO:0051389

123	GO:0014826
124	GO:0061302
125	GO:0031077
126	GO:0030214
127	GO:2001028
128	GO:0051142
129	GO:0071222
130	GO:0001974
131	GO:0048009
132	GO:0005534
133	GO:0032757
134	GO:0048678
135	GO:0070301
136	GO:0052033
137	GO:0007256
138	GO:0048245
139	GO:0009881
140	GO:0005615
141	GO:0030155
142	GO:0032387
143	GO:0033141
144	GO:0051898
145	GO:0005072
146	GO:0071062
147	GO:0030593
148	GO:0005178
149	GO:0007568
150	GO:0006700
151	GO:0050777
152	GO:0042159
153	GO:0051000
154	GO:0070206
155	GO:0051580
156	GO:0010625
157	GO:0044464
158	GO:0019430
159	GO:0001937
160	GO:0031017
161	GO:0060128
162	GO:0032270
163	GO:0030168
164	GO:0048246
165	GO:0042094

166	GO:0010165
167	GO:0051280
168	GO:0021517
169	GO:0019049
170	GO:0045869
171	GO:0007603
172	GO:0031714
173	GO:0010760
174	GO:0006029
175	GO:0045359
176	GO:0034185
177	GO:0042832
178	GO:0071346
179	GO:0042056
180	GO:0030229
181	GO:0042583
182	GO:0005161
183	GO:0010900
184	GO:0032368
185	GO:0018243
186	GO:0018242
187	GO:0034369
188	GO:0006741
189	GO:0004509
190	GO:0008360
191	GO:0060129
192	GO:0032467
193	GO:0061045
194	GO:0002291
195	GO:0014904
196	GO:0014896
197	GO:0009441
198	GO:0060020
199	GO:0034056
200	GO:0030247
201	GO:0071230
202	GO:0060394
203	GO:0010902
204	GO:0005576
205	GO:0009597
206	GO:0055093
207	GO:0032700
208	GO:0032722

209	GO:0045884
210	GO:0038052
211	GO:0060745
212	GO:0031798
213	GO:0022009
214	GO:0001569
215	GO:0043200
216	GO:0032417
217	GO:0010873
218	GO:0048598
219	GO:0071312
220	GO:0035630
221	GO:0051659
222	GO:0014909
223	GO:0060763
224	GO:0051926
225	GO:0033552
226	GO:0030195
227	GO:0048251
228	GO:0051712
229	GO:0033081
230	GO:0030284
231	GO:0035082
232	GO:0005044
233	GO:0042461
234	GO:0006705
235	GO:0060697
236	GO:0060644
237	GO:0046620
238	GO:0048386
239	GO:0005010
240	GO:0060591
241	GO:0030516
242	GO:0005006
243	GO:0042116
244	GO:0005548
245	GO:0042310
246	GO:0006801
247	GO:0006809
248	GO:0060228
249	GO:0070541
250	GO:0001957
251	GO:0045785

252	GO:0060325
253	GO:0009583
254	GO:0070022
255	GO:0060561
256	GO:0016056
257	GO:0001666
258	GO:0030308
259	GO:0010916
260	GO:0004252
261	GO:0060509
262	GO:0060011
263	GO:0050868
264	GO:0050689
265	GO:0043017
266	GO:0007181
267	GO:0033700
268	GO:0019064
269	GO:0060230
270	GO:0031102
271	GO:0070326
272	GO:0070483
273	GO:0033591
274	GO:0048661
275	GO:0060510
276	GO:0002262
277	GO:0046716
278	GO:0051894
279	GO:0000060
280	GO:0070371
281	GO:0060744
282	GO:0010987
283	GO:0004903
284	GO:0070195
285	GO:0004473
286	GO:0005125
287	GO:0043406
288	GO:0050679
289	GO:0034363
290	GO:0008217
291	GO:2000379
292	GO:0005114
293	GO:0010466
294	GO:0005586

295	hsa05143
296	GO:0071456
297	GO:0030838
298	GO:0003823
299	GO:0070670
300	GO:0007565
301	GO:0032732
302	GO:0070434
303	GO:0016046
304	GO:0070430
305	GO:0060265
306	GO:0030895
307	GO:0006649
308	GO:0060229
309	GO:0070383
310	GO:0032490
311	GO:0002232
312	GO:0014056
313	GO:0048525
314	GO:0035624
315	GO:0031093
316	GO:0048010
317	GO:0055094
318	GO:0051873
319	GO:0043154
320	GO:0015643
321	hsa05219
322	GO:0001763
323	GO:0048864
324	GO:0042092
325	GO:0002456
326	GO:0033160
327	GO:0050921
328	GO:0043325
329	GO:0010888
330	GO:0042325
331	GO:0051044
332	GO:0051881
333	GO:0046579
334	GO:0046668
335	GO:0034358
336	GO:0055090
337	GO:0010897

338	GO:0051005
339	GO:2000048
340	GO:0050840
341	GO:0016485
342	GO:0035924
343	GO:0071374
344	GO:0046010
345	GO:0014806
346	hsa05416
347	GO:0008395
348	GO:0010700
349	GO:0016493
350	GO:0038084
351	GO:0031999
352	GO:0090197
353	hsa05133
354	GO:0048565
355	GO:0004175
356	GO:0060351
357	GO:2001141
358	GO:0090031
359	GO:0060527
360	GO:0032943
361	GO:0032269
362	GO:0031953
363	GO:0003032
364	GO:0030279
365	GO:0051246
366	GO:0060707
367	GO:0002021
368	GO:0003340
369	GO:0090050
370	GO:0070373
371	GO:0034393
372	GO:0051607
373	GO:0051795
374	hsa04640
375	GO:0004517
376	GO:0006916
377	GO:0005520
378	GO:0072332
379	GO:0035458
380	GO:0042977

381	GO:0042060
382	GO:0031663
383	GO:0001934
384	GO:0005585
385	GO:0001502
386	GO:0043410
387	GO:2000502
388	GO:0002062
389	GO:0030023
390	GO:0035684
391	GO:0071347
392	GO:0010544
393	GO:0002523
394	GO:0085029
395	GO:0010763
396	GO:0032878
397	GO:0060729
398	GO:0030235
399	GO:0040007
400	GO:0042612
401	GO:0006527
402	GO:0002480
403	GO:0022617
404	GO:0032374
405	GO:0045078
406	GO:0019882
407	GO:0046434
408	GO:0010759
409	GO:0002687
410	GO:2000121
411	GO:0050995
412	GO:0045084
413	GO:0060068
414	GO:0061035
415	GO:0002446
416	GO:0072308
417	GO:0042698
418	GO:0042770
419	GO:0001955
420	GO:0002384
421	GO:0002532
422	GO:0034145
423	GO:0005130

424	hsa04672
425	GO:0042554
426	GO:0042637
427	GO:0043330
428	GO:0070050
429	GO:0070173
430	GO:0031012
431	GO:0019730
432	GO:0032897
433	GO:0001867
434	GO:0034154
435	GO:0033093
436	GO:0005579
437	GO:0060123
438	GO:0004950
439	GO:2001199
440	GO:2000663
441	GO:0032996
442	GO:2000666
443	GO:0034392
444	GO:2000309
445	GO:0071351
446	GO:0044146
447	GO:0060552
448	GO:0044429
449	GO:0060550
450	GO:0044444
451	GO:0071359
452	GO:0005540
453	GO:0030335
454	GO:0014065
455	hsa05310
456	GO:0002430
457	GO:0043206
458	GO:0060087
459	GO:0051272
460	GO:0006917
461	GO:0007606
462	GO:0048407
463	GO:0048536
464	hsa05146
465	GO:0071356
466	GO:0090265

467	GO:2000427
468	GO:0031727
469	GO:0010332
470	GO:0043627
471	GO:0045179
472	GO:0043066
473	GO:0048298
474	GO:0051152
475	GO:0021795
476	GO:0050927
477	GO:0014823
478	GO:0051054
479	GO:0051591
480	GO:0010224
481	GO:0042307
482	GO:0042511
483	GO:0030139
484	GO:0045445
485	GO:0016004
486	GO:0035767
487	GO:0032994
488	GO:0005597
489	GO:0034378
490	GO:0008513
491	GO:0034445
492	GO:0034365
493	GO:0065005
494	GO:0045343
495	GO:0031284
496	GO:0071674
497	GO:0002544
498	GO:0032819
499	GO:0005608
500	GO:0034701

b) mRMR features list

Rank	Feature name
1	GO:0030449
2	GO:0046629
3	GO:0043183
4	hsa05150
5	GO:0006956

6	GO:1900006
7	GO:0018243
8	GO:0002429
9	GO:0071482
10	GO:0048739
11	GO:0010001
12	hsa04610
13	GO:0006029
14	GO:0001970
15	GO:0050868
16	GO:0035645
17	GO:0002462
18	GO:0043184
19	GO:0034436
20	GO:0008228
21	GO:0004509
22	GO:0072563
23	GO:0006957
24	GO:0018242
25	GO:0051400
26	GO:0007603
27	GO:0004943
28	GO:0071953
29	GO:0038091
30	GO:0061302
31	GO:0006741
32	GO:0030214
33	GO:0006958
34	GO:0034437
35	GO:0004876
36	GO:0002042
37	GO:0000302
38	GO:0070206
39	GO:0004866
40	GO:0016495
41	GO:0006705
42	GO:0032994
43	GO:0001850
44	GO:0043537
45	GO:0072686
46	GO:0032463
47	GO:0005597
48	GO:0034405

49	GO:0004944
50	GO:0031616
51	GO:0034189
52	GO:0051918
53	GO:0042612
54	GO:0090037
55	GO:0033364
56	GO:0050689
57	GO:0004903
58	GO:0008395
59	GO:0001798
60	GO:1900086
61	GO:0031491
62	GO:0001540
63	GO:0035082
64	GO:0000003
65	GO:0010711
66	GO:0010900
67	GO:0060744
68	GO:0006940
69	GO:0002504
70	GO:0060346
71	GO:0032809
72	GO:0034378
73	GO:0043331
74	GO:0051873
75	GO:0071230
76	GO:0030247
77	GO:0060754
78	GO:0042461
79	GO:0032368
80	GO:0060342
81	GO:0014056
82	GO:0032930
83	GO:0004473
84	GO:0010369
85	GO:0010703
86	GO:0070195
87	GO:0060229
88	GO:0043536
89	GO:0000932
90	GO:0060265
91	GO:0001968

92	GO:0034369
93	GO:0034103
94	GO:0019064
95	GO:0005172
96	GO:0008200
97	GO:0051297
98	GO:0010165
99	GO:0005044
100	GO:0031109
101	GO:0006649
102	GO:0034445
103	GO:0003823
104	GO:0043117
105	GO:0033962
106	GO:0060311
107	GO:0051280
108	GO:0032417
109	GO:0009881
110	GO:0032490
111	GO:0019882
112	GO:0004784
113	GO:0034365
114	GO:0001867
115	GO:0014043
116	GO:0031714
117	GO:0065005
118	GO:0021762
119	GO:0072384
120	GO:0060313
121	GO:0090305
122	GO:0004653
123	GO:0042583
124	GO:2001199
125	GO:0001763
126	GO:0010760
127	GO:0038025
128	GO:0002575
129	GO:0002232
130	GO:0005582
131	GO:0014826
132	GO:0051537
133	GO:0003273
134	GO:0002480

135	GO:0010501
136	GO:0071062
137	GO:0008209
138	GO:0045869
139	GO:0090281
140	GO:0060394
141	GO:0034755
142	GO:0030621
143	GO:0033627
144	GO:0060319
145	GO:0010902
146	GO:0035270
147	GO:0010873
148	GO:2001141
149	GO:0003746
150	GO:0010987
151	GO:0017158
152	GO:0014909
153	GO:0047915
154	GO:0034346
155	GO:0015074
156	GO:0050777
157	GO:0005548
158	GO:0035624
159	GO:0043022
160	GO:0043171
161	GO:0045309
162	GO:0034056
163	GO:0031077
164	GO:0043024
165	GO:0007256
166	GO:0004441
167	GO:0038026
168	GO:0051635
169	GO:0034021
170	GO:0006438
171	GO:0010764
172	GO:0010821
173	GO:0032855
174	GO:0019049
175	GO:0046814
176	GO:0060697
177	GO:0007091

178	GO:0097186
179	GO:0071622
180	GO:0004962
181	GO:0072321
182	GO:0048009
183	GO:0005540
184	GO:0004832
185	GO:0043178
186	GO:0060011
187	GO:0034358
188	GO:0030895
189	GO:0006700
190	GO:0004221
191	GO:0042325
192	GO:0006930
193	GO:0032432
194	GO:0000930
195	GO:0030593
196	GO:0043064
197	GO:0003948
198	GO:0051856
199	GO:0038063
200	GO:0001315
201	GO:0009258
202	GO:0055090
203	GO:0019836
204	GO:0070722
205	GO:0070383
206	GO:0048640
207	GO:0030023
208	GO:0051926
209	GO:0031362
210	GO:0032990
211	GO:0001938
212	hsa00290
213	GO:0016619
214	GO:0034447
215	GO:0021858
216	GO:0001552
217	GO:0008347
218	GO:0005161
219	GO:0048525
220	GO:0060042

221	GO:0007497
222	GO:0045423
223	GO:0005579
224	GO:2001028
225	GO:0060228
226	GO:0035417
227	GO:0003069
228	GO:0006122
229	GO:0032958
230	GO:0035845
231	GO:0051654
232	GO:0032387
233	GO:0043498
234	GO:0070173
235	GO:0005488
236	GO:0009409
237	GO:0048243
238	GO:0060123
239	GO:0048386
240	GO:0010510
241	GO:0048845
242	GO:0046434
243	GO:0050884
244	GO:0045414
245	GO:0032510
246	GO:0042310
247	GO:0034185
248	GO:0070050
249	GO:0042253
250	GO:0005198
251	GO:0015030
252	GO:0005072
253	GO:0032393
254	GO:0008390
255	GO:0002551
256	GO:0050655
257	GO:0004252
258	GO:0034605
259	GO:0010863
260	GO:0010700
261	GO:0048773
262	GO:0070287
263	GO:0010388

264	GO:0004843
265	GO:0045103
266	GO:0003729
267	GO:0046548
268	GO:2000256
269	GO:0005876
270	GO:0002204
271	GO:0007606
272	GO:0007181
273	GO:0005608
274	GO:0000303
275	GO:0030949
276	GO:0035545
277	GO:0019730
278	GO:0005602
279	GO:0042660
280	GO:0008817
281	GO:0010716
282	GO:0008513
283	GO:0006809
284	GO:0016056
285	GO:0009236
286	GO:0048549
287	GO:0042159
288	GO:0001919
289	GO:0042231
290	GO:0051264
291	GO:0006527
292	GO:0002666
293	GO:0050840
294	GO:0046314
295	GO:0005534
296	hsa05416
297	GO:0000910
298	GO:0006311
299	GO:0051265
300	GO:0042033
301	GO:0032793
302	GO:0034875
303	GO:0002457
304	GO:0030382
305	hsa04962
306	GO:0003747

307	GO:0007080
308	GO:0032139
309	GO:0045618
310	GO:0030229
311	GO:0001300
312	GO:0043405
313	GO:0019835
314	GO:0006086
315	GO:0045541
316	GO:0001502
317	GO:0045116
318	GO:0060312
319	GO:0010916
320	GO:0045039
321	GO:0072332
322	GO:0042753
323	GO:0046649
324	GO:0052871
325	GO:0070052
326	GO:0034701
327	GO:0005930
328	GO:0001910
329	GO:0016209
330	GO:0038084
331	GO:0045179
332	GO:0005537
333	GO:0033141
334	GO:0005010
335	GO:0008428
336	GO:0008360
337	GO:0032996
338	GO:0016578
339	GO:0061035
340	GO:0050665
341	GO:0005585
342	GO:0045128
343	GO:0021517
344	GO:0055094
345	GO:0046605
346	GO:0055106
347	GO:0009597
348	GO:0033700
349	GO:0002729

350	GO:0005462
351	GO:0060510
352	GO:0031012
353	GO:0002693
354	GO:0031576
355	GO:0045591
356	GO:0006168
357	GO:0060745
358	GO:0046599
359	GO:0060230
360	GO:0043330
361	GO:0045494
362	GO:0016272
363	GO:0001974
364	GO:0030497
365	GO:0033116
366	GO:0001957
367	GO:0044241
368	GO:0030279
369	GO:0010477
370	GO:0015788
371	GO:0042462
372	GO:0051651
373	GO:0042719
374	GO:0060707
375	GO:0008187
376	GO:0021772
377	GO:0005586
378	GO:0060128
379	GO:2000405
380	GO:0021885
381	GO:0060052
382	GO:0033188
383	GO:0002282
384	GO:0002430
385	GO:0031573
386	GO:0047493
387	GO:0051898
388	GO:0030145
389	GO:0001917
390	GO:0048306
391	GO:0061338
392	GO:0003340

393	GO:0032805
394	GO:0051272
395	GO:0019509
396	GO:1900016
397	GO:0060351
398	GO:0045884
399	GO:0019543
400	GO:0042116
401	GO:0021678
402	GO:0021940
403	GO:0010826
404	GO:0031134
405	hsa05323
406	GO:0031701
407	GO:0001865
408	GO:0019430
409	GO:0042761
410	GO:0060068
411	GO:0031867
412	GO:0060588
413	GO:0001937
414	GO:0032812
415	GO:0005604
416	GO:0010897
417	GO:0051438
418	GO:0001952
419	GO:0042095
420	GO:0071569
421	GO:0051101
422	GO:0003416
423	GO:0047547
424	GO:0010972
425	GO:0030682
426	GO:0016493
427	GO:0070253
428	GO:0046911
429	GO:0009583
430	GO:0070576
431	GO:0003032
432	GO:0003100
433	GO:0051894
434	GO:0051148
435	GO:0015966

436	GO:0010466
437	GO:0001848
438	GO:0071253
439	GO:0042637
440	GO:0030284
441	GO:0006888
442	GO:0032633
443	GO:0015643
444	GO:0085029
445	GO:0046904
446	GO:0061304
447	GO:0050432
448	GO:0071329
449	GO:0042770
450	GO:0002262
451	GO:0051536
452	GO:0051389
453	GO:0030529
454	GO:0004012
455	GO:0034154
456	GO:0032374
457	GO:0071638
458	GO:0048245
459	GO:0030496
460	GO:2000379
461	GO:0019367
462	GO:0050995
463	GO:0048844
464	GO:0042393
465	GO:0034145
466	GO:0052872
467	GO:0090230
468	GO:0005591
469	GO:0072308
470	GO:0032304
471	GO:0043017
472	GO:0055087
473	GO:0050327
474	GO:0031061
475	GO:0032691
476	GO:0060561
477	GO:0045359
478	GO:0060621

479	GO:0005114
480	GO:0060242
481	GO:0008612
482	GO:0060129
483	GO:0060345
484	GO:0033165
485	GO:0016603
486	GO:0032943
487	GO:0000189
488	GO:2000323
489	GO:0046010
490	GO:0016554
491	GO:0035767
492	GO:0006108
493	GO:0005381
494	GO:0016485
495	GO:0034363
496	GO:0030155
497	GO:0005881
498	GO:0032270
499	GO:0017186
500	GO:0004333

(2) Dataset D_2

a) MaxRel features list

Rank	Feature name
1	GO:0071482
2	GO:0071953
3	GO:0030449
4	GO:0001970
5	GO:0016495
6	GO:0010711
7	GO:0034103
8	GO:0010703
9	GO:0060311
10	GO:0060313
11	GO:0034189
12	GO:0035645
13	hsa05150
14	GO:0043537
15	GO:0051400
16	GO:0031109

17	GO:0034437
18	GO:0034436
19	GO:0048678
20	GO:0060342
21	GO:0003273
22	GO:0072563
23	GO:0032855
24	GO:0004962
25	GO:0034447
26	GO:0014043
27	GO:0043536
28	GO:0001315
29	GO:0003069
30	GO:0034021
31	GO:0048773
32	hsa04610
33	GO:0032930
34	GO:0001798
35	GO:2001028
36	GO:0043183
37	GO:0004784
38	GO:0051389
39	GO:0032467
40	GO:0032432
41	GO:0038025
42	GO:0005172
43	GO:0060052
44	GO:0010001
45	GO:0045541
46	GO:0031012
47	GO:0004944
48	GO:0005072
49	GO:0021762
50	GO:0006957
51	GO:0043331
52	GO:0034346
53	GO:0048245
54	GO:0043206
55	GO:0000302
56	GO:0046911
57	GO:0032805
58	GO:0051651
59	GO:0042095

60	GO:0001938
61	GO:0042159
62	GO:0000303
63	GO:0045103
64	GO:0002551
65	GO:0051856
66	GO:0060319
67	GO:0048844
68	GO:0042660
69	GO:0043117
70	GO:0002575
71	GO:0051580
72	GO:2000256
73	GO:0007256
74	GO:0042253
75	GO:0042231
76	GO:0014826
77	GO:0042033
78	GO:0010625
79	GO:0070483
80	GO:0044464
81	GO:0002282
82	GO:0050777
83	GO:0048598
84	GO:1900086
85	GO:0060011
86	hsa05323
87	GO:0032809
88	GO:0042088
89	GO:0045766
90	GO:0051142
91	GO:0060346
92	GO:0006956
93	GO:0038026
94	GO:0005615
95	GO:0005520
96	GO:0070371
97	GO:0002693
98	GO:0001910
99	GO:0010477
100	GO:0002457
101	GO:0050665
102	GO:0005586

103	GO:0032488
104	GO:0033364
105	GO:0033141
106	GO:0001300
107	GO:0032700
108	GO:0001957
109	GO:0003084
110	GO:0048845
111	GO:0051894
112	GO:0005534
113	GO:0090031
114	GO:1900006
115	GO:0007603
116	GO:0006958
117	GO:0042612
118	GO:0008360
119	GO:0052033
120	GO:0042832
121	GO:0060020
122	GO:0046813
123	GO:0051898
124	GO:0009409
125	GO:0045429
126	GO:0060744
127	GO:0002042
128	GO:0072332
129	GO:0051918
130	GO:0002462
131	GO:0004943
132	GO:0004876
133	GO:0034185
134	GO:0001850
135	GO:0071222
136	GO:0001968
137	GO:0050921
138	GO:0010466
139	GO:0051881
140	GO:0005114
141	GO:0051607
142	GO:0004866
143	GO:0008395
144	GO:0048739
145	GO:0008209

146	GO:0051000
147	GO:0070050
148	GO:0071346
149	GO:0033591
150	GO:0008228
151	GO:0034393
152	GO:0003032
153	GO:0031017
154	GO:0048246
155	GO:0016056
156	GO:0009583
157	GO:0000189
158	GO:0033160
159	GO:0031077
160	GO:0010760
161	GO:0031714
162	GO:0042461
163	GO:0035082
164	GO:0032784
165	GO:0042583
166	GO:0048251
167	GO:0032463
168	GO:0030229
169	GO:0030195
170	GO:0019430
171	GO:0004509
172	GO:0070633
173	GO:0070091
174	GO:0002384
175	GO:0060527
176	GO:0001895
177	GO:0001937
178	GO:0051712
179	GO:0003340
180	GO:0070301
181	GO:0019882
182	GO:0034363
183	GO:0007179
184	GO:0032757
185	GO:0017158
186	GO:0019836
187	GO:0030214
188	GO:0002262

189	GO:0009441
190	GO:0014896
191	GO:0014904
192	GO:0033081
193	GO:0043498
194	GO:0007568
195	GO:0032958
196	GO:0048386
197	GO:0021940
198	GO:0046620
199	GO:0005010
200	GO:0030023
201	GO:0006029
202	GO:0032816
203	GO:0006917
204	GO:0060745
205	GO:0005579
206	GO:0055087
207	GO:0004906
208	GO:0002429
209	GO:0046629
210	GO:0035630
211	GO:0010763
212	GO:0016942
213	GO:0060510
214	GO:0001781
215	GO:0008090
216	GO:0043200
217	GO:0085029
218	GO:0046716
219	GO:0030516
220	GO:0006801
221	GO:0042060
222	GO:0009597
223	GO:0043381
224	GO:0032003
225	GO:0052871
226	GO:0070524
227	hsa05310
228	GO:0035624
229	GO:0048535
230	GO:0001666
231	GO:0015485

232	GO:0010902
233	GO:0060545
234	GO:0070926
235	GO:0060552
236	GO:2000309
237	GO:0060550
238	GO:0071351
239	GO:0044146
240	GO:0060509
241	GO:0004950
242	GO:0007181
243	GO:0033299
244	GO:0048661
245	GO:0043171
246	GO:0021517
247	GO:0031798
248	GO:0072308
249	GO:0038052
250	GO:0030346
251	GO:0006700
252	GO:0045348
253	GO:0010764
254	GO:0032387
255	GO:0004473
256	GO:0006741
257	GO:0030838
258	GO:0010165
259	GO:0002523
260	hsa05416
261	GO:0036094
262	GO:0042310
263	GO:0001974
264	GO:0045188
265	GO:0042094
266	GO:0042056
267	GO:0010224
268	GO:0031701
269	GO:0032691
270	GO:0001569
271	GO:0030235
272	GO:0070430
273	GO:0045079
274	GO:0032732

275	GO:0070434
276	GO:0016046
277	GO:0072606
278	GO:0060229
279	GO:0070383
280	GO:0071253
281	GO:0022617
282	GO:0006935
283	GO:0070373
284	GO:0045906
285	GO:0050927
286	GO:0035767
287	GO:0043154
288	GO:0042092
289	GO:0071356
290	GO:0051098
291	GO:0009408
292	GO:0090037
293	GO:0007497
294	GO:0002021
295	GO:0002291
296	GO:0060087
297	GO:0010951
298	GO:0034358
299	GO:0060697
300	GO:0055090
301	GO:0019064
302	GO:0005578
303	GO:0071360
304	GO:0060228
305	GO:0005178
306	GO:0005576
307	GO:0042511
308	GO:0035684
309	GO:0005548
310	GO:0010544
311	GO:0010332
312	GO:2000502
313	GO:0008061
314	GO:0010700
315	GO:0032510
316	GO:0060561
317	GO:0010863

318	GO:0071813
319	GO:0032497
320	GO:0060394
321	GO:0042325
322	GO:0045109
323	GO:0043388
324	GO:0051246
325	GO:0060740
326	GO:0046688
327	GO:0060137
328	GO:0031102
329	GO:0070326
330	GO:0035924
331	GO:0060351
332	GO:0048009
333	GO:0002446
334	GO:0055093
335	GO:0014012
336	GO:0060068
337	GO:0051054
338	GO:0060763
339	GO:0051045
340	GO:0015643
341	GO:0006290
342	GO:0019835
343	GO:0051873
344	GO:0031093
345	GO:0001540
346	GO:0010512
347	GO:0019049
348	GO:0035458
349	GO:0048407
350	GO:0007183
351	GO:0032332
352	hsa05146
353	GO:0005125
354	GO:0006704
355	GO:0031663
356	GO:0007184
357	GO:0030595
358	GO:0038091
359	GO:0031953
360	GO:0030279

361	GO:0045742
362	GO:0060750
363	GO:0051280
364	GO:0005896
365	GO:0060754
366	GO:0048549
367	GO:0002756
368	GO:0072255
369	GO:0072126
370	GO:0031232
371	GO:0045869
372	GO:0042554
373	GO:2000591
374	GO:0072264
375	GO:0035663
376	GO:2000340
377	GO:0032738
378	GO:0035665
379	GO:0035662
380	GO:0010875
381	GO:0010804
382	GO:0030308
383	GO:0009314
384	GO:0060047
385	GO:0002218
386	GO:0014911
387	GO:0060312
388	GO:0007565
389	GO:0007569
390	hsa05133
391	GO:0060559
392	GO:0009817
393	GO:0002513
394	GO:0060751
395	GO:0002460
396	GO:1900126
397	GO:0034056
398	GO:0048185
399	GO:0034188
400	GO:0030199
401	GO:0038027
402	GO:0002790
403	GO:0005581

404	GO:0070173
405	GO:0034382
406	GO:0002504
407	GO:0034141
408	GO:0001848
409	GO:0042627
410	GO:0034137
411	GO:0032269
412	GO:0032417
413	GO:0010718
414	GO:0014065
415	GO:0008201
416	GO:0030512
417	GO:0014834
418	GO:0061045
419	GO:0060591
420	GO:0043066
421	hsa05142
422	GO:0090265
423	GO:0007263
424	GO:0010716
425	GO:0034616
426	GO:0032494
427	GO:0010940
428	GO:0061302
429	GO:0007021
430	GO:0030949
431	GO:0048146
432	GO:0031727
433	GO:0031702
434	GO:0002227
435	GO:0030879
436	GO:0010742
437	GO:0060644
438	GO:0030247
439	GO:0070022
440	GO:0051152
441	GO:0042698
442	GO:0051271
443	GO:0046907
444	GO:0006940
445	GO:0033627
446	GO:0045084

447	GO:0000254
448	GO:0034365
449	GO:0016554
450	GO:0033781
451	GO:0032994
452	GO:0034445
453	GO:0031616
454	GO:0051262
455	GO:0001917
456	GO:0015920
457	GO:0071638
458	GO:0030169
459	GO:0051216
460	GO:0070098
461	GO:0007250
462	GO:0040007
463	GO:0015811
464	GO:0015184
465	GO:2000349
466	GO:0048525
467	GO:0035519
468	GO:0070429
469	GO:0061043
470	GO:0002033
471	GO:0034148
472	GO:0030895
473	GO:0070433
474	GO:0001730
475	GO:0045824
476	GO:0072573
477	GO:0034392
478	GO:0008089
479	GO:0002302
480	hsa05164
481	GO:0009611
482	GO:0005539
483	GO:0050829
484	GO:0033145
485	GO:0007435
486	GO:0005584
487	GO:0050795
488	GO:0030185
489	GO:0032496

490	GO:0030828
491	GO:0070886
492	GO:0005006
493	GO:0022009
494	GO:0019934
495	GO:0010759
496	GO:0051044
497	GO:0051926
498	GO:0004525
499	GO:0002035
500	GO:0045953

b) mRMR features list

Rank	Feature name
1	GO:0071482
2	GO:0043537
3	hsa05150
4	GO:0042612
5	GO:0034189
6	GO:0030449
7	GO:0048739
8	GO:0008395
9	GO:0071953
10	GO:0050777
11	GO:0060052
12	GO:0031012
13	GO:0001970
14	GO:0070524
15	GO:0031616
16	GO:0016495
17	GO:0007603
18	GO:2001028
19	hsa04610
20	GO:0002429
21	GO:0035645
22	GO:0060744
23	GO:0010711
24	GO:0004509
25	GO:0051683
26	GO:0051400
27	GO:0006957
28	GO:0072563
29	GO:0019882

30	GO:0042461
31	GO:0034103
32	GO:0060229
33	GO:0005586
34	GO:0006956
35	GO:0051894
36	GO:0070050
37	GO:0004944
38	GO:0048678
39	GO:0048535
40	GO:0010703
41	GO:0055087
42	GO:0005520
43	GO:0043022
44	GO:0033364
45	GO:0060342
46	GO:0035082
47	GO:0034437
48	GO:0046629
49	GO:0005172
50	GO:0060311
51	GO:0072606
52	GO:0010001
53	GO:0042490
54	GO:0008228
55	GO:0001315
56	GO:0004906
57	GO:0032930
58	GO:0014826
59	GO:0015485
60	GO:0060313
61	GO:0004866
62	GO:0000254
63	GO:0060011
64	GO:0007179
65	GO:0001798
66	GO:0004962
67	GO:0002462
68	GO:0038063
69	GO:0030023
70	GO:0032463
71	GO:0034447
72	GO:0031362

73	GO:0043405
74	GO:0060319
75	GO:0031109
76	GO:0070584
77	GO:0060346
78	GO:0010760
79	GO:0032855
80	GO:0031701
81	GO:0034365
82	GO:0000003
83	GO:0005114
84	GO:0006958
85	GO:0003069
86	GO:0004943
87	GO:0043206
88	GO:0002504
89	GO:0048598
90	GO:0072384
91	GO:0038025
92	GO:0043381
93	GO:0016554
94	GO:0004473
95	GO:0070633
96	GO:0031714
97	GO:0032003
98	GO:0085029
99	GO:0000302
100	GO:0032994
101	GO:0003273
102	GO:0032691
103	GO:0034436
104	GO:0006741
105	GO:0005581
106	GO:0021678
107	GO:0014043
108	GO:0034445
109	GO:0004876
110	GO:0034021
111	GO:0007183
112	GO:0017158
113	GO:0043117
114	GO:0072686
115	GO:0007181

116	GO:0043024
117	GO:0043331
118	GO:0006029
119	GO:0042583
120	GO:0001850
121	GO:0043171
122	GO:0045103
123	GO:0010821
124	GO:0042159
125	GO:0052871
126	GO:0043183
127	GO:0035624
128	GO:0048773
129	GO:0000268
130	GO:0051918
131	GO:0015811
132	GO:0007184
133	GO:0090281
134	GO:0038026
135	GO:0043536
136	GO:0016056
137	GO:0000152
138	GO:0006704
139	GO:0072332
140	GO:0051873
141	GO:0051389
142	GO:0071253
143	GO:0015184
144	GO:0033299
145	GO:0004784
146	GO:0071472
147	GO:0035767
148	GO:0007256
149	GO:0019799
150	GO:0001957
151	GO:0010826
152	GO:0033781
153	GO:0032809
154	GO:0005488
155	GO:0051607
156	GO:1900086
157	GO:0001917
158	GO:0032488

159	GO:0008106
160	GO:0005579
161	GO:0006168
162	GO:0032467
163	GO:0032395
164	GO:0006700
165	GO:0051856
166	GO:0030950
167	GO:0030497
168	GO:0048245
169	GO:1900006
170	GO:0030185
171	GO:0035845
172	GO:0001968
173	GO:0055090
174	GO:0010466
175	GO:0038062
176	GO:0032432
177	GO:0006323
178	GO:0007063
179	GO:0005072
180	GO:0042310
181	GO:0048549
182	GO:0031077
183	GO:0071503
184	GO:0045541
185	GO:0045953
186	GO:0019835
187	GO:0009583
188	GO:0051881
189	GO:0010747
190	GO:0002551
191	GO:0008187
192	GO:0050921
193	GO:0015074
194	GO:0071622
195	GO:0000303
196	GO:0010902
197	GO:0042613
198	GO:0008209
199	GO:0090321
200	GO:0035254
201	GO:0034346

202	GO:0000910
203	GO:0010923
204	GO:0097186
205	GO:0032784
206	GO:0001867
207	hsa05416
208	GO:0032816
209	GO:0045824
210	GO:0010763
211	GO:0003084
212	GO:0035473
213	GO:0001552
214	GO:2001199
215	GO:0008360
216	GO:0034358
217	GO:0002033
218	hsa00450
219	GO:0051148
220	GO:0002575
221	GO:0034185
222	GO:0005537
223	GO:0070483
224	GO:0070206
225	GO:0035478
226	GO:0015079
227	GO:0021762
228	GO:0006108
229	GO:0050655
230	GO:0046599
231	GO:0071248
232	GO:0051098
233	GO:0051580
234	GO:0008817
235	GO:0052723
236	GO:0032417
237	GO:0010700
238	GO:0019064
239	GO:0008428
240	GO:0001537
241	GO:0046878
242	GO:0002042
243	GO:0017038
244	GO:0010369

245	GO:0010625
246	GO:0051635
247	GO:0070173
248	GO:0003032
249	GO:0006705
250	GO:0060137
251	GO:0046548
252	GO:0034363
253	GO:0060545
254	GO:0090207
255	GO:0020003
256	hsa05323
257	GO:0032510
258	GO:0001895
259	GO:2000349
260	GO:0000832
261	GO:0044464
262	GO:0048025
263	GO:0042761
264	GO:0034393
265	GO:0060697
266	GO:0033591
267	GO:0071241
268	GO:0001561
269	GO:0070383
270	GO:0060527
271	GO:0010165
272	GO:0033116
273	GO:0016817
274	GO:0005534
275	GO:0007387
276	GO:0060510
277	GO:0002032
278	GO:0002282
279	GO:0020005
280	GO:0001730
281	GO:0002262
282	GO:0000293
283	GO:0048386
284	GO:0051271
285	GO:0060699
286	GO:0046911
287	GO:0070429

288	GO:0008747
289	GO:0021509
290	GO:0007388
291	GO:0048844
292	GO:0035377
293	GO:0052724
294	GO:0031531
295	GO:0042660
296	GO:0060806
297	GO:2001051
298	GO:0061043
299	GO:0019262
300	GO:0070926
301	GO:0060068
302	GO:0050795
303	GO:0030184
304	GO:0007413
305	GO:0070371
306	GO:0019166
307	GO:0032805
308	GO:0030247
309	GO:0044314
310	GO:0016619
311	GO:0046813
312	GO:0005876
313	GO:0085018
314	GO:0001937
315	GO:0016603
316	GO:0060020
317	GO:0060123
318	GO:0030229
319	GO:0008630
320	GO:0034148
321	GO:0030199
322	GO:0010804
323	GO:0007606
324	GO:0050665
325	GO:0060047
326	GO:0005930
327	GO:0002523
328	GO:0032700
329	GO:0035378
330	GO:0003334

331	GO:0022617
332	GO:0021772
333	GO:0030214
334	GO:0070433
335	GO:0051651
336	GO:0006601
337	GO:0008061
338	GO:0004470
339	GO:0008090
340	GO:0035379
341	GO:0017186
342	GO:0005041
343	GO:0005010
344	GO:0045964
345	GO:0072573
346	GO:0001848
347	GO:0009881
348	GO:0035519
349	GO:0042832
350	GO:0006931
351	GO:0021681
352	GO:0090031
353	GO:0045906
354	GO:0033145
355	GO:0032990
356	GO:0019049
357	GO:0004525
358	GO:0060228
359	GO:0055106
360	GO:0019836
361	GO:0051988
362	GO:0048845
363	GO:0043178
364	GO:0034122
365	GO:0015914
366	GO:0001938
367	GO:0006576
368	GO:0051782
369	GO:0060351
370	GO:0043064
371	GO:0010951
372	GO:2000256
373	GO:0050884

374	GO:0034451
375	GO:0051224
376	GO:0004631
377	GO:0048251
378	GO:0031752
379	GO:0051142
380	GO:0045332
381	GO:0046814
382	GO:0050927
383	GO:0002480
384	GO:0006353
385	GO:0042325
386	GO:0042627
387	GO:0035455
388	GO:0048246
389	GO:0030279
390	GO:0015828
391	GO:0051898
392	GO:0016500
393	GO:0046907
394	GO:0006290
395	GO:0015643
396	GO:0032332
397	GO:0005302
398	GO:0042095
399	GO:0001578
400	GO:0051654
401	GO:0034140
402	GO:0019430
403	GO:0030169
404	GO:0003747
405	GO:0060394
406	GO:0061302
407	GO:0051345
408	GO:0007130
409	GO:0004950
410	hsa01040
411	GO:0010189
412	GO:0042253
413	GO:0005578
414	GO:0010519
415	GO:0003777
416	GO:0046904

417	GO:0005883
418	GO:0004301
419	GO:0005588
420	GO:0051262
421	GO:0042978
422	GO:0042231
423	GO:0036002
424	GO:0050689
425	GO:0021517
426	GO:0010523
427	GO:0002513
428	GO:2001179
429	GO:0035663
430	GO:0016559
431	GO:0060745
432	GO:0008154
433	GO:0038161
434	GO:0042033
435	GO:0032673
436	GO:0043654
437	GO:0006940
438	GO:0071360
439	GO:0032226
440	GO:0019367
441	GO:0051272
442	GO:0036094
443	GO:0009586
444	GO:0034382
445	GO:0031573
446	GO:0002693
447	GO:0030346
448	GO:0032354
449	GO:0030595
450	GO:0004925
451	GO:0031232
452	GO:2000340
453	GO:0031702
454	GO:0043159
455	GO:0001605
456	GO:0006710
457	GO:0045046
458	GO:0042088
459	GO:0044324

460	GO:0070530
461	GO:0071329
462	GO:0004012
463	GO:0006975
464	GO:0002767
465	GO:0061299
466	GO:0001910
467	GO:0070195
468	GO:0003828
469	GO:0032738
470	GO:0090037
471	GO:0043422
472	GO:0016557
473	GO:0005539
474	GO:0071209
475	GO:0071638
476	GO:0033141
477	hsa03013
478	GO:0007497
479	GO:0014056
480	GO:0050262
481	GO:0047547
482	GO:0005591
483	GO:0060750
484	GO:0035665
485	GO:0007527
486	GO:0001778
487	GO:0008299
488	GO:0045104
489	GO:0010477
490	GO:0004422
491	GO:0051438
492	GO:0050765
493	GO:0004903
494	GO:0046620
495	GO:0042752
496	GO:0071813
497	GO:0002820
498	GO:0019543
499	GO:2000402
500	GO:0001300

(3) Dataset D_3

a) MaxRel features list

Rank	Feature name
1	GO:0030449
2	GO:0071482
3	hsa04610
4	GO:0060342
5	GO:0004866
6	GO:0072563
7	GO:0034437
8	GO:0034436
9	GO:0043537
10	hsa05150
11	GO:0003273
12	GO:0043536
13	GO:2001028
14	GO:0006956
15	GO:0010466
16	GO:0034189
17	GO:0048845
18	GO:0002575
19	GO:0048844
20	GO:0032757
21	GO:0043183
22	GO:0016495
23	GO:0035645
24	GO:0042088
25	GO:0034447
26	GO:0051389
27	GO:0071953
28	GO:0001300
29	GO:0001970
30	GO:0021762
31	GO:0005172
32	GO:0004962
33	GO:0070483
34	GO:0031012
35	GO:0045359
36	GO:0002282
37	GO:0031077
38	GO:0043117
39	GO:0038025
40	GO:0005010
41	GO:0042660

42	GO:0005072
43	hsa05323
44	GO:0032467
45	GO:0043171
46	GO:0051400
47	GO:0032958
48	GO:0048739
49	GO:0034103
50	GO:0034346
51	GO:0042253
52	GO:0010711
53	GO:0060313
54	GO:0010703
55	GO:2000256
56	GO:0042033
57	GO:0042231
58	GO:0060311
59	GO:0001315
60	GO:0048773
61	GO:0003069
62	GO:0034021
63	GO:0005114
64	GO:0043331
65	GO:0071222
66	GO:0005534
67	GO:0010764
68	GO:0001938
69	GO:0014043
70	GO:1900086
71	GO:0061302
72	GO:0052033
73	GO:0010863
74	GO:0004784
75	GO:0000302
76	GO:0001798
77	GO:0002042
78	GO:0005615
79	GO:0001974
80	GO:0043498
81	GO:0001666
82	GO:0000303
83	GO:0042095
84	GO:0045766

85	GO:0035624
86	GO:0051873
87	GO:0006958
88	GO:0060510
89	GO:0051856
90	GO:0035630
91	GO:0038091
92	GO:0061045
93	GO:0070022
94	GO:0045429
95	GO:0090031
96	hsa05146
97	GO:0030949
98	GO:0060011
99	GO:0032732
100	GO:0048009
101	GO:0016046
102	GO:0070434
103	GO:0070430
104	GO:0002537
105	GO:0007603
106	GO:0032497
107	hsa04145
108	GO:0051142
109	GO:0031109
110	GO:0048245
111	GO:0009597
112	GO:0008228
113	GO:0032722
114	GO:0060312
115	GO:0043154
116	GO:0055090
117	GO:0006957
118	GO:0060319
119	GO:0060754
120	GO:0042116
121	GO:0002693
122	GO:0002457
123	GO:0010477
124	GO:0070301
125	GO:0001910
126	GO:0002576
127	GO:0005581

128	GO:0034185
129	GO:0046911
130	GO:0051651
131	GO:0070373
132	GO:0032805
133	GO:0060346
134	GO:0042060
135	GO:0032855
136	GO:0046716
137	GO:0002551
138	GO:0002384
139	GO:0009441
140	GO:0014896
141	GO:0014904
142	GO:0043559
143	GO:0001574
144	GO:0010001
145	GO:0048598
146	GO:0033627
147	GO:1900006
148	GO:0043206
149	GO:0030214
150	GO:0070091
151	GO:0051894
152	GO:0002021
153	GO:0010716
154	GO:0045103
155	GO:0014826
156	GO:0060394
157	GO:0006029
158	GO:0005586
159	GO:0048010
160	hsa05133
161	GO:0014065
162	GO:0009583
163	GO:0006801
164	GO:0005576
165	GO:0022617
166	GO:0048407
167	GO:0032432
168	GO:0060729
169	GO:0009409
170	GO:0060509

171	GO:0046813
172	GO:0035767
173	GO:0002262
174	GO:0032930
175	GO:0000189
176	GO:0045541
177	GO:0004473
178	GO:0006741
179	GO:0070633
180	GO:0030195
181	GO:0042056
182	GO:0002291
183	GO:0038052
184	GO:0035924
185	GO:0033081
186	GO:0031017
187	GO:0031798
188	GO:0060744
189	GO:0048678
190	GO:0005585
191	GO:0004252
192	GO:0030198
193	GO:0042832
194	GO:0009881
195	GO:0044464
196	GO:0051580
197	GO:0010625
198	GO:0030023
199	GO:0005604
200	GO:0010875
201	GO:0051918
202	GO:0048565
203	GO:0050665
204	GO:0048251
205	GO:0021940
206	GO:0043388
207	GO:0051000
208	GO:0042159
209	GO:0060129
210	GO:0045348
211	GO:0001569
212	GO:0032964
213	GO:0055093

214	GO:0042637
215	GO:0006108
216	GO:0008209
217	GO:0009612
218	GO:0002429
219	GO:0035082
220	GO:0046629
221	GO:0042461
222	GO:0034358
223	GO:0060068
224	GO:0050921
225	GO:0060325
226	GO:0008083
227	GO:0030335
228	GO:0009586
229	GO:0016056
230	GO:0030199
231	GO:0003823
232	GO:0010951
233	GO:0060052
234	GO:0060527
235	GO:0060020
236	GO:0033141
237	GO:0001957
238	GO:0060128
239	GO:0001968
240	GO:0001955
241	GO:0019430
242	GO:0033160
243	GO:0030166
244	GO:0031953
245	GO:0034056
246	GO:0060351
247	GO:0033299
248	GO:0007181
249	GO:0006917
250	GO:0033691
251	GO:0071346
252	GO:0030229
253	GO:0050829
254	GO:2000366
255	GO:0051898
256	GO:0030284

257	GO:0030235
258	GO:0048246
259	GO:0038026
260	GO:0042310
261	GO:0042094
262	GO:0002523
263	GO:0004943
264	GO:0004876
265	GO:0002462
266	GO:0009749
267	GO:0001850
268	GO:0010512
269	GO:0051607
270	GO:0005160
271	GO:0031093
272	GO:0030247
273	GO:0008090
274	GO:0001895
275	GO:0008360
276	GO:0030145
277	GO:0001937
278	GO:0070886
279	GO:0007568
280	GO:0035458
281	GO:0005578
282	GO:0034713
283	GO:0042612
284	GO:0031663
285	GO:0008236
286	GO:0071253
287	GO:0010189
288	GO:0060229
289	GO:0051262
290	GO:0051001
291	GO:0090037
292	GO:0048386
293	GO:0048864
294	GO:0005896
295	GO:0034392
296	GO:0032269
297	GO:0090050
298	GO:0010902
299	GO:0045725

300	GO:0014910
301	GO:0017158
302	GO:0005537
303	GO:0032700
304	GO:0045906
305	GO:0051054
306	GO:0002446
307	GO:0032270
308	GO:0007497
309	GO:0004944
310	GO:0030593
311	GO:0071062
312	GO:0002218
313	GO:0032570
314	hsa05142
315	GO:0032940
316	GO:0070371
317	GO:0046620
318	GO:0072126
319	GO:0006497
320	GO:0072255
321	GO:0016176
322	GO:0072264
323	GO:2000591
324	GO:0019882
325	GO:0003828
326	GO:0001502
327	GO:0070093
328	GO:0006290
329	GO:0032494
330	GO:0007179
331	GO:0033364
332	GO:0051956
333	GO:0033686
334	GO:0071298
335	GO:0009892
336	GO:0060587
337	GO:0005201
338	GO:0030020
339	GO:0009611
340	GO:0030879
341	GO:0050930
342	GO:0033552

343	GO:0002758
344	hsa05143
345	GO:0042583
346	GO:0002687
347	GO:0016619
348	GO:0043200
349	GO:0014834
350	GO:0010763
351	GO:0009314
352	GO:0045188
353	GO:0050777
354	GO:0051280
355	GO:0042698
356	GO:0060591
357	GO:0035066
358	GO:0014909
359	GO:0051897
360	GO:0034505
361	GO:0034188
362	GO:0038027
363	GO:0060228
364	GO:0032089
365	GO:0002790
366	GO:0005518
367	GO:0005158
368	GO:0050679
369	GO:0071356
370	GO:0045084
371	GO:0060047
372	GO:0005161
373	GO:0007183
374	GO:0045630
375	GO:0032609
376	GO:0014823
377	GO:0002092
378	GO:2000288
379	GO:0060741
380	GO:0032878
381	GO:0060740
382	GO:0007569
383	GO:0003032
384	GO:0010936
385	GO:0009408

386	GO:0070051
387	GO:0002430
388	GO:0008191
389	GO:2000098
390	GO:0006707
391	GO:0001540
392	GO:0009311
393	GO:0035491
394	GO:0006508
395	GO:0043017
396	GO:0015920
397	GO:0070887
398	GO:0061044
399	GO:0005548
400	GO:0060230
401	GO:0071258
402	GO:0060426
403	GO:0042307
404	GO:0060644
405	GO:0010595
406	GO:0001541
407	hsa05144
408	GO:0042622
409	GO:0071347
410	GO:0014911
411	GO:0004175
412	GO:0050650
413	GO:0060763
414	GO:0010757
415	GO:0005006
416	GO:0048661
417	GO:0071456
418	GO:0005178
419	GO:0042462
420	GO:0050927
421	GO:0045740
422	GO:0051098
423	GO:0090201
424	GO:0072332
425	GO:0060745
426	GO:0006106
427	GO:0009615
428	GO:0055087

429	GO:0035747
430	GO:0004333
431	GO:0010519
432	GO:0004064
433	GO:0045076
434	GO:0045916
435	GO:0001867
436	GO:0060697
437	GO:0072672
438	GO:0030574
439	GO:0001848
440	GO:0045239
441	GO:0019049
442	GO:0051926
443	GO:0042325
444	GO:0048593
445	GO:0031954
446	GO:0030104
447	GO:0032943
448	GO:0045109
449	GO:0060087
450	GO:0051881
451	GO:0002666
452	GO:0043410
453	GO:0005539
454	GO:0060463
455	GO:0010628
456	GO:0005905
457	GO:0007256
458	GO:2000504
459	GO:0035473
460	GO:0035478
461	GO:0017038
462	GO:0071503
463	GO:0090321
464	GO:2001027
465	GO:0005610
466	GO:0070287
467	GO:0032496
468	GO:0043499
469	GO:0002520
470	GO:0002480
471	GO:0032816

472	GO:0051795
473	hsa05164
474	GO:0030512
475	GO:0017015
476	GO:0016942
477	GO:0051384
478	GO:0042535
479	GO:0050840
480	GO:0007565
481	GO:0043066
482	GO:0051591
483	GO:0051346
484	GO:0010873
485	GO:0048074
486	hsa04620
487	GO:0002532
488	GO:0005130
489	GO:0006491
490	GO:0097193
491	GO:0032510
492	GO:0030704
493	GO:0060220
494	GO:0010751
495	GO:2000048
496	GO:0001525
497	GO:0040007
498	hsa04512
499	GO:0045494
500	GO:0010754

b) mRMR features list

Rank	Feature name
1	GO:0030449
2	GO:2001028
3	GO:0051873
4	hsa04610
5	GO:0043537
6	GO:0071482
7	GO:0055090
8	GO:0006956
9	GO:0072563
10	GO:0048739
11	GO:0005581

12	GO:0004866
13	GO:0060342
14	GO:0001574
15	GO:0034437
16	GO:0002429
17	GO:0031012
18	GO:0005114
19	hsa05150
20	GO:0010466
21	GO:0061302
22	GO:0006029
23	GO:0035624
24	GO:0043536
25	GO:0004473
26	GO:0034436
27	GO:0046629
28	GO:0007603
29	GO:0043171
30	GO:0048844
31	GO:0006741
32	GO:0003273
33	GO:0005585
34	GO:0008228
35	GO:0031077
36	GO:0030023
37	GO:0001970
38	GO:0060744
39	GO:0032757
40	GO:0005604
41	GO:0006958
42	GO:0071953
43	GO:0060229
44	GO:0035082
45	GO:0005010
46	GO:0005586
47	GO:0002575
48	GO:0042612
49	GO:0034447
50	GO:0008236
51	GO:0042461
52	GO:0048407
53	GO:0016495
54	GO:0005610

55	hsa04145
56	GO:0001315
57	GO:0006108
58	GO:0006957
59	GO:0060346
60	GO:0034189
61	GO:0019882
62	GO:0048845
63	GO:0003823
64	GO:0043183
65	GO:0010189
66	GO:0060011
67	GO:0042637
68	GO:0070633
69	GO:0002042
70	GO:0032964
71	GO:0051389
72	GO:0005537
73	GO:0003828
74	GO:0072686
75	GO:0043331
76	GO:0034358
77	GO:0009749
78	GO:0043117
79	GO:0016619
80	GO:0035645
81	GO:0030020
82	GO:0004784
83	hsa05146
84	GO:0031362
85	GO:0005172
86	GO:0051262
87	GO:0001974
88	GO:0001798
89	GO:0070052
90	GO:0001867
91	GO:0001300
92	GO:0030199
93	GO:0048773
94	GO:0021762
95	GO:0004252
96	GO:0035473
97	GO:0042088

98	GO:0038063
99	GO:0038025
100	GO:0055087
101	GO:0038091
102	GO:0032089
103	GO:0045494
104	GO:0035478
105	GO:0071253
106	GO:0030145
107	GO:0007181
108	GO:0005534
109	GO:0033691
110	GO:0051635
111	GO:0045359
112	GO:0005201
113	GO:0017038
114	GO:1900086
115	GO:0009583
116	GO:0004943
117	GO:0032467
118	GO:0030497
119	GO:0003069
120	GO:0071503
121	GO:0070483
122	GO:0060754
123	GO:0010763
124	hsa05323
125	GO:0090321
126	GO:0008191
127	GO:0051894
128	GO:0004470
129	GO:0060394
130	GO:0004962
131	GO:0072384
132	GO:0000302
133	GO:0004876
134	GO:0034605
135	GO:0030247
136	GO:0070287
137	GO:0051918
138	GO:0002282
139	GO:0006497
140	GO:0005160

141	GO:0032393
142	GO:0032958
143	GO:0005608
144	GO:0034021
145	GO:0009311
146	GO:0035767
147	GO:0043498
148	GO:0002462
149	GO:0010902
150	GO:0048565
151	GO:0031701
152	GO:0034185
153	GO:0033299
154	GO:0046548
155	GO:0051400
156	GO:0070051
157	GO:0045076
158	GO:0030214
159	GO:0034103
160	GO:0005592
161	GO:0042490
162	GO:0060510
163	GO:0001850
164	GO:0032793
165	GO:0005072
166	GO:0035455
167	GO:0009586
168	GO:0035845
169	GO:0014909
170	GO:0002021
171	GO:0048598
172	GO:0010711
173	GO:0021678
174	GO:0005540
175	GO:0012507
176	GO:0060319
177	GO:0010951
178	GO:0051683
179	GO:0034346
180	GO:0007179
181	GO:0005597
182	GO:0060313
183	GO:0010923

184	GO:0060052
185	GO:0050921
186	GO:0043206
187	GO:0002480
188	GO:0046716
189	GO:0003085
190	hsa05133
191	GO:0000303
192	GO:0001502
193	GO:0010764
194	GO:0010983
195	GO:0002666
196	GO:0016056
197	hsa04512
198	GO:1900006
199	GO:0060068
200	GO:0042462
201	GO:0034713
202	GO:0004509
203	GO:0010001
204	GO:0032497
205	GO:0097193
206	GO:0010703
207	GO:0015074
208	GO:0002092
209	GO:0010751
210	GO:0042310
211	GO:0033627
212	GO:0001957
213	GO:0006705
214	GO:0006801
215	GO:0055106
216	GO:0006994
217	GO:0060311
218	GO:0001666
219	GO:0006491
220	GO:0019287
221	GO:0010165
222	GO:0005539
223	GO:0048010
224	GO:0009881
225	GO:0010519
226	GO:0030903

227	GO:0051856
228	GO:0006351
229	GO:0051272
230	GO:0035630
231	GO:0019166
232	GO:0006106
233	GO:0022617
234	GO:0010501
235	GO:0060047
236	GO:0042660
237	GO:0060351
238	GO:0050501
239	GO:0052033
240	GO:0010754
241	GO:0045953
242	GO:0090031
243	GO:0030621
244	GO:0010875
245	GO:0008347
246	GO:0004333
247	GO:0061045
248	GO:0001578
249	GO:0051142
250	GO:0007184
251	GO:0090037
252	GO:0072332
253	GO:0043405
254	GO:0035747
255	GO:0045239
256	GO:0032940
257	GO:0005548
258	GO:0048245
259	GO:0048549
260	GO:0046314
261	GO:0048593
262	GO:0071222
263	GO:0019799
264	GO:0010863
265	GO:2001199
266	GO:0032996
267	GO:0030574
268	GO:0038026
269	GO:2000504

270	GO:0035924
271	GO:0042583
272	GO:0072672
273	GO:0042253
274	GO:0038062
275	GO:0006168
276	GO:0002576
277	GO:0002581
278	GO:0048009
279	GO:2001027
280	GO:0016817
281	GO:0042307
282	GO:0031109
283	GO:0045423
284	GO:0002430
285	GO:0060228
286	GO:0014826
287	GO:0030949
288	GO:0019730
289	GO:0060325
290	GO:0002504
291	GO:0005158
292	GO:0061338
293	GO:0031491
294	GO:2000256
295	GO:0006353
296	GO:0045198
297	GO:0017158
298	GO:0042116
299	GO:0051271
300	GO:0005578
301	GO:0009597
302	GO:0045906
303	GO:0034122
304	GO:0005582
305	GO:0043152
306	GO:0002605
307	GO:0004064
308	GO:0042033
309	GO:0001968
310	GO:0045414
311	GO:0014043
312	GO:0050968

313	GO:0035066
314	GO:0035375
315	GO:0043559
316	GO:0050665
317	GO:0008970
318	GO:0005588
319	GO:0042231
320	GO:0060765
321	GO:0001763
322	GO:0003948
323	GO:0010760
324	GO:0043256
325	GO:0008474
326	GO:0050746
327	GO:0045916
328	GO:0014065
329	GO:0045209
330	GO:0033364
331	GO:0050928
332	GO:0033145
333	GO:0060697
334	GO:0060174
335	GO:0019064
336	GO:0004181
337	GO:0004944
338	GO:0007183
339	GO:0031954
340	GO:0070022
341	GO:0019962
342	GO:0034188
343	GO:0002551
344	GO:0035270
345	GO:0045204
346	GO:0016939
347	GO:0043654
348	GO:0071062
349	GO:0034056
350	GO:0047915
351	GO:0050777
352	GO:0031714
353	GO:0001937
354	GO:0060042
355	GO:0050689

356	GO:0005583
357	GO:0034370
358	GO:0001938
359	GO:0004906
360	GO:0038027
361	GO:0001848
362	GO:0032364
363	GO:0045964
364	GO:0070093
365	hsa04974
366	GO:0047485
367	GO:0031953
368	GO:0008209
369	GO:0008241
370	GO:0008109
371	GO:0051098
372	GO:0042056
373	GO:0005813
374	GO:0046813
375	GO:0009258
376	GO:0070373
377	GO:0000003
378	GO:0050655
379	GO:0032510
380	GO:0010512
381	GO:0002790
382	GO:0016018
383	GO:0002262
384	GO:0032374
385	GO:0002687
386	GO:0042060
387	GO:0050819
388	GO:0070584
389	GO:0032930
390	GO:0048074
391	GO:0032432
392	GO:0010873
393	GO:0001552
394	GO:0005905
395	GO:0008373
396	GO:0043184
397	GO:0010936
398	GO:0002767

399	GO:0032855
400	GO:0036117
401	GO:0002693
402	GO:0006869
403	GO:0030704
404	GO:0010070
405	GO:0045263
406	GO:0030229
407	GO:0030284
408	GO:0051101
409	GO:0006290
410	GO:0050930
411	GO:0015722
412	GO:0003417
413	GO:0060123
414	GO:0048147
415	GO:0030512
416	GO:0003032
417	GO:0002523
418	GO:0005930
419	GO:0032732
420	GO:0060220
421	GO:0005607
422	GO:0018298
423	GO:0014910
424	GO:0005615
425	GO:0004496
426	GO:0043570
427	GO:0002457
428	GO:0048306
429	GO:0010740
430	GO:0043388
431	GO:0090207
432	GO:0031093
433	GO:0008817
434	GO:0032722
435	GO:0034505
436	GO:0032691
437	GO:0051054
438	GO:0051956
439	GO:0032633
440	GO:0001895
441	GO:0048251

442	GO:0007387
443	GO:0043499
444	GO:0042622
445	GO:0060129
446	GO:0002820
447	GO:0001865
448	GO:0048386
449	GO:0030198
450	GO:0055085
451	GO:0032816
452	GO:0008119
453	GO:0010477
454	GO:0008395
455	GO:0004175
456	GO:0031573
457	GO:0033686
458	GO:0050840
459	GO:0051898
460	GO:0042534
461	GO:0007388
462	GO:0045103
463	GO:0030108
464	GO:0016046
465	GO:0004525
466	GO:0030950
467	GO:0032269
468	GO:0001750
469	GO:0033081
470	GO:0090050
471	GO:0060448
472	GO:0071298
473	GO:0010917
474	GO:0055093
475	GO:0034369
476	GO:0017015
477	GO:0048664
478	GO:0004903
479	GO:0001910
480	GO:0002764
481	GO:0009409
482	GO:0072126
483	GO:0032368
484	GO:0043154

485	GO:0010813
486	GO:0035458
487	GO:0070195
488	hsa01040
489	GO:0070434
490	GO:0060707
491	GO:0001844
492	GO:0005584
493	GO:0010900
494	GO:0030195
495	GO:0042405
496	GO:0042832
497	GO:0003334
498	GO:0030879
499	GO:0001783
500	GO:0019865

(4) Dataset D_4

a) MaxRel features list

Rank	Feature name
1	GO:0001970
2	GO:0030449
3	GO:0034436
4	GO:0051400
5	GO:0034437
6	GO:0001798
7	GO:0034189
8	GO:0038025
9	GO:0016495
10	GO:0006956
11	hsa05150
12	GO:0072563
13	GO:0071953
14	hsa04610
15	GO:1900006
16	GO:0003273
17	GO:0035645
18	GO:0006957
19	GO:0010001
20	GO:0042088
21	GO:0071482
22	GO:0051389
23	GO:0038026

24	GO:0001938
25	GO:0003069
26	GO:0048773
27	GO:0034021
28	GO:0001315
29	GO:0048245
30	GO:0031109
31	GO:0004866
32	GO:0045103
33	GO:0002504
34	GO:0001850
35	GO:0002462
36	GO:0004943
37	GO:0004876
38	GO:0021762
39	GO:0034447
40	GO:0014043
41	GO:0010863
42	GO:0043537
43	GO:0043183
44	GO:0002042
45	GO:0060311
46	GO:0034103
47	GO:0060313
48	GO:0010703
49	GO:0010711
50	GO:0032805
51	GO:0051651
52	GO:0046911
53	GO:0008228
54	GO:0090031
55	GO:0016046
56	GO:0032467
57	GO:0042832
58	GO:0070430
59	GO:0070434
60	GO:0032732
61	GO:0043171
62	GO:0005581
63	GO:0004944
64	GO:0043536
65	GO:0042660
66	GO:0048844

67	GO:0001300
68	GO:0045429
69	GO:2001028
70	GO:0051580
71	GO:0032463
72	GO:0044464
73	GO:0034185
74	GO:0019882
75	GO:0010625
76	GO:0050665
77	hsa05133
78	GO:0045766
79	GO:0042613
80	GO:0031012
81	GO:0043206
82	GO:0032958
83	GO:0005172
84	GO:0060342
85	GO:0002537
86	GO:0004962
87	GO:0032432
88	GO:0032809
89	GO:0060394
90	GO:0005072
91	GO:0031714
92	GO:0010760
93	GO:0035924
94	GO:0030949
95	GO:0004784
96	GO:0032700
97	GO:0033627
98	GO:0051918
99	GO:0005586
100	GO:0051856
101	GO:0032930
102	GO:0046813
103	GO:0043498
104	GO:0030195
105	GO:0070483
106	GO:0071346
107	GO:0034346
108	GO:0048407
109	GO:0000189

110	GO:1900086
111	GO:0006940
112	GO:0019430
113	GO:0060052
114	GO:0010759
115	GO:0006958
116	GO:0035630
117	GO:0043154
118	GO:0007497
119	GO:0002693
120	GO:0001910
121	GO:0002457
122	GO:0060129
123	GO:0010477
124	GO:0002575
125	GO:0002384
126	GO:0001974
127	GO:0001968
128	GO:0000303
129	GO:0007603
130	GO:0042060
131	GO:0060312
132	GO:0048845
133	hsa05323
134	GO:0002523
135	GO:0006029
136	GO:0002551
137	GO:0052033
138	GO:0002282
139	GO:0006801
140	GO:0005615
141	GO:0051000
142	GO:0060754
143	GO:0060319
144	GO:0070091
145	GO:0032964
146	GO:0043200
147	GO:0032269
148	GO:0031017
149	GO:0010764
150	GO:0005201
151	GO:0032855
152	GO:0002021

153	GO:0005534
154	GO:0008360
155	GO:0030247
156	GO:0060128
157	GO:0043331
158	GO:0071361
159	GO:0048678
160	GO:0021517
161	GO:0043117
162	GO:0030229
163	GO:0042583
164	GO:0042253
165	GO:2000256
166	GO:0042033
167	GO:0009597
168	GO:0042231
169	GO:0048010
170	GO:0005114
171	GO:0042095
172	GO:0070301
173	GO:0022614
174	GO:0045541
175	GO:0014904
176	GO:0009441
177	GO:0042116
178	GO:0014896
179	GO:0000302
180	GO:0061045
181	GO:0032757
182	GO:0051142
183	GO:0055093
184	GO:0032387
185	GO:0071222
186	GO:0042056
187	GO:0003032
188	GO:0007568
189	GO:0060763
190	GO:0032497
191	GO:0005161
192	GO:0001666
193	GO:0070670
194	GO:0019836
195	GO:0002429

196	GO:0046629
197	GO:0002262
198	GO:0005010
199	GO:0048598
200	GO:0050777
201	GO:0071230
202	GO:0048246
203	GO:0010875
204	GO:0005178
205	GO:0030335
206	GO:0033364
207	GO:0030214
208	GO:0009409
209	GO:0017158
210	GO:0051898
211	GO:0051971
212	GO:0033141
213	GO:0048009
214	hsa05020
215	GO:0009408
216	GO:0050921
217	GO:0001569
218	GO:0007181
219	GO:0032393
220	GO:0005520
221	GO:0009881
222	GO:0030828
223	GO:0032364
224	GO:0060509
225	GO:0060510
226	GO:0060020
227	GO:0060011
228	GO:0045494
229	GO:0032874
230	GO:0030838
231	GO:0034505
232	GO:0032722
233	GO:0050840
234	GO:0002576
235	GO:0006935
236	GO:0030516
237	GO:0001957
238	GO:0051607

239	GO:0031663
240	GO:0061302
241	GO:0005548
242	GO:0045179
243	GO:0050718
244	GO:0050766
245	GO:0070383
246	GO:0071253
247	GO:0051881
248	GO:0060351
249	GO:0051262
250	GO:0070022
251	GO:0038091
252	GO:0004301
253	GO:2001199
254	GO:0032510
255	GO:0046696
256	GO:0014910
257	GO:0033552
258	hsa05143
259	GO:0051894
260	GO:0001937
261	GO:0001955
262	GO:0030023
263	GO:0071062
264	GO:0071347
265	GO:0070541
266	GO:0090197
267	GO:0016004
268	GO:0006700
269	GO:0070373
270	GO:0060729
271	GO:0001917
272	GO:0060426
273	GO:0045188
274	GO:0016209
275	GO:0031093
276	GO:0007250
277	GO:0030198
278	GO:0001869
279	GO:0002381
280	GO:0005579
281	GO:0035082

282	GO:0070206
283	GO:0004252
284	GO:0014909
285	GO:0005576
286	GO:0035458
287	GO:2000502
288	GO:0035684
289	hsa05310
290	GO:0032417
291	hsa04620
292	GO:0008209
293	GO:0042493
294	GO:0032609
295	GO:0030155
296	GO:0002758
297	GO:0010466
298	GO:0007256
299	GO:2000340
300	GO:0035665
301	GO:0035662
302	GO:0006741
303	GO:0010900
304	GO:0034369
305	GO:0035663
306	GO:0032738
307	GO:0032368
308	GO:0004509
309	GO:2000121
310	GO:0060346
311	GO:0046620
312	GO:0031953
313	GO:0002446
314	GO:0021940
315	GO:0042094
316	hsa05142
317	hsa05146
318	GO:0008217
319	GO:0042311
320	GO:0048074
321	GO:0005130
322	GO:0060220
323	GO:0030704
324	GO:0008009

325	GO:0032089
326	GO:0046330
327	GO:0090265
328	GO:0070093
329	GO:0030199
330	GO:2000427
331	hsa05140
332	GO:0031727
333	GO:0002548
334	GO:0045359
335	GO:0043491
336	GO:0050927
337	GO:0051054
338	GO:0042542
339	GO:0030593
340	GO:0033081
341	GO:0060527
342	GO:0072332
343	GO:0032270
344	GO:0002291
345	GO:0001890
346	GO:0030279
347	GO:0010572
348	GO:0046716
349	GO:0014834
350	GO:0010595
351	GO:0001776
352	GO:0006290
353	GO:0051873
354	GO:0008201
355	GO:0035624
356	GO:0004950
357	GO:0022617
358	GO:0019835
359	GO:0002430
360	hsa05164
361	GO:0043017
362	GO:0050871
363	GO:0035988
364	GO:0002218
365	GO:0009611
366	GO:0051602
367	hsa05144

368	GO:0050918
369	GO:0048635
370	GO:0071356
371	GO:0070326
372	GO:0006917
373	GO:0048661
374	GO:0070371
375	GO:0045906
376	GO:0090022
377	GO:0002767
378	GO:0001867
379	GO:0019730
380	GO:0002352
381	GO:0004064
382	GO:0019865
383	GO:0034358
384	GO:0032996
385	GO:0048597
386	GO:0042159
387	GO:2000366
388	GO:0051272
389	GO:0043184
390	GO:0048739
391	GO:0034392
392	GO:0001895
393	GO:0022009
394	GO:0008089
395	GO:0005584
396	GO:0006874
397	GO:0006749
398	GO:0005158
399	GO:0009887
400	GO:0032494
401	GO:0005585
402	GO:0051788
403	GO:0002020
404	GO:0070287
405	GO:0070524
406	GO:0035478
407	GO:0090321
408	GO:0071503
409	GO:0060084
410	GO:0035473

411	GO:0017038
412	GO:0043330
413	GO:0014826
414	GO:0045725
415	GO:0032816
416	GO:0007584
417	GO:0042511
418	GO:0008083
419	GO:0042535
420	GO:0043066
421	GO:0050966
422	GO:0005539
423	GO:0019439
424	GO:0060545
425	GO:0070424
426	GO:0070926
427	GO:0008061
428	GO:0034405
429	GO:0031077
430	GO:0015920
431	GO:0002544
432	GO:2000048
433	GO:0033299
434	GO:0008395
435	GO:0071638
436	GO:0032374
437	GO:0060137
438	GO:0034394
439	GO:0050867
440	GO:0045084
441	GO:0010574
442	GO:0019049
443	GO:0010763
444	GO:0051280
445	GO:0010888
446	GO:0030166
447	GO:0051044
448	GO:0060740
449	GO:0008090
450	GO:0045630
451	GO:0032943
452	GO:0032701
453	GO:2000587

454	GO:0035419
455	GO:0002710
456	GO:0071608
457	GO:0070633
458	GO:0032370
459	GO:0038063
460	GO:0002367
461	GO:0032498
462	GO:2000363
463	GO:0005146
464	GO:0032500
465	GO:0046645
466	GO:0018298
467	GO:0043410
468	GO:0031702
469	GO:0007179
470	GO:0010544
471	GO:0007606
472	GO:0005125
473	GO:0009583
474	GO:0006979
475	GO:0030336
476	GO:0048018
477	GO:0004859
478	GO:0009612
479	GO:0016056
480	GO:0060325
481	GO:0051659
482	GO:0051787
483	GO:0030235
484	GO:0032707
485	GO:0045079
486	GO:0005588
487	GO:0048520
488	GO:0001923
489	GO:0034701
490	GO:0061338
491	GO:0006562
492	GO:0006710
493	GO:0072606
494	GO:0060587
495	GO:0009892
496	GO:0033686

497	GO:0051956
498	GO:0034612
499	GO:0060452
500	GO:0071260

b) mRMR features list

Rank	Feature name
1	GO:0001970
2	GO:0006029
3	GO:0006956
4	GO:0051400
5	GO:0002504
6	GO:0034189
7	GO:0005581
8	GO:0030449
9	GO:0019882
10	GO:2001028
11	GO:0001850
12	GO:0034436
13	GO:0002429
14	GO:0071482
15	hsa04610
16	GO:0072563
17	GO:0032463
18	GO:0042613
19	GO:0001798
20	GO:1900006
21	GO:0008228
22	GO:0031012
23	GO:0046629
24	GO:0016495
25	GO:0002462
26	GO:0034437
27	GO:0006957
28	GO:0048407
29	hsa05150
30	GO:0002042
31	GO:0060394
32	GO:0004943
33	GO:0005201
34	GO:0071953
35	GO:0004866
36	GO:0007603

37	GO:0038025
38	GO:0004876
39	GO:0038063
40	GO:0004301
41	GO:0030247
42	GO:0090031
43	GO:0032393
44	GO:0005586
45	GO:0010001
46	GO:0070524
47	GO:0035645
48	GO:0031714
49	GO:0042088
50	GO:0035082
51	GO:0005114
52	GO:0003069
53	GO:0010760
54	GO:0048739
55	GO:0043171
56	GO:0060052
57	GO:0042583
58	GO:0045494
59	GO:0003273
60	GO:0008395
61	GO:0045179
62	GO:0032089
63	GO:0051389
64	GO:2001199
65	GO:0043537
66	GO:0005588
67	GO:0045103
68	GO:0070287
69	GO:0031109
70	GO:0004509
71	GO:0043183
72	GO:0004944
73	GO:0038026
74	GO:0070206
75	GO:0006741
76	GO:0048773
77	GO:0014909
78	GO:0010863
79	GO:0032964

80	GO:0035478
81	GO:0006958
82	GO:0030214
83	GO:0048245
84	GO:0002381
85	GO:0070584
86	GO:0090321
87	GO:0014043
88	GO:0001867
89	GO:0051918
90	GO:0034021
91	GO:0070424
92	GO:0032510
93	GO:0035924
94	GO:0031616
95	GO:0051873
96	GO:0010759
97	GO:0030023
98	GO:0071253
99	GO:0060311
100	GO:0071503
101	GO:0001968
102	GO:0048074
103	GO:0051262
104	GO:0034185
105	GO:0072686
106	GO:0001315
107	GO:0005585
108	GO:0060011
109	hsa05133
110	GO:0035473
111	GO:0060342
112	GO:0002767
113	GO:0007181
114	GO:0034103
115	GO:0060754
116	GO:0042612
117	GO:0034447
118	GO:0051010
119	GO:0032269
120	GO:0017038
121	GO:0035624
122	GO:0033627

123	GO:0005172
124	GO:0032467
125	GO:0010900
126	GO:0060220
127	GO:0042832
128	GO:0006940
129	GO:0050777
130	GO:0034369
131	GO:0043206
132	GO:0042461
133	GO:0050766
134	GO:0050665
135	GO:0030279
136	GO:0032417
137	GO:0060313
138	GO:0005161
139	GO:0010991
140	GO:0019430
141	GO:0070383
142	GO:0005597
143	GO:0030704
144	GO:0032368
145	GO:0048844
146	GO:0010703
147	GO:0019825
148	GO:0060744
149	GO:0010875
150	GO:1900086
151	GO:0004784
152	GO:0021678
153	GO:0004962
154	GO:0071230
155	GO:0032701
156	GO:0046703
157	GO:0002523
158	GO:0010711
159	GO:0019439
160	GO:0001917
161	GO:0005010
162	GO:0019730
163	GO:0001938
164	GO:0033961
165	GO:0047617

166	GO:0050840
167	GO:0006801
168	GO:0046813
169	GO:0004473
170	GO:0002262
171	GO:0004843
172	GO:0051580
173	GO:0035419
174	GO:0043536
175	GO:0005537
176	GO:0016046
177	GO:0061302
178	GO:0032996
179	GO:0009249
180	GO:0060128
181	GO:0000932
182	GO:0051272
183	GO:0009881
184	GO:0060084
185	GO:0044464
186	GO:0055087
187	GO:0002710
188	GO:0070430
189	GO:0035845
190	GO:0006930
191	GO:0034358
192	GO:0007497
193	GO:0035988
194	GO:0002666
195	GO:0055092
196	GO:0032432
197	GO:0050921
198	GO:0071361
199	GO:0030020
200	GO:0004030
201	GO:0010625
202	GO:0038062
203	GO:0002021
204	GO:0031362
205	GO:0002575
206	GO:0034365
207	GO:0070434
208	GO:0071608

209	GO:0033364
210	GO:0005488
211	GO:0060129
212	GO:0016409
213	GO:0005072
214	GO:0018243
215	GO:0005579
216	GO:0030199
217	GO:0032809
218	GO:0010949
219	GO:0002352
220	GO:0005930
221	GO:0019835
222	GO:0005548
223	GO:0032732
224	GO:0030145
225	GO:0003032
226	GO:0002367
227	GO:0045116
228	GO:0051856
229	GO:0007183
230	GO:0000254
231	GO:0060351
232	GO:0006562
233	GO:0030949
234	GO:0016209
235	hsa04974
236	GO:0016620
237	GO:0072332
238	GO:0090281
239	GO:0021762
240	GO:0006994
241	GO:0032498
242	GO:0048018
243	GO:0032805
244	GO:0033299
245	GO:0072606
246	GO:0018242
247	GO:0061338
248	GO:0048597
249	GO:0007184
250	GO:0001300
251	GO:0034445

252	GO:0002430
253	GO:0046599
254	GO:0032364
255	GO:0001974
256	GO:0001578
257	GO:0071638
258	GO:0000506
259	GO:0032930
260	GO:0045796
261	GO:0043117
262	GO:2000363
263	GO:0051651
264	GO:0001750
265	GO:0004085
266	GO:0048845
267	GO:0004221
268	GO:0060123
269	GO:0015074
270	GO:0071346
271	GO:0017176
272	GO:0071062
273	GO:0032500
274	GO:0006108
275	GO:0034505
276	GO:0043331
277	GO:0046911
278	GO:0005462
279	GO:0043531
280	GO:0048598
281	GO:2000340
282	GO:0046359
283	GO:0000303
284	GO:0033145
285	GO:0010763
286	GO:0060319
287	GO:0046645
288	GO:0035665
289	GO:0048549
290	GO:0031953
291	GO:0034346
292	GO:0048306
293	GO:0015788
294	GO:0006705

295	GO:0021517
296	GO:0035270
297	GO:0035662
298	GO:0006700
299	GO:0005158
300	GO:0005338
301	GO:0008209
302	GO:0005146
303	GO:0005520
304	GO:0035663
305	GO:0002315
306	GO:0022614
307	GO:0070052
308	GO:0050718
309	GO:0042311
310	GO:0032387
311	GO:0000910
312	GO:0032958
313	GO:0072488
314	GO:0032855
315	GO:0003823
316	GO:0045204
317	GO:0008194
318	GO:0032738
319	GO:0055070
320	GO:0007606
321	GO:0038091
322	GO:0002551
323	GO:0015780
324	GO:0004252
325	GO:0006710
326	GO:0004906
327	GO:0035630
328	GO:0048025
329	GO:0006749
330	GO:0045209
331	GO:0009893
332	GO:0030195
333	GO:0044242
334	GO:0032700
335	GO:0061299
336	GO:0030198
337	GO:0010466

338	GO:0090022
339	GO:0008519
340	GO:0070483
341	GO:0010821
342	GO:0019049
343	GO:0002693
344	GO:0001574
345	GO:0034605
346	GO:0030229
347	GO:0042267
348	GO:0048010
349	GO:0015918
350	GO:0016254
351	GO:0004012
352	GO:0042660
353	GO:0006168
354	hsa05020
355	GO:0005534
356	GO:0042116
357	GO:0032383
358	GO:0070633
359	GO:0030382
360	GO:0051788
361	GO:0016619
362	GO:0000302
363	GO:0001512
364	GO:0051098
365	GO:0002537
366	GO:0005883
367	GO:0002446
368	GO:0045263
369	GO:0004064
370	GO:0033165
371	GO:0008360
372	GO:0043394
373	GO:0051881
374	GO:0048806
375	GO:0044297
376	GO:0051894
377	GO:0045309
378	GO:0001910
379	GO:0006290
380	GO:0042447

381	GO:0005539
382	GO:0004128
383	GO:0008753
384	GO:0030828
385	GO:0070278
386	GO:0009597
387	GO:0006704
388	GO:0043024
389	GO:0030838
390	GO:0071352
391	GO:0002455
392	GO:0031902
393	GO:0032691
394	GO:0055093
395	GO:0045869
396	GO:0070722
397	GO:0002457
398	GO:0005540
399	GO:0019836
400	GO:0046548
401	GO:0043154
402	GO:0090207
403	GO:0071622
404	GO:0001957
405	GO:0070670
406	GO:0007250
407	GO:0021541
408	GO:0001869
409	GO:0010826
410	GO:0045591
411	GO:0009595
412	GO:0042056
413	GO:0085029
414	GO:0010260
415	GO:0014826
416	GO:0045429
417	GO:0045541
418	GO:0031491
419	GO:0010942
420	GO:0043184
421	GO:0051054
422	GO:0045414
423	GO:2000587

424	GO:0006351
425	GO:0010477
426	GO:0097190
427	GO:0032391
428	GO:0030492
429	GO:0010936
430	GO:0060042
431	GO:0009624
432	GO:0019865
433	GO:0032497
434	GO:0016667
435	GO:0032370
436	GO:0016829
437	GO:0043200
438	GO:0008347
439	GO:0003828
440	GO:0018298
441	GO:0045423
442	GO:0060510
443	GO:0019706
444	GO:0004181
445	hsa05323
446	GO:0071208
447	GO:2000870
448	GO:0046417
449	GO:0042060
450	GO:0046620
451	GO:0050689
452	GO:0033192
453	GO:0034014
454	GO:0002480
455	GO:0043498
456	GO:0010700
457	GO:0043654
458	GO:0050927
459	GO:0008187
460	GO:0004903
461	GO:0006537
462	GO:0031593
463	GO:0001923
464	GO:0010764
465	GO:0070266
466	GO:0006122

467	GO:0006649
468	GO:0043618
469	GO:0052033
470	GO:0034041
471	GO:0032502
472	GO:0060279
473	GO:0005592
474	GO:0048246
475	GO:0010804
476	GO:0070195
477	GO:0070051
478	GO:0051148
479	GO:0018894
480	GO:0003951
481	GO:0032816
482	GO:0003334
483	GO:0061045
484	GO:2000116
485	GO:0001729
486	GO:0001937
487	GO:0009720
488	GO:0032793
489	GO:0008287
490	GO:0043491
491	GO:0072498
492	GO:0005178
493	GO:0046314
494	GO:0035767
495	GO:0035458
496	GO:0002005
497	GO:0032937
498	GO:0006516
499	GO:0045766
500	GO:0090240

(5) Dataset D_5

a) MaxRel features list

Rank	Feature name
1	GO:0043537
2	GO:0034189
3	GO:0030449
4	hsa05150
5	GO:1900006

6	GO:0001970
7	GO:0071482
8	GO:0034437
9	GO:0034436
10	GO:0072563
11	GO:0038025
12	GO:0006957
13	GO:0071953
14	hsa04610
15	GO:2001028
16	GO:0003273
17	GO:0051400
18	GO:0016495
19	GO:0090031
20	GO:0048844
21	GO:0060342
22	GO:0051389
23	GO:0001968
24	GO:0048739
25	GO:0043536
26	GO:0031012
27	GO:0031109
28	GO:0032930
29	GO:0008228
30	GO:0034447
31	GO:0035645
32	GO:0021762
33	GO:0002042
34	GO:0043183
35	GO:0006956
36	GO:0010703
37	GO:0034103
38	GO:0060313
39	GO:0010711
40	GO:0009597
41	GO:0060311
42	GO:0001798
43	GO:0005615
44	GO:0060394
45	GO:0045766
46	GO:0032467
47	GO:0001938
48	GO:0006958

49	GO:0046911
50	GO:0045429
51	GO:0051651
52	GO:0032805
53	GO:0010001
54	GO:0051898
55	GO:0032958
56	GO:0002575
57	GO:0001569
58	GO:0034346
59	GO:0030229
60	GO:0002282
61	GO:0032855
62	GO:0048845
63	GO:0070483
64	GO:0043498
65	GO:0061302
66	GO:1900086
67	GO:0014043
68	GO:0033627
69	GO:0048246
70	GO:0030023
71	GO:0001300
72	GO:0061045
73	GO:0051856
74	GO:0071222
75	hsa05323
76	GO:0000303
77	GO:0045103
78	GO:0043331
79	GO:0002551
80	GO:0043206
81	GO:0042159
82	GO:0043117
83	GO:0005172
84	GO:0009409
85	GO:0006940
86	GO:0021517
87	GO:0010759
88	GO:0000302
89	GO:0051000
90	GO:0046813
91	GO:0042095

92	GO:0048598
93	GO:0050665
94	GO:0032757
95	GO:0060128
96	GO:0055093
97	GO:0030949
98	GO:0002446
99	GO:0051580
100	GO:0032463
101	GO:0048245
102	GO:0044464
103	GO:0010625
104	GO:0014904
105	GO:0014896
106	GO:0048251
107	GO:0010863
108	GO:0060020
109	GO:0009441
110	GO:0060754
111	GO:0038091
112	GO:0070371
113	GO:0002537
114	GO:0050777
115	GO:0030247
116	GO:0031093
117	GO:0051142
118	GO:0007603
119	GO:0007568
120	GO:0001957
121	GO:0001974
122	GO:0060129
123	GO:0035924
124	GO:0042660
125	GO:0005010
126	GO:0048678
127	GO:0005072
128	GO:0004866
129	GO:0033364
130	GO:0060319
131	GO:0002384
132	GO:0038026
133	GO:0042832
134	GO:0045359

135	GO:0052033
136	GO:0042088
137	GO:0006029
138	GO:0005581
139	GO:0002457
140	GO:0016046
141	GO:0001910
142	GO:0070434
143	GO:0070430
144	GO:0010477
145	GO:0033160
146	GO:0043171
147	GO:0032732
148	GO:0002693
149	GO:0060527
150	GO:0002262
151	GO:0060591
152	GO:0042116
153	GO:0043184
154	GO:0019934
155	GO:0060011
156	GO:0034021
157	GO:0048773
158	GO:0001315
159	GO:0003069
160	GO:0030214
161	GO:0001937
162	GO:0060346
163	GO:0048009
164	GO:0004784
165	GO:0005534
166	GO:0002462
167	GO:0001850
168	GO:0004876
169	GO:0004943
170	GO:0032809
171	GO:0004962
172	GO:2000256
173	GO:0042231
174	GO:0060763
175	GO:0009881
176	GO:0042583
177	GO:0042253

178	GO:0042033
179	GO:0051001
180	GO:0045541
181	GO:0005576
182	GO:0005178
183	GO:0051918
184	GO:0035988
185	GO:0007565
186	GO:0007179
187	GO:0001917
188	GO:0070091
189	GO:0042060
190	GO:0032964
191	GO:0060312
192	GO:0032497
193	GO:0071346
194	GO:0032432
195	GO:0000189
196	GO:0060744
197	GO:0021940
198	GO:0034405
199	GO:0014065
200	GO:0005201
201	GO:0030020
202	GO:0010764
203	GO:0030516
204	GO:0008209
205	GO:0070022
206	GO:0005578
207	GO:0070383
208	GO:0048635
209	GO:0033141
210	GO:0031017
211	GO:0070373
212	GO:0090037
213	GO:0035630
214	GO:0060509
215	GO:0019049
216	GO:0007183
217	GO:0048661
218	GO:0007256
219	GO:0051971
220	GO:0030279

221	GO:0045348
222	GO:0070886
223	GO:0002291
224	GO:0019430
225	GO:0008360
226	GO:0007497
227	GO:2000121
228	GO:0050840
229	GO:0030168
230	GO:0010544
231	GO:0001666
232	GO:0002523
233	GO:0032488
234	GO:0005586
235	GO:0001781
236	GO:0070301
237	GO:0005896
238	GO:0060729
239	GO:0046668
240	GO:0038052
241	GO:0031798
242	GO:0072332
243	GO:0003416
244	GO:0019064
245	GO:0035082
246	GO:0042461
247	GO:0004944
248	GO:0035684
249	GO:0005585
250	GO:0035458
251	GO:2000502
252	GO:0048018
253	GO:0010740
254	GO:0030195
255	GO:0031077
256	GO:0060745
257	GO:0051712
258	GO:0046620
259	GO:0042622
260	GO:0005114
261	GO:0043066
262	GO:0071062
263	GO:0030336

264	GO:0042511
265	GO:0060090
266	GO:0032510
267	GO:0030704
268	GO:0048074
269	GO:0060220
270	hsa04145
271	GO:0045669
272	GO:0034363
273	GO:0017046
274	GO:0030199
275	GO:0030335
276	GO:0002548
277	GO:0050679
278	GO:0008217
279	GO:0048407
280	GO:0005006
281	GO:0019836
282	GO:0051098
283	GO:0070326
284	GO:0033552
285	GO:0002456
286	GO:0032609
287	GO:0090303
288	GO:0032700
289	GO:0060052
290	GO:0032270
291	GO:0043559
292	GO:0046579
293	GO:0060559
294	GO:0051044
295	GO:2000379
296	GO:0009408
297	GO:0034185
298	GO:0002576
299	GO:0030512
300	GO:0008083
301	GO:0008201
302	GO:0005520
303	GO:0030169
304	GO:2000048
305	GO:0045869
306	GO:0010763

307	GO:0051272
308	GO:0045742
309	GO:0003340
310	GO:0043200
311	GO:0051262
312	GO:0034505
313	hsa05144
314	GO:0031701
315	GO:0051146
316	GO:0032722
317	GO:0003417
318	GO:0045445
319	GO:0030828
320	GO:0007263
321	GO:0045630
322	GO:0033690
323	GO:0030139
324	GO:0060550
325	GO:2000309
326	GO:0060552
327	GO:0044146
328	GO:0071351
329	GO:0035845
330	GO:0060557
331	GO:0051280
332	GO:0031953
333	GO:0060741
334	GO:0085029
335	GO:0032269
336	GO:0060662
337	GO:0030235
338	GO:0030104
339	GO:0032878
340	GO:0032943
341	GO:0060644
342	GO:2000427
343	GO:0051607
344	GO:0043410
345	GO:0002062
346	GO:0050829
347	GO:0035473
348	GO:0071503
349	GO:0035478

350	GO:0090321
351	GO:0017038
352	GO:0006935
353	GO:0010595
354	GO:0030198
355	GO:0014826
356	GO:0017015
357	GO:0006707
358	GO:0070052
359	GO:0071560
360	GO:0002430
361	GO:0070051
362	hsa05143
363	GO:0005161
364	GO:0001786
365	GO:0001502
366	GO:0016056
367	GO:0048565
368	GO:0002218
369	hsa05146
370	GO:0070541
371	GO:0042542
372	GO:0060325
373	GO:0060351
374	GO:0048146
375	GO:0030593
376	GO:0007160
377	GO:0070926
378	GO:0060545
379	GO:0004012
380	GO:0007181
381	GO:0022617
382	GO:0048185
383	GO:0005212
384	GO:0051054
385	GO:0071356
386	GO:0070670
387	GO:0042698
388	GO:0002687
389	GO:0042056
390	GO:0007569
391	GO:0042092
392	GO:0022009

393	GO:0045599
394	GO:0002758
395	hsa05219
396	GO:0051152
397	GO:0031102
398	GO:0045740
399	GO:0051926
400	GO:0071391
401	GO:0030346
402	GO:0035767
403	GO:0032966
404	GO:2000288
405	GO:0050921
406	GO:0038063
407	GO:0070633
408	GO:0031362
409	GO:0010628
410	GO:0090190
411	GO:0060174
412	GO:0005138
413	GO:0008285
414	GO:0031727
415	GO:0090265
416	GO:0033081
417	GO:0045188
418	GO:0060561
419	GO:0010332
420	GO:0030308
421	GO:0009583
422	GO:0071456
423	GO:0043277
424	GO:0060510
425	GO:0010189
426	GO:0051216
427	GO:0001822
428	GO:0007566
429	GO:0010718
430	GO:0032417
431	GO:0071260
432	GO:0032784
433	GO:0071223
434	GO:0070208
435	GO:0002520

436	GO:0043491
437	GO:0010574
438	GO:0017158
439	GO:0060751
440	GO:0034393
441	GO:0009817
442	GO:0002460
443	GO:1900126
444	GO:0001955
445	GO:0050927
446	GO:0042993
447	GO:0008089
448	GO:0051894
449	GO:0002021
450	GO:0051246
451	GO:0060740
452	GO:0060087
453	GO:0046716
454	GO:0014834
455	GO:0001895
456	GO:0043526
457	GO:0002767
458	GO:0010760
459	GO:0014061
460	GO:0010897
461	GO:0034358
462	GO:0031714
463	GO:0010519
464	GO:0001848
465	GO:0002581
466	GO:0033299
467	GO:0008009
468	GO:0005125
469	GO:0051919
470	GO:0002605
471	GO:0005604
472	GO:0006801
473	GO:0010754
474	GO:0071674
475	GO:0010751
476	GO:0030177
477	GO:0009612
478	GO:0006928

479	GO:0010694
480	GO:0031663
481	GO:0031702
482	GO:0043654
483	GO:0038027
484	GO:0006165
485	GO:0006183
486	GO:0006228
487	GO:0002790
488	GO:0042612
489	GO:0016525
490	GO:0004550
491	GO:0046548
492	GO:0034188
493	GO:0043325
494	GO:0042462
495	GO:0034392
496	GO:0051591
497	GO:0051659
498	GO:0010165
499	GO:0010812
500	GO:0071230

b) mRMR features list

Rank	Feature name
1	GO:0043537
2	hsa04610
3	GO:0071482
4	GO:0001968
5	GO:0034189
6	GO:0005581
7	GO:0030449
8	GO:0048739
9	hsa05150
10	GO:0030023
11	GO:1900006
12	GO:0061302
13	GO:0006029
14	GO:0001970
15	GO:0060744
16	GO:0034437
17	GO:0006957
18	GO:0072563

19	GO:0008228
20	GO:0030020
21	GO:0032930
22	GO:0006956
23	GO:0031012
24	GO:0002042
25	GO:0071953
26	GO:0006168
27	GO:2001028
28	GO:0007603
29	GO:0035473
30	GO:0032463
31	GO:0034436
32	GO:0006958
33	GO:0060394
34	GO:0035082
35	GO:0035988
36	GO:0030247
37	GO:0031362
38	GO:0051400
39	GO:0010001
40	GO:0032964
41	GO:0042612
42	GO:0051389
43	GO:0006940
44	GO:0042583
45	GO:0002462
46	GO:0060342
47	GO:0002446
48	GO:0042461
49	GO:0038025
50	GO:0071503
51	GO:0010759
52	GO:0048018
53	GO:0050777
54	GO:0035478
55	GO:0004866
56	GO:0090031
57	GO:0038063
58	GO:0070052
59	GO:0072332
60	GO:0043184
61	GO:0031701

62	GO:0035645
63	GO:0001798
64	GO:0005201
65	GO:0090321
66	GO:0009597
67	GO:0003416
68	GO:0001850
69	GO:0070383
70	GO:0034450
71	GO:0016495
72	GO:0045254
73	GO:0048844
74	GO:0001957
75	GO:0035845
76	GO:0017038
77	GO:0060754
78	GO:0030229
79	GO:0030176
80	GO:0003273
81	GO:0004876
82	GO:0010826
83	GO:0007179
84	GO:0033364
85	GO:0031109
86	GO:0017046
87	GO:1900086
88	GO:0019509
89	GO:0043206
90	GO:0004943
91	GO:0030704
92	GO:0021517
93	GO:0010369
94	GO:0005010
95	GO:0051272
96	GO:0002767
97	GO:0001917
98	GO:0000302
99	GO:0005578
100	GO:0004181
101	GO:0055093
102	GO:0008187
103	GO:0005592
104	GO:0034447

105	GO:0043248
106	GO:0061045
107	GO:0051262
108	GO:0009881
109	GO:0051898
110	GO:0048074
111	GO:0006165
112	GO:0019064
113	GO:0002262
114	GO:0043022
115	GO:0000303
116	GO:0002575
117	GO:0046548
118	GO:0032467
119	GO:0005540
120	GO:0004944
121	GO:0043331
122	GO:0051918
123	GO:0005585
124	GO:0051148
125	GO:0060220
126	GO:0001937
127	GO:0032958
128	GO:0003997
129	GO:0035924
130	GO:0060090
131	GO:0032855
132	GO:0006183
133	GO:0030279
134	GO:0038026
135	GO:0060346
136	GO:0005586
137	GO:0004784
138	GO:0060011
139	GO:0071529
140	GO:0046599
141	GO:0048246
142	GO:0043654
143	GO:0010703
144	GO:0010519
145	GO:0048251
146	GO:0032510
147	GO:0043183

148	GO:0032393
149	GO:0006228
150	GO:0007183
151	GO:0002429
152	GO:0000003
153	GO:0034103
154	GO:0070522
155	GO:0045869
156	GO:0043117
157	GO:0060128
158	GO:0007181
159	GO:0006086
160	GO:0060052
161	hsa05323
162	GO:0008340
163	GO:0005930
164	GO:0043536
165	GO:0010189
166	GO:0019799
167	GO:0060313
168	GO:0030199
169	GO:0002551
170	GO:0009451
171	GO:0004550
172	GO:2000764
173	GO:0001569
174	GO:0001919
175	GO:0072686
176	GO:0030169
177	GO:0010711
178	GO:2000763
179	GO:2001199
180	GO:0034405
181	GO:0050665
182	GO:0007184
183	GO:0046629
184	GO:0055087
185	GO:0030949
186	GO:0004012
187	GO:0070483
188	GO:0014909
189	GO:0048476
190	GO:0046359

191	GO:0060311
192	GO:0001974
193	GO:0008266
194	GO:0060662
195	GO:0038091
196	GO:0005602
197	GO:0034711
198	GO:0051856
199	GO:0045494
200	GO:0045103
201	GO:0050840
202	GO:0031616
203	GO:0010987
204	GO:0060123
205	GO:0048598
206	GO:0004509
207	GO:0043498
208	GO:0020028
209	GO:0034021
210	hsa04145
211	GO:0048845
212	GO:0004085
213	GO:0017002
214	GO:0033572
215	GO:0001786
216	GO:0032417
217	GO:0042159
218	GO:0005582
219	GO:0045964
220	GO:0034346
221	GO:0019882
222	GO:0033690
223	GO:0032691
224	GO:0072384
225	GO:0021762
226	GO:0038062
227	GO:0043178
228	GO:0014043
229	GO:0043171
230	GO:0070584
231	GO:0002666
232	GO:0007263
233	GO:0033627

234	GO:0008395
235	GO:0019934
236	GO:0031593
237	GO:0071222
238	GO:0043024
239	GO:0060129
240	GO:0035624
241	GO:0055106
242	GO:0001552
243	GO:0034358
244	GO:0005615
245	GO:0034605
246	GO:0003823
247	GO:0045204
248	GO:0002430
249	GO:0060351
250	GO:0048773
251	hsa00290
252	GO:0004962
253	GO:0060427
254	GO:0010760
255	GO:0001938
256	GO:0016500
257	GO:0071777
258	GO:0051271
259	GO:0070371
260	GO:0045209
261	GO:0048640
262	GO:0009409
263	GO:0071638
264	GO:0060319
265	GO:0043159
266	GO:0031093
267	GO:0060658
268	GO:0005212
269	GO:0001867
270	GO:0043405
271	GO:0042157
272	GO:0048245
273	GO:0048593
274	GO:0060591
275	GO:0038027
276	GO:0031714

277	GO:0030184
278	GO:0001315
279	GO:0050953
280	GO:0004169
281	GO:0016578
282	GO:0033299
283	GO:0022617
284	GO:0060174
285	GO:0008554
286	GO:0002282
287	GO:0051988
288	GO:0071241
289	GO:0007565
290	GO:0015074
291	GO:0042116
292	GO:0015126
293	GO:0090101
294	GO:0005172
295	GO:0060699
296	GO:0005583
297	GO:0042613
298	GO:0034363
299	GO:0003417
300	GO:0014904
301	GO:0042978
302	GO:0007062
303	GO:0007497
304	GO:0003069
305	GO:0016044
306	GO:0002790
307	GO:0070208
308	GO:0045766
309	GO:0070506
310	GO:0005223
311	GO:0001502
312	GO:0070633
313	GO:0002504
314	GO:0015432
315	GO:0032784
316	GO:0070513
317	GO:0046813
318	GO:0031497
319	GO:0032809

320	GO:0003429
321	GO:0032488
322	GO:0005114
323	GO:0045116
324	GO:0007387
325	GO:0060527
326	GO:0048257
327	GO:0070051
328	GO:0090207
329	GO:0046911
330	GO:0045591
331	GO:0008209
332	GO:0020005
333	GO:0034505
334	GO:0038161
335	GO:0014065
336	GO:0007388
337	GO:0014896
338	GO:0090037
339	GO:0010821
340	GO:0005604
341	GO:0050689
342	GO:0051000
343	GO:0051873
344	GO:0031491
345	GO:0001300
346	GO:0004422
347	GO:0010165
348	GO:0006741
349	GO:0004925
350	GO:0042993
351	GO:0006707
352	GO:0020003
353	GO:0005577
354	GO:0001844
355	GO:0071062
356	GO:0045423
357	GO:0042622
358	GO:0010740
359	GO:0030516
360	GO:0097241
361	GO:0034188
362	GO:0009441

363	GO:0001578
364	GO:0005588
365	GO:0006999
366	GO:0035377
367	GO:0042267
368	GO:0051146
369	GO:0060763
370	GO:0006705
371	GO:0051651
372	GO:0071622
373	GO:0045779
374	GO:0002523
375	GO:0021681
376	GO:0070926
377	GO:0016272
378	GO:0001848
379	GO:1900142
380	GO:0005520
381	GO:0009249
382	GO:0030496
383	GO:0042095
384	GO:0070287
385	GO:0035092
386	GO:0010763
387	GO:0010917
388	GO:0035379
389	GO:0032805
390	GO:0032226
391	GO:0042698
392	GO:0033186
393	GO:0005883
394	GO:0045429
395	GO:0060020
396	GO:0035378
397	GO:0090303
398	GO:0042147
399	GO:2000048
400	GO:0005044
401	GO:0051098
402	GO:0045414
403	GO:0035458
404	GO:0000930
405	GO:0060545

406	GO:1900165
407	GO:0034185
408	GO:0048407
409	GO:0010764
410	GO:0085018
411	GO:0030492
412	GO:0035767
413	GO:0004873
414	GO:0002541
415	GO:0010498
416	GO:0019348
417	GO:0051001
418	GO:0019835
419	GO:2000533
420	GO:0007269
421	GO:0002537
422	hsa04974
423	GO:0030214
424	GO:0048185
425	GO:0002543
426	GO:0005522
427	GO:0042832
428	GO:0002062
429	GO:0009106
430	GO:0042405
431	GO:0005548
432	GO:0032786
433	GO:0010934
434	GO:2001033
435	GO:0048009
436	GO:0007568
437	GO:0016139
438	GO:0006428
439	GO:0032924
440	GO:0051345
441	GO:0031077
442	GO:0016056
443	GO:0008028
444	GO:0032269
445	GO:0005746
446	GO:0045541
447	GO:0008356
448	GO:0030574

449	GO:0031052
450	GO:0001522
451	GO:0006178
452	GO:0010863
453	GO:0015718
454	GO:0043394
455	GO:0002687
456	GO:0019430
457	GO:0050655
458	GO:0060452
459	GO:0004609
460	GO:0048549
461	GO:0030514
462	GO:0009583
463	GO:0043559
464	GO:0008262
465	GO:0046464
466	GO:0031065
467	GO:0043544
468	GO:0071593
469	GO:0002820
470	GO:0005072
471	hsa03013
472	GO:0007160
473	GO:0042801
474	GO:0014826
475	GO:0003131
476	GO:0006457
477	GO:0042660
478	GO:0001952
479	GO:0032757
480	GO:0012501
481	GO:0008061
482	GO:0010897
483	GO:0033185
484	GO:0005178
485	GO:0007256
486	GO:0009116
487	GO:0071712
488	GO:0051580
489	GO:0046083
490	GO:0032793
491	GO:0046907

492	GO:0090150
493	GO:0003006
494	GO:0048678
495	GO:2001051
496	GO:0051142
497	GO:0003828
498	GO:0015485
499	GO:0085029
500	GO:0048525

(6) Dataset D_6

a) MaxRel features list

Rank	Feature name
1	GO:0034436
2	GO:0034437
3	GO:0034189
4	GO:0038025
5	GO:1900006
6	GO:0030449
7	GO:0016495
8	GO:0043498
9	GO:0043537
10	GO:0071953
11	GO:2001028
12	GO:0043117
13	GO:0001970
14	GO:0031701
15	GO:0032855
16	GO:0010001
17	GO:0071482
18	GO:0002575
19	GO:0048739
20	GO:0031109
21	GO:0006290
22	hsa04610
23	GO:0043206
24	GO:0048844
25	GO:0090031
26	GO:0001300
27	GO:0034447
28	GO:0003273
29	GO:0060342
30	GO:0051400

31	GO:0043183
32	GO:0009409
33	GO:0010703
34	GO:0010711
35	GO:0060313
36	GO:0060311
37	GO:0034103
38	GO:0005010
39	GO:0072563
40	GO:0005172
41	GO:0045103
42	GO:0032757
43	GO:0005615
44	GO:0035645
45	GO:0043536
46	GO:0021762
47	GO:0033364
48	GO:0042231
49	GO:2000256
50	GO:0042033
51	GO:0042253
52	GO:0001938
53	GO:0051389
54	GO:0045429
55	GO:0060754
56	GO:0032805
57	GO:0042660
58	GO:0001315
59	GO:0051856
60	GO:0048773
61	GO:0046911
62	GO:0003069
63	GO:0051651
64	GO:0034021
65	GO:0005586
66	GO:0031012
67	GO:0002032
68	GO:0060319
69	GO:0010759
70	GO:0042095
71	GO:0050777
72	GO:0000303
73	GO:0002446

74	GO:0032467
75	GO:0034185
76	GO:0051580
77	GO:0060763
78	GO:0010625
79	GO:0002282
80	GO:0044464
81	GO:0030229
82	GO:0038026
83	GO:0060312
84	GO:0001974
85	GO:0038091
86	GO:1900086
87	GO:0001798
88	GO:0014896
89	GO:0000302
90	GO:0030949
91	GO:0009441
92	GO:0035767
93	GO:0014904
94	GO:0005548
95	GO:0002523
96	GO:0061045
97	GO:0035630
98	GO:0032930
99	GO:0032364
100	GO:0032809
101	GO:0043331
102	GO:0032432
103	GO:0042088
104	GO:0034346
105	GO:0002384
106	GO:0060644
107	GO:0046813
108	GO:0035924
109	GO:0006958
110	GO:0045766
111	GO:0002457
112	GO:0007497
113	GO:0001910
114	hsa05150
115	GO:0002693
116	GO:0010477

117	GO:0070091
118	GO:0051971
119	GO:0071222
120	GO:0050665
121	GO:0048246
122	GO:0005576
123	GO:0032463
124	GO:0007256
125	GO:0002021
126	GO:0070886
127	GO:0051272
128	GO:0002262
129	GO:0017158
130	GO:0030166
131	GO:0032700
132	GO:0033160
133	GO:0033627
134	GO:0004943
135	GO:0055091
136	GO:0002462
137	GO:0001850
138	GO:0006956
139	GO:0006957
140	GO:0004876
141	GO:0014043
142	GO:0032387
143	GO:0048678
144	GO:0060509
145	GO:0021517
146	GO:0060744
147	GO:0005072
148	GO:0043129
149	GO:0004866
150	GO:0034405
151	GO:0009881
152	GO:0009597
153	GO:0002551
154	GO:0048245
155	GO:0034363
156	GO:0019430
157	GO:0014909
158	GO:0033700
159	GO:0010764

160	GO:0005006
161	GO:0070301
162	GO:0004784
163	GO:0045541
164	GO:0060741
165	GO:0010863
166	GO:0051001
167	GO:0044146
168	GO:0060552
169	GO:0060550
170	GO:2000309
171	GO:0071351
172	GO:0042159
173	GO:0033141
174	GO:0060011
175	GO:0042056
176	GO:0048845
177	GO:0051142
178	GO:0070050
179	GO:0055099
180	GO:0032417
181	GO:0042583
182	GO:0052033
183	GO:0004962
184	GO:0046629
185	GO:0002429
186	GO:0055090
187	GO:0010519
188	GO:0001890
189	GO:0032958
190	GO:0050840
191	GO:0022617
192	GO:0042094
193	GO:0004944
194	GO:0045359
195	GO:0060228
196	GO:0061302
197	GO:0008395
198	GO:0046716
199	GO:0002042
200	GO:0060020
201	GO:0010288
202	GO:0060052

203	GO:0030104
204	GO:0019934
205	GO:0072332
206	GO:0033691
207	GO:0005534
208	GO:0032722
209	GO:0034393
210	GO:0071813
211	GO:0002291
212	GO:0070022
213	GO:0060510
214	GO:0070430
215	GO:0070434
216	GO:0016046
217	GO:0016942
218	GO:0043200
219	GO:0008209
220	GO:0032732
221	GO:0042149
222	GO:0071258
223	GO:0048549
224	GO:0061044
225	GO:0007181
226	GO:0035491
227	GO:0070887
228	GO:0070483
229	GO:0019836
230	GO:0001666
231	GO:0042832
232	GO:0071346
233	GO:0048635
234	GO:0000189
235	GO:0005581
236	GO:0032488
237	GO:2000098
238	GO:0055093
239	hsa05323
240	GO:0042698
241	GO:0048009
242	GO:0014826
243	GO:0050650
244	GO:0042060
245	GO:0060128

246	GO:0030247
247	GO:2000121
248	GO:0032270
249	GO:0050921
250	GO:0001895
251	GO:0006707
252	GO:0010189
253	GO:0060591
254	GO:0048598
255	GO:0002537
256	GO:2000366
257	GO:0070371
258	GO:0045188
259	GO:0051898
260	GO:0051000
261	GO:0034188
262	GO:0038027
263	GO:0002790
264	GO:0032510
265	GO:0008228
266	GO:0070670
267	GO:0007584
268	hsa05143
269	GO:0051926
270	GO:0008360
271	GO:2000379
272	GO:0060394
273	GO:0008090
274	GO:0048251
275	GO:0048407
276	GO:0005114
277	GO:0008217
278	GO:0006940
279	GO:0070373
280	GO:0001781
281	GO:0030235
282	GO:0033081
283	GO:0031953
284	GO:0051712
285	GO:0048593
286	GO:0003340
287	GO:0007250
288	GO:0033299

289	GO:0005041
290	GO:2000097
291	GO:0043576
292	GO:0042627
293	GO:0004252
294	GO:0050766
295	GO:0005905
296	GO:0007179
297	GO:0005578
298	GO:0035624
299	GO:0060068
300	GO:0045740
301	GO:0071456
302	GO:0030139
303	GO:0071356
304	GO:0034369
305	GO:0010900
306	GO:0004509
307	GO:0030516
308	GO:0010740
309	GO:0001569
310	GO:0051246
311	GO:0007568
312	GO:0030195
313	GO:0003032
314	GO:0051894
315	GO:0043388
316	GO:0021940
317	GO:0042116
318	GO:0031017
319	GO:0045721
320	GO:0071361
321	GO:0010875
322	GO:0006704
323	GO:0051659
324	GO:0016056
325	GO:0007263
326	GO:0007399
327	GO:0010902
328	GO:0032367
329	GO:0010916
330	GO:0004465
331	GO:0042158

332	GO:0034382
333	GO:0045953
334	GO:0034097
335	GO:0005585
336	GO:0005161
337	GO:0050927
338	GO:0051054
339	GO:0014910
340	GO:0002687
341	GO:0033591
342	GO:0045084
343	GO:0032287
344	GO:0031077
345	GO:2000288
346	GO:0001937
347	GO:0032878
348	GO:0001957
349	GO:0009408
350	GO:0060087
351	GO:0001968
352	GO:0001540
353	GO:0060452
354	GO:0051873
355	GO:0005520
356	GO:0032784
357	GO:0042612
358	GO:0019064
359	GO:0072126
360	GO:0010873
361	GO:0072264
362	GO:0006898
363	GO:2000591
364	GO:0072255
365	GO:0051005
366	GO:0005133
367	GO:0046620
368	GO:0030214
369	GO:0051280
370	GO:0016004
371	GO:0008083
372	GO:0047485
373	GO:0090197
374	GO:0006917

375	GO:0001502
376	GO:0009583
377	GO:0051602
378	GO:0015643
379	GO:0032691
380	GO:0002690
381	GO:0045725
382	GO:0009611
383	GO:0042092
384	GO:0070326
385	GO:0034605
386	GO:0010951
387	GO:0044241
388	GO:0060697
389	GO:0010760
390	GO:0010897
391	GO:0031714
392	GO:0055102
393	GO:0034358
394	GO:0045742
395	GO:0019049
396	GO:0072308
397	GO:0042307
398	GO:0046677
399	GO:0043559
400	GO:0043154
401	GO:0032269
402	GO:0002576
403	GO:0003084
404	GO:0042310
405	GO:0031702
406	GO:0060084
407	GO:0008289
408	GO:0034362
409	GO:0035473
410	GO:0035478
411	GO:0017038
412	GO:0090321
413	GO:0001567
414	GO:0006869
415	GO:0070287
416	GO:0034041
417	GO:0050051

418	GO:0052871
419	GO:0021858
420	GO:0010874
421	GO:0009720
422	GO:0010872
423	GO:0071503
424	GO:0031999
425	GO:0045884
426	GO:0005158
427	GO:0014065
428	GO:0032496
429	GO:0010512
430	GO:0008201
431	GO:0010224
432	GO:0070541
433	GO:0050995
434	GO:0045348
435	hsa05144
436	GO:0051346
437	GO:0030155
438	GO:0007565
439	GO:0014834
440	GO:0006029
441	GO:0051918
442	GO:0071062
443	GO:0032181
444	GO:0000406
445	GO:0001991
446	GO:0001543
447	GO:0033602
448	GO:0032226
449	GO:0032302
450	GO:0042953
451	GO:0008009
452	GO:2000048
453	GO:0015920
454	GO:0015485
455	hsa05020
456	GO:0042267
457	GO:0033552
458	GO:0090050
459	GO:2000378
460	GO:0045906

461	GO:0010757
462	GO:0048661
463	GO:0003823
464	GO:0045079
465	GO:0060729
466	GO:0010763
467	GO:0090037
468	GO:0051044
469	GO:0060740
470	GO:0043184
471	GO:0008089
472	GO:0034392
473	GO:0033025
474	GO:0032368
475	GO:0031726
476	GO:0071506
477	GO:0031663
478	GO:0032494
479	GO:0032964
480	GO:0031362
481	GO:0071347
482	hsa05416
483	GO:0070093
484	GO:0018298
485	GO:0043491
486	GO:0002028
487	GO:0006700
488	GO:0043066
489	GO:0042535
490	GO:0010165
491	GO:0002666
492	GO:0030838
493	GO:0045785
494	GO:0045630
495	GO:0071230
496	GO:0005537
497	GO:0010466
498	GO:2001141
499	GO:0002218
500	hsa05133

b) mRMR features list

Rank	Feature name

1	GO:0034436
2	GO:0006956
3	GO:0048739
4	GO:0031701
5	GO:0030449
6	GO:0050777
7	GO:1900006
8	GO:0060744
9	GO:0043498
10	GO:0071482
11	GO:0034437
12	GO:0046629
13	hsa04610
14	GO:0005586
15	GO:0008395
16	GO:0034189
17	GO:0002032
18	GO:0035767
19	GO:0033364
20	GO:0014909
21	GO:0016495
22	GO:0031012
23	GO:0002429
24	GO:0071953
25	GO:0002446
26	GO:0005581
27	GO:0032364
28	GO:0043206
29	GO:0010759
30	GO:0010001
31	GO:0006958
32	GO:0055090
33	GO:0043117
34	GO:0032417
35	GO:0038025
36	GO:0055099
37	GO:0001970
38	GO:0042612
39	GO:0005548
40	GO:0031362
41	GO:0043537
42	GO:0045953
43	GO:2001028

44	GO:0004509
45	GO:0032855
46	GO:0007181
47	GO:0010519
48	GO:0006290
49	GO:0051272
50	GO:0006957
51	GO:0032463
52	GO:0005010
53	GO:0048549
54	GO:0055091
55	GO:0061302
56	GO:0048844
57	GO:0047485
58	GO:0004943
59	GO:0060342
60	GO:0004866
61	GO:0072563
62	GO:0015355
63	GO:0060011
64	GO:0060754
65	GO:0010189
66	GO:0031109
67	GO:0032226
68	GO:0030247
69	GO:0032510
70	GO:0021858
71	GO:0070050
72	GO:0050840
73	GO:0002462
74	GO:0043183
75	GO:0033700
76	GO:0006168
77	GO:0001315
78	GO:0035624
79	GO:0008289
80	GO:0035645
81	GO:0051873
82	GO:0038026
83	GO:0042698
84	GO:0051400
85	GO:0001850
86	GO:0005578

87	GO:0002575
88	GO:0006869
89	GO:0034447
90	GO:0042149
91	GO:0003273
92	GO:0038063
93	GO:0060052
94	GO:0001974
95	GO:0004876
96	GO:0034362
97	GO:0045103
98	GO:0061045
99	GO:0005537
100	GO:0033299
101	GO:0048593
102	GO:0000302
103	GO:0035473
104	GO:0010703
105	GO:0005585
106	GO:0006704
107	hsa05150
108	GO:0034041
109	GO:1900086
110	GO:0034185
111	hsa04974
112	GO:0002523
113	GO:0001300
114	GO:0035478
115	GO:0009881
116	GO:0000003
117	GO:0010711
118	GO:0005610
119	GO:0048773
120	GO:0009720
121	GO:0007179
122	GO:0034369
123	GO:0015718
124	GO:0038091
125	GO:0042583
126	GO:0017038
127	GO:0060313
128	GO:0032691
129	GO:0007497

130	GO:0019064
131	GO:0005597
132	GO:0001798
133	GO:0010872
134	GO:0005615
135	GO:0090321
136	GO:0006741
137	GO:0072332
138	GO:0060311
139	GO:0042147
140	GO:0051856
141	GO:0034605
142	GO:0030229
143	GO:0008228
144	GO:0005172
145	GO:0008028
146	GO:0010900
147	GO:0090031
148	GO:0008271
149	GO:0005883
150	GO:0070287
151	GO:0048407
152	GO:0034103
153	GO:0003069
154	GO:0010874
155	GO:0032930
156	GO:0090305
157	GO:0043576
158	GO:0042627
159	GO:0072686
160	GO:0050766
161	GO:0071503
162	GO:2001141
163	GO:0022617
164	GO:0032181
165	GO:0033691
166	GO:0032757
167	GO:0008272
168	GO:0034188
169	GO:0034021
170	GO:0006029
171	GO:0060319
172	GO:0060229

173	GO:0001778
174	GO:0060510
175	GO:0071258
176	GO:0034122
177	GO:0035924
178	GO:0044241
179	GO:0070052
180	GO:0046813
181	GO:0000406
182	GO:0001867
183	GO:0004252
184	GO:0001567
185	GO:0005201
186	GO:0000303
187	GO:0004944
188	GO:0061044
189	GO:0005520
190	GO:0038027
191	GO:0021517
192	GO:0007603
193	GO:0030621
194	GO:0017158
195	GO:0034755
196	GO:0051635
197	GO:2000097
198	GO:0051580
199	GO:0010501
200	GO:0002031
201	GO:0035491
202	hsa00290
203	GO:0042231
204	GO:0002790
205	GO:0032302
206	GO:0043536
207	GO:0008186
208	GO:0051270
209	GO:0018184
210	GO:0005114
211	GO:0010625
212	GO:0090281
213	GO:0071813
214	GO:0043129
215	GO:0010760

216	GO:0060084
217	GO:0060068
218	GO:0004962
219	GO:0003999
220	GO:2000256
221	GO:0035375
222	GO:0001786
223	GO:0006707
224	GO:0070887
225	GO:0031491
226	GO:0060123
227	GO:0031714
228	GO:0044464
229	GO:0060452
230	GO:0060394
231	GO:0060228
232	GO:0006165
233	GO:0043152
234	GO:0042033
235	GO:0001502
236	GO:2000098
237	GO:0031573
238	GO:0033627
239	GO:0006705
240	GO:0051389
241	GO:0019882
242	GO:0002042
243	GO:0045254
244	GO:0015074
245	GO:0060763
246	GO:0035379
247	hsa00450
248	GO:0050051
249	GO:0032784
250	GO:0005577
251	GO:0050665
252	GO:0030166
253	GO:0034358
254	GO:0010740
255	GO:0019237
256	GO:0042253
257	GO:0052871
258	GO:0048273

259	GO:0042158
260	GO:0006228
261	GO:0010288
262	GO:0015728
263	GO:0034405
264	GO:0046836
265	GO:0030070
266	GO:0045116
267	GO:0021762
268	GO:0055092
269	GO:0035378
270	GO:0002262
271	GO:0010902
272	GO:0032432
273	GO:0002055
274	GO:0016056
275	GO:0003823
276	GO:0034375
277	GO:0007184
278	GO:0014910
279	GO:0060644
280	GO:0015130
281	GO:0002666
282	GO:0014043
283	GO:0045046
284	GO:0010388
285	GO:0016209
286	GO:0001574
287	GO:0007256
288	GO:0004465
289	GO:0070195
290	GO:0042056
291	GO:0021678
292	GO:0034382
293	GO:0035377
294	GO:0033025
295	GO:0005041
296	GO:0006183
297	GO:0032809
298	GO:1900025
299	GO:0032964
300	GO:0032467
301	GO:0032367

302	GO:0033602
303	GO:0060591
304	GO:0030949
305	GO:0045796
306	GO:0045128
307	GO:0043331
308	GO:0005539
309	GO:0033145
310	GO:0034363
311	GO:1900028
312	GO:0001543
313	GO:0015116
314	GO:0050655
315	GO:0002282
316	GO:0034365
317	GO:0042310
318	GO:0070022
319	GO:0004473
320	GO:0043654
321	GO:0070698
322	GO:0097186
323	GO:0009409
324	GO:0015168
325	GO:0005319
326	GO:0006700
327	GO:0010951
328	GO:0019430
329	GO:0001537
330	GO:0006311
331	GO:0032393
332	GO:0014826
333	GO:0032994
334	GO:0048246
335	GO:0004550
336	GO:0004903
337	GO:0004181
338	GO:0030023
339	GO:0043184
340	GO:0006898
341	GO:0050790
342	GO:0043196
343	GO:0034445
344	GO:0051894

345	GO:0071622
346	GO:0003032
347	GO:0005604
348	GO:0016557
349	GO:0060312
350	GO:0003828
351	GO:0005223
352	GO:0002551
353	GO:0010949
354	GO:0003104
355	GO:0002204
356	GO:0046548
357	GO:0019836
358	GO:0006940
359	GO:0042632
360	GO:0042095
361	GO:0038062
362	GO:0006488
363	GO:0035630
364	GO:0034373
365	GO:0005006
366	GO:0030184
367	GO:0042660
368	GO:0042461
369	GO:0048814
370	GO:0005588
371	GO:0032368
372	GO:0032805
373	GO:0007601
374	GO:0005158
375	GO:0020003
376	GO:0004784
377	GO:0030199
378	GO:0051971
379	GO:0016556
380	GO:0006508
381	GO:0070278
382	GO:0020005
383	GO:0042267
384	GO:0046314
385	GO:0008209
386	GO:0035082
387	GO:0005905

388	GO:0030948
389	GO:0005576
390	GO:0021885
391	GO:0050953
392	GO:0051005
393	GO:0043159
394	GO:0046911
395	GO:0002543
396	GO:0043405
397	GO:0001938
398	GO:0003097
399	GO:0008396
400	GO:0010916
401	GO:0031593
402	GO:0032387
403	GO:0051148
404	GO:0016705
405	GO:0033783
406	GO:0010260
407	GO:0085018
408	GO:0060128
409	GO:0010747
410	GO:0042088
411	GO:0002541
412	GO:0004111
413	GO:0008191
414	GO:0045429
415	GO:0016189
416	GO:0007606
417	GO:0051651
418	GO:0071241
419	GO:0017112
420	GO:0001895
421	GO:0060041
422	GO:0010641
423	GO:0031077
424	GO:0009914
425	GO:0005969
426	GO:0009597
427	GO:0008187
428	GO:0071062
429	GO:0045910
430	GO:0050927

431	GO:0005813
432	GO:0009586
433	GO:0006497
434	GO:0010991
435	GO:0002767
436	GO:0055093
437	GO:0070483
438	GO:0008340
439	GO:0001890
440	GO:0006086
441	GO:0031702
442	GO:0016139
443	GO:0010764
444	GO:0051636
445	GO:0050746
446	GO:0030162
447	GO:0003723
448	GO:0005072
449	GO:0055106
450	GO:0042953
451	GO:0060509
452	GO:0060351
453	GO:0055102
454	GO:2001199
455	GO:0000461
456	GO:0048678
457	GO:0031616
458	GO:0050921
459	GO:0019835
460	GO:0018298
461	GO:0031953
462	GO:0015301
463	GO:0048598
464	GO:0035501
465	GO:0010466
466	GO:0060697
467	GO:0016817
468	GO:0034346
469	GO:0030532
470	GO:0090050
471	GO:0010934
472	GO:0048484
473	hsa01040

474	GO:0048845
475	GO:0048672
476	GO:0060047
477	GO:0003131
478	GO:0016715
479	GO:0005212
480	GO:0051926
481	GO:0009451
482	GO:0060090
483	GO:0019800
484	GO:0015643
485	GO:0001991
486	GO:0030950
487	GO:0060806
488	GO:0070670
489	GO:0048535
490	GO:0019825
491	GO:0008253
492	GO:0002457
493	GO:0051661
494	GO:1900142
495	GO:0007183
496	GO:0051918
497	GO:0035987
498	GO:0008090
499	GO:0008747
500	GO:0007250

(7) Dataset D_7

a) MaxRel features list

Rank	Feature name
1	GO:0043537
2	GO:0030449
3	hsa05150
4	GO:0005586
5	GO:0001970
6	GO:0035645
7	GO:0016495
8	GO:0034436
9	GO:0034437
10	GO:0034189
11	GO:0048739
12	GO:0048844

13	GO:0006956
14	GO:0010711
15	GO:0034103
16	GO:0010703
17	GO:0060311
18	GO:0060313
19	GO:0035767
20	GO:1900006
21	GO:0002575
22	GO:0038025
23	GO:0004962
24	GO:0051400
25	GO:0001798
26	GO:0071482
27	GO:0031109
28	GO:0045359
29	GO:0032432
30	GO:0005615
31	GO:0004944
32	GO:0071953
33	GO:0060754
34	GO:0030949
35	GO:0043331
36	GO:0043206
37	GO:0006958
38	GO:0072563
39	GO:0043171
40	GO:0003273
41	GO:0034447
42	GO:0043183
43	GO:0007497
44	GO:0048245
45	GO:0005581
46	GO:0034346
47	GO:0002282
48	GO:0060011
49	GO:0010001
50	hsa04610
51	GO:0036020
52	GO:0045356
53	GO:0031012
54	GO:0043536
55	GO:0008228

56	GO:0032757
57	GO:0001300
58	GO:0032805
59	GO:0042088
60	GO:0051651
61	GO:0046911
62	GO:0006957
63	GO:0045766
64	GO:0060342
65	GO:0014043
66	GO:0035924
67	GO:0071222
68	GO:0001938
69	GO:0005172
70	GO:0002551
71	GO:0009597
72	GO:0005576
73	hsa05133
74	GO:0014826
75	hsa05146
76	GO:0000302
77	GO:0010863
78	hsa05323
79	GO:0004866
80	GO:0002042
81	GO:0043117
82	GO:1900086
83	GO:0034021
84	GO:0003069
85	GO:0043154
86	GO:0001315
87	GO:0045429
88	GO:0048773
89	GO:0019836
90	GO:0052033
91	GO:0010759
92	GO:0038091
93	GO:0006700
94	GO:0042159
95	GO:0001974
96	GO:0010716
97	GO:0032855
98	GO:0070430

99	GO:0051389
100	GO:0002537
101	GO:0016046
102	GO:0032732
103	GO:0070434
104	GO:0051873
105	GO:0006029
106	GO:0051607
107	GO:0060319
108	GO:0032964
109	GO:0019934
110	GO:0042832
111	GO:0008209
112	GO:0033627
113	GO:0007568
114	GO:0001569
115	GO:0008217
116	GO:0005125
117	GO:0043498
118	GO:0042554
119	GO:0001666
120	GO:0021762
121	GO:0009409
122	GO:0031077
123	GO:0043129
124	GO:0050777
125	GO:0001968
126	GO:0010740
127	GO:0002544
128	GO:0032269
129	GO:0002462
130	GO:0001850
131	GO:0004943
132	GO:0004876
133	GO:0006935
134	GO:0060394
135	GO:0030195
136	GO:0048845
137	GO:0048407
138	GO:2001028
139	GO:0006940
140	GO:0051856
141	GO:0070373

142	GO:0032467
143	GO:0002446
144	GO:0030198
145	GO:0001957
146	GO:0045671
147	GO:0055093
148	GO:0042060
149	GO:0071346
150	GO:0070541
151	GO:0038026
152	GO:0009881
153	GO:0005161
154	GO:0042583
155	GO:0002548
156	GO:0022617
157	GO:0004175
158	GO:0032463
159	GO:0061045
160	GO:0046813
161	GO:0045541
162	GO:0010466
163	GO:0004784
164	GO:0035665
165	GO:2000340
166	GO:0035663
167	GO:0032738
168	GO:0004509
169	GO:0035662
170	GO:0019826
171	GO:0051142
172	GO:0010575
173	GO:0005534
174	GO:0051894
175	GO:0042660
176	GO:0042116
177	GO:0021517
178	GO:0060527
179	GO:0030828
180	GO:0050665
181	GO:0032930
182	GO:0007179
183	GO:0033364
184	GO:0005585

185	GO:0006917
186	GO:0009611
187	GO:0051659
188	GO:0048246
189	GO:0005178
190	GO:0019064
191	GO:0043184
192	GO:0051881
193	GO:0090050
194	GO:0005010
195	GO:0050921
196	GO:0032700
197	GO:0042095
198	GO:0033141
199	GO:0009408
200	GO:0060346
201	GO:0032809
202	GO:0030593
203	GO:0048010
204	GO:2000256
205	GO:0070483
206	GO:0042033
207	GO:0007256
208	GO:0007263
209	GO:0042253
210	GO:0042231
211	GO:0030335
212	GO:0002429
213	GO:0046629
214	GO:0034154
215	GO:0002457
216	GO:0070301
217	GO:0001910
218	GO:0002693
219	GO:0032958
220	GO:0007565
221	GO:0070371
222	GO:0010477
223	GO:0002523
224	GO:0010875
225	GO:0030023
226	GO:0035988
227	hsa05140

228	GO:0051918
229	GO:0071260
230	GO:0046010
231	GO:0006955
232	GO:0034185
233	GO:0034363
234	GO:0032364
235	GO:0005072
236	GO:0045103
237	hsa05143
238	GO:0001937
239	GO:0001955
240	GO:0060312
241	GO:0030247
242	GO:0048598
243	GO:0090031
244	GO:0060068
245	GO:0051272
246	GO:0043200
247	GO:0008009
248	GO:0050927
249	GO:0006801
250	GO:0010763
251	GO:0031701
252	GO:0010595
253	GO:0004950
254	GO:0030020
255	GO:0048565
256	GO:0048009
257	GO:0031017
258	GO:0060129
259	GO:0071356
260	GO:0048251
261	GO:2000121
262	GO:0072573
263	GO:0070429
264	GO:0033192
265	GO:2000349
266	GO:0070433
267	GO:0034148
268	GO:0061043
269	GO:0034056
270	GO:0060729

271	GO:0019049
272	GO:0072332
273	GO:0000189
274	GO:0051000
275	GO:0009441
276	GO:0014896
277	GO:0014904
278	GO:2001199
279	GO:0004252
280	GO:0002767
281	GO:0032497
282	GO:0042092
283	GO:0033552
284	GO:0048661
285	GO:0060763
286	GO:0032722
287	GO:0007183
288	GO:0030229
289	GO:0034393
290	GO:0002758
291	GO:0000303
292	GO:0060128
293	GO:0051280
294	GO:0050953
295	GO:0045768
296	hsa05142
297	GO:0014065
298	GO:0032816
299	GO:0003417
300	GO:0034505
301	GO:0008083
302	GO:0071347
303	GO:0046620
304	GO:0048678
305	GO:0071351
306	GO:0031798
307	GO:2000309
308	GO:0044146
309	GO:0060509
310	GO:0060550
311	GO:0060552
312	GO:0051898
313	GO:0038052

314	GO:0030214
315	GO:0060745
316	GO:0042953
317	GO:0016209
318	GO:0042612
319	GO:0007181
320	GO:0048185
321	GO:0045869
322	GO:0005044
323	GO:0060697
324	GO:0010760
325	GO:0031714
326	GO:0005579
327	GO:2000502
328	GO:0042310
329	GO:0030199
330	GO:0061302
331	GO:0035684
332	GO:0070051
333	GO:0019882
334	GO:0005201
335	GO:0042493
336	GO:0042056
337	GO:0010625
338	GO:0044464
339	GO:0051098
340	GO:0051580
341	GO:0014910
342	GO:0009612
343	GO:0033591
344	GO:0001872
345	GO:0019430
346	GO:0060351
347	hsa05020
348	GO:2000379
349	GO:0022009
350	GO:0045348
351	GO:0032270
352	GO:0032695
353	GO:0032943
354	GO:0045084
355	GO:0070091
356	GO:0033081

357	GO:0090037
358	GO:0005006
359	GO:0060644
360	GO:0035630
361	GO:0002262
362	GO:0031953
363	GO:0071361
364	GO:0010764
365	GO:0051001
366	GO:0060749
367	GO:0009583
368	GO:0090197
369	GO:0002666
370	GO:0002218
371	GO:0048535
372	GO:0051591
373	hsa05144
374	GO:0050728
375	GO:0005520
376	GO:0016004
377	GO:0009615
378	GO:0060220
379	GO:0048074
380	GO:0010902
381	GO:0008061
382	GO:0002532
383	GO:0016525
384	GO:0043410
385	GO:0035458
386	GO:0010811
387	GO:0031663
388	GO:2000427
389	GO:0005578
390	GO:0050766
391	GO:0045740
392	GO:0015643
393	GO:0060020
394	GO:0030284
395	GO:0033160
396	GO:0043388
397	GO:0030516
398	GO:0030346
399	GO:0001781

400	GO:0009314
401	GO:0051926
402	GO:0055094
403	GO:0008191
404	GO:0008201
405	GO:0030169
406	GO:0009749
407	hsa04620
408	GO:0001503
409	GO:0006290
410	GO:0007252
411	GO:0001946
412	GO:0070098
413	GO:0010751
414	GO:0010754
415	GO:0008395
416	GO:0002581
417	GO:0002605
418	GO:0071312
419	GO:0021940
420	GO:0035066
421	GO:0005537
422	GO:0071062
423	GO:0010544
424	GO:0071230
425	GO:0045765
426	GO:0071359
427	GO:0050714
428	GO:0005114
429	GO:0070670
430	GO:0005577
431	GO:0045906
432	GO:0060137
433	GO:0008285
434	GO:0043277
435	GO:0002456
436	GO:0050829
437	GO:0048662
438	GO:0001786
439	GO:0007569
440	hsa05219
441	GO:0045188
442	GO:0003340

443	GO:2000669
444	GO:0070206
445	GO:0004906
446	GO:0055090
447	GO:0034382
448	GO:0034137
449	GO:0030166
450	GO:0008090
451	GO:0002291
452	GO:0045630
453	GO:0030279
454	GO:0002021
455	GO:0070022
456	GO:0060591
457	GO:0009897
458	GO:0032494
459	GO:0031727
460	GO:0008236
461	GO:0031702
462	GO:0072593
463	GO:0002062
464	GO:0090265
465	GO:0032735
466	GO:0032874
467	GO:0019062
468	GO:0060279
469	GO:0030171
470	GO:0043153
471	GO:0097186
472	GO:0051454
473	GO:0009590
474	GO:0046795
475	GO:0046649
476	GO:0071547
477	GO:0006746
478	GO:0070287
479	GO:0000003
480	GO:0050840
481	GO:0032717
482	GO:0048247
483	GO:0005584
484	GO:0005548
485	GO:0071223

486	GO:0017134
487	GO:0010512
488	GO:0001922
489	GO:0031093
490	GO:0002520
491	GO:0007603
492	hsa04672
493	GO:0071374
494	GO:0043066
495	GO:0060325
496	GO:0034145
497	GO:0030704
498	GO:0060545
499	GO:0019835
500	GO:0001991

b) mRMR features list

Rank	Feature name
1	GO:0043537
2	hsa05150
3	GO:0005586
4	GO:0030449
5	GO:0048739
6	GO:0005581
7	GO:0006956
8	GO:0035767
9	GO:0035645
10	GO:0002429
11	GO:0051873
12	GO:0048844
13	GO:0006958
14	GO:0031012
15	GO:0071482
16	GO:0034436
17	GO:0006029
18	GO:0001970
19	GO:0004509
20	GO:0060754
21	GO:0008228
22	GO:0043171
23	GO:0046629
24	GO:0004944
25	GO:0002446

26	GO:0016495
27	GO:0033192
28	hsa04610
29	GO:0035924
30	GO:0034437
31	hsa05146
32	GO:0060011
33	GO:0042612
34	GO:0006957
35	GO:0032964
36	GO:0043206
37	GO:0019064
38	GO:0010759
39	GO:0005585
40	GO:0014826
41	GO:0010740
42	GO:0004866
43	GO:0034189
44	GO:0007179
45	hsa05133
46	GO:0006700
47	GO:0036020
48	GO:0031701
49	GO:0000302
50	GO:0002462
51	GO:0019882
52	GO:0010001
53	GO:0001968
54	GO:0043331
55	GO:0042583
56	GO:0001798
57	GO:0030020
58	GO:1900006
59	GO:0019826
60	GO:0071777
61	GO:0001850
62	GO:0045356
63	GO:0048407
64	GO:0000003
65	GO:0004175
66	GO:0007497
67	GO:0008395
68	GO:0050777

69	GO:0045359
70	GO:0004943
71	GO:0038091
72	GO:0008236
73	GO:0030023
74	GO:0030247
75	GO:0035988
76	GO:0004962
77	GO:2001199
78	GO:0002042
79	GO:0038025
80	GO:0004876
81	GO:0002544
82	GO:0009597
83	GO:0070287
84	GO:0038063
85	GO:0001974
86	GO:1900086
87	GO:0010711
88	GO:0051272
89	GO:0043229
90	GO:0005537
91	GO:0071953
92	GO:0002767
93	GO:0043536
94	GO:0051881
95	GO:0043153
96	GO:0034103
97	GO:0001957
98	GO:0019836
99	GO:0072573
100	GO:0010703
101	GO:0072384
102	GO:0060841
103	GO:0048535
104	GO:0060311
105	GO:0097186
106	GO:0060394
107	GO:0005044
108	GO:0031077
109	GO:0060313
110	GO:0009749
111	GO:0051607

112	GO:0007603
113	GO:0002820
114	GO:0005576
115	GO:0090050
116	GO:0070429
117	GO:0003828
118	GO:0031109
119	GO:0010763
120	GO:0051400
121	GO:0005588
122	GO:0070206
123	GO:0010863
124	GO:0031362
125	GO:0016209
126	GO:0006940
127	GO:0070052
128	GO:0032757
129	GO:2000349
130	GO:0002575
131	GO:0034122
132	GO:0004252
133	GO:0032432
134	GO:0002666
135	GO:0007181
136	GO:0005615
137	GO:0019835
138	GO:0019934
139	GO:0070433
140	GO:0005201
141	GO:2001028
142	GO:0016362
143	GO:0060342
144	GO:0034154
145	GO:0035624
146	GO:0042554
147	GO:0038062
148	GO:0034148
149	GO:0030949
150	GO:0007183
151	GO:0035665
152	GO:0042613
153	GO:0042088
154	GO:0000254

155	GO:0005577
156	GO:0033364
157	GO:0061043
158	GO:0072563
159	GO:0006705
160	GO:2000340
161	GO:0034447
162	GO:0010760
163	GO:0060220
164	GO:0043117
165	GO:0050921
166	GO:0051683
167	GO:0034365
168	GO:0034021
169	GO:0001946
170	GO:0014043
171	GO:0030199
172	GO:0035663
173	GO:0005172
174	GO:0031714
175	GO:0002504
176	GO:0032738
177	GO:0060744
178	GO:0032463
179	GO:0048245
180	GO:0048074
181	GO:0035662
182	GO:0009881
183	GO:0051098
184	hsa05323
185	GO:0001574
186	GO:0038026
187	GO:0001867
188	GO:0061302
189	GO:0032816
190	GO:0002282
191	GO:0015722
192	GO:0070051
193	GO:0005579
194	GO:0071168
195	GO:0005161
196	GO:0003069
197	GO:0035082

198	GO:0008217
199	GO:0033627
200	GO:0043183
201	GO:0060279
202	GO:0008187
203	GO:0034346
204	GO:0060706
205	GO:0005010
206	GO:0051280
207	GO:0060697
208	GO:0072686
209	GO:0043184
210	GO:0060765
211	GO:0001300
212	GO:0045423
213	GO:0001569
214	GO:0034605
215	GO:0008209
216	GO:0004169
217	GO:0050689
218	GO:2000669
219	GO:0001315
220	GO:0051148
221	GO:0043129
222	GO:0072332
223	GO:0010189
224	GO:0022617
225	GO:0070584
226	GO:0042953
227	GO:0002523
228	GO:0071222
229	GO:0051894
230	GO:0060052
231	GO:0046548
232	GO:0045869
233	GO:0048773
234	GO:0003417
235	GO:0010501
236	GO:0061045
237	GO:0045414
238	GO:0006704
239	GO:0003273
240	hsa04974

241	GO:0030621
242	GO:0032269
243	GO:0001786
244	GO:0060123
245	GO:0031491
246	GO:0030198
247	GO:0002551
248	GO:0055090
249	GO:0060319
250	GO:0002480
251	GO:0070815
252	GO:0010466
253	GO:0034755
254	GO:0006935
255	GO:0045953
256	GO:0030195
257	GO:0050766
258	GO:0018395
259	GO:0050927
260	GO:0010751
261	GO:0010902
262	GO:0044140
263	GO:0009615
264	GO:0019049
265	GO:0051389
266	GO:0070078
267	GO:0051653
268	GO:0032855
269	GO:0007263
270	GO:0060806
271	GO:0001727
272	GO:0033691
273	GO:0042159
274	GO:0010700
275	GO:0032695
276	GO:0010991
277	GO:0033005
278	GO:0060068
279	GO:0050953
280	GO:0070079
281	GO:0010754
282	GO:0032090
283	GO:0052033

284	GO:0043509
285	GO:0032805
286	GO:0005582
287	GO:0046834
288	GO:0060346
289	GO:0033749
290	GO:0030496
291	GO:0070541
292	GO:0007184
293	GO:0042701
294	GO:0048010
295	GO:0004906
296	GO:0051651
297	GO:0021681
298	GO:0043654
299	GO:0004222
300	GO:0046649
301	GO:0032364
302	GO:0046880
303	GO:0033746
304	GO:0070373
305	GO:0042461
306	GO:0002581
307	GO:0046911
308	GO:0005125
309	GO:0045254
310	GO:0051918
311	GO:0006741
312	GO:0001938
313	GO:0030054
314	GO:0010875
315	GO:0008191
316	GO:0060527
317	GO:0009893
318	GO:0006438
319	GO:0043154
320	GO:0060229
321	GO:0042310
322	GO:0017134
323	GO:0032510
324	GO:0003823
325	GO:0031953
326	GO:0045309

327	GO:0060090
328	GO:0045116
329	GO:0021517
330	GO:0002605
331	GO:0042116
332	GO:0007568
333	GO:0045494
334	GO:0004832
335	GO:0060681
336	GO:0045209
337	GO:0030171
338	GO:0071529
339	GO:0070050
340	GO:0030214
341	GO:0009409
342	GO:0045204
343	GO:0006168
344	GO:0071253
345	GO:0046332
346	GO:0070430
347	GO:0031616
348	GO:0048565
349	GO:0097193
350	GO:0051454
351	GO:0045766
352	GO:0034145
353	GO:0006746
354	GO:0019062
355	GO:0010369
356	GO:0008312
357	GO:0015126
358	GO:0002537
359	GO:0072163
360	GO:0010455
361	GO:0046795
362	GO:0051659
363	GO:0006290
364	GO:0030169
365	GO:0010575
366	GO:0055093
367	GO:0034363
368	GO:0010454
369	GO:0043277

370	GO:0055087
371	GO:0004784
372	GO:0090207
373	GO:0006917
374	GO:0003987
375	GO:0010716
376	GO:0015432
377	GO:0042060
378	GO:0003854
379	GO:0002430
380	GO:0016046
381	GO:0046947
382	GO:0000930
383	GO:0008237
384	GO:0032467
385	GO:0044241
386	GO:0002548
387	GO:0008241
388	GO:0008475
389	GO:0042267
390	GO:0032930
391	GO:0008554
392	GO:0032637
393	GO:0005592
394	GO:0005178
395	GO:0032732
396	GO:0001578
397	GO:0005583
398	GO:0002032
399	GO:0001844
400	GO:0003085
401	GO:0051856
402	GO:0006801
403	GO:0030704
404	GO:0070633
405	GO:0030574
406	GO:0046010
407	GO:0003429
408	GO:0090037
409	GO:0004473
410	GO:0070434
411	GO:0005602
412	GO:0008354

413	GO:0010498
414	GO:0055106
415	GO:0090101
416	GO:0032691
417	GO:0034137
418	GO:0042832
419	GO:0034361
420	GO:1900165
421	GO:0005114
422	GO:0043394
423	GO:0005534
424	GO:0043178
425	GO:0050665
426	GO:0048593
427	GO:0015355
428	GO:0005520
429	GO:0019962
430	GO:0004950
431	GO:0010917
432	GO:0001955
433	GO:0034382
434	GO:1900142
435	GO:0071547
436	GO:0033299
437	GO:0048246
438	GO:0033116
439	GO:0048185
440	GO:0032909
441	GO:0004905
442	GO:0043064
443	GO:0006710
444	GO:0030279
445	GO:0046813
446	GO:0032809
447	GO:0008277
448	GO:0003131
449	GO:0043498
450	GO:0000910
451	GO:0002262
452	GO:0060351
453	GO:0043159
454	GO:0042462
455	GO:0060370

456	GO:0050711
457	GO:0009583
458	GO:0032717
459	GO:0032943
460	GO:0021678
461	GO:0048845
462	GO:0009590
463	GO:0008200
464	GO:0035987
465	GO:0019439
466	GO:0005578
467	GO:0032481
468	GO:0010934
469	GO:0051654
470	GO:0001666
471	GO:0048273
472	GO:0032784
473	GO:0051591
474	GO:0035473
475	GO:0045671
476	GO:0045076
477	GO:0033602
478	GO:0001937
479	GO:0061153
480	GO:0005584
481	GO:0030346
482	GO:0032497
483	GO:0071158
484	GO:0034056
485	GO:0071208
486	GO:0005604
487	GO:0009611
488	GO:0046814
489	GO:0070722
490	GO:0071260
491	GO:0050728
492	GO:0016922
493	GO:0007256
494	GO:2000533
495	GO:0090321
496	GO:0071622
497	GO:0001848
498	GO:0045906

499	GO:0014909
500	GO:0071346

(8) Dataset D_8

a) MaxRel features list

Rank	Feature name
1	GO:0030449
2	GO:0034189
3	GO:0034437
4	GO:0034436
5	GO:0038025
6	GO:0006956
7	GO:0071482
8	GO:0035645
9	hsa04610
10	hsa05150
11	GO:0001970
12	GO:0006958
13	GO:0034447
14	GO:0016495
15	GO:0071953
16	GO:0006957
17	GO:0008228
18	GO:0004962
19	GO:0060342
20	GO:0032805
21	GO:0051651
22	GO:0046911
23	GO:0045359
24	GO:0010711
25	GO:0060311
26	GO:0038026
27	GO:0034103
28	GO:0032463
29	GO:0060313
30	GO:0010703
31	GO:0051400
32	GO:0014043
33	GO:0090031
34	GO:0034185
35	GO:0043536
36	GO:0001798
37	GO:0060011

38	GO:0032467
39	GO:0051389
40	GO:0031109
41	GO:0042583
42	GO:0042088
43	GO:0048773
44	GO:0001315
45	GO:0051000
46	GO:0034021
47	GO:0003069
48	GO:0033364
49	GO:0042159
50	GO:2001028
51	GO:0043537
52	GO:0007497
53	GO:0001938
54	GO:0003273
55	GO:0004944
56	GO:0021762
57	GO:0060228
58	GO:0045103
59	GO:0034346
60	GO:0043183
61	GO:0002575
62	GO:0051856
63	GO:0005172
64	GO:0043498
65	GO:0032855
66	GO:0051580
67	GO:0010625
68	GO:0044464
69	GO:0051898
70	GO:0060754
71	GO:0043117
72	GO:1900006
73	GO:0004943
74	GO:0004876
75	GO:0001850
76	GO:0002462
77	GO:0045766
78	GO:0043331
79	GO:0032809
80	GO:0071222

81	GO:0045429
82	GO:0050665
83	GO:0001300
84	GO:0002262
85	GO:0061302
86	GO:0032432
87	GO:0019836
88	GO:0030229
89	GO:0004866
90	GO:0008209
91	GO:0042660
92	GO:0030235
93	GO:0033081
94	GO:0009409
95	GO:0048844
96	GO:1900086
97	GO:0019430
98	GO:0000303
99	GO:0032757
100	GO:0035624
101	GO:0042832
102	GO:0002282
103	GO:2000256
104	GO:0042231
105	GO:0042033
106	GO:0042253
107	GO:0009881
108	GO:0052033
109	GO:0070430
110	GO:0002537
111	GO:0016046
112	GO:0070434
113	GO:0045541
114	GO:0042095
115	GO:0032732
116	GO:0048598
117	GO:0030949
118	GO:0048246
119	GO:0009583
120	GO:0006290
121	GO:0000302
122	GO:0046813
123	GO:0019934

124	GO:0060319
125	GO:0005072
126	GO:0033627
127	GO:0021517
128	GO:0060129
129	GO:0001910
130	GO:0032700
131	GO:0002693
132	GO:0010477
133	GO:0004784
134	GO:0002457
135	GO:0050777
136	GO:0032958
137	GO:0072563
138	GO:0034405
139	GO:0002042
140	GO:0030023
141	GO:0060312
142	GO:0051881
143	GO:0001569
144	GO:0050766
145	GO:0051272
146	GO:0035924
147	GO:0046620
148	GO:0072332
149	GO:0010001
150	GO:0031798
151	GO:0038052
152	GO:0004252
153	GO:0055093
154	GO:0048661
155	GO:0060346
156	GO:0048245
157	GO:0002551
158	GO:0060052
159	GO:0008089
160	GO:0031017
161	GO:0060128
162	GO:0048845
163	GO:0048009
164	GO:0009408
165	GO:0001895
166	GO:0010875

167	GO:0048018
168	GO:0007256
169	GO:0014826
170	GO:0070541
171	GO:0042094
172	GO:0000189
173	GO:0035630
174	GO:0090037
175	GO:0009441
176	GO:0014904
177	GO:0014896
178	GO:0060744
179	GO:0019064
180	GO:0002429
181	GO:0046629
182	GO:0009597
183	GO:0046907
184	GO:0070670
185	GO:0042462
186	GO:0030346
187	GO:0003823
188	GO:0071813
189	GO:0071346
190	GO:0055106
191	GO:0018242
192	GO:0018243
193	GO:0010466
194	GO:0050921
195	GO:0002291
196	GO:0051894
197	GO:2000121
198	GO:0048678
199	GO:0032497
200	GO:0017158
201	GO:0007603
202	GO:0007584
203	GO:0005586
204	GO:0014911
205	GO:0006801
206	GO:0032510
207	GO:0033700
208	GO:0006707
209	GO:0031102

210	GO:0004175
211	GO:0070326
212	GO:0051712
213	GO:0006940
214	GO:0008090
215	GO:0043200
216	GO:0003032
217	GO:0032364
218	GO:0031954
219	GO:0030247
220	GO:0061045
221	GO:0008395
222	GO:0008236
223	GO:0007181
224	GO:0033299
225	GO:0015920
226	GO:0060745
227	GO:0048739
228	GO:0021940
229	GO:0005010
230	GO:0071062
231	GO:0001666
232	GO:0051918
233	GO:0060763
234	GO:0001937
235	GO:0032722
236	GO:0010863
237	GO:0005615
238	GO:0060509
239	GO:0033141
240	GO:0060020
241	GO:0031714
242	GO:0010760
243	GO:0001867
244	GO:0035082
245	GO:0070373
246	GO:0060527
247	GO:0070301
248	GO:0034392
249	GO:0030195
250	GO:0005534
251	GO:0046716
252	GO:0090201

253	GO:0070022
254	GO:0002523
255	GO:0005585
256	GO:0035473
257	GO:0090321
258	GO:0000003
259	GO:0035478
260	GO:0033961
261	GO:0071503
262	GO:0017038
263	GO:0001567
264	GO:0032417
265	GO:0002576
266	GO:0051873
267	GO:0031093
268	GO:0001540
269	GO:0010716
270	GO:0006749
271	GO:0030838
272	GO:0043654
273	GO:0060550
274	GO:0060552
275	GO:0044146
276	GO:2000309
277	GO:0071351
278	GO:0048549
279	GO:0005178
280	GO:0033552
281	GO:0001974
282	GO:0007568
283	GO:0043154
284	GO:0048386
285	GO:0043325
286	GO:0051246
287	GO:0046677
288	GO:0010764
289	GO:0030516
290	GO:0060740
291	GO:0038063
292	GO:0004509
293	GO:0006029
294	GO:0051607
295	GO:0035458

296	GO:0051788
297	GO:0005548
298	GO:0010804
299	GO:0010224
300	GO:0042149
301	GO:2000379
302	GO:0051659
303	GO:0060068
304	GO:0051602
305	GO:0010033
306	GO:0048565
307	GO:0002666
308	GO:0051142
309	GO:0032269
310	GO:0060729
311	GO:0072573
312	GO:2000349
313	GO:0061043
314	GO:0070429
315	GO:0034148
316	GO:0070433
317	GO:0048305
318	GO:0034363
319	GO:0048666
320	GO:0032488
321	GO:0010512
322	GO:0045599
323	GO:0007569
324	GO:0015643
325	GO:0050927
326	GO:0070483
327	GO:0048251
328	GO:0001917
329	GO:0060750
330	GO:0008360
331	GO:0043559
332	GO:0010888
333	GO:0038091
334	GO:0010763
335	GO:0000060
336	GO:0030166
337	GO:0060087
338	GO:0005579

339	GO:0042461
340	GO:0055090
341	GO:0005041
342	GO:0016176
343	GO:0002839
344	GO:0045869
345	GO:0072255
346	GO:2000591
347	GO:0072126
348	GO:0072264
349	GO:0043277
350	GO:0072593
351	GO:0045179
352	GO:0046010
353	GO:0043066
354	GO:0043405
355	GO:0070050
356	GO:0033602
357	GO:0010902
358	GO:0004301
359	GO:2000288
360	GO:0030145
361	hsa05133
362	GO:0051346
363	GO:0002218
364	GO:0001968
365	GO:0007263
366	GO:0001955
367	GO:0060644
368	GO:0070371
369	GO:0051926
370	GO:0034393
371	GO:0045348
372	GO:0051054
373	GO:0030828
374	GO:0002758
375	GO:0031077
376	GO:0001922
377	GO:0003104
378	GO:0008047
379	GO:0031232
380	GO:0060557
381	GO:0045884

382	GO:0007606
383	GO:0010544
384	GO:0071356
385	GO:0006809
386	GO:0031663
387	GO:0032793
388	GO:0035684
389	GO:0008217
390	GO:0051023
391	hsa05323
392	GO:2000502
393	GO:0046668
394	GO:0002021
395	GO:0001781
396	GO:0060741
397	GO:0045356
398	GO:0007250
399	GO:0036020
400	GO:0004517
401	GO:0032368
402	GO:0004653
403	GO:0034369
404	GO:0006741
405	GO:0070506
406	GO:0010900
407	GO:0002891
408	GO:0004903
409	GO:0070633
410	GO:0009100
411	GO:0070195
412	GO:0030139
413	GO:0042092
414	GO:0030308
415	GO:0010759
416	GO:0042056
417	GO:0071456
418	GO:0016056
419	GO:0046688
420	GO:0051280
421	GO:0003009
422	GO:0002446
423	GO:0045188
424	GO:0046548

425	GO:0006935
426	GO:0015485
427	GO:0060220
428	GO:0030704
429	GO:0048074
430	GO:0006537
431	GO:0001848
432	GO:0055087
433	GO:0021885
434	GO:0005044
435	GO:0034358
436	GO:0019730
437	GO:0019865
438	GO:0032996
439	GO:0010519
440	GO:0034382
441	GO:0033093
442	hsa00512
443	GO:0032930
444	GO:0044429
445	GO:0030214
446	GO:0033145
447	GO:0055094
448	GO:0022009
449	GO:0032878
450	GO:0060510
451	GO:0043184
452	GO:0031953
453	GO:0050790
454	GO:0014834
455	GO:0014806
456	GO:0032332
457	GO:0001875
458	GO:0070542
459	GO:0002020
460	GO:0006917
461	GO:0031667
462	GO:0042511
463	GO:0045765
464	GO:0031362
465	GO:0071347
466	GO:0042622
467	GO:0043206

468	GO:0046696
469	GO:0033160
470	GO:0045630
471	GO:0030336
472	GO:2001141
473	GO:0042311
474	GO:0005462
475	GO:0000254
476	GO:0015788
477	GO:0048242
478	GO:0002820
479	GO:0032784
480	GO:0051005
481	GO:0030169
482	GO:0034186
483	GO:2000048
484	GO:0004489
485	GO:0071253
486	GO:0061338
487	GO:0006562
488	GO:0001923
489	GO:0038062
490	GO:0010189
491	GO:0033005
492	GO:0002064
493	GO:0085029
494	GO:0043388
495	GO:0043570
496	GO:0010744
497	GO:0031134
498	GO:0008201
499	GO:0060445
500	GO:0002548

b) mRMR features list

Rank	Feature name
1	GO:0030449
2	GO:0032463
3	GO:0060754
4	GO:0006956
5	GO:0034189
6	GO:0071482
7	GO:0002429

8	hsa04610
9	GO:0035645
10	GO:0000003
11	GO:0008228
12	GO:0034437
13	GO:0006958
14	GO:0030023
15	GO:0042583
16	GO:0060744
17	GO:0006957
18	GO:0038025
19	GO:0008395
20	GO:0055106
21	hsa05150
22	GO:0048018
23	GO:0034436
24	GO:0033961
25	GO:0060011
26	GO:0038063
27	GO:0016495
28	GO:0046629
29	GO:0033364
30	GO:0060342
31	GO:0048739
32	GO:0004943
33	GO:0060228
34	GO:0061302
35	GO:0005462
36	GO:0001970
37	GO:0035624
38	GO:0071953
39	GO:0050777
40	GO:0035473
41	GO:0038026
42	GO:0001867
43	GO:0002575
44	GO:0015788
45	GO:0004962
46	GO:0018242
47	GO:0051400
48	GO:0004509
49	GO:0007603
50	GO:0034447

51	GO:0090321
52	GO:0035082
53	GO:0004866
54	GO:0060346
55	GO:0034185
56	GO:0004876
57	GO:0045359
58	GO:0042462
59	hsa00512
60	GO:0002820
61	GO:0051873
62	GO:0007497
63	GO:0031616
64	GO:0035478
65	GO:0005585
66	GO:0043537
67	GO:0001850
68	GO:0018243
69	GO:2001028
70	GO:0072332
71	GO:0031362
72	GO:0048773
73	GO:0071503
74	GO:0051272
75	GO:0009881
76	GO:0002462
77	GO:0032510
78	GO:0001567
79	GO:0048598
80	GO:0034450
81	GO:0019064
82	GO:0004944
83	GO:0004252
84	GO:0046907
85	GO:0014043
86	GO:0043405
87	GO:0008209
88	GO:0003823
89	GO:0031954
90	GO:0017038
91	GO:0031109
92	GO:0006029
93	GO:0042461

94	GO:0008236
95	GO:0004653
96	GO:0010711
97	GO:0033299
98	GO:0030229
99	GO:0055087
100	GO:0090031
101	GO:0007181
102	GO:0047696
103	GO:0001315
104	GO:0001798
105	GO:0009583
106	GO:0043654
107	GO:0038062
108	GO:0050766
109	GO:0006741
110	GO:0051881
111	GO:0052724
112	GO:0043117
113	GO:0060052
114	GO:0015074
115	GO:0034405
116	GO:0045103
117	GO:0010815
118	GO:1900006
119	GO:0052723
120	GO:0033700
121	GO:0051224
122	GO:0060311
123	GO:0032364
124	GO:0072384
125	GO:0030247
126	GO:0000254
127	GO:0051898
128	GO:0005688
129	GO:0043331
130	GO:0071813
131	GO:0046548
132	GO:0000832
133	GO:0003273
134	GO:0032793
135	GO:0006705
136	GO:0032805

137	GO:0010501
138	GO:0006537
139	GO:0034103
140	GO:0005586
141	GO:0072573
142	GO:1900086
143	GO:0019836
144	GO:0030621
145	GO:0034021
146	GO:0043196
147	GO:0004903
148	GO:0014826
149	GO:0032467
150	GO:0047915
151	GO:0048549
152	GO:0042088
153	GO:0019799
154	GO:0035257
155	GO:0010991
156	GO:0002262
157	GO:0006657
158	GO:0010804
159	GO:0060313
160	GO:0002666
161	GO:0010923
162	GO:0032809
163	GO:0060706
164	GO:2000349
165	GO:0021858
166	GO:0032417
167	GO:0051651
168	GO:0042612
169	GO:0005172
170	GO:0007063
171	GO:0003069
172	GO:0005930
173	GO:0008440
174	GO:0010703
175	GO:0031714
176	GO:0005540
177	GO:0061043
178	GO:0047497
179	GO:0060220

180	GO:0051389
181	GO:0001917
182	GO:0030145
183	GO:0016603
184	GO:0010760
185	GO:0043536
186	GO:0070429
187	GO:0043570
188	GO:0034346
189	GO:0006168
190	GO:0045494
191	GO:0003948
192	GO:0046911
193	GO:0010747
194	GO:0070195
195	GO:0033145
196	GO:0034148
197	GO:0004864
198	GO:0006290
199	GO:0017186
200	GO:0051788
201	GO:0004175
202	GO:0021517
203	GO:0060068
204	GO:0070722
205	GO:0048666
206	GO:0035845
207	GO:0070433
208	GO:0051106
209	GO:0031575
210	GO:0004517
211	GO:0051635
212	GO:0006562
213	GO:0042159
214	GO:0072686
215	GO:0016422
216	GO:0045179
217	GO:0033627
218	GO:0008271
219	GO:0016254
220	GO:0030235
221	GO:0048242
222	GO:0008574

223	GO:0090037
224	GO:0006122
225	GO:0030704
226	GO:0060745
227	GO:0035924
228	GO:2001141
229	GO:0015920
230	GO:0051580
231	GO:0010738
232	GO:0009258
233	GO:0004012
234	GO:0018963
235	GO:0015432
236	GO:0003743
237	GO:0051856
238	GO:0051894
239	GO:0005579
240	GO:0055090
241	GO:0006749
242	GO:0015116
243	GO:0032855
244	GO:0008554
245	GO:0051271
246	GO:0019730
247	GO:0010625
248	GO:0048074
249	GO:0006707
250	GO:0043183
251	GO:0031593
252	GO:0051280
253	GO:0015126
254	GO:0009100
255	GO:0021762
256	GO:0004496
257	GO:0051028
258	GO:0051000
259	GO:0045263
260	GO:0021681
261	GO:0010466
262	GO:0003100
263	GO:0043277
264	GO:0044464
265	GO:0010902

266	GO:0030903
267	GO:0032996
268	GO:0072563
269	hsa04962
270	GO:0000506
271	GO:0032432
272	GO:0004489
273	GO:0008753
274	GO:0043498
275	GO:0006869
276	GO:0030346
277	GO:0003999
278	GO:0031134
279	GO:0010519
280	GO:0060319
281	GO:0070633
282	GO:0021885
283	GO:0008253
284	GO:0033602
285	GO:0000710
286	GO:0004301
287	GO:0050665
288	GO:0050884
289	GO:0001569
290	GO:0009597
291	GO:0034358
292	GO:0017176
293	GO:0010821
294	GO:0060129
295	GO:0010875
296	GO:0006108
297	GO:0005922
298	GO:0032757
299	GO:0071593
300	GO:0005010
301	GO:0045869
302	GO:0001919
303	GO:0070247
304	GO:0005537
305	GO:0019430
306	GO:0071253
307	GO:0060272
308	GO:0006351

309	GO:0070541
310	GO:0060750
311	GO:0042801
312	GO:0061153
313	GO:0004784
314	GO:0010369
315	GO:0019166
316	GO:0009409
317	GO:0001848
318	GO:0032302
319	GO:0007256
320	GO:0002055
321	GO:0070287
322	GO:0007606
323	GO:0042622
324	GO:0061338
325	GO:0033269
326	GO:0018894
327	GO:0001937
328	GO:0032368
329	GO:0007398
330	GO:0006576
331	GO:0002042
332	GO:0005548
333	GO:0004631
334	GO:0048306
335	GO:0042832
336	GO:0048878
337	GO:0043490
338	GO:0046813
339	GO:0034369
340	hsa03013
341	GO:0008397
342	GO:0045599
343	GO:0006801
344	GO:0030497
345	GO:0002891
346	GO:0033081
347	GO:0000406
348	GO:0005124
349	GO:0055094
350	GO:0070050
351	GO:0019825

352	GO:2001027
353	GO:1900142
354	GO:0010001
355	GO:0051782
356	GO:0048255
357	GO:0033778
358	GO:0017158
359	GO:0070530
360	GO:0009449
361	GO:0019835
362	GO:0004181
363	GO:0015301
364	GO:0070052
365	GO:2000504
366	GO:0034767
367	GO:0052033
368	GO:0015485
369	GO:0006940
370	GO:0045174
371	GO:1900165
372	GO:0005367
373	GO:0010900
374	GO:0007632
375	GO:0060128
376	GO:0045309
377	GO:0002523
378	GO:0016805
379	GO:0043381
380	GO:0008187
381	GO:0050610
382	GO:0000956
383	GO:0070430
384	GO:0010165
385	GO:0008272
386	GO:0060613
387	GO:0005581
388	GO:0033192
389	GO:0070326
390	GO:0051301
391	GO:0033005
392	GO:0050711
393	GO:0010763
394	GO:0032181

395	GO:0032003
396	GO:0061077
397	GO:0043142
398	GO:0050790
399	GO:0035092
400	GO:0001300
401	GO:0012501
402	GO:0033093
403	GO:0071028
404	GO:0035630
405	GO:0051148
406	GO:0002537
407	GO:0070328
408	GO:0001923
409	GO:0000267
410	GO:0031232
411	GO:0006491
412	GO:0048246
413	GO:0002028
414	GO:0005577
415	GO:0031573
416	GO:0005041
417	GO:0050655
418	GO:2000533
419	GO:0097186
420	GO:0047547
421	GO:0001938
422	GO:0016619
423	GO:0050262
424	GO:0015727
425	GO:0044241
426	GO:0003009
427	GO:0031798
428	GO:0031061
429	GO:0019439
430	GO:0001922
431	GO:0008094
432	GO:0060266
433	GO:0071062
434	GO:0015129
435	GO:0060229
436	GO:0019543
437	GO:0035873

438	GO:0016046
439	GO:0007567
440	GO:0010934
441	GO:0031077
442	GO:0003032
443	GO:0035417
444	GO:0006649
445	GO:0032449
446	GO:0004473
447	GO:0014909
448	GO:0051346
449	GO:0002282
450	GO:0010814
451	GO:0047408
452	GO:2000402
453	GO:0038052
454	GO:0050921
455	GO:0070506
456	GO:0009432
457	GO:0047409
458	GO:0003085
459	GO:0032958
460	GO:0031302
461	GO:0006311
462	GO:0030492
463	GO:0048672
464	GO:0030421
465	GO:0071922
466	GO:0032784
467	GO:0046485
468	GO:0009404
469	GO:0051918
470	GO:0070434
471	GO:0045159
472	GO:0030020
473	GO:0010816
474	GO:0003684
475	GO:0038091
476	GO:0010189
477	GO:0071222
478	GO:0061299
479	GO:0045076
480	GO:0019934

481	GO:0034959
482	GO:0042660
483	GO:0030331
484	GO:0051101
485	GO:0009624
486	GO:0008089
487	GO:0005687
488	GO:2000256
489	GO:0055099
490	GO:0015355
491	GO:0010232
492	GO:0002204
493	GO:0001540
494	GO:0050746
495	GO:0005072
496	GO:0046010
497	GO:0046784
498	GO:0021545
499	GO:0042345
500	GO:0016056

(9) Dataset D_9

a) MaxRel features list

Rank	Feature name
1	GO:0071482
2	GO:0038025
3	GO:0034436
4	GO:0034437
5	GO:0010001
6	GO:0003069
7	GO:0034021
8	GO:0001315
9	GO:0001970
10	GO:0048773
11	GO:0016495
12	GO:0034447
13	GO:0034189
14	GO:0004866
15	GO:0043537
16	GO:2001028
17	GO:0002575
18	GO:0048739
19	GO:0060342

20	hsa05150
21	GO:0038026
22	GO:0071953
23	hsa04610
24	GO:0043536
25	GO:0072563
26	GO:0050665
27	GO:0035645
28	GO:0038091
29	GO:0043183
30	GO:0048844
31	GO:0045103
32	GO:0051400
33	GO:0001938
34	GO:0004944
35	GO:0005172
36	GO:0032930
37	GO:0048678
38	GO:0010863
39	GO:0008228
40	GO:0051389
41	GO:0014043
42	GO:0003273
43	GO:0030449
44	GO:0006956
45	GO:0051856
46	GO:0034185
47	GO:0002042
48	GO:0001798
49	GO:1900086
50	GO:0032467
51	GO:0010759
52	GO:0060311
53	GO:0010711
54	GO:0051580
55	GO:0044464
56	GO:0060313
57	GO:0010703
58	GO:0010625
59	GO:0034103
60	GO:0048245
61	GO:0048845
62	GO:0051651

63	GO:0046911
64	GO:0032805
65	GO:0021762
66	GO:0060319
67	GO:0048593
68	GO:0045429
69	GO:0003032
70	GO:0001300
71	GO:0060754
72	GO:0043331
73	GO:0030229
74	GO:0004962
75	GO:0002282
76	GO:0000302
77	GO:0046813
78	GO:0015920
79	GO:0051881
80	GO:0090031
81	GO:0030949
82	GO:0004784
83	GO:1900006
84	GO:0006940
85	GO:0042159
86	GO:0032855
87	GO:0007603
88	GO:0000303
89	GO:0048246
90	GO:0005581
91	GO:0009881
92	GO:0034346
93	GO:0031109
94	GO:0009597
95	GO:0016056
96	GO:0060527
97	GO:0051898
98	GO:0035630
99	GO:0060011
100	GO:0010477
101	GO:0001910
102	GO:0002693
103	GO:0035924
104	GO:0002457
105	GO:0010466

106	GO:0043498
107	GO:0032757
108	GO:0033691
109	GO:0001917
110	GO:0043206
111	GO:0071361
112	GO:0032463
113	GO:0002551
114	GO:0052033
115	GO:0042583
116	GO:0042622
117	GO:0042554
118	GO:0006957
119	GO:0019064
120	GO:0032958
121	GO:0043171
122	GO:0060020
123	GO:0032364
124	GO:0032387
125	GO:0001968
126	GO:0001957
127	GO:0042056
128	GO:2000379
129	GO:0050918
130	GO:0008360
131	GO:0032809
132	GO:0001991
133	GO:0071346
134	GO:0033552
135	GO:0055090
136	GO:0035082
137	GO:0042461
138	GO:0005010
139	GO:0042660
140	GO:0043117
141	GO:0048018
142	GO:0002523
143	GO:0031012
144	GO:0051272
145	GO:0014904
146	GO:0009441
147	GO:0014896
148	GO:0051000

149	GO:0060509
150	GO:0017158
151	GO:0060510
152	GO:0005072
153	GO:0005534
154	GO:0071222
155	GO:0045766
156	GO:0060346
157	GO:0030214
158	GO:0070434
159	GO:0032732
160	GO:0002092
161	GO:0070430
162	GO:0016046
163	GO:0002537
164	GO:0070301
165	GO:0032432
166	GO:0005615
167	GO:0070670
168	GO:0021940
169	GO:0060052
170	GO:0010875
171	GO:0010595
172	GO:0009611
173	GO:0031701
174	GO:0004943
175	GO:0060228
176	GO:0002462
177	GO:0004876
178	GO:0001850
179	GO:0002291
180	GO:0006700
181	GO:0007497
182	GO:0010764
183	GO:0090190
184	GO:0019430
185	GO:0070371
186	GO:0060128
187	GO:0008090
188	GO:0060129
189	GO:0043200
190	GO:0033364
191	GO:0034363

192	GO:0060265
193	GO:0002232
194	GO:0060229
195	GO:0032490
196	GO:0045541
197	GO:0031077
198	GO:0043388
199	GO:0009409
200	GO:0031953
201	GO:0006958
202	GO:0010902
203	GO:0070050
204	GO:0043154
205	GO:0007256
206	GO:0042088
207	GO:0006801
208	GO:0060763
209	GO:0001937
210	GO:0030195
211	GO:0060394
212	GO:0050921
213	GO:0005586
214	GO:0001666
215	GO:0010763
216	GO:0002262
217	GO:0035767
218	GO:0060591
219	GO:0046620
220	GO:0048598
221	GO:0033160
222	GO:0002021
223	GO:0005506
224	GO:0060123
225	GO:0035684
226	GO:2000427
227	GO:0008395
228	GO:0002548
229	GO:2000502
230	GO:0006290
231	GO:0048661
232	GO:0001974
233	GO:0002218
234	GO:0031102

235	GO:0070483
236	GO:0070326
237	GO:0055093
238	GO:0001569
239	GO:0060042
240	GO:0001998
241	GO:0035411
242	GO:0031703
243	GO:0003331
244	GO:0001999
245	GO:0061049
246	GO:0014873
247	GO:0034104
248	GO:0048251
249	GO:0030516
250	GO:0060351
251	GO:0070886
252	GO:0032270
253	GO:0060744
254	GO:0033081
255	GO:0021517
256	GO:0070373
257	GO:0009583
258	GO:0010804
259	GO:0019836
260	GO:0070541
261	GO:0043654
262	GO:0033700
263	GO:0042310
264	GO:0009586
265	GO:0006917
266	GO:0051607
267	GO:0071356
268	GO:0071456
269	GO:0007568
270	GO:0048009
271	GO:0009408
272	GO:0042832
273	GO:0002384
274	GO:0002446
275	GO:0031017
276	GO:0070052
277	GO:0035624

278	GO:0010899
279	GO:0007250
280	GO:0010873
281	GO:0048144
282	GO:0071360
283	GO:0042095
284	GO:0050777
285	GO:0060729
286	GO:0051659
287	GO:0030336
288	GO:0048306
289	GO:0048146
290	GO:0071347
291	GO:0045765
292	GO:0032148
293	GO:0060325
294	GO:0051918
295	GO:0033627
296	GO:0002758
297	hsa05323
298	GO:0015643
299	GO:0070091
300	GO:0060745
301	GO:0060697
302	GO:0035845
303	GO:2000270
304	GO:0034358
305	GO:0002429
306	GO:0046629
307	GO:2000288
308	GO:0090037
309	GO:0043559
310	GO:2000121
311	GO:0061045
312	GO:0060426
313	GO:0046716
314	GO:0005548
315	GO:0061302
316	GO:0045359
317	GO:0008210
318	GO:0035458
319	GO:0001502
320	GO:0072593

321	GO:0000003
322	GO:0021858
323	GO:0034384
324	GO:0048635
325	GO:0031093
326	GO:0048010
327	GO:0032417
328	GO:0048286
329	GO:0008217
330	GO:0071230
331	GO:0034505
332	GO:0051591
333	GO:0016004
334	GO:0004252
335	GO:0005041
336	GO:0004012
337	GO:0002035
338	GO:0045188
339	GO:0021952
340	GO:0006935
341	GO:0060230
342	GO:0050930
343	GO:0071813
344	GO:0045906
345	GO:0032497
346	GO:0042325
347	GO:0051894
348	GO:0030346
349	GO:0043184
350	GO:0016942
351	GO:0001895
352	GO:0070506
353	GO:0035987
354	GO:0032368
355	GO:0004509
356	GO:0010900
357	GO:0031052
358	GO:0031507
359	GO:0003131
360	GO:0035978
361	GO:0034369
362	GO:0035500
363	GO:2000685

364	GO:0035501
365	GO:2001033
366	GO:0060613
367	GO:0000189
368	GO:0005883
369	GO:0090265
370	GO:0008584
371	GO:0046579
372	GO:0010544
373	GO:0043066
374	GO:0007263
375	GO:0048407
376	GO:0031727
377	hsa05133
378	GO:0005178
379	GO:0007565
380	GO:0007584
381	GO:0010574
382	GO:0051384
383	GO:0042060
384	GO:0051602
385	GO:0008209
386	GO:0038052
387	GO:0031798
388	GO:0060436
389	GO:0002033
390	GO:0060879
391	GO:0060496
392	GO:0046877
393	GO:0071253
394	GO:0070352
395	GO:0061115
396	GO:0060661
397	GO:0008107
398	GO:0001730
399	GO:0070384
400	GO:0070075
401	GO:0050674
402	GO:0050677
403	GO:0048535
404	GO:0051873
405	GO:0070051
406	GO:2000098

407	GO:0003104
408	GO:0006707
409	GO:0042231
410	GO:0010757
411	GO:2000256
412	GO:0060707
413	GO:0014012
414	GO:0010740
415	GO:0050650
416	GO:0032700
417	GO:0042253
418	GO:0042033
419	GO:0008089
420	GO:0014834
421	GO:0032878
422	GO:0090201
423	GO:0034056
424	GO:0061304
425	GO:0001867
426	GO:0008009
427	GO:0044241
428	GO:0005930
429	GO:0042627
430	GO:0016209
431	GO:0014826
432	GO:0048247
433	GO:0061044
434	GO:0030198
435	GO:0035491
436	GO:0071258
437	GO:0070887
438	GO:0016176
439	GO:2000591
440	GO:0045869
441	GO:0033299
442	GO:0022617
443	GO:0005201
444	GO:0072255
445	GO:0040007
446	GO:0072126
447	GO:0072264
448	GO:2000048
449	GO:0030023

450	GO:0007606
451	GO:0007435
452	GO:0043410
453	GO:0030155
454	GO:0003823
455	GO:0031091
456	GO:0034145
457	GO:0034382
458	GO:0001675
459	GO:0010951
460	GO:0048659
461	GO:0051387
462	GO:0097193
463	GO:0004301
464	GO:0008061
465	GO:0030235
466	GO:0044429
467	GO:0042307
468	hsa05143
469	GO:0010812
470	GO:0042092
471	GO:0048565
472	GO:0070542
473	GO:0042311
474	GO:0019900
475	GO:0032287
476	GO:0070022
477	GO:0060740
478	GO:0046677
479	GO:0033591
480	GO:0003009
481	GO:0060137
482	GO:0001955
483	GO:0050927
484	GO:0005577
485	GO:0070508
486	GO:0032488
487	GO:0071223
488	GO:0043570
489	GO:0010512
490	GO:0072332
491	GO:0034392
492	GO:0010716

493	GO:0031232
494	GO:0001540
495	GO:0060174
496	GO:0071506
497	GO:0051450
498	GO:0090263
499	GO:0006029
500	GO:0005576

b) mRMR features list

Rank	Feature name
1	GO:0071482
2	GO:0048739
3	GO:0055090
4	GO:0004866
5	GO:0038025
6	GO:0005581
7	GO:0010001
8	GO:0048018
9	GO:0003069
10	GO:0006956
11	GO:0070052
12	GO:0008228
13	GO:0038026
14	GO:0048593
15	hsa05150
16	GO:0000003
17	GO:0060229
18	GO:0001970
19	GO:0072563
20	GO:0031701
21	hsa04610
22	GO:0019064
23	GO:0034021
24	GO:0035082
25	GO:0038091
26	GO:0002429
27	GO:0030449
28	GO:0034436
29	GO:0005506
30	GO:0060342
31	GO:0031012
32	GO:0004944

33	GO:0043537
34	GO:0042461
35	GO:0032930
36	GO:0060265
37	GO:0007603
38	GO:0033691
39	GO:0051272
40	GO:0015920
41	GO:0008395
42	GO:0001315
43	GO:0002575
44	GO:0021858
45	GO:0001991
46	GO:0016495
47	GO:0072686
48	GO:0046629
49	GO:0060042
50	GO:0034437
51	GO:0006940
52	GO:0060744
53	GO:0002232
54	GO:0060123
55	GO:0010759
56	GO:0001957
57	GO:0034384
58	GO:0048773
59	GO:0006626
60	GO:2001028
61	GO:0006957
62	GO:0032490
63	GO:0048844
64	GO:0009881
65	GO:0071953
66	GO:0005597
67	GO:0042583
68	GO:0004509
69	GO:0034189
70	GO:0038063
71	GO:0043654
72	GO:0001998
73	GO:0001917
74	GO:1900086
75	GO:0060436

76	GO:0060011
77	GO:0001867
78	GO:0034447
79	GO:0060346
80	GO:0005883
81	GO:0001798
82	GO:0008107
83	GO:0002042
84	GO:0035411
85	GO:0035624
86	GO:0035645
87	GO:0001578
88	GO:0006700
89	GO:0035845
90	GO:0003032
91	GO:0006958
92	GO:0030214
93	GO:0031703
94	GO:0060879
95	GO:0043536
96	GO:0034185
97	GO:0001574
98	GO:0032364
99	GO:0048306
100	GO:0043171
101	GO:0003331
102	GO:0055106
103	GO:0050665
104	GO:0018184
105	GO:0002092
106	GO:2000270
107	GO:0031362
108	GO:0035924
109	GO:0004943
110	GO:0045103
111	GO:0001999
112	GO:0060496
113	GO:0046813
114	GO:0010501
115	GO:0048845
116	GO:0010804
117	GO:0061049
118	GO:0060754

119	GO:0001675
120	GO:0002462
121	GO:0060527
122	GO:0060806
123	GO:0010763
124	GO:0060228
125	GO:0060345
126	GO:0014873
127	GO:0016056
128	GO:0005930
129	GO:0032463
130	GO:0046877
131	GO:0004301
132	GO:0030621
133	GO:0043331
134	GO:0001502
135	GO:0031073
136	GO:0002523
137	GO:0060394
138	GO:0004876
139	GO:0035767
140	GO:0034104
141	GO:0004962
142	GO:0007080
143	GO:0008210
144	GO:0070352
145	GO:0051400
146	GO:0010902
147	GO:0005586
148	GO:0006705
149	GO:0090031
150	GO:0045046
151	GO:0070050
152	GO:0003823
153	GO:0061302
154	GO:0016715
155	GO:0005172
156	GO:0032994
157	GO:0014043
158	GO:0019799
159	GO:0001850
160	GO:0001730
161	GO:0042554

162	GO:0008200
163	GO:0051881
164	GO:0047749
165	GO:0061115
166	GO:0005577
167	GO:0043117
168	GO:0060052
169	GO:0010863
170	GO:0031491
171	GO:0034445
172	GO:0032417
173	GO:1900006
174	GO:0048535
175	GO:0043152
176	GO:0060661
177	GO:0043183
178	GO:0042612
179	GO:0019836
180	GO:0016557
181	GO:0048678
182	GO:0043206
183	GO:0016817
184	hsa04146
185	GO:0003273
186	GO:0031616
187	GO:0070384
188	GO:0010466
189	GO:0035375
190	GO:0004252
191	GO:0001750
192	GO:0043570
193	GO:0051873
194	GO:0042310
195	GO:0060319
196	GO:0035987
197	GO:0030229
198	GO:0006168
199	GO:0070075
200	GO:0005105
201	GO:0048246
202	GO:0044241
203	GO:0090190
204	GO:0006290

205	GO:0021678
206	GO:0005010
207	GO:0030023
208	GO:0002033
209	GO:0000302
210	GO:0070206
211	GO:0042622
212	GO:0050674
213	GO:0050689
214	GO:0090101
215	GO:0010388
216	GO:0043196
217	GO:0001938
218	GO:0070584
219	GO:0031052
220	GO:0051389
221	GO:0043159
222	GO:0006029
223	GO:0033700
224	GO:0051635
225	GO:0050677
226	GO:0071253
227	GO:0031091
228	GO:0021952
229	GO:0051856
230	GO:0051345
231	GO:0001968
232	GO:0002262
233	GO:0005055
234	GO:0031507
235	GO:0005537
236	GO:0051580
237	GO:0034875
238	GO:0060351
239	GO:0009597
240	GO:0010430
241	GO:0004016
242	GO:0033364
243	GO:0032855
244	GO:0003131
245	GO:0008187
246	GO:0005201
247	GO:0034363

248	GO:0005592
249	GO:0007606
250	GO:0044464
251	GO:0045618
252	GO:0005540
253	GO:2000379
254	GO:0014826
255	GO:0010873
256	GO:0048245
257	GO:0034088
258	GO:0071622
259	GO:0035978
260	GO:0071360
261	GO:0010747
262	GO:0097186
263	GO:0010625
264	GO:0016209
265	GO:0043088
266	GO:0031714
267	GO:0070506
268	GO:0032467
269	GO:0051148
270	GO:0070722
271	GO:0071779
272	GO:0035500
273	GO:0005813
274	GO:2000533
275	GO:0002134
276	GO:0004784
277	GO:0031077
278	GO:0048487
279	GO:0032958
280	GO:0060174
281	GO:2000685
282	GO:2000777
283	GO:0009583
284	GO:0036002
285	GO:0051898
286	GO:0010760
287	GO:0071361
288	GO:0051636
289	GO:0055009
290	GO:0004180

291	GO:0060020
292	GO:0050746
293	GO:1900165
294	GO:0060697
295	GO:0007183
296	GO:0035501
297	GO:2001141
298	GO:0034358
299	GO:0009586
300	GO:0009234
301	GO:0042447
302	GO:0043405
303	GO:0010934
304	GO:0007497
305	GO:2001033
306	GO:0060700
307	GO:0072593
308	GO:0015643
309	GO:0072332
310	GO:0032368
311	GO:0034122
312	GO:0005110
313	GO:0034041
314	GO:0002282
315	GO:0016705
316	GO:0008090
317	GO:0060613
318	GO:0001937
319	GO:0072606
320	GO:1900142
321	GO:0060311
322	GO:0090207
323	GO:0042359
324	GO:0043184
325	GO:0010900
326	GO:0038062
327	GO:0032226
328	GO:0000303
329	GO:0010872
330	GO:0033578
331	GO:0010711
332	GO:0061338
333	GO:0030497

334	GO:0004012
335	GO:0008747
336	GO:0010875
337	GO:0030247
338	GO:0042309
339	GO:0010595
340	GO:0016018
341	GO:0008817
342	GO:0060313
343	GO:0055087
344	GO:0002035
345	GO:0060510
346	GO:2001051
347	GO:0034369
348	GO:0000166
349	GO:0004657
350	GO:0050961
351	GO:0010703
352	GO:0004496
353	GO:0009720
354	GO:0070051
355	GO:0048343
356	GO:0042267
357	GO:0050907
358	GO:0043200
359	GO:0019262
360	GO:0034103
361	GO:0008202
362	GO:0008028
363	GO:0021509
364	GO:0002443
365	GO:0017158
366	GO:0045964
367	GO:0050777
368	GO:0006560
369	GO:0010815
370	GO:0042930
371	GO:0003828
372	GO:0060509
373	GO:0016922
374	GO:0060129
375	GO:0003051
376	GO:0000406

377	GO:0042931
378	GO:0048625
379	GO:0045239
380	GO:0015718
381	GO:0010133
382	GO:0034755
383	GO:0019166
384	GO:0060765
385	GO:0042660
386	hsa04974
387	GO:0050327
388	GO:0034466
389	GO:0005534
390	GO:0008061
391	GO:0046548
392	GO:0002820
393	GO:0008360
394	GO:0003104
395	GO:0030145
396	GO:0051084
397	GO:0008299
398	GO:0060325
399	GO:0048549
400	GO:0006869
401	GO:0035630
402	GO:0004333
403	GO:0050051
404	GO:0006576
405	GO:0050795
406	GO:0070541
407	GO:0043129
408	GO:0005041
409	GO:0021912
410	GO:0010764
411	GO:0046168
412	GO:0046333
413	GO:0021772
414	GO:0031102
415	GO:0001300
416	GO:0035473
417	GO:0032181
418	GO:0051387
419	GO:0033299

420	GO:0042157
421	GO:0071230
422	GO:0005762
423	GO:0052871
424	GO:0007289
425	GO:0006106
426	GO:0021913
427	GO:0070326
428	GO:0034145
429	GO:0015079
430	GO:0045429
431	GO:0016500
432	GO:0070576
433	GO:0035478
434	GO:0008428
435	GO:0009100
436	GO:0006741
437	GO:0045906
438	GO:0043295
439	GO:0061304
440	GO:0032302
441	GO:0097193
442	GO:0032809
443	GO:2001199
444	GO:0071503
445	GO:0030949
446	GO:0010917
447	GO:0019825
448	GO:0001786
449	GO:0090321
450	GO:0006801
451	GO:0090037
452	GO:0033145
453	GO:0019439
454	GO:0010972
455	GO:0021681
456	GO:0017038
457	GO:0050953
458	GO:0050918
459	GO:0060035
460	GO:0010498
461	GO:0051659
462	GO:0043178

463	GO:0019367
464	GO:0048144
465	GO:0030184
466	GO:0070670
467	GO:0070328
468	GO:0055010
469	GO:0030492
470	GO:0042371
471	GO:0048598
472	GO:0016332
473	GO:0002551
474	GO:0042627
475	GO:0001763
476	GO:0060666
477	GO:0043498
478	GO:0072061
479	GO:0004874
480	GO:0001844
481	GO:0005608
482	GO:0008334
483	GO:0030950
484	GO:0060128
485	GO:0004181
486	GO:0060482
487	GO:0055093
488	GO:0042737
489	GO:0070886
490	GO:0016556
491	GO:0040007
492	GO:0072060
493	GO:0007250
494	GO:0005104
495	GO:0010826
496	GO:0071241
497	GO:0033552
498	GO:0072236
499	GO:0048850
500	GO:0004598

(10) Dataset D_{10}

a) MaxRel features list

Rank	Feature name
1	GO:0030449

2	hsa05150
3	GO:0034189
4	GO:0038025
5	hsa04610
6	GO:0004866
7	GO:0001970
8	GO:1900006
9	GO:0006956
10	GO:0071953
11	GO:0031109
12	GO:0032432
13	GO:0043537
14	GO:0042554
15	GO:0034436
16	GO:0034437
17	GO:0010711
18	GO:0060311
19	GO:0034346
20	GO:0060313
21	GO:0010703
22	GO:0034103
23	GO:0006957
24	GO:0071482
25	GO:0006958
26	GO:0034447
27	GO:0035645
28	GO:0051651
29	GO:0046911
30	GO:0032805
31	GO:0051400
32	GO:0016495
33	GO:0043331
34	GO:0048844
35	GO:0051389
36	GO:0001798
37	GO:0021762
38	GO:0030949
39	GO:0038026
40	GO:0043536
41	GO:0034021
42	GO:0001315
43	GO:0003069
44	GO:0051000

45	GO:0050665
46	GO:0048773
47	GO:0045429
48	GO:0043183
49	GO:0042088
50	GO:0032757
51	GO:0060342
52	GO:0032467
53	GO:0032722
54	GO:0051856
55	GO:0043206
56	GO:0009409
57	GO:0060011
58	GO:0003273
59	GO:0045541
60	GO:0014043
61	GO:0002282
62	GO:0005615
63	GO:0043498
64	GO:0045766
65	GO:0001666
66	GO:0004962
67	GO:0043117
68	GO:0002042
69	GO:0032958
70	GO:0010477
71	GO:0070434
72	GO:0001910
73	GO:0001938
74	GO:0070430
75	GO:0004784
76	GO:0016046
77	GO:0032732
78	GO:0002693
79	GO:0002457
80	GO:0004252
81	GO:0032930
82	GO:0030195
83	GO:0042253
84	GO:2000256
85	GO:0042033
86	GO:0009597
87	GO:0042231

88	GO:0007256
89	GO:0045359
90	GO:1900086
91	GO:0005172
92	GO:0042660
93	GO:0042116
94	GO:0001300
95	GO:0002537
96	GO:0035458
97	GO:0034405
98	GO:0001974
99	GO:0032855
100	GO:0045103
101	GO:0000303
102	GO:0010575
103	GO:0034185
104	GO:0052033
105	GO:0046813
106	GO:0010001
107	GO:0072332
108	GO:0043154
109	GO:0010863
110	GO:0042159
111	GO:0002575
112	GO:0006801
113	GO:0051580
114	GO:0044464
115	GO:0010625
116	GO:0006917
117	GO:0005520
118	GO:0032488
119	GO:0005041
120	GO:0071360
121	GO:0001569
122	GO:0033627
123	GO:0048678
124	GO:0031798
125	GO:0038052
126	GO:0010466
127	GO:0060745
128	GO:0060754
129	GO:2001028
130	GO:0002291

131	GO:0008228
132	GO:0055093
133	GO:0007252
134	GO:0010760
135	GO:0031714
136	GO:0002446
137	GO:0034056
138	GO:0008209
139	GO:0036020
140	GO:0002523
141	GO:0005586
142	GO:0004944
143	GO:0045356
144	GO:0033364
145	GO:0033081
146	GO:0000302
147	GO:0019934
148	GO:0060319
149	GO:0002384
150	GO:0005072
151	GO:0007568
152	hsa05323
153	GO:0045671
154	GO:0090031
155	GO:0071222
156	GO:0048845
157	GO:0005010
158	GO:0032497
159	GO:0043200
160	GO:0050777
161	GO:0042095
162	GO:0048739
163	GO:0045343
164	hsa05133
165	GO:0050829
166	GO:0035924
167	GO:0071359
168	GO:0048661
169	GO:0030229
170	GO:0014896
171	GO:0014904
172	GO:0009441
173	GO:0060020

174	GO:0000189
175	GO:0031701
176	GO:0010875
177	GO:0048246
178	GO:0008201
179	GO:0042056
180	GO:0061045
181	GO:0070022
182	GO:0001957
183	GO:0008217
184	GO:0019430
185	GO:0060346
186	GO:0010764
187	GO:0060129
188	GO:0060228
189	GO:0051881
190	GO:0051272
191	GO:0070091
192	GO:0019966
193	GO:0002551
194	GO:0060312
195	GO:0048245
196	GO:0014826
197	GO:0045084
198	GO:0019836
199	GO:0008360
200	GO:0060128
201	GO:2000379
202	GO:0051142
203	GO:0030516
204	GO:0060750
205	GO:0031017
206	GO:0032809
207	GO:0032270
208	GO:0005534
209	GO:0043325
210	GO:0046620
211	GO:0001968
212	GO:0034363
213	GO:0005114
214	GO:0071312
215	GO:0070301
216	GO:2000378

217	GO:0048598
218	GO:0021940
219	GO:0060052
220	GO:0002262
221	GO:0060744
222	GO:0072563
223	GO:0019064
224	GO:0016209
225	GO:0007250
226	GO:0010873
227	GO:0002429
228	GO:0055090
229	GO:0034358
230	GO:0046629
231	GO:0008395
232	GO:0051607
233	GO:0034616
234	GO:0010544
235	GO:0007183
236	GO:0035624
237	GO:0051873
238	GO:0006707
239	GO:0030308
240	GO:0043559
241	GO:0009881
242	GO:0060068
243	GO:0007263
244	GO:0007497
245	GO:0090037
246	GO:0038091
247	GO:0046716
248	GO:0006940
249	GO:0051926
250	GO:0030346
251	GO:0060527
252	GO:0021517
253	GO:0051898
254	GO:0060510
255	GO:0060644
256	GO:0019049
257	GO:0070373
258	GO:0001781
259	GO:0030284

260	GO:0030214
261	GO:0032700
262	GO:0070541
263	GO:0002218
264	GO:0014909
265	GO:0042094
266	GO:0060090
267	GO:0002504
268	GO:0090265
269	GO:0007584
270	GO:0005576
271	GO:2000502
272	GO:0035684
273	GO:0061302
274	GO:0031727
275	GO:0042583
276	GO:0045630
277	GO:0033552
278	GO:0043171
279	GO:0071456
280	GO:0070483
281	GO:0071356
282	GO:0071346
283	GO:0035630
284	GO:0051712
285	GO:0060509
286	GO:0009408
287	GO:0035767
288	GO:0048009
289	GO:0071813
290	GO:0003032
291	GO:0008090
292	GO:0060561
293	GO:0006290
294	GO:0004943
295	GO:0004876
296	GO:0060364
297	GO:0001850
298	GO:0002462
299	GO:0022617
300	GO:0035666
301	GO:0034138
302	GO:0002756

303	hsa04623
304	GO:0006898
305	GO:0016942
306	GO:0051280
307	GO:0060591
308	GO:0016004
309	GO:0042060
310	GO:0071347
311	GO:0016056
312	GO:0051918
313	GO:0010804
314	GO:0001763
315	GO:0051054
316	GO:0001540
317	GO:0001934
318	GO:0045188
319	GO:0045079
320	GO:0045076
321	GO:0051092
322	GO:0005579
323	GO:0001869
324	GO:0010897
325	GO:2000270
326	GO:0032481
327	GO:0032269
328	GO:0060729
329	GO:0002021
330	GO:0085029
331	GO:0032387
332	GO:0010288
333	GO:0031953
334	GO:2000121
335	GO:0060137
336	GO:2000427
337	GO:0071503
338	GO:0017038
339	GO:0035478
340	GO:0000003
341	GO:0090321
342	GO:0009590
343	GO:0001567
344	GO:0035473
345	GO:0052871

346	GO:0070287
347	hsa05164
348	GO:0051795
349	GO:0010574
350	GO:0002032
351	GO:0060394
352	GO:0030168
353	GO:0031575
354	GO:0071230
355	GO:0009586
356	GO:0007565
357	GO:0032691
358	GO:0060545
359	GO:0004301
360	GO:0034382
361	GO:0043405
362	GO:0009615
363	GO:0070926
364	GO:0031077
365	GO:0050689
366	GO:0045869
367	GO:0015920
368	GO:0005105
369	GO:0033700
370	GO:0060763
371	GO:0042832
372	GO:0050927
373	GO:0051602
374	GO:0051098
375	GO:0009314
376	GO:0030828
377	GO:0048386
378	GO:0032364
379	GO:0032707
380	GO:0046677
381	GO:0008089
382	GO:0048864
383	GO:0050921
384	GO:0051894
385	GO:0045423
386	GO:0010987
387	GO:0038063
388	GO:0019826

389	GO:0045414
390	GO:0017162
391	GO:0048635
392	GO:0010595
393	GO:0009612
394	GO:0005585
395	GO:0042310
396	GO:0030335
397	GO:0032355
398	GO:0007184
399	GO:0001875
400	GO:0031702
401	GO:0032494
402	GO:0033141
403	GO:0006979
404	GO:0014911
405	GO:0051659
406	hsa05143
407	GO:0071361
408	GO:0042307
409	GO:1900165
410	GO:0048520
411	GO:2000533
412	GO:0010934
413	GO:0002033
414	GO:0010189
415	GO:1900142
416	hsa04145
417	GO:0010718
418	GO:0032417
419	GO:0032909
420	GO:0032720
421	GO:0031093
422	GO:0008083
423	GO:0030247
424	GO:0002758
425	GO:0015643
426	GO:0070326
427	GO:0042993
428	GO:0008285
429	GO:0050790
430	GO:0071391
431	GO:0060087

432	GO:0035082
433	GO:0042627
434	GO:0042954
435	GO:0060123
436	GO:0001596
437	GO:0045358
438	GO:0001867
439	GO:0042613
440	GO:0060697
441	GO:0010519
442	GO:0032430
443	GO:0051345
444	GO:0033299
445	GO:0048549
446	GO:0009986
447	GO:0051005
448	GO:0017015
449	GO:0007181
450	GO:0043065
451	hsa05144
452	GO:0043410
453	GO:0031663
454	GO:0001502
455	GO:0006029
456	GO:0090190
457	GO:0006916
458	GO:0007021
459	GO:0042637
460	GO:0060220
461	hsa04621
462	GO:0034134
463	GO:0002224
464	GO:0032728
465	GO:0030704
466	GO:0010902
467	GO:0007179
468	GO:0048074
469	GO:0034142
470	GO:0034130
471	GO:0044429
472	GO:0044146
473	GO:2000309
474	GO:0060552

475	GO:0060550
476	GO:0071351
477	GO:0045785
478	GO:0002020
479	GO:0043615
480	GO:0045725
481	GO:0001937
482	GO:0045740
483	GO:0045768
484	GO:0009583
485	GO:0051087
486	GO:0008354
487	GO:0010224
488	GO:0032878
489	GO:0010888
490	GO:0071364
491	GO:0010763
492	GO:0014834
493	GO:0009817
494	GO:0070371
495	GO:0033591
496	GO:0010942
497	GO:0046328
498	GO:0014910
499	GO:0045348
500	GO:1900126

b) mRMR features list

Rank	Feature name
1	GO:0030449
2	GO:0045343
3	hsa05150
4	GO:0034189
5	GO:0006956
6	GO:0043537
7	hsa04610
8	GO:0042554
9	GO:0008395
10	GO:0071482
11	GO:0002429
12	GO:0004866
13	GO:1900006
14	GO:0048739

15	GO:0001970
16	GO:0000003
17	GO:0038025
18	GO:0060744
19	GO:0071953
20	GO:0006958
21	GO:0031701
22	GO:0006957
23	GO:0002446
24	GO:0031109
25	GO:0060364
26	GO:0038063
27	GO:0046629
28	GO:0032432
29	GO:0051873
30	GO:1900086
31	GO:0060011
32	GO:0034436
33	GO:0002504
34	GO:0005586
35	GO:0035645
36	GO:0072332
37	GO:0005041
38	GO:0034021
39	GO:0004252
40	GO:0007183
41	GO:0006438
42	GO:0034346
43	GO:0008228
44	GO:0060090
45	GO:0043206
46	GO:0031575
47	GO:0051400
48	GO:0071503
49	GO:0004832
50	GO:0001798
51	GO:0034437
52	GO:0051272
53	GO:0050777
54	GO:0035624
55	GO:0043331
56	GO:0048844
57	GO:0035417

58	GO:0043117
59	GO:0017038
60	GO:0032930
61	GO:0034616
62	GO:0010711
63	GO:0005105
64	GO:0050665
65	GO:0042613
66	GO:0034447
67	GO:0001867
68	GO:0030949
69	GO:0045423
70	GO:0042490
71	GO:0035478
72	GO:0060311
73	GO:0008119
74	GO:0038026
75	GO:0010760
76	GO:0001315
77	GO:0001763
78	GO:0019064
79	GO:0035082
80	GO:0060313
81	GO:0031201
82	GO:0060342
83	GO:0090321
84	GO:0001974
85	GO:0031714
86	GO:0051389
87	GO:0019882
88	GO:2000270
89	GO:0010703
90	GO:0043405
91	GO:0034358
92	GO:0002042
93	GO:0032691
94	GO:0042583
95	GO:0032722
96	GO:0035473
97	GO:0005597
98	GO:0034103
99	GO:0034405
100	GO:0004301

101	GO:0060052
102	GO:0031362
103	GO:0051651
104	GO:0007184
105	GO:0003069
106	GO:0004944
107	GO:0005540
108	GO:0004962
109	GO:0005537
110	GO:0043183
111	GO:0070287
112	GO:0010575
113	GO:0006705
114	GO:0016209
115	GO:0045414
116	GO:0006917
117	GO:0043536
118	GO:0032226
119	GO:0046911
120	GO:0031491
121	GO:0050689
122	GO:0004943
123	GO:0048773
124	GO:1900165
125	GO:0009409
126	GO:0032395
127	GO:0042116
128	GO:0060123
129	GO:0010001
130	GO:2000533
131	GO:0051856
132	GO:0033364
133	GO:0014909
134	GO:0055087
135	GO:0016495
136	GO:0051635
137	GO:0007263
138	GO:0042147
139	GO:0017162
140	GO:0010369
141	GO:0032805
142	GO:0007256
143	GO:0010934

144	GO:2001028
145	GO:0060068
146	GO:0004876
147	GO:0045076
148	GO:0055090
149	GO:0014043
150	GO:0042461
151	GO:0032467
152	GO:0006029
153	GO:0035666
154	GO:0016409
155	GO:0004784
156	GO:1900142
157	GO:0071360
158	GO:0034185
159	GO:0002033
160	GO:0035924
161	GO:0002032
162	GO:0045429
163	GO:0034365
164	GO:0010501
165	GO:0005114
166	GO:0060750
167	GO:0001567
168	GO:0032876
169	GO:0001850
170	GO:0008209
171	GO:0006801
172	GO:0038062
173	GO:0055093
174	GO:0008187
175	GO:0009597
176	GO:0016554
177	GO:0032757
178	GO:0042310
179	GO:0060754
180	GO:0002523
181	GO:0005010
182	GO:0030621
183	GO:0043171
184	GO:0034445
185	GO:0021762
186	GO:0019966

187	GO:0007603
188	GO:0005488
189	GO:0051000
190	GO:0048549
191	GO:0055106
192	GO:0035458
193	GO:0019826
194	GO:0031616
195	GO:0002462
196	GO:0050790
197	GO:0007252
198	GO:0010873
199	GO:0005520
200	GO:0010987
201	GO:0043498
202	GO:0003746
203	GO:0051148
204	GO:0034056
205	GO:0034138
206	GO:0017176
207	GO:0009881
208	GO:0035767
209	GO:0005579
210	GO:0051098
211	GO:0061302
212	GO:0006707
213	GO:0019799
214	GO:0001666
215	GO:0045953
216	GO:0006944
217	GO:0042088
218	GO:0004085
219	GO:0090031
220	GO:0052871
221	GO:0004509
222	GO:0030195
223	GO:0051881
224	GO:0010466
225	GO:0046359
226	GO:0019836
227	GO:0038091
228	GO:0010804
229	GO:0047006

230	GO:0002756
231	GO:0032417
232	GO:0060745
233	GO:0042393
234	GO:0046813
235	GO:0043196
236	GO:0010875
237	GO:0031702
238	GO:0001968
239	GO:0032855
240	GO:0004016
241	GO:0014826
242	GO:0005172
243	GO:0016922
244	GO:0010897
245	GO:2000095
246	GO:0007062
247	GO:0085029
248	GO:0034122
249	GO:0045541
250	GO:0019835
251	GO:0009590
252	GO:0010821
253	GO:0005615
254	GO:0032481
255	GO:0001087
256	GO:0030247
257	GO:0033781
258	GO:0000303
259	GO:0033116
260	GO:0010522
261	GO:0050711
262	GO:0042253
263	GO:0010988
264	GO:0090037
265	GO:0004441
266	GO:0032488
267	GO:0001093
268	GO:0032958
269	GO:0006940
270	GO:0021858
271	GO:0090200
272	GO:0060346

273	GO:0070584
274	GO:0033299
275	GO:0035501
276	GO:0001596
277	GO:0045766
278	GO:0001578
279	GO:0097241
280	GO:0051280
281	GO:2000256
282	GO:0070384
283	GO:0032938
284	GO:0001869
285	GO:0010863
286	GO:0016422
287	GO:0034755
288	GO:0045359
289	GO:0070206
290	GO:0006290
291	GO:0006433
292	GO:0006741
293	GO:0045103
294	GO:0042406
295	GO:0007250
296	GO:0060220
297	GO:0010477
298	GO:0001011
299	GO:0032364
300	GO:0042033
301	GO:0046877
302	GO:0032430
303	GO:0006168
304	GO:0006898
305	GO:0002575
306	GO:0010718
307	GO:0002282
308	GO:0033961
309	GO:0004827
310	GO:0035987
311	GO:0008217
312	GO:0003032
313	GO:0010189
314	GO:2000379
315	GO:0071248

316	GO:0003273
317	GO:0060228
318	GO:0019207
319	GO:0042231
320	GO:0048243
321	GO:0005585
322	GO:0033081
323	GO:2000723
324	GO:0016056
325	GO:0050674
326	GO:0008390
327	GO:0070524
328	GO:0048598
329	GO:0043570
330	GO:0001910
331	GO:0002001
332	GO:0001957
333	GO:0070541
334	GO:0045607
335	GO:0008200
336	GO:0050927
337	GO:0031507
338	GO:0032510
339	GO:0030127
340	GO:0070434
341	GO:0060948
342	GO:0090207
343	GO:0036020
344	GO:0019067
345	GO:0030308
346	GO:0060319
347	GO:0017112
348	GO:0060436
349	GO:0002693
350	GO:0010288
351	GO:0035545
352	GO:0032786
353	GO:0043559
354	GO:0045084
355	GO:0035730
356	GO:0019377
357	GO:0019439
358	GO:0043325

359	GO:0030704
360	GO:0001938
361	GO:0016272
362	GO:0035978
363	GO:0010917
364	GO:0033145
365	GO:0001300
366	GO:0070430
367	GO:0060229
368	GO:0050677
369	GO:0051918
370	GO:0008456
371	GO:0042660
372	GO:0001919
373	GO:0042954
374	GO:0045356
375	GO:0070664
376	GO:0046620
377	GO:0060213
378	GO:0032463
379	GO:0045309
380	GO:0031077
381	hsa04623
382	GO:0003743
383	GO:0002457
384	GO:0042627
385	GO:0035500
386	GO:0060047
387	GO:0031134
388	GO:0021772
389	GO:0016046
390	GO:0010498
391	GO:0030284
392	GO:0061115
393	GO:0034237
394	GO:0048074
395	GO:0071359
396	GO:0001991
397	GO:0035732
398	GO:0061045
399	GO:0045820
400	GO:0048242
401	GO:0010519

402	GO:0072686
403	GO:0032732
404	GO:0071779
405	GO:0060129
406	GO:0019262
407	GO:0042637
408	GO:0051092
409	GO:0034220
410	GO:0031052
411	GO:0060697
412	GO:2000777
413	GO:0009586
414	hsa05133
415	GO:0030229
416	GO:0060496
417	GO:0019673
418	GO:2000820
419	GO:0070026
420	GO:0060394
421	GO:0001844
422	GO:0002262
423	GO:0007497
424	GO:0005930
425	GO:0008201
426	GO:0010544
427	GO:0008747
428	GO:0009249
429	GO:0033627
430	GO:0002035
431	GO:0019430
432	GO:0060661
433	GO:0060545
434	GO:0019049
435	GO:0003131
436	GO:0010826
437	GO:0060137
438	GO:0006700
439	GO:0006508
440	GO:0042335
441	GO:0010900
442	GO:0048025
443	GO:0097057
444	GO:0052033

445	GO:0051345
446	GO:0031798
447	GO:0070352
448	GO:0016139
449	GO:0019509
450	GO:0009615
451	GO:0050840
452	GO:2001033
453	GO:2000378
454	GO:0004169
455	GO:0033159
456	GO:0032368
457	GO:0030346
458	GO:0030214
459	GO:0070926
460	GO:0035731
461	GO:0003824
462	GO:0071813
463	GO:0090230
464	GO:0070022
465	GO:0043576
466	GO:0016192
467	GO:0010165
468	GO:0070075
469	GO:0001569
470	GO:0046703
471	GO:0000302
472	GO:0034369
473	GO:0045056
474	GO:0022617
475	GO:0043422
476	GO:0010700
477	GO:0071208
478	GO:0002537
479	GO:0060510
480	GO:0051101
481	GO:2000685
482	GO:0003918
483	GO:0038052
484	GO:0060879
485	GO:0051271
486	GO:0034363
487	GO:0060128

488	GO:0032020
489	GO:0007584
490	GO:0016556
491	GO:0048678
492	GO:0035092
493	GO:0004473
494	GO:0072563
495	GO:2000504
496	GO:0034134
497	GO:0005602
498	hsa04145
499	GO:0072163
500	GO:0033093
