# Supplementary material for: Gene Ontology and KEGG Enrichment Analyses of Genes Related to Age-Related Macular Degeneration
Source: Biomed Res Int. 2014 Aug 6;2014:450386. doi: 10.1155/2014/450386 (PMC4140130; doi:10.1155/2014/450386)
Supplement: Supplementary file 1 — The Supplementary Material contains five files. In detail, Supplementary Material I lists 39 known AMD related genes and 1,950 randomly selected genes; Supplementary Material II lists the output of mRMR program on each dataset; Supplementary Material III lists the accuracies obtained by IFS and SMO on each dataset; Supplementary Material IV lists the IFS curve on each dataset; Supplementary Material V lists the features in the final optimal feature set. [file 450386.f1.zip › Supp-III.pdf]

Supplementary Material III. The SNs, SPs, ACCs and MCCs obtained by IFS and SMO for each dataset D_i.

(1) Dataset D_1

Number of features	SN	SP	ACC	MCC
4	0.461538	0.989744	0.901709	0.601556
5	0.461538	0.989744	0.901709	0.601556
6	0.435897	0.994872	0.901709	0.602464
7	0.410256	0.984615	0.888889	0.538778
8	0.410256	0.994872	0.897436	0.581685
9	0.461538	0.989744	0.901709	0.601556
10	0.512821	0.989744	0.910256	0.641744
11	0.461538	0.989744	0.901709	0.601556
12	0.487179	0.984615	0.901709	0.602454
13	0.538462	0.979487	0.905983	0.624875
14	0.487179	0.969231	0.888889	0.550633
15	0.487179	0.969231	0.888889	0.550633
16	0.512821	0.969231	0.893162	0.571643
17	0.538462	0.964103	0.893162	0.57707
18	0.538462	0.958974	0.888889	0.562614
19	0.564103	0.953846	0.888889	0.569387
20	0.589744	0.958974	0.897436	0.603212
21	0.589744	0.953846	0.893162	0.589617
22	0.564103	0.948718	0.884615	0.556243
23	0.589744	0.948718	0.888889	0.576566
24	0.589744	0.953846	0.893162	0.589617
25	0.564103	0.953846	0.888889	0.569387
26	0.564103	0.958974	0.893162	0.583095
27	0.564103	0.958974	0.893162	0.583095
28	0.564103	0.964103	0.897436	0.597415
29	0.564103	0.958974	0.893162	0.583095
30	0.564103	0.958974	0.893162	0.583095
31	0.564103	0.974359	0.905983	0.628112
32	0.564103	0.974359	0.905983	0.628112
33	0.564103	0.974359	0.905983	0.628112
34	0.564103	0.974359	0.905983	0.628112
35	0.564103	0.974359	0.905983	0.628112
36	0.564103	0.974359	0.905983	0.628112
37	0.564103	0.974359	0.905983	0.628112
38	0.564103	0.969231	0.901709	0.612401
39	0.564103	0.969231	0.901709	0.612401
40	0.564103	0.974359	0.905983	0.628112

41	0.564103	0.974359	0.905983	0.628112
42	0.589744	0.979487	0.91453	0.664004
43	0.589744	0.979487	0.91453	0.664004
44	0.589744	0.979487	0.91453	0.664004
45	0.589744	0.979487	0.91453	0.664004
46	0.589744	0.979487	0.91453	0.664004
47	0.589744	0.979487	0.91453	0.664004
48	0.589744	0.979487	0.91453	0.664004
49	0.589744	0.979487	0.91453	0.664004
50	0.589744	0.969231	0.905983	0.632216
51	0.589744	0.974359	0.910256	0.647732
52	0.589744	0.969231	0.905983	0.632216
53	0.589744	0.974359	0.910256	0.647732
54	0.589744	0.969231	0.905983	0.632216
55	0.589744	0.969231	0.905983	0.632216
56	0.564103	0.969231	0.901709	0.612401
57	0.564103	0.974359	0.905983	0.628112
58	0.564103	0.969231	0.901709	0.612401
59	0.589744	0.969231	0.905983	0.632216
60	0.564103	0.969231	0.901709	0.612401
61	0.564103	0.969231	0.901709	0.612401
62	0.564103	0.974359	0.905983	0.628112
63	0.564103	0.969231	0.901709	0.612401
64	0.589744	0.964103	0.901709	0.617395
65	0.589744	0.964103	0.901709	0.617395
66	0.589744	0.964103	0.901709	0.617395
67	0.589744	0.964103	0.901709	0.617395
68	0.589744	0.964103	0.901709	0.617395
69	0.589744	0.958974	0.897436	0.603212
70	0.589744	0.953846	0.893162	0.589617
71	0.589744	0.953846	0.893162	0.589617
72	0.589744	0.953846	0.893162	0.589617
73	0.589744	0.953846	0.893162	0.589617
74	0.589744	0.958974	0.897436	0.603212
75	0.589744	0.953846	0.893162	0.589617
76	0.589744	0.953846	0.893162	0.589617
77	0.615385	0.953846	0.897436	0.609513
78	0.615385	0.953846	0.897436	0.609513
79	0.589744	0.953846	0.893162	0.589617
80	0.615385	0.948718	0.893162	0.596559
81	0.589744	0.948718	0.888889	0.576566
82	0.564103	0.948718	0.884615	0.556243
83	0.564103	0.953846	0.888889	0.569387

84	0.589744	0.958974	0.897436	0.603212
85	0.589744	0.953846	0.893162	0.589617
86	0.589744	0.948718	0.888889	0.576566
87	0.589744	0.948718	0.888889	0.576566
88	0.615385	0.948718	0.893162	0.596559
89	0.589744	0.948718	0.888889	0.576566
90	0.589744	0.948718	0.888889	0.576566
91	0.564103	0.948718	0.884615	0.556243
92	0.564103	0.948718	0.884615	0.556243
93	0.564103	0.948718	0.884615	0.556243
94	0.564103	0.94359	0.880342	0.54362
95	0.564103	0.94359	0.880342	0.54362
96	0.589744	0.953846	0.893162	0.589617
97	0.589744	0.953846	0.893162	0.589617
98	0.589744	0.948718	0.888889	0.576566
99	0.589744	0.953846	0.893162	0.589617
100	0.589744	0.953846	0.893162	0.589617
101	0.589744	0.953846	0.893162	0.589617
102	0.589744	0.953846	0.893162	0.589617
103	0.641026	0.964103	0.910256	0.656366
104	0.641026	0.964103	0.910256	0.656366
105	0.641026	0.964103	0.910256	0.656366
106	0.641026	0.964103	0.910256	0.656366
107	0.615385	0.964103	0.905983	0.637037
108	0.615385	0.964103	0.905983	0.637037
109	0.615385	0.964103	0.905983	0.637037
110	0.615385	0.964103	0.905983	0.637037
111	0.615385	0.964103	0.905983	0.637037
112	0.615385	0.964103	0.905983	0.637037
113	0.615385	0.964103	0.905983	0.637037
114	0.615385	0.969231	0.910256	0.651695
115	0.615385	0.964103	0.905983	0.637037
116	0.615385	0.964103	0.905983	0.637037
117	0.615385	0.969231	0.910256	0.651695
118	0.615385	0.969231	0.910256	0.651695
119	0.615385	0.969231	0.910256	0.651695
120	0.615385	0.969231	0.910256	0.651695
121	0.615385	0.969231	0.910256	0.651695
122	0.615385	0.969231	0.910256	0.651695
123	0.615385	0.969231	0.910256	0.651695
124	0.615385	0.969231	0.910256	0.651695
125	0.615385	0.958974	0.901709	0.622992
126	0.615385	0.958974	0.901709	0.622992

127	0.615385	0.958974	0.901709	0.622992
128	0.615385	0.958974	0.901709	0.622992
129	0.615385	0.958974	0.901709	0.622992
130	0.615385	0.958974	0.901709	0.622992
131	0.641026	0.958974	0.905983	0.64246
132	0.641026	0.964103	0.910256	0.656366
133	0.615385	0.958974	0.901709	0.622992
134	0.641026	0.953846	0.901709	0.629098
135	0.641026	0.953846	0.901709	0.629098
136	0.666667	0.969231	0.918803	0.689741
137	0.615385	0.969231	0.910256	0.651695
138	0.615385	0.964103	0.905983	0.637037
139	0.692308	0.964103	0.918803	0.694177
140	0.692308	0.964103	0.918803	0.694177
141	0.641026	0.964103	0.910256	0.656366
142	0.641026	0.964103	0.910256	0.656366
143	0.641026	0.964103	0.910256	0.656366
144	0.641026	0.958974	0.905983	0.64246
145	0.641026	0.958974	0.905983	0.64246
146	0.615385	0.958974	0.901709	0.622992
147	0.615385	0.958974	0.901709	0.622992
148	0.641026	0.958974	0.905983	0.64246
149	0.641026	0.964103	0.910256	0.656366
150	0.641026	0.953846	0.901709	0.629098
151	0.641026	0.958974	0.905983	0.64246
152	0.641026	0.964103	0.910256	0.656366
153	0.641026	0.958974	0.905983	0.64246
154	0.641026	0.958974	0.905983	0.64246
155	0.641026	0.958974	0.905983	0.64246
156	0.641026	0.958974	0.905983	0.64246
157	0.641026	0.953846	0.901709	0.629098
158	0.641026	0.958974	0.905983	0.64246
159	0.641026	0.958974	0.905983	0.64246
160	0.641026	0.953846	0.901709	0.629098
161	0.615385	0.953846	0.897436	0.609513
162	0.615385	0.953846	0.897436	0.609513
163	0.615385	0.958974	0.901709	0.622992
164	0.615385	0.958974	0.901709	0.622992
165	0.615385	0.958974	0.901709	0.622992
166	0.589744	0.964103	0.901709	0.617395
167	0.589744	0.964103	0.901709	0.617395
168	0.589744	0.964103	0.901709	0.617395
169	0.589744	0.958974	0.897436	0.603212

170	0.615385	0.964103	0.905983	0.637037
171	0.589744	0.964103	0.901709	0.617395
172	0.589744	0.969231	0.905983	0.632216
173	0.589744	0.958974	0.897436	0.603212
174	0.615385	0.953846	0.897436	0.609513
175	0.615385	0.953846	0.897436	0.609513
176	0.615385	0.953846	0.897436	0.609513
177	0.615385	0.953846	0.897436	0.609513
178	0.589744	0.948718	0.888889	0.576566
179	0.641026	0.948718	0.897436	0.616243
180	0.615385	0.948718	0.893162	0.596559
181	0.641026	0.948718	0.897436	0.616243
182	0.641026	0.948718	0.897436	0.616243
183	0.641026	0.948718	0.897436	0.616243
184	0.666667	0.948718	0.901709	0.635642
185	0.666667	0.948718	0.901709	0.635642
186	0.641026	0.94359	0.893162	0.60386
187	0.666667	0.94359	0.897436	0.623344
188	0.641026	0.94359	0.893162	0.60386
189	0.641026	0.94359	0.893162	0.60386
190	0.666667	0.953846	0.905983	0.648395
191	0.641026	0.953846	0.901709	0.629098
192	0.641026	0.953846	0.901709	0.629098
193	0.615385	0.953846	0.897436	0.609513
194	0.615385	0.953846	0.897436	0.609513
195	0.615385	0.958974	0.901709	0.622992
196	0.615385	0.958974	0.901709	0.622992
197	0.615385	0.958974	0.901709	0.622992
198	0.615385	0.958974	0.901709	0.622992
199	0.615385	0.958974	0.901709	0.622992
200	0.615385	0.958974	0.901709	0.622992
201	0.641026	0.958974	0.905983	0.64246
202	0.615385	0.958974	0.901709	0.622992
203	0.615385	0.958974	0.901709	0.622992
204	0.615385	0.958974	0.901709	0.622992
205	0.615385	0.958974	0.901709	0.622992
206	0.615385	0.958974	0.901709	0.622992
207	0.589744	0.953846	0.893162	0.589617
208	0.589744	0.958974	0.897436	0.603212
209	0.589744	0.948718	0.888889	0.576566
210	0.589744	0.948718	0.888889	0.576566
211	0.589744	0.948718	0.888889	0.576566
212	0.589744	0.953846	0.893162	0.589617

213	0.589744	0.958974	0.897436	0.603212
214	0.589744	0.953846	0.893162	0.589617
215	0.589744	0.958974	0.897436	0.603212
216	0.615385	0.964103	0.905983	0.637037
217	0.615385	0.958974	0.901709	0.622992
218	0.615385	0.958974	0.901709	0.622992
219	0.615385	0.958974	0.901709	0.622992
220	0.615385	0.958974	0.901709	0.622992
221	0.641026	0.958974	0.905983	0.64246
222	0.666667	0.958974	0.910256	0.661638
223	0.641026	0.958974	0.905983	0.64246
224	0.666667	0.953846	0.905983	0.648395
225	0.666667	0.953846	0.905983	0.648395
226	0.666667	0.953846	0.905983	0.648395
227	0.666667	0.953846	0.905983	0.648395
228	0.666667	0.958974	0.910256	0.661638
229	0.666667	0.958974	0.910256	0.661638
230	0.666667	0.953846	0.905983	0.648395
231	0.666667	0.958974	0.910256	0.661638
232	0.666667	0.958974	0.910256	0.661638
233	0.666667	0.958974	0.910256	0.661638
234	0.666667	0.958974	0.910256	0.661638
235	0.666667	0.958974	0.910256	0.661638
236	0.666667	0.958974	0.910256	0.661638
237	0.692308	0.958974	0.91453	0.680547
238	0.692308	0.953846	0.910256	0.667424
239	0.692308	0.958974	0.91453	0.680547
240	0.692308	0.958974	0.91453	0.680547
241	0.692308	0.958974	0.91453	0.680547
242	0.692308	0.958974	0.91453	0.680547
243	0.666667	0.958974	0.910256	0.661638
244	0.666667	0.958974	0.910256	0.661638
245	0.666667	0.958974	0.910256	0.661638
246	0.666667	0.958974	0.910256	0.661638
247	0.666667	0.958974	0.910256	0.661638
248	0.692308	0.958974	0.91453	0.680547
249	0.692308	0.958974	0.91453	0.680547
250	0.692308	0.958974	0.91453	0.680547
251	0.692308	0.958974	0.91453	0.680547
252	0.692308	0.953846	0.910256	0.667424
253	0.692308	0.953846	0.910256	0.667424
254	0.692308	0.953846	0.910256	0.667424
255	0.692308	0.958974	0.91453	0.680547

256	0.692308	0.958974	0.91453	0.680547
257	0.692308	0.953846	0.910256	0.667424
258	0.692308	0.953846	0.910256	0.667424
259	0.692308	0.953846	0.910256	0.667424
260	0.692308	0.958974	0.91453	0.680547
261	0.692308	0.958974	0.91453	0.680547
262	0.692308	0.958974	0.91453	0.680547
263	0.692308	0.958974	0.91453	0.680547
264	0.692308	0.958974	0.91453	0.680547
265	0.692308	0.958974	0.91453	0.680547
266	0.692308	0.958974	0.91453	0.680547
267	0.692308	0.958974	0.91453	0.680547
268	0.692308	0.958974	0.91453	0.680547
269	0.692308	0.958974	0.91453	0.680547
270	0.692308	0.958974	0.91453	0.680547
271	0.692308	0.958974	0.91453	0.680547
272	0.666667	0.958974	0.910256	0.661638
273	0.666667	0.958974	0.910256	0.661638
274	0.666667	0.964103	0.91453	0.675406
275	0.666667	0.964103	0.91453	0.675406
276	0.666667	0.964103	0.91453	0.675406
277	0.666667	0.964103	0.91453	0.675406
278	0.666667	0.964103	0.91453	0.675406
279	0.666667	0.964103	0.91453	0.675406
280	0.666667	0.964103	0.91453	0.675406
281	0.666667	0.964103	0.91453	0.675406
282	0.666667	0.964103	0.91453	0.675406
283	0.666667	0.964103	0.91453	0.675406
284	0.666667	0.964103	0.91453	0.675406
285	0.666667	0.964103	0.91453	0.675406
286	0.666667	0.964103	0.91453	0.675406
287	0.666667	0.964103	0.91453	0.675406
288	0.666667	0.964103	0.91453	0.675406
289	0.666667	0.964103	0.91453	0.675406
290	0.666667	0.964103	0.91453	0.675406
291	0.666667	0.964103	0.91453	0.675406
292	0.666667	0.964103	0.91453	0.675406
293	0.666667	0.964103	0.91453	0.675406
294	0.666667	0.964103	0.91453	0.675406
295	0.666667	0.964103	0.91453	0.675406
296	0.666667	0.964103	0.91453	0.675406
297	0.666667	0.964103	0.91453	0.675406
298	0.666667	0.964103	0.91453	0.675406

299	0.666667	0.964103	0.91453	0.675406
300	0.666667	0.964103	0.91453	0.675406
301	0.666667	0.964103	0.91453	0.675406
302	0.666667	0.964103	0.91453	0.675406
303	0.666667	0.964103	0.91453	0.675406
304	0.692308	0.964103	0.918803	0.694177
305	0.666667	0.964103	0.91453	0.675406
306	0.666667	0.964103	0.91453	0.675406
307	0.666667	0.964103	0.91453	0.675406
308	0.666667	0.964103	0.91453	0.675406
309	0.692308	0.964103	0.918803	0.694177
310	0.692308	0.964103	0.918803	0.694177
311	0.666667	0.964103	0.91453	0.675406
312	0.666667	0.964103	0.91453	0.675406
313	0.666667	0.964103	0.91453	0.675406
314	0.666667	0.964103	0.91453	0.675406
315	0.666667	0.964103	0.91453	0.675406
316	0.666667	0.964103	0.91453	0.675406
317	0.666667	0.964103	0.91453	0.675406
318	0.666667	0.964103	0.91453	0.675406
319	0.666667	0.964103	0.91453	0.675406
320	0.666667	0.964103	0.91453	0.675406
321	0.666667	0.964103	0.91453	0.675406
322	0.666667	0.964103	0.91453	0.675406
323	0.666667	0.964103	0.91453	0.675406
324	0.666667	0.964103	0.91453	0.675406
325	0.641026	0.958974	0.905983	0.64246
326	0.641026	0.958974	0.905983	0.64246
327	0.641026	0.964103	0.910256	0.656366
328	0.641026	0.958974	0.905983	0.64246
329	0.641026	0.958974	0.905983	0.64246
330	0.666667	0.964103	0.91453	0.675406
331	0.666667	0.964103	0.91453	0.675406
332	0.692308	0.964103	0.918803	0.694177
333	0.692308	0.958974	0.91453	0.680547
334	0.692308	0.953846	0.910256	0.667424
335	0.666667	0.958974	0.910256	0.661638
336	0.692308	0.964103	0.918803	0.694177
337	0.692308	0.964103	0.918803	0.694177
338	0.692308	0.964103	0.918803	0.694177
339	0.692308	0.964103	0.918803	0.694177
340	0.692308	0.964103	0.918803	0.694177
341	0.666667	0.964103	0.91453	0.675406

342	0.666667	0.964103	0.91453	0.675406
343	0.692308	0.964103	0.918803	0.694177
344	0.717949	0.964103	0.923077	0.712699
345	0.717949	0.964103	0.923077	0.712699
346	0.717949	0.964103	0.923077	0.712699
347	0.717949	0.964103	0.923077	0.712699
348	0.717949	0.964103	0.923077	0.712699
349	0.717949	0.964103	0.923077	0.712699
350	0.717949	0.964103	0.923077	0.712699
351	0.717949	0.964103	0.923077	0.712699
352	0.692308	0.958974	0.91453	0.680547
353	0.717949	0.958974	0.918803	0.699206
354	0.692308	0.958974	0.91453	0.680547
355	0.692308	0.958974	0.91453	0.680547
356	0.692308	0.964103	0.918803	0.694177
357	0.692308	0.964103	0.918803	0.694177
358	0.692308	0.964103	0.918803	0.694177
359	0.692308	0.958974	0.91453	0.680547
360	0.692308	0.958974	0.91453	0.680547
361	0.692308	0.958974	0.91453	0.680547
362	0.692308	0.958974	0.91453	0.680547
363	0.692308	0.958974	0.91453	0.680547
364	0.692308	0.958974	0.91453	0.680547
365	0.692308	0.958974	0.91453	0.680547
366	0.692308	0.958974	0.91453	0.680547
367	0.692308	0.958974	0.91453	0.680547
368	0.692308	0.958974	0.91453	0.680547
369	0.692308	0.958974	0.91453	0.680547
370	0.692308	0.958974	0.91453	0.680547
371	0.692308	0.958974	0.91453	0.680547
372	0.717949	0.958974	0.918803	0.699206
373	0.717949	0.958974	0.918803	0.699206
374	0.717949	0.958974	0.918803	0.699206
375	0.717949	0.958974	0.918803	0.699206
376	0.717949	0.964103	0.923077	0.712699
377	0.717949	0.964103	0.923077	0.712699
378	0.717949	0.958974	0.918803	0.699206
379	0.717949	0.958974	0.918803	0.699206
380	0.692308	0.953846	0.910256	0.667424
381	0.717949	0.953846	0.91453	0.686203
382	0.717949	0.958974	0.918803	0.699206
383	0.717949	0.958974	0.918803	0.699206
384	0.717949	0.958974	0.918803	0.699206

385	0.717949	0.958974	0.918803	0.699206
386	0.717949	0.958974	0.918803	0.699206
387	0.692308	0.958974	0.91453	0.680547
388	0.692308	0.958974	0.91453	0.680547
389	0.717949	0.953846	0.91453	0.686203
390	0.692308	0.953846	0.910256	0.667424
391	0.692308	0.953846	0.910256	0.667424
392	0.692308	0.953846	0.910256	0.667424
393	0.692308	0.953846	0.910256	0.667424
394	0.692308	0.964103	0.918803	0.694177
395	0.692308	0.964103	0.918803	0.694177
396	0.692308	0.964103	0.918803	0.694177
397	0.692308	0.964103	0.918803	0.694177
398	0.692308	0.958974	0.91453	0.680547
399	0.692308	0.958974	0.91453	0.680547
400	0.692308	0.958974	0.91453	0.680547
401	0.692308	0.953846	0.910256	0.667424
402	0.692308	0.948718	0.905983	0.654773
403	0.692308	0.948718	0.905983	0.654773
404	0.692308	0.958974	0.91453	0.680547
405	0.692308	0.958974	0.91453	0.680547
406	0.692308	0.958974	0.91453	0.680547
407	0.692308	0.953846	0.910256	0.667424
408	0.692308	0.953846	0.910256	0.667424
409	0.692308	0.948718	0.905983	0.654773
410	0.692308	0.948718	0.905983	0.654773
411	0.692308	0.948718	0.905983	0.654773
412	0.692308	0.953846	0.910256	0.667424
413	0.692308	0.948718	0.905983	0.654773
414	0.692308	0.948718	0.905983	0.654773
415	0.692308	0.948718	0.905983	0.654773
416	0.692308	0.948718	0.905983	0.654773
417	0.692308	0.948718	0.905983	0.654773
418	0.692308	0.953846	0.910256	0.667424
419	0.692308	0.953846	0.910256	0.667424
420	0.692308	0.953846	0.910256	0.667424
421	0.692308	0.948718	0.905983	0.654773
422	0.692308	0.953846	0.910256	0.667424
423	0.692308	0.953846	0.910256	0.667424
424	0.692308	0.953846	0.910256	0.667424
425	0.692308	0.953846	0.910256	0.667424
426	0.692308	0.953846	0.910256	0.667424
427	0.692308	0.953846	0.910256	0.667424

428	0.692308	0.953846	0.910256	0.667424
429	0.692308	0.953846	0.910256	0.667424
430	0.692308	0.953846	0.910256	0.667424
431	0.692308	0.953846	0.910256	0.667424
432	0.692308	0.953846	0.910256	0.667424
433	0.692308	0.948718	0.905983	0.654773
434	0.692308	0.948718	0.905983	0.654773
435	0.692308	0.948718	0.905983	0.654773
436	0.692308	0.948718	0.905983	0.654773
437	0.692308	0.953846	0.910256	0.667424
438	0.692308	0.953846	0.910256	0.667424
439	0.692308	0.948718	0.905983	0.654773
440	0.666667	0.948718	0.901709	0.635642
441	0.666667	0.948718	0.901709	0.635642
442	0.666667	0.948718	0.901709	0.635642
443	0.666667	0.948718	0.901709	0.635642
444	0.666667	0.948718	0.901709	0.635642
445	0.666667	0.948718	0.901709	0.635642
446	0.666667	0.948718	0.901709	0.635642
447	0.666667	0.948718	0.901709	0.635642
448	0.666667	0.948718	0.901709	0.635642
449	0.692308	0.948718	0.905983	0.654773
450	0.692308	0.948718	0.905983	0.654773
451	0.692308	0.948718	0.905983	0.654773
452	0.692308	0.948718	0.905983	0.654773
453	0.692308	0.948718	0.905983	0.654773
454	0.692308	0.948718	0.905983	0.654773
455	0.692308	0.948718	0.905983	0.654773
456	0.692308	0.948718	0.905983	0.654773
457	0.692308	0.948718	0.905983	0.654773
458	0.692308	0.948718	0.905983	0.654773
459	0.692308	0.948718	0.905983	0.654773
460	0.692308	0.948718	0.905983	0.654773
461	0.692308	0.94359	0.901709	0.642565
462	0.692308	0.94359	0.901709	0.642565
463	0.692308	0.94359	0.901709	0.642565
464	0.692308	0.94359	0.901709	0.642565
465	0.692308	0.94359	0.901709	0.642565
466	0.692308	0.94359	0.901709	0.642565
467	0.692308	0.938462	0.897436	0.630769
468	0.717949	0.938462	0.901709	0.649822
469	0.717949	0.938462	0.901709	0.649822
470	0.717949	0.938462	0.901709	0.649822

471	0.717949	0.938462	0.901709	0.649822
472	0.717949	0.938462	0.901709	0.649822
473	0.717949	0.938462	0.901709	0.649822
474	0.717949	0.938462	0.901709	0.649822
475	0.717949	0.938462	0.901709	0.649822
476	0.717949	0.938462	0.901709	0.649822
477	0.717949	0.938462	0.901709	0.649822
478	0.717949	0.938462	0.901709	0.649822
479	0.717949	0.938462	0.901709	0.649822
480	0.717949	0.94359	0.905983	0.661538
481	0.717949	0.94359	0.905983	0.661538
482	0.717949	0.938462	0.901709	0.649822
483	0.717949	0.94359	0.905983	0.661538
484	0.717949	0.938462	0.901709	0.649822
485	0.717949	0.94359	0.905983	0.661538
486	0.717949	0.948718	0.910256	0.673657
487	0.717949	0.94359	0.905983	0.661538
488	0.717949	0.948718	0.910256	0.673657
489	0.717949	0.948718	0.910256	0.673657
490	0.717949	0.948718	0.910256	0.673657
491	0.717949	0.948718	0.910256	0.673657
492	0.717949	0.948718	0.910256	0.673657
493	0.717949	0.948718	0.910256	0.673657
494	0.717949	0.953846	0.91453	0.686203
495	0.717949	0.953846	0.91453	0.686203
496	0.717949	0.953846	0.91453	0.686203
497	0.717949	0.953846	0.91453	0.686203
498	0.717949	0.953846	0.91453	0.686203
499	0.717949	0.953846	0.91453	0.686203
500	0.717949	0.953846	0.91453	0.686203

(2) Dataset D_2

Number of features	SN	SP	ACC	MCC
4	0.538462	0.969231	0.897436	0.59222
5	0.435897	0.948718	0.863248	0.448652
6	0.435897	0.953846	0.867521	0.462179
7	0.435897	0.969231	0.880342	0.507151
8	0.435897	0.974359	0.884615	0.523873
9	0.410256	0.989744	0.893162	0.559431
10	0.410256	0.989744	0.893162	0.559431
11	0.461538	0.989744	0.901709	0.601556
12	0.461538	0.989744	0.901709	0.601556

13	0.461538	0.989744	0.901709	0.601556
14	0.461538	0.989744	0.901709	0.601556
15	0.512821	0.989744	0.910256	0.641744
16	0.487179	0.989744	0.905983	0.621868
17	0.461538	0.989744	0.901709	0.601556
18	0.461538	0.989744	0.901709	0.601556
19	0.461538	0.984615	0.897436	0.581748
20	0.487179	0.984615	0.901709	0.602454
21	0.487179	0.989744	0.905983	0.621868
22	0.461538	0.989744	0.901709	0.601556
23	0.461538	0.989744	0.901709	0.601556
24	0.512821	0.989744	0.910256	0.641744
25	0.538462	0.984615	0.910256	0.64254
26	0.538462	0.989744	0.91453	0.661222
27	0.538462	0.989744	0.91453	0.661222
28	0.538462	0.989744	0.91453	0.661222
29	0.538462	0.989744	0.91453	0.661222
30	0.538462	0.989744	0.91453	0.661222
31	0.538462	0.989744	0.91453	0.661222
32	0.538462	0.989744	0.91453	0.661222
33	0.538462	0.984615	0.910256	0.64254
34	0.538462	0.984615	0.910256	0.64254
35	0.538462	0.984615	0.910256	0.64254
36	0.538462	0.984615	0.910256	0.64254
37	0.538462	0.984615	0.910256	0.64254
38	0.564103	0.984615	0.91453	0.661996
39	0.538462	0.984615	0.910256	0.64254
40	0.538462	0.984615	0.910256	0.64254
41	0.538462	0.979487	0.905983	0.624875
42	0.538462	0.979487	0.905983	0.624875
43	0.538462	0.979487	0.905983	0.624875
44	0.538462	0.979487	0.905983	0.624875
45	0.538462	0.979487	0.905983	0.624875
46	0.538462	0.979487	0.905983	0.624875
47	0.538462	0.979487	0.905983	0.624875
48	0.564103	0.979487	0.910256	0.644618
49	0.564103	0.979487	0.910256	0.644618
50	0.564103	0.979487	0.910256	0.644618
51	0.564103	0.979487	0.910256	0.644618
52	0.564103	0.979487	0.910256	0.644618
53	0.564103	0.979487	0.910256	0.644618
54	0.564103	0.979487	0.910256	0.644618
55	0.564103	0.979487	0.910256	0.644618

56	0.564103	0.974359	0.905983	0.628112
57	0.564103	0.974359	0.905983	0.628112
58	0.564103	0.974359	0.905983	0.628112
59	0.564103	0.974359	0.905983	0.628112
60	0.564103	0.974359	0.905983	0.628112
61	0.564103	0.974359	0.905983	0.628112
62	0.564103	0.969231	0.901709	0.612401
63	0.564103	0.974359	0.905983	0.628112
64	0.589744	0.974359	0.910256	0.647732
65	0.564103	0.974359	0.905983	0.628112
66	0.564103	0.979487	0.910256	0.644618
67	0.564103	0.974359	0.905983	0.628112
68	0.589744	0.974359	0.910256	0.647732
69	0.564103	0.974359	0.905983	0.628112
70	0.589744	0.969231	0.905983	0.632216
71	0.589744	0.974359	0.910256	0.647732
72	0.564103	0.969231	0.901709	0.612401
73	0.564103	0.969231	0.901709	0.612401
74	0.564103	0.969231	0.901709	0.612401
75	0.564103	0.969231	0.901709	0.612401
76	0.564103	0.974359	0.905983	0.628112
77	0.538462	0.969231	0.897436	0.59222
78	0.538462	0.958974	0.888889	0.562614
79	0.564103	0.953846	0.888889	0.569387
80	0.564103	0.953846	0.888889	0.569387
81	0.564103	0.958974	0.893162	0.583095
82	0.564103	0.958974	0.893162	0.583095
83	0.564103	0.958974	0.893162	0.583095
84	0.564103	0.958974	0.893162	0.583095
85	0.564103	0.953846	0.888889	0.569387
86	0.564103	0.948718	0.884615	0.556243
87	0.538462	0.969231	0.897436	0.59222
88	0.538462	0.969231	0.897436	0.59222
89	0.538462	0.974359	0.901709	0.60813
90	0.538462	0.969231	0.897436	0.59222
91	0.538462	0.969231	0.897436	0.59222
92	0.564103	0.969231	0.901709	0.612401
93	0.538462	0.969231	0.897436	0.59222
94	0.538462	0.969231	0.897436	0.59222
95	0.538462	0.969231	0.897436	0.59222
96	0.564103	0.969231	0.901709	0.612401
97	0.538462	0.969231	0.897436	0.59222
98	0.538462	0.953846	0.884615	0.548795

99	0.538462	0.953846	0.884615	0.548795
100	0.538462	0.953846	0.884615	0.548795
101	0.538462	0.953846	0.884615	0.548795
102	0.538462	0.953846	0.884615	0.548795
103	0.538462	0.953846	0.884615	0.548795
104	0.538462	0.948718	0.880342	0.535562
105	0.564103	0.948718	0.884615	0.556243
106	0.692308	0.953846	0.910256	0.667424
107	0.692308	0.953846	0.910256	0.667424
108	0.692308	0.953846	0.910256	0.667424
109	0.692308	0.953846	0.910256	0.667424
110	0.692308	0.964103	0.918803	0.694177
111	0.692308	0.953846	0.910256	0.667424
112	0.692308	0.953846	0.910256	0.667424
113	0.692308	0.953846	0.910256	0.667424
114	0.692308	0.953846	0.910256	0.667424
115	0.692308	0.948718	0.905983	0.654773
116	0.692308	0.953846	0.910256	0.667424
117	0.692308	0.94359	0.901709	0.642565
118	0.666667	0.958974	0.910256	0.661638
119	0.641026	0.958974	0.905983	0.64246
120	0.641026	0.958974	0.905983	0.64246
121	0.641026	0.953846	0.901709	0.629098
122	0.641026	0.953846	0.901709	0.629098
123	0.666667	0.953846	0.905983	0.648395
124	0.666667	0.953846	0.905983	0.648395
125	0.666667	0.953846	0.905983	0.648395
126	0.666667	0.953846	0.905983	0.648395
127	0.641026	0.953846	0.901709	0.629098
128	0.641026	0.953846	0.901709	0.629098
129	0.641026	0.958974	0.905983	0.64246
130	0.641026	0.958974	0.905983	0.64246
131	0.641026	0.958974	0.905983	0.64246
132	0.641026	0.958974	0.905983	0.64246
133	0.641026	0.969231	0.91453	0.670862
134	0.641026	0.969231	0.91453	0.670862
135	0.641026	0.969231	0.91453	0.670862
136	0.641026	0.969231	0.91453	0.670862
137	0.615385	0.969231	0.910256	0.651695
138	0.615385	0.958974	0.901709	0.622992
139	0.615385	0.964103	0.905983	0.637037
140	0.615385	0.964103	0.905983	0.637037
141	0.615385	0.964103	0.905983	0.637037

142	0.615385	0.964103	0.905983	0.637037
143	0.615385	0.964103	0.905983	0.637037
144	0.615385	0.958974	0.901709	0.622992
145	0.615385	0.958974	0.901709	0.622992
146	0.615385	0.958974	0.901709	0.622992
147	0.615385	0.964103	0.905983	0.637037
148	0.615385	0.964103	0.905983	0.637037
149	0.615385	0.969231	0.910256	0.651695
150	0.615385	0.969231	0.910256	0.651695
151	0.615385	0.969231	0.910256	0.651695
152	0.615385	0.969231	0.910256	0.651695
153	0.615385	0.969231	0.910256	0.651695
154	0.615385	0.969231	0.910256	0.651695
155	0.615385	0.969231	0.910256	0.651695
156	0.615385	0.969231	0.910256	0.651695
157	0.615385	0.969231	0.910256	0.651695
158	0.615385	0.969231	0.910256	0.651695
159	0.615385	0.969231	0.910256	0.651695
160	0.615385	0.974359	0.91453	0.667017
161	0.615385	0.974359	0.91453	0.667017
162	0.615385	0.969231	0.910256	0.651695
163	0.615385	0.969231	0.910256	0.651695
164	0.615385	0.969231	0.910256	0.651695
165	0.615385	0.969231	0.910256	0.651695
166	0.615385	0.964103	0.905983	0.637037
167	0.641026	0.958974	0.905983	0.64246
168	0.641026	0.958974	0.905983	0.64246
169	0.615385	0.958974	0.901709	0.622992
170	0.615385	0.958974	0.901709	0.622992
171	0.615385	0.958974	0.901709	0.622992
172	0.615385	0.958974	0.901709	0.622992
173	0.615385	0.958974	0.901709	0.622992
174	0.615385	0.958974	0.901709	0.622992
175	0.615385	0.953846	0.897436	0.609513
176	0.615385	0.953846	0.897436	0.609513
177	0.615385	0.948718	0.893162	0.596559
178	0.615385	0.953846	0.897436	0.609513
179	0.615385	0.948718	0.893162	0.596559
180	0.615385	0.948718	0.893162	0.596559
181	0.641026	0.964103	0.910256	0.656366
182	0.641026	0.964103	0.910256	0.656366
183	0.641026	0.964103	0.910256	0.656366
184	0.641026	0.964103	0.910256	0.656366

185	0.641026	0.964103	0.910256	0.656366
186	0.641026	0.964103	0.910256	0.656366
187	0.641026	0.964103	0.910256	0.656366
188	0.641026	0.964103	0.910256	0.656366
189	0.641026	0.958974	0.905983	0.64246
190	0.641026	0.958974	0.905983	0.64246
191	0.641026	0.964103	0.910256	0.656366
192	0.641026	0.958974	0.905983	0.64246
193	0.641026	0.953846	0.901709	0.629098
194	0.641026	0.958974	0.905983	0.64246
195	0.641026	0.958974	0.905983	0.64246
196	0.641026	0.953846	0.901709	0.629098
197	0.641026	0.953846	0.901709	0.629098
198	0.641026	0.953846	0.901709	0.629098
199	0.641026	0.953846	0.901709	0.629098
200	0.641026	0.953846	0.901709	0.629098
201	0.641026	0.953846	0.901709	0.629098
202	0.641026	0.958974	0.905983	0.64246
203	0.641026	0.958974	0.905983	0.64246
204	0.641026	0.969231	0.91453	0.670862
205	0.641026	0.969231	0.91453	0.670862
206	0.641026	0.964103	0.910256	0.656366
207	0.641026	0.953846	0.901709	0.629098
208	0.641026	0.953846	0.901709	0.629098
209	0.641026	0.953846	0.901709	0.629098
210	0.641026	0.953846	0.901709	0.629098
211	0.641026	0.953846	0.901709	0.629098
212	0.641026	0.958974	0.905983	0.64246
213	0.641026	0.953846	0.901709	0.629098
214	0.641026	0.953846	0.901709	0.629098
215	0.666667	0.969231	0.918803	0.689741
216	0.666667	0.969231	0.918803	0.689741
217	0.666667	0.969231	0.918803	0.689741
218	0.666667	0.969231	0.918803	0.689741
219	0.666667	0.969231	0.918803	0.689741
220	0.666667	0.969231	0.918803	0.689741
221	0.666667	0.969231	0.918803	0.689741
222	0.666667	0.969231	0.918803	0.689741
223	0.666667	0.969231	0.918803	0.689741
224	0.666667	0.969231	0.918803	0.689741
225	0.666667	0.969231	0.918803	0.689741
226	0.692308	0.974359	0.92735	0.723116
227	0.692308	0.969231	0.923077	0.708353

228	0.692308	0.964103	0.918803	0.694177
229	0.666667	0.964103	0.91453	0.675406
230	0.666667	0.964103	0.91453	0.675406
231	0.666667	0.964103	0.91453	0.675406
232	0.692308	0.964103	0.918803	0.694177
233	0.692308	0.964103	0.918803	0.694177
234	0.692308	0.964103	0.918803	0.694177
235	0.692308	0.964103	0.918803	0.694177
236	0.692308	0.969231	0.923077	0.708353
237	0.692308	0.964103	0.918803	0.694177
238	0.692308	0.964103	0.918803	0.694177
239	0.692308	0.969231	0.923077	0.708353
240	0.692308	0.969231	0.923077	0.708353
241	0.692308	0.969231	0.923077	0.708353
242	0.692308	0.969231	0.923077	0.708353
243	0.692308	0.969231	0.923077	0.708353
244	0.692308	0.969231	0.923077	0.708353
245	0.692308	0.969231	0.923077	0.708353
246	0.692308	0.964103	0.918803	0.694177
247	0.692308	0.969231	0.923077	0.708353
248	0.692308	0.969231	0.923077	0.708353
249	0.692308	0.969231	0.923077	0.708353
250	0.692308	0.969231	0.923077	0.708353
251	0.692308	0.969231	0.923077	0.708353
252	0.692308	0.969231	0.923077	0.708353
253	0.692308	0.964103	0.918803	0.694177
254	0.692308	0.964103	0.918803	0.694177
255	0.692308	0.964103	0.918803	0.694177
256	0.692308	0.974359	0.92735	0.723116
257	0.692308	0.974359	0.92735	0.723116
258	0.692308	0.969231	0.923077	0.708353
259	0.692308	0.969231	0.923077	0.708353
260	0.692308	0.969231	0.923077	0.708353
261	0.692308	0.969231	0.923077	0.708353
262	0.692308	0.969231	0.923077	0.708353
263	0.692308	0.969231	0.923077	0.708353
264	0.692308	0.969231	0.923077	0.708353
265	0.692308	0.969231	0.923077	0.708353
266	0.692308	0.969231	0.923077	0.708353
267	0.692308	0.969231	0.923077	0.708353
268	0.692308	0.969231	0.923077	0.708353
269	0.692308	0.969231	0.923077	0.708353
270	0.692308	0.969231	0.923077	0.708353

271	0.692308	0.969231	0.923077	0.708353
272	0.692308	0.969231	0.923077	0.708353
273	0.692308	0.969231	0.923077	0.708353
274	0.692308	0.969231	0.923077	0.708353
275	0.692308	0.969231	0.923077	0.708353
276	0.692308	0.969231	0.923077	0.708353
277	0.692308	0.969231	0.923077	0.708353
278	0.692308	0.969231	0.923077	0.708353
279	0.692308	0.969231	0.923077	0.708353
280	0.692308	0.969231	0.923077	0.708353
281	0.692308	0.964103	0.918803	0.694177
282	0.692308	0.964103	0.918803	0.694177
283	0.692308	0.964103	0.918803	0.694177
284	0.692308	0.964103	0.918803	0.694177
285	0.692308	0.969231	0.923077	0.708353
286	0.692308	0.969231	0.923077	0.708353
287	0.692308	0.969231	0.923077	0.708353
288	0.692308	0.969231	0.923077	0.708353
289	0.692308	0.969231	0.923077	0.708353
290	0.692308	0.969231	0.923077	0.708353
291	0.692308	0.969231	0.923077	0.708353
292	0.692308	0.969231	0.923077	0.708353
293	0.692308	0.969231	0.923077	0.708353
294	0.692308	0.969231	0.923077	0.708353
295	0.692308	0.969231	0.923077	0.708353
296	0.666667	0.969231	0.918803	0.689741
297	0.666667	0.969231	0.918803	0.689741
298	0.666667	0.969231	0.918803	0.689741
299	0.666667	0.969231	0.918803	0.689741
300	0.666667	0.969231	0.918803	0.689741
301	0.666667	0.969231	0.918803	0.689741
302	0.666667	0.969231	0.918803	0.689741
303	0.666667	0.969231	0.918803	0.689741
304	0.666667	0.969231	0.918803	0.689741
305	0.666667	0.969231	0.918803	0.689741
306	0.666667	0.969231	0.918803	0.689741
307	0.666667	0.969231	0.918803	0.689741
308	0.666667	0.969231	0.918803	0.689741
309	0.666667	0.969231	0.918803	0.689741
310	0.666667	0.969231	0.918803	0.689741
311	0.666667	0.969231	0.918803	0.689741
312	0.666667	0.969231	0.918803	0.689741
313	0.666667	0.969231	0.918803	0.689741

314	0.666667	0.969231	0.918803	0.689741
315	0.666667	0.969231	0.918803	0.689741
316	0.666667	0.969231	0.918803	0.689741
317	0.666667	0.969231	0.918803	0.689741
318	0.666667	0.969231	0.918803	0.689741
319	0.666667	0.969231	0.918803	0.689741
320	0.666667	0.969231	0.918803	0.689741
321	0.666667	0.969231	0.918803	0.689741
322	0.666667	0.969231	0.918803	0.689741
323	0.666667	0.969231	0.918803	0.689741
324	0.666667	0.969231	0.918803	0.689741
325	0.666667	0.974359	0.923077	0.704687
326	0.666667	0.974359	0.923077	0.704687
327	0.666667	0.974359	0.923077	0.704687
328	0.666667	0.974359	0.923077	0.704687
329	0.666667	0.974359	0.923077	0.704687
330	0.666667	0.974359	0.923077	0.704687
331	0.666667	0.969231	0.918803	0.689741
332	0.666667	0.964103	0.91453	0.675406
333	0.666667	0.964103	0.91453	0.675406
334	0.666667	0.964103	0.91453	0.675406
335	0.666667	0.964103	0.91453	0.675406
336	0.666667	0.964103	0.91453	0.675406
337	0.666667	0.964103	0.91453	0.675406
338	0.666667	0.964103	0.91453	0.675406
339	0.666667	0.964103	0.91453	0.675406
340	0.666667	0.964103	0.91453	0.675406
341	0.666667	0.964103	0.91453	0.675406
342	0.666667	0.964103	0.91453	0.675406
343	0.666667	0.964103	0.91453	0.675406
344	0.692308	0.964103	0.918803	0.694177
345	0.692308	0.964103	0.918803	0.694177
346	0.641026	0.964103	0.910256	0.656366
347	0.641026	0.964103	0.910256	0.656366
348	0.641026	0.969231	0.91453	0.670862
349	0.666667	0.964103	0.91453	0.675406
350	0.666667	0.964103	0.91453	0.675406
351	0.641026	0.964103	0.910256	0.656366
352	0.641026	0.964103	0.910256	0.656366
353	0.641026	0.964103	0.910256	0.656366
354	0.641026	0.964103	0.910256	0.656366
355	0.641026	0.964103	0.910256	0.656366
356	0.641026	0.964103	0.910256	0.656366

357	0.641026	0.964103	0.910256	0.656366
358	0.641026	0.964103	0.910256	0.656366
359	0.641026	0.964103	0.910256	0.656366
360	0.666667	0.964103	0.91453	0.675406
361	0.666667	0.964103	0.91453	0.675406
362	0.666667	0.964103	0.91453	0.675406
363	0.666667	0.969231	0.918803	0.689741
364	0.666667	0.969231	0.918803	0.689741
365	0.666667	0.969231	0.918803	0.689741
366	0.666667	0.969231	0.918803	0.689741
367	0.666667	0.969231	0.918803	0.689741
368	0.666667	0.969231	0.918803	0.689741
369	0.666667	0.969231	0.918803	0.689741
370	0.666667	0.969231	0.918803	0.689741
371	0.666667	0.969231	0.918803	0.689741
372	0.666667	0.964103	0.91453	0.675406
373	0.666667	0.964103	0.91453	0.675406
374	0.666667	0.958974	0.910256	0.661638
375	0.666667	0.958974	0.910256	0.661638
376	0.666667	0.958974	0.910256	0.661638
377	0.666667	0.953846	0.905983	0.648395
378	0.666667	0.953846	0.905983	0.648395
379	0.666667	0.958974	0.910256	0.661638
380	0.666667	0.958974	0.910256	0.661638
381	0.666667	0.958974	0.910256	0.661638
382	0.666667	0.958974	0.910256	0.661638
383	0.666667	0.964103	0.91453	0.675406
384	0.666667	0.964103	0.91453	0.675406
385	0.666667	0.964103	0.91453	0.675406
386	0.666667	0.964103	0.91453	0.675406
387	0.666667	0.964103	0.91453	0.675406
388	0.666667	0.964103	0.91453	0.675406
389	0.666667	0.964103	0.91453	0.675406
390	0.666667	0.964103	0.91453	0.675406
391	0.666667	0.964103	0.91453	0.675406
392	0.641026	0.958974	0.905983	0.64246
393	0.641026	0.964103	0.910256	0.656366
394	0.641026	0.964103	0.910256	0.656366
395	0.641026	0.964103	0.910256	0.656366
396	0.641026	0.964103	0.910256	0.656366
397	0.641026	0.964103	0.910256	0.656366
398	0.641026	0.964103	0.910256	0.656366
399	0.641026	0.964103	0.910256	0.656366

400	0.641026	0.964103	0.910256	0.656366
401	0.641026	0.964103	0.910256	0.656366
402	0.641026	0.964103	0.910256	0.656366
403	0.641026	0.964103	0.910256	0.656366
404	0.641026	0.964103	0.910256	0.656366
405	0.641026	0.964103	0.910256	0.656366
406	0.641026	0.964103	0.910256	0.656366
407	0.641026	0.964103	0.910256	0.656366
408	0.615385	0.958974	0.901709	0.622992
409	0.615385	0.958974	0.901709	0.622992
410	0.615385	0.958974	0.901709	0.622992
411	0.615385	0.958974	0.901709	0.622992
412	0.615385	0.958974	0.901709	0.622992
413	0.615385	0.958974	0.901709	0.622992
414	0.615385	0.958974	0.901709	0.622992
415	0.615385	0.958974	0.901709	0.622992
416	0.615385	0.958974	0.901709	0.622992
417	0.615385	0.958974	0.901709	0.622992
418	0.615385	0.958974	0.901709	0.622992
419	0.615385	0.958974	0.901709	0.622992
420	0.615385	0.964103	0.905983	0.637037
421	0.615385	0.964103	0.905983	0.637037
422	0.615385	0.964103	0.905983	0.637037
423	0.615385	0.964103	0.905983	0.637037
424	0.615385	0.964103	0.905983	0.637037
425	0.615385	0.964103	0.905983	0.637037
426	0.615385	0.964103	0.905983	0.637037
427	0.615385	0.969231	0.910256	0.651695
428	0.615385	0.969231	0.910256	0.651695
429	0.615385	0.969231	0.910256	0.651695
430	0.615385	0.964103	0.905983	0.637037
431	0.615385	0.969231	0.910256	0.651695
432	0.615385	0.969231	0.910256	0.651695
433	0.615385	0.969231	0.910256	0.651695
434	0.615385	0.969231	0.910256	0.651695
435	0.615385	0.969231	0.910256	0.651695
436	0.615385	0.969231	0.910256	0.651695
437	0.589744	0.969231	0.905983	0.632216
438	0.615385	0.969231	0.910256	0.651695
439	0.589744	0.969231	0.905983	0.632216
440	0.589744	0.969231	0.905983	0.632216
441	0.641026	0.969231	0.91453	0.670862
442	0.641026	0.969231	0.91453	0.670862

443	0.641026	0.969231	0.91453	0.670862
444	0.641026	0.969231	0.91453	0.670862
445	0.615385	0.969231	0.910256	0.651695
446	0.615385	0.969231	0.910256	0.651695
447	0.615385	0.969231	0.910256	0.651695
448	0.615385	0.969231	0.910256	0.651695
449	0.615385	0.969231	0.910256	0.651695
450	0.615385	0.969231	0.910256	0.651695
451	0.615385	0.969231	0.910256	0.651695
452	0.615385	0.969231	0.910256	0.651695
453	0.615385	0.969231	0.910256	0.651695
454	0.615385	0.969231	0.910256	0.651695
455	0.615385	0.969231	0.910256	0.651695
456	0.615385	0.969231	0.910256	0.651695
457	0.615385	0.969231	0.910256	0.651695
458	0.615385	0.969231	0.910256	0.651695
459	0.615385	0.969231	0.910256	0.651695
460	0.615385	0.969231	0.910256	0.651695
461	0.615385	0.969231	0.910256	0.651695
462	0.615385	0.969231	0.910256	0.651695
463	0.615385	0.969231	0.910256	0.651695
464	0.615385	0.969231	0.910256	0.651695
465	0.615385	0.969231	0.910256	0.651695
466	0.615385	0.969231	0.910256	0.651695
467	0.615385	0.969231	0.910256	0.651695
468	0.615385	0.969231	0.910256	0.651695
469	0.615385	0.969231	0.910256	0.651695
470	0.615385	0.964103	0.905983	0.637037
471	0.615385	0.964103	0.905983	0.637037
472	0.615385	0.964103	0.905983	0.637037
473	0.615385	0.964103	0.905983	0.637037
474	0.615385	0.964103	0.905983	0.637037
475	0.615385	0.964103	0.905983	0.637037
476	0.589744	0.953846	0.893162	0.589617
477	0.589744	0.953846	0.893162	0.589617
478	0.589744	0.958974	0.897436	0.603212
479	0.589744	0.958974	0.897436	0.603212
480	0.589744	0.953846	0.893162	0.589617
481	0.589744	0.953846	0.893162	0.589617
482	0.589744	0.953846	0.893162	0.589617
483	0.589744	0.958974	0.897436	0.603212
484	0.589744	0.958974	0.897436	0.603212
485	0.589744	0.958974	0.897436	0.603212

486	0.589744	0.964103	0.901709	0.617395
487	0.589744	0.964103	0.901709	0.617395
488	0.589744	0.964103	0.901709	0.617395
489	0.589744	0.964103	0.901709	0.617395
490	0.589744	0.964103	0.901709	0.617395
491	0.589744	0.964103	0.901709	0.617395
492	0.589744	0.964103	0.901709	0.617395
493	0.589744	0.964103	0.901709	0.617395
494	0.589744	0.964103	0.901709	0.617395
495	0.589744	0.964103	0.901709	0.617395
496	0.589744	0.958974	0.897436	0.603212
497	0.589744	0.958974	0.897436	0.603212
498	0.589744	0.958974	0.897436	0.603212
499	0.564103	0.958974	0.893162	0.583095
500	0.589744	0.964103	0.901709	0.617395

(3) Dataset D_3

Number of features	SN	SP	ACC	MCC
4	0.410256	0.994872	0.897436	0.581685
5	0.461538	0.989744	0.901709	0.601556
6	0.512821	0.989744	0.910256	0.641744
7	0.615385	0.989744	0.92735	0.717594
8	0.641026	0.994872	0.935897	0.754082
9	0.641026	0.994872	0.935897	0.754082
10	0.589744	0.994872	0.92735	0.718132
11	0.641026	0.994872	0.935897	0.754082
12	0.641026	0.994872	0.935897	0.754082
13	0.666667	0.984615	0.931624	0.736619
14	0.615385	0.979487	0.918803	0.683062
15	0.615385	0.979487	0.918803	0.683062
16	0.641026	0.974359	0.918803	0.685994
17	0.615385	0.979487	0.918803	0.683062
18	0.641026	0.984615	0.92735	0.718393
19	0.666667	0.979487	0.92735	0.720294
20	0.666667	0.974359	0.923077	0.704687
21	0.641026	0.974359	0.918803	0.685994
22	0.666667	0.979487	0.92735	0.720294
23	0.641026	0.979487	0.923077	0.701818
24	0.641026	0.979487	0.923077	0.701818
25	0.641026	0.979487	0.923077	0.701818
26	0.641026	0.984615	0.92735	0.718393
27	0.666667	0.984615	0.931624	0.736619

28	0.666667	0.984615	0.931624	0.736619
29	0.666667	0.984615	0.931624	0.736619
30	0.666667	0.984615	0.931624	0.736619
31	0.641026	0.984615	0.92735	0.718393
32	0.615385	0.984615	0.923077	0.699896
33	0.666667	0.984615	0.931624	0.736619
34	0.641026	0.984615	0.92735	0.718393
35	0.615385	0.984615	0.923077	0.699896
36	0.666667	0.989744	0.935897	0.753724
37	0.666667	0.989744	0.935897	0.753724
38	0.641026	0.989744	0.931624	0.735789
39	0.692308	0.984615	0.935897	0.754594
40	0.692308	0.984615	0.935897	0.754594
41	0.692308	0.984615	0.935897	0.754594
42	0.615385	0.984615	0.923077	0.699896
43	0.641026	0.984615	0.92735	0.718393
44	0.641026	0.984615	0.92735	0.718393
45	0.641026	0.974359	0.918803	0.685994
46	0.641026	0.979487	0.923077	0.701818
47	0.641026	0.979487	0.923077	0.701818
48	0.641026	0.979487	0.923077	0.701818
49	0.641026	0.979487	0.923077	0.701818
50	0.641026	0.979487	0.923077	0.701818
51	0.641026	0.979487	0.923077	0.701818
52	0.641026	0.984615	0.92735	0.718393
53	0.641026	0.984615	0.92735	0.718393
54	0.641026	0.984615	0.92735	0.718393
55	0.666667	0.979487	0.92735	0.720294
56	0.641026	0.979487	0.923077	0.701818
57	0.641026	0.979487	0.923077	0.701818
58	0.641026	0.979487	0.923077	0.701818
59	0.641026	0.979487	0.923077	0.701818
60	0.666667	0.979487	0.92735	0.720294
61	0.692308	0.979487	0.931624	0.738512
62	0.641026	0.979487	0.923077	0.701818
63	0.666667	0.979487	0.92735	0.720294
64	0.666667	0.979487	0.92735	0.720294
65	0.641026	0.979487	0.923077	0.701818
66	0.641026	0.979487	0.923077	0.701818
67	0.641026	0.984615	0.92735	0.718393
68	0.641026	0.989744	0.931624	0.735789
69	0.666667	0.984615	0.931624	0.736619
70	0.666667	0.979487	0.92735	0.720294

71	0.666667	0.984615	0.931624	0.736619
72	0.641026	0.989744	0.931624	0.735789
73	0.641026	0.989744	0.931624	0.735789
74	0.641026	0.989744	0.931624	0.735789
75	0.641026	0.984615	0.92735	0.718393
76	0.641026	0.989744	0.931624	0.735789
77	0.641026	0.984615	0.92735	0.718393
78	0.641026	0.984615	0.92735	0.718393
79	0.641026	0.984615	0.92735	0.718393
80	0.692308	0.989744	0.940171	0.77142
81	0.692308	0.989744	0.940171	0.77142
82	0.692308	0.984615	0.935897	0.754594
83	0.692308	0.989744	0.940171	0.77142
84	0.692308	0.989744	0.940171	0.77142
85	0.692308	0.989744	0.940171	0.77142
86	0.692308	0.989744	0.940171	0.77142
87	0.692308	0.989744	0.940171	0.77142
88	0.692308	0.989744	0.940171	0.77142
89	0.692308	0.994872	0.944444	0.789055
90	0.692308	0.994872	0.944444	0.789055
91	0.692308	0.994872	0.944444	0.789055
92	0.692308	0.989744	0.940171	0.77142
93	0.692308	0.989744	0.940171	0.77142
94	0.692308	0.989744	0.940171	0.77142
95	0.692308	0.989744	0.940171	0.77142
96	0.692308	0.989744	0.940171	0.77142
97	0.692308	0.989744	0.940171	0.77142
98	0.692308	0.994872	0.944444	0.789055
99	0.692308	0.994872	0.944444	0.789055
100	0.692308	0.989744	0.940171	0.77142
101	0.692308	0.989744	0.940171	0.77142
102	0.794872	0.994872	0.961538	0.856614
103	0.794872	0.994872	0.961538	0.856614
104	0.820513	0.994872	0.965812	0.873086
105	0.794872	0.994872	0.961538	0.856614
106	0.820513	0.994872	0.965812	0.873086
107	0.820513	0.994872	0.965812	0.873086
108	0.820513	0.994872	0.965812	0.873086
109	0.820513	0.994872	0.965812	0.873086
110	0.820513	0.994872	0.965812	0.873086
111	0.820513	0.994872	0.965812	0.873086
112	0.820513	0.994872	0.965812	0.873086
113	0.820513	0.994872	0.965812	0.873086

114	0.820513	0.994872	0.965812	0.873086
115	0.820513	0.994872	0.965812	0.873086
116	0.820513	0.994872	0.965812	0.873086
117	0.820513	0.994872	0.965812	0.873086
118	0.820513	0.994872	0.965812	0.873086
119	0.820513	0.994872	0.965812	0.873086
120	0.820513	0.994872	0.965812	0.873086
121	0.820513	0.994872	0.965812	0.873086
122	0.820513	0.989744	0.961538	0.856875
123	0.794872	0.989744	0.957265	0.84014
124	0.820513	0.994872	0.965812	0.873086
125	0.820513	0.994872	0.965812	0.873086
126	0.820513	0.994872	0.965812	0.873086
127	0.794872	0.994872	0.961538	0.856614
128	0.794872	0.994872	0.961538	0.856614
129	0.794872	0.994872	0.961538	0.856614
130	0.794872	0.994872	0.961538	0.856614
131	0.794872	0.994872	0.961538	0.856614
132	0.820513	0.994872	0.965812	0.873086
133	0.794872	0.994872	0.961538	0.856614
134	0.769231	0.994872	0.957265	0.839987
135	0.769231	0.989744	0.952991	0.823239
136	0.769231	0.989744	0.952991	0.823239
137	0.769231	0.989744	0.952991	0.823239
138	0.769231	0.989744	0.952991	0.823239
139	0.769231	0.989744	0.952991	0.823239
140	0.769231	0.989744	0.952991	0.823239
141	0.769231	0.994872	0.957265	0.839987
142	0.769231	0.994872	0.957265	0.839987
143	0.769231	0.994872	0.957265	0.839987
144	0.769231	0.994872	0.957265	0.839987
145	0.74359	0.994872	0.952991	0.823193
146	0.74359	0.989744	0.948718	0.806162
147	0.74359	0.989744	0.948718	0.806162
148	0.74359	0.989744	0.948718	0.806162
149	0.74359	0.989744	0.948718	0.806162
150	0.74359	0.989744	0.948718	0.806162
151	0.74359	0.989744	0.948718	0.806162
152	0.74359	0.989744	0.948718	0.806162
153	0.74359	0.989744	0.948718	0.806162
154	0.74359	0.989744	0.948718	0.806162
155	0.74359	0.989744	0.948718	0.806162
156	0.74359	0.984615	0.944444	0.789865

157	0.74359	0.989744	0.948718	0.806162
158	0.74359	0.994872	0.952991	0.823193
159	0.74359	0.994872	0.952991	0.823193
160	0.74359	0.994872	0.952991	0.823193
161	0.74359	0.994872	0.952991	0.823193
162	0.74359	0.989744	0.948718	0.806162
163	0.74359	0.989744	0.948718	0.806162
164	0.74359	0.984615	0.944444	0.789865
165	0.74359	0.984615	0.944444	0.789865
166	0.74359	0.984615	0.944444	0.789865
167	0.74359	0.989744	0.948718	0.806162
168	0.74359	0.989744	0.948718	0.806162
169	0.74359	0.989744	0.948718	0.806162
170	0.74359	0.989744	0.948718	0.806162
171	0.74359	0.989744	0.948718	0.806162
172	0.74359	0.989744	0.948718	0.806162
173	0.74359	0.989744	0.948718	0.806162
174	0.74359	0.994872	0.952991	0.823193
175	0.74359	0.994872	0.952991	0.823193
176	0.74359	0.994872	0.952991	0.823193
177	0.74359	0.994872	0.952991	0.823193
178	0.74359	0.989744	0.948718	0.806162
179	0.74359	0.984615	0.944444	0.789865
180	0.74359	0.989744	0.948718	0.806162
181	0.74359	0.989744	0.948718	0.806162
182	0.74359	0.989744	0.948718	0.806162
183	0.74359	0.984615	0.944444	0.789865
184	0.74359	0.984615	0.944444	0.789865
185	0.769231	0.984615	0.948718	0.807193
186	0.769231	0.984615	0.948718	0.807193
187	0.769231	0.984615	0.948718	0.807193
188	0.769231	0.984615	0.948718	0.807193
189	0.74359	0.974359	0.935897	0.759257
190	0.74359	0.974359	0.935897	0.759257
191	0.74359	0.974359	0.935897	0.759257
192	0.74359	0.979487	0.940171	0.774246
193	0.74359	0.979487	0.940171	0.774246
194	0.74359	0.979487	0.940171	0.774246
195	0.74359	0.974359	0.935897	0.759257
196	0.74359	0.974359	0.935897	0.759257
197	0.717949	0.979487	0.935897	0.75649
198	0.717949	0.974359	0.931624	0.7413
199	0.74359	0.974359	0.935897	0.759257

200	0.74359	0.974359	0.935897	0.759257
201	0.769231	0.974359	0.940171	0.777003
202	0.769231	0.974359	0.940171	0.777003
203	0.769231	0.974359	0.940171	0.777003
204	0.769231	0.974359	0.940171	0.777003
205	0.769231	0.974359	0.940171	0.777003
206	0.769231	0.974359	0.940171	0.777003
207	0.769231	0.974359	0.940171	0.777003
208	0.769231	0.974359	0.940171	0.777003
209	0.769231	0.974359	0.940171	0.777003
210	0.769231	0.974359	0.940171	0.777003
211	0.769231	0.974359	0.940171	0.777003
212	0.769231	0.974359	0.940171	0.777003
213	0.769231	0.974359	0.940171	0.777003
214	0.769231	0.974359	0.940171	0.777003
215	0.769231	0.969231	0.935897	0.76277
216	0.769231	0.969231	0.935897	0.76277
217	0.769231	0.969231	0.935897	0.76277
218	0.769231	0.969231	0.935897	0.76277
219	0.769231	0.969231	0.935897	0.76277
220	0.769231	0.969231	0.935897	0.76277
221	0.769231	0.974359	0.940171	0.777003
222	0.74359	0.979487	0.940171	0.774246
223	0.74359	0.979487	0.940171	0.774246
224	0.74359	0.979487	0.940171	0.774246
225	0.74359	0.979487	0.940171	0.774246
226	0.74359	0.979487	0.940171	0.774246
227	0.74359	0.974359	0.935897	0.759257
228	0.769231	0.974359	0.940171	0.777003
229	0.74359	0.974359	0.935897	0.759257
230	0.74359	0.974359	0.935897	0.759257
231	0.74359	0.974359	0.935897	0.759257
232	0.74359	0.974359	0.935897	0.759257
233	0.74359	0.974359	0.935897	0.759257
234	0.74359	0.974359	0.935897	0.759257
235	0.769231	0.974359	0.940171	0.777003
236	0.769231	0.974359	0.940171	0.777003
237	0.74359	0.974359	0.935897	0.759257
238	0.717949	0.974359	0.931624	0.7413
239	0.717949	0.974359	0.931624	0.7413
240	0.717949	0.974359	0.931624	0.7413
241	0.74359	0.974359	0.935897	0.759257
242	0.74359	0.974359	0.935897	0.759257

243	0.74359	0.974359	0.935897	0.759257
244	0.74359	0.974359	0.935897	0.759257
245	0.717949	0.974359	0.931624	0.7413
246	0.717949	0.974359	0.931624	0.7413
247	0.74359	0.974359	0.935897	0.759257
248	0.74359	0.974359	0.935897	0.759257
249	0.717949	0.974359	0.931624	0.7413
250	0.717949	0.974359	0.931624	0.7413
251	0.717949	0.974359	0.931624	0.7413
252	0.74359	0.974359	0.935897	0.759257
253	0.74359	0.974359	0.935897	0.759257
254	0.74359	0.974359	0.935897	0.759257
255	0.74359	0.974359	0.935897	0.759257
256	0.74359	0.974359	0.935897	0.759257
257	0.74359	0.974359	0.935897	0.759257
258	0.74359	0.974359	0.935897	0.759257
259	0.74359	0.974359	0.935897	0.759257
260	0.717949	0.974359	0.931624	0.7413
261	0.717949	0.974359	0.931624	0.7413
262	0.717949	0.974359	0.931624	0.7413
263	0.717949	0.974359	0.931624	0.7413
264	0.717949	0.974359	0.931624	0.7413
265	0.717949	0.974359	0.931624	0.7413
266	0.717949	0.974359	0.931624	0.7413
267	0.717949	0.974359	0.931624	0.7413
268	0.717949	0.974359	0.931624	0.7413
269	0.717949	0.974359	0.931624	0.7413
270	0.717949	0.974359	0.931624	0.7413
271	0.717949	0.974359	0.931624	0.7413
272	0.717949	0.974359	0.931624	0.7413
273	0.717949	0.974359	0.931624	0.7413
274	0.717949	0.974359	0.931624	0.7413
275	0.692308	0.974359	0.92735	0.723116
276	0.692308	0.974359	0.92735	0.723116
277	0.692308	0.974359	0.92735	0.723116
278	0.692308	0.974359	0.92735	0.723116
279	0.692308	0.974359	0.92735	0.723116
280	0.692308	0.974359	0.92735	0.723116
281	0.692308	0.974359	0.92735	0.723116
282	0.692308	0.974359	0.92735	0.723116
283	0.692308	0.974359	0.92735	0.723116
284	0.692308	0.974359	0.92735	0.723116
285	0.692308	0.974359	0.92735	0.723116

286	0.692308	0.974359	0.92735	0.723116
287	0.717949	0.974359	0.931624	0.7413
288	0.717949	0.974359	0.931624	0.7413
289	0.717949	0.974359	0.931624	0.7413
290	0.717949	0.974359	0.931624	0.7413
291	0.717949	0.974359	0.931624	0.7413
292	0.717949	0.974359	0.931624	0.7413
293	0.717949	0.974359	0.931624	0.7413
294	0.717949	0.974359	0.931624	0.7413
295	0.717949	0.974359	0.931624	0.7413
296	0.717949	0.974359	0.931624	0.7413
297	0.717949	0.974359	0.931624	0.7413
298	0.717949	0.974359	0.931624	0.7413
299	0.717949	0.974359	0.931624	0.7413
300	0.717949	0.974359	0.931624	0.7413
301	0.717949	0.974359	0.931624	0.7413
302	0.717949	0.974359	0.931624	0.7413
303	0.717949	0.974359	0.931624	0.7413
304	0.717949	0.974359	0.931624	0.7413
305	0.717949	0.974359	0.931624	0.7413
306	0.717949	0.974359	0.931624	0.7413
307	0.717949	0.974359	0.931624	0.7413
308	0.717949	0.974359	0.931624	0.7413
309	0.74359	0.974359	0.935897	0.759257
310	0.74359	0.974359	0.935897	0.759257
311	0.74359	0.974359	0.935897	0.759257
312	0.74359	0.974359	0.935897	0.759257
313	0.74359	0.974359	0.935897	0.759257
314	0.74359	0.974359	0.935897	0.759257
315	0.74359	0.974359	0.935897	0.759257
316	0.74359	0.974359	0.935897	0.759257
317	0.74359	0.974359	0.935897	0.759257
318	0.74359	0.974359	0.935897	0.759257
319	0.74359	0.974359	0.935897	0.759257
320	0.74359	0.974359	0.935897	0.759257
321	0.74359	0.974359	0.935897	0.759257
322	0.74359	0.974359	0.935897	0.759257
323	0.74359	0.974359	0.935897	0.759257
324	0.74359	0.974359	0.935897	0.759257
325	0.74359	0.974359	0.935897	0.759257
326	0.74359	0.974359	0.935897	0.759257
327	0.74359	0.974359	0.935897	0.759257
328	0.74359	0.974359	0.935897	0.759257

329	0.74359	0.974359	0.935897	0.759257
330	0.74359	0.974359	0.935897	0.759257
331	0.717949	0.974359	0.931624	0.7413
332	0.74359	0.974359	0.935897	0.759257
333	0.74359	0.974359	0.935897	0.759257
334	0.74359	0.974359	0.935897	0.759257
335	0.74359	0.974359	0.935897	0.759257
336	0.74359	0.974359	0.935897	0.759257
337	0.74359	0.974359	0.935897	0.759257
338	0.74359	0.974359	0.935897	0.759257
339	0.717949	0.974359	0.931624	0.7413
340	0.74359	0.974359	0.935897	0.759257
341	0.74359	0.974359	0.935897	0.759257
342	0.74359	0.974359	0.935897	0.759257
343	0.717949	0.974359	0.931624	0.7413
344	0.717949	0.974359	0.931624	0.7413
345	0.717949	0.974359	0.931624	0.7413
346	0.717949	0.974359	0.931624	0.7413
347	0.717949	0.974359	0.931624	0.7413
348	0.74359	0.974359	0.935897	0.759257
349	0.74359	0.969231	0.931624	0.744851
350	0.74359	0.969231	0.931624	0.744851
351	0.74359	0.969231	0.931624	0.744851
352	0.74359	0.969231	0.931624	0.744851
353	0.74359	0.969231	0.931624	0.744851
354	0.74359	0.969231	0.931624	0.744851
355	0.74359	0.969231	0.931624	0.744851
356	0.74359	0.969231	0.931624	0.744851
357	0.74359	0.969231	0.931624	0.744851
358	0.74359	0.969231	0.931624	0.744851
359	0.74359	0.969231	0.931624	0.744851
360	0.74359	0.969231	0.931624	0.744851
361	0.74359	0.969231	0.931624	0.744851
362	0.74359	0.969231	0.931624	0.744851
363	0.74359	0.969231	0.931624	0.744851
364	0.74359	0.969231	0.931624	0.744851
365	0.74359	0.969231	0.931624	0.744851
366	0.74359	0.969231	0.931624	0.744851
367	0.74359	0.969231	0.931624	0.744851
368	0.74359	0.969231	0.931624	0.744851
369	0.74359	0.969231	0.931624	0.744851
370	0.74359	0.969231	0.931624	0.744851
371	0.74359	0.969231	0.931624	0.744851

372	0.74359	0.969231	0.931624	0.744851
373	0.74359	0.969231	0.931624	0.744851
374	0.74359	0.969231	0.931624	0.744851
375	0.74359	0.969231	0.931624	0.744851
376	0.74359	0.969231	0.931624	0.744851
377	0.74359	0.969231	0.931624	0.744851
378	0.74359	0.969231	0.931624	0.744851
379	0.74359	0.969231	0.931624	0.744851
380	0.74359	0.969231	0.931624	0.744851
381	0.74359	0.969231	0.931624	0.744851
382	0.74359	0.969231	0.931624	0.744851
383	0.74359	0.969231	0.931624	0.744851
384	0.74359	0.969231	0.931624	0.744851
385	0.74359	0.969231	0.931624	0.744851
386	0.74359	0.969231	0.931624	0.744851
387	0.74359	0.969231	0.931624	0.744851
388	0.74359	0.969231	0.931624	0.744851
389	0.74359	0.974359	0.935897	0.759257
390	0.74359	0.974359	0.935897	0.759257
391	0.74359	0.974359	0.935897	0.759257
392	0.74359	0.974359	0.935897	0.759257
393	0.74359	0.974359	0.935897	0.759257
394	0.74359	0.969231	0.931624	0.744851
395	0.74359	0.969231	0.931624	0.744851
396	0.74359	0.969231	0.931624	0.744851
397	0.74359	0.969231	0.931624	0.744851
398	0.74359	0.969231	0.931624	0.744851
399	0.74359	0.969231	0.931624	0.744851
400	0.74359	0.969231	0.931624	0.744851
401	0.74359	0.969231	0.931624	0.744851
402	0.74359	0.969231	0.931624	0.744851
403	0.74359	0.969231	0.931624	0.744851
404	0.74359	0.969231	0.931624	0.744851
405	0.74359	0.969231	0.931624	0.744851
406	0.74359	0.969231	0.931624	0.744851
407	0.74359	0.969231	0.931624	0.744851
408	0.74359	0.969231	0.931624	0.744851
409	0.74359	0.969231	0.931624	0.744851
410	0.74359	0.969231	0.931624	0.744851
411	0.74359	0.969231	0.931624	0.744851
412	0.74359	0.964103	0.92735	0.730988
413	0.74359	0.964103	0.92735	0.730988
414	0.74359	0.964103	0.92735	0.730988

415	0.74359	0.964103	0.92735	0.730988
416	0.74359	0.964103	0.92735	0.730988
417	0.74359	0.964103	0.92735	0.730988
418	0.74359	0.964103	0.92735	0.730988
419	0.74359	0.964103	0.92735	0.730988
420	0.74359	0.964103	0.92735	0.730988
421	0.74359	0.964103	0.92735	0.730988
422	0.74359	0.964103	0.92735	0.730988
423	0.74359	0.964103	0.92735	0.730988
424	0.74359	0.964103	0.92735	0.730988
425	0.74359	0.964103	0.92735	0.730988
426	0.74359	0.964103	0.92735	0.730988
427	0.74359	0.964103	0.92735	0.730988
428	0.74359	0.964103	0.92735	0.730988
429	0.74359	0.964103	0.92735	0.730988
430	0.74359	0.964103	0.92735	0.730988
431	0.74359	0.964103	0.92735	0.730988
432	0.74359	0.964103	0.92735	0.730988
433	0.74359	0.964103	0.92735	0.730988
434	0.74359	0.964103	0.92735	0.730988
435	0.74359	0.964103	0.92735	0.730988
436	0.74359	0.964103	0.92735	0.730988
437	0.74359	0.964103	0.92735	0.730988
438	0.74359	0.964103	0.92735	0.730988
439	0.74359	0.964103	0.92735	0.730988
440	0.74359	0.969231	0.931624	0.744851
441	0.74359	0.969231	0.931624	0.744851
442	0.74359	0.969231	0.931624	0.744851
443	0.74359	0.969231	0.931624	0.744851
444	0.74359	0.969231	0.931624	0.744851
445	0.74359	0.969231	0.931624	0.744851
446	0.74359	0.969231	0.931624	0.744851
447	0.74359	0.969231	0.931624	0.744851
448	0.74359	0.969231	0.931624	0.744851
449	0.74359	0.969231	0.931624	0.744851
450	0.74359	0.964103	0.92735	0.730988
451	0.74359	0.958974	0.923077	0.717632
452	0.74359	0.958974	0.923077	0.717632
453	0.74359	0.958974	0.923077	0.717632
454	0.74359	0.958974	0.923077	0.717632
455	0.74359	0.958974	0.923077	0.717632
456	0.74359	0.958974	0.923077	0.717632
457	0.74359	0.958974	0.923077	0.717632

458	0.74359	0.958974	0.923077	0.717632
459	0.74359	0.964103	0.92735	0.730988
460	0.74359	0.964103	0.92735	0.730988
461	0.74359	0.964103	0.92735	0.730988
462	0.74359	0.958974	0.923077	0.717632
463	0.74359	0.958974	0.923077	0.717632
464	0.74359	0.958974	0.923077	0.717632
465	0.74359	0.958974	0.923077	0.717632
466	0.74359	0.964103	0.92735	0.730988
467	0.74359	0.964103	0.92735	0.730988
468	0.74359	0.964103	0.92735	0.730988
469	0.74359	0.964103	0.92735	0.730988
470	0.74359	0.964103	0.92735	0.730988
471	0.74359	0.964103	0.92735	0.730988
472	0.74359	0.964103	0.92735	0.730988
473	0.74359	0.964103	0.92735	0.730988
474	0.74359	0.964103	0.92735	0.730988
475	0.74359	0.964103	0.92735	0.730988
476	0.74359	0.964103	0.92735	0.730988
477	0.74359	0.964103	0.92735	0.730988
478	0.74359	0.964103	0.92735	0.730988
479	0.74359	0.964103	0.92735	0.730988
480	0.74359	0.964103	0.92735	0.730988
481	0.74359	0.964103	0.92735	0.730988
482	0.74359	0.964103	0.92735	0.730988
483	0.74359	0.964103	0.92735	0.730988
484	0.74359	0.964103	0.92735	0.730988
485	0.74359	0.964103	0.92735	0.730988
486	0.74359	0.964103	0.92735	0.730988
487	0.74359	0.964103	0.92735	0.730988
488	0.74359	0.964103	0.92735	0.730988
489	0.74359	0.964103	0.92735	0.730988
490	0.717949	0.964103	0.923077	0.712699
491	0.717949	0.964103	0.923077	0.712699
492	0.717949	0.964103	0.923077	0.712699
493	0.717949	0.964103	0.923077	0.712699
494	0.692308	0.969231	0.923077	0.708353
495	0.717949	0.969231	0.92735	0.726717
496	0.717949	0.969231	0.92735	0.726717
497	0.717949	0.969231	0.92735	0.726717
498	0.717949	0.969231	0.92735	0.726717
499	0.717949	0.969231	0.92735	0.726717
500	0.717949	0.969231	0.92735	0.726717

(4) Dataset D_4

Number of features	SN	SP	ACC	MCC
4	0.487179	0.989744	0.905983	0.621868
5	0.461538	0.989744	0.901709	0.601556
6	0.461538	0.984615	0.897436	0.581748
7	0.487179	0.984615	0.901709	0.602454
8	0.487179	0.984615	0.901709	0.602454
9	0.512821	0.984615	0.905983	0.622704
10	0.538462	0.984615	0.910256	0.64254
11	0.564103	0.984615	0.91453	0.661996
12	0.564103	0.984615	0.91453	0.661996
13	0.589744	0.984615	0.918803	0.681106
14	0.564103	0.984615	0.91453	0.661996
15	0.589744	0.984615	0.918803	0.681106
16	0.589744	0.984615	0.918803	0.681106
17	0.589744	0.984615	0.918803	0.681106
18	0.564103	0.984615	0.91453	0.661996
19	0.564103	0.984615	0.91453	0.661996
20	0.538462	0.984615	0.910256	0.64254
21	0.564103	0.979487	0.910256	0.644618
22	0.564103	0.979487	0.910256	0.644618
23	0.564103	0.984615	0.91453	0.661996
24	0.564103	0.984615	0.91453	0.661996
25	0.564103	0.984615	0.91453	0.661996
26	0.564103	0.984615	0.91453	0.661996
27	0.564103	0.984615	0.91453	0.661996
28	0.564103	0.984615	0.91453	0.661996
29	0.564103	0.984615	0.91453	0.661996
30	0.564103	0.984615	0.91453	0.661996
31	0.564103	0.984615	0.91453	0.661996
32	0.589744	0.984615	0.918803	0.681106
33	0.589744	0.984615	0.918803	0.681106
34	0.589744	0.984615	0.918803	0.681106
35	0.589744	0.984615	0.918803	0.681106
36	0.589744	0.984615	0.918803	0.681106
37	0.615385	0.979487	0.918803	0.683062
38	0.615385	0.974359	0.91453	0.667017
39	0.641026	0.979487	0.923077	0.701818
40	0.641026	0.979487	0.923077	0.701818
41	0.641026	0.984615	0.92735	0.718393
42	0.641026	0.984615	0.92735	0.718393
43	0.641026	0.984615	0.92735	0.718393

44	0.641026	0.979487	0.923077	0.701818
45	0.666667	0.979487	0.92735	0.720294
46	0.692308	0.984615	0.935897	0.754594
47	0.692308	0.984615	0.935897	0.754594
48	0.666667	0.984615	0.931624	0.736619
49	0.666667	0.984615	0.931624	0.736619
50	0.666667	0.984615	0.931624	0.736619
51	0.666667	0.989744	0.935897	0.753724
52	0.666667	0.989744	0.935897	0.753724
53	0.666667	0.989744	0.935897	0.753724
54	0.666667	0.989744	0.935897	0.753724
55	0.666667	0.989744	0.935897	0.753724
56	0.666667	0.989744	0.935897	0.753724
57	0.692308	0.989744	0.940171	0.77142
58	0.692308	0.989744	0.940171	0.77142
59	0.692308	0.989744	0.940171	0.77142
60	0.692308	0.989744	0.940171	0.77142
61	0.692308	0.989744	0.940171	0.77142
62	0.692308	0.979487	0.931624	0.738512
63	0.692308	0.979487	0.931624	0.738512
64	0.692308	0.979487	0.931624	0.738512
65	0.692308	0.979487	0.931624	0.738512
66	0.666667	0.984615	0.931624	0.736619
67	0.666667	0.984615	0.931624	0.736619
68	0.641026	0.979487	0.923077	0.701818
69	0.641026	0.979487	0.923077	0.701818
70	0.641026	0.979487	0.923077	0.701818
71	0.666667	0.979487	0.92735	0.720294
72	0.641026	0.979487	0.923077	0.701818
73	0.641026	0.979487	0.923077	0.701818
74	0.641026	0.979487	0.923077	0.701818
75	0.641026	0.984615	0.92735	0.718393
76	0.641026	0.984615	0.92735	0.718393
77	0.641026	0.979487	0.923077	0.701818
78	0.641026	0.984615	0.92735	0.718393
79	0.641026	0.984615	0.92735	0.718393
80	0.641026	0.974359	0.918803	0.685994
81	0.641026	0.979487	0.923077	0.701818
82	0.641026	0.979487	0.923077	0.701818
83	0.615385	0.969231	0.910256	0.651695
84	0.641026	0.969231	0.91453	0.670862
85	0.692308	0.984615	0.935897	0.754594
86	0.692308	0.984615	0.935897	0.754594

87	0.692308	0.984615	0.935897	0.754594
88	0.692308	0.984615	0.935897	0.754594
89	0.692308	0.984615	0.935897	0.754594
90	0.692308	0.984615	0.935897	0.754594
91	0.666667	0.984615	0.931624	0.736619
92	0.666667	0.979487	0.92735	0.720294
93	0.666667	0.984615	0.931624	0.736619
94	0.666667	0.984615	0.931624	0.736619
95	0.666667	0.979487	0.92735	0.720294
96	0.666667	0.979487	0.92735	0.720294
97	0.666667	0.984615	0.931624	0.736619
98	0.666667	0.984615	0.931624	0.736619
99	0.666667	0.979487	0.92735	0.720294
100	0.641026	0.979487	0.923077	0.701818
101	0.666667	0.979487	0.92735	0.720294
102	0.641026	0.969231	0.91453	0.670862
103	0.641026	0.969231	0.91453	0.670862
104	0.641026	0.969231	0.91453	0.670862
105	0.615385	0.969231	0.910256	0.651695
106	0.641026	0.969231	0.91453	0.670862
107	0.615385	0.969231	0.910256	0.651695
108	0.666667	0.969231	0.918803	0.689741
109	0.641026	0.969231	0.91453	0.670862
110	0.641026	0.969231	0.91453	0.670862
111	0.641026	0.969231	0.91453	0.670862
112	0.641026	0.969231	0.91453	0.670862
113	0.589744	0.969231	0.905983	0.632216
114	0.615385	0.969231	0.910256	0.651695
115	0.589744	0.974359	0.910256	0.647732
116	0.615385	0.969231	0.910256	0.651695
117	0.615385	0.969231	0.910256	0.651695
118	0.641026	0.969231	0.91453	0.670862
119	0.641026	0.974359	0.918803	0.685994
120	0.641026	0.974359	0.918803	0.685994
121	0.641026	0.974359	0.918803	0.685994
122	0.641026	0.974359	0.918803	0.685994
123	0.641026	0.974359	0.918803	0.685994
124	0.641026	0.974359	0.918803	0.685994
125	0.641026	0.974359	0.918803	0.685994
126	0.641026	0.974359	0.918803	0.685994
127	0.666667	0.979487	0.92735	0.720294
128	0.666667	0.979487	0.92735	0.720294
129	0.666667	0.979487	0.92735	0.720294

130	0.666667	0.979487	0.92735	0.720294
131	0.666667	0.974359	0.923077	0.704687
132	0.641026	0.974359	0.918803	0.685994
133	0.641026	0.974359	0.918803	0.685994
134	0.666667	0.974359	0.923077	0.704687
135	0.666667	0.979487	0.92735	0.720294
136	0.666667	0.979487	0.92735	0.720294
137	0.666667	0.979487	0.92735	0.720294
138	0.666667	0.979487	0.92735	0.720294
139	0.641026	0.979487	0.923077	0.701818
140	0.641026	0.979487	0.923077	0.701818
141	0.641026	0.979487	0.923077	0.701818
142	0.641026	0.979487	0.923077	0.701818
143	0.641026	0.979487	0.923077	0.701818
144	0.641026	0.974359	0.918803	0.685994
145	0.641026	0.974359	0.918803	0.685994
146	0.641026	0.974359	0.918803	0.685994
147	0.615385	0.979487	0.918803	0.683062
148	0.641026	0.979487	0.923077	0.701818
149	0.641026	0.979487	0.923077	0.701818
150	0.641026	0.979487	0.923077	0.701818
151	0.641026	0.979487	0.923077	0.701818
152	0.666667	0.979487	0.92735	0.720294
153	0.641026	0.979487	0.923077	0.701818
154	0.641026	0.979487	0.923077	0.701818
155	0.641026	0.979487	0.923077	0.701818
156	0.641026	0.974359	0.918803	0.685994
157	0.641026	0.979487	0.923077	0.701818
158	0.641026	0.979487	0.923077	0.701818
159	0.641026	0.979487	0.923077	0.701818
160	0.641026	0.979487	0.923077	0.701818
161	0.641026	0.979487	0.923077	0.701818
162	0.641026	0.979487	0.923077	0.701818
163	0.641026	0.979487	0.923077	0.701818
164	0.641026	0.979487	0.923077	0.701818
165	0.641026	0.979487	0.923077	0.701818
166	0.641026	0.979487	0.923077	0.701818
167	0.641026	0.979487	0.923077	0.701818
168	0.641026	0.974359	0.918803	0.685994
169	0.641026	0.974359	0.918803	0.685994
170	0.641026	0.974359	0.918803	0.685994
171	0.641026	0.974359	0.918803	0.685994
172	0.641026	0.974359	0.918803	0.685994

173	0.641026	0.974359	0.918803	0.685994
174	0.641026	0.974359	0.918803	0.685994
175	0.641026	0.969231	0.91453	0.670862
176	0.641026	0.969231	0.91453	0.670862
177	0.641026	0.969231	0.91453	0.670862
178	0.641026	0.969231	0.91453	0.670862
179	0.641026	0.964103	0.910256	0.656366
180	0.641026	0.964103	0.910256	0.656366
181	0.641026	0.964103	0.910256	0.656366
182	0.641026	0.958974	0.905983	0.64246
183	0.641026	0.958974	0.905983	0.64246
184	0.641026	0.958974	0.905983	0.64246
185	0.641026	0.958974	0.905983	0.64246
186	0.641026	0.958974	0.905983	0.64246
187	0.641026	0.958974	0.905983	0.64246
188	0.641026	0.958974	0.905983	0.64246
189	0.666667	0.958974	0.910256	0.661638
190	0.641026	0.953846	0.901709	0.629098
191	0.641026	0.953846	0.901709	0.629098
192	0.641026	0.958974	0.905983	0.64246
193	0.641026	0.958974	0.905983	0.64246
194	0.641026	0.958974	0.905983	0.64246
195	0.641026	0.958974	0.905983	0.64246
196	0.641026	0.958974	0.905983	0.64246
197	0.641026	0.958974	0.905983	0.64246
198	0.641026	0.958974	0.905983	0.64246
199	0.641026	0.958974	0.905983	0.64246
200	0.666667	0.958974	0.910256	0.661638
201	0.666667	0.964103	0.91453	0.675406
202	0.666667	0.958974	0.910256	0.661638
203	0.641026	0.948718	0.897436	0.616243
204	0.641026	0.938462	0.888889	0.591915
205	0.641026	0.938462	0.888889	0.591915
206	0.641026	0.938462	0.888889	0.591915
207	0.641026	0.938462	0.888889	0.591915
208	0.641026	0.933333	0.884615	0.580381
209	0.641026	0.948718	0.897436	0.616243
210	0.641026	0.948718	0.897436	0.616243
211	0.641026	0.948718	0.897436	0.616243
212	0.641026	0.953846	0.901709	0.629098
213	0.641026	0.948718	0.897436	0.616243
214	0.641026	0.94359	0.893162	0.60386
215	0.641026	0.94359	0.893162	0.60386

216	0.641026	0.948718	0.897436	0.616243
217	0.641026	0.948718	0.897436	0.616243
218	0.641026	0.948718	0.897436	0.616243
219	0.615385	0.948718	0.893162	0.596559
220	0.615385	0.948718	0.893162	0.596559
221	0.615385	0.953846	0.897436	0.609513
222	0.615385	0.953846	0.897436	0.609513
223	0.615385	0.953846	0.897436	0.609513
224	0.641026	0.953846	0.901709	0.629098
225	0.641026	0.953846	0.901709	0.629098
226	0.641026	0.953846	0.901709	0.629098
227	0.615385	0.953846	0.897436	0.609513
228	0.615385	0.948718	0.893162	0.596559
229	0.615385	0.948718	0.893162	0.596559
230	0.615385	0.948718	0.893162	0.596559
231	0.615385	0.948718	0.893162	0.596559
232	0.615385	0.948718	0.893162	0.596559
233	0.615385	0.948718	0.893162	0.596559
234	0.615385	0.948718	0.893162	0.596559
235	0.615385	0.948718	0.893162	0.596559
236	0.615385	0.948718	0.893162	0.596559
237	0.615385	0.948718	0.893162	0.596559
238	0.615385	0.953846	0.897436	0.609513
239	0.589744	0.953846	0.893162	0.589617
240	0.589744	0.948718	0.888889	0.576566
241	0.589744	0.948718	0.888889	0.576566
242	0.589744	0.948718	0.888889	0.576566
243	0.589744	0.948718	0.888889	0.576566
244	0.589744	0.953846	0.893162	0.589617
245	0.589744	0.953846	0.893162	0.589617
246	0.615385	0.953846	0.897436	0.609513
247	0.615385	0.953846	0.897436	0.609513
248	0.615385	0.953846	0.897436	0.609513
249	0.589744	0.948718	0.888889	0.576566
250	0.589744	0.948718	0.888889	0.576566
251	0.589744	0.948718	0.888889	0.576566
252	0.589744	0.948718	0.888889	0.576566
253	0.589744	0.948718	0.888889	0.576566
254	0.615385	0.948718	0.893162	0.596559
255	0.615385	0.948718	0.893162	0.596559
256	0.641026	0.948718	0.897436	0.616243
257	0.615385	0.94359	0.888889	0.584092
258	0.641026	0.938462	0.888889	0.591915

259	0.641026	0.938462	0.888889	0.591915
260	0.641026	0.938462	0.888889	0.591915
261	0.641026	0.938462	0.888889	0.591915
262	0.641026	0.938462	0.888889	0.591915
263	0.641026	0.938462	0.888889	0.591915
264	0.666667	0.938462	0.893162	0.611473
265	0.641026	0.938462	0.888889	0.591915
266	0.641026	0.938462	0.888889	0.591915
267	0.641026	0.938462	0.888889	0.591915
268	0.615385	0.938462	0.884615	0.572078
269	0.615385	0.938462	0.884615	0.572078
270	0.615385	0.938462	0.884615	0.572078
271	0.615385	0.938462	0.884615	0.572078
272	0.615385	0.933333	0.880342	0.560486
273	0.615385	0.933333	0.880342	0.560486
274	0.615385	0.938462	0.884615	0.572078
275	0.615385	0.938462	0.884615	0.572078
276	0.615385	0.938462	0.884615	0.572078
277	0.615385	0.938462	0.884615	0.572078
278	0.641026	0.938462	0.888889	0.591915
279	0.692308	0.953846	0.910256	0.667424
280	0.692308	0.948718	0.905983	0.654773
281	0.692308	0.948718	0.905983	0.654773
282	0.692308	0.948718	0.905983	0.654773
283	0.692308	0.948718	0.905983	0.654773
284	0.692308	0.948718	0.905983	0.654773
285	0.692308	0.948718	0.905983	0.654773
286	0.692308	0.94359	0.901709	0.642565
287	0.692308	0.94359	0.901709	0.642565
288	0.692308	0.948718	0.905983	0.654773
289	0.692308	0.94359	0.901709	0.642565
290	0.692308	0.948718	0.905983	0.654773
291	0.692308	0.948718	0.905983	0.654773
292	0.692308	0.948718	0.905983	0.654773
293	0.692308	0.948718	0.905983	0.654773
294	0.692308	0.948718	0.905983	0.654773
295	0.692308	0.953846	0.910256	0.667424
296	0.692308	0.953846	0.910256	0.667424
297	0.692308	0.953846	0.910256	0.667424
298	0.692308	0.953846	0.910256	0.667424
299	0.717949	0.953846	0.91453	0.686203
300	0.692308	0.948718	0.905983	0.654773
301	0.692308	0.948718	0.905983	0.654773

302	0.692308	0.948718	0.905983	0.654773
303	0.692308	0.948718	0.905983	0.654773
304	0.692308	0.948718	0.905983	0.654773
305	0.692308	0.948718	0.905983	0.654773
306	0.692308	0.948718	0.905983	0.654773
307	0.692308	0.953846	0.910256	0.667424
308	0.692308	0.953846	0.910256	0.667424
309	0.692308	0.953846	0.910256	0.667424
310	0.692308	0.953846	0.910256	0.667424
311	0.692308	0.953846	0.910256	0.667424
312	0.692308	0.948718	0.905983	0.654773
313	0.692308	0.953846	0.910256	0.667424
314	0.666667	0.948718	0.901709	0.635642
315	0.666667	0.948718	0.901709	0.635642
316	0.666667	0.953846	0.905983	0.648395
317	0.666667	0.953846	0.905983	0.648395
318	0.666667	0.953846	0.905983	0.648395
319	0.666667	0.948718	0.901709	0.635642
320	0.666667	0.948718	0.901709	0.635642
321	0.666667	0.948718	0.901709	0.635642
322	0.666667	0.948718	0.901709	0.635642
323	0.666667	0.948718	0.901709	0.635642
324	0.666667	0.948718	0.901709	0.635642
325	0.666667	0.948718	0.901709	0.635642
326	0.666667	0.948718	0.901709	0.635642
327	0.666667	0.948718	0.901709	0.635642
328	0.666667	0.948718	0.901709	0.635642
329	0.666667	0.948718	0.901709	0.635642
330	0.666667	0.948718	0.901709	0.635642
331	0.666667	0.948718	0.901709	0.635642
332	0.666667	0.948718	0.901709	0.635642
333	0.666667	0.948718	0.901709	0.635642
334	0.666667	0.948718	0.901709	0.635642
335	0.666667	0.948718	0.901709	0.635642
336	0.666667	0.948718	0.901709	0.635642
337	0.641026	0.953846	0.901709	0.629098
338	0.641026	0.953846	0.901709	0.629098
339	0.641026	0.953846	0.901709	0.629098
340	0.641026	0.953846	0.901709	0.629098
341	0.641026	0.953846	0.901709	0.629098
342	0.615385	0.953846	0.897436	0.609513
343	0.615385	0.953846	0.897436	0.609513
344	0.615385	0.948718	0.893162	0.596559

345	0.615385	0.94359	0.888889	0.584092
346	0.615385	0.94359	0.888889	0.584092
347	0.589744	0.933333	0.876068	0.540295
348	0.589744	0.933333	0.876068	0.540295
349	0.589744	0.933333	0.876068	0.540295
350	0.589744	0.933333	0.876068	0.540295
351	0.589744	0.933333	0.876068	0.540295
352	0.589744	0.933333	0.876068	0.540295
353	0.589744	0.933333	0.876068	0.540295
354	0.589744	0.933333	0.876068	0.540295
355	0.589744	0.933333	0.876068	0.540295
356	0.589744	0.933333	0.876068	0.540295
357	0.589744	0.933333	0.876068	0.540295
358	0.589744	0.933333	0.876068	0.540295
359	0.589744	0.933333	0.876068	0.540295
360	0.589744	0.933333	0.876068	0.540295
361	0.589744	0.933333	0.876068	0.540295
362	0.589744	0.933333	0.876068	0.540295
363	0.589744	0.933333	0.876068	0.540295
364	0.589744	0.933333	0.876068	0.540295
365	0.589744	0.933333	0.876068	0.540295
366	0.589744	0.933333	0.876068	0.540295
367	0.589744	0.933333	0.876068	0.540295
368	0.589744	0.933333	0.876068	0.540295
369	0.589744	0.933333	0.876068	0.540295
370	0.589744	0.933333	0.876068	0.540295
371	0.589744	0.933333	0.876068	0.540295
372	0.589744	0.933333	0.876068	0.540295
373	0.589744	0.933333	0.876068	0.540295
374	0.589744	0.933333	0.876068	0.540295
375	0.589744	0.928205	0.871795	0.529057
376	0.589744	0.928205	0.871795	0.529057
377	0.589744	0.928205	0.871795	0.529057
378	0.589744	0.928205	0.871795	0.529057
379	0.589744	0.928205	0.871795	0.529057
380	0.589744	0.928205	0.871795	0.529057
381	0.589744	0.928205	0.871795	0.529057
382	0.589744	0.928205	0.871795	0.529057
383	0.589744	0.933333	0.876068	0.540295
384	0.589744	0.923077	0.867521	0.518197
385	0.589744	0.923077	0.867521	0.518197
386	0.589744	0.928205	0.871795	0.529057
387	0.589744	0.928205	0.871795	0.529057

388	0.589744	0.923077	0.867521	0.518197
389	0.615385	0.928205	0.876068	0.549289
390	0.615385	0.928205	0.876068	0.549289
391	0.615385	0.928205	0.876068	0.549289
392	0.615385	0.933333	0.880342	0.560486
393	0.615385	0.933333	0.880342	0.560486
394	0.615385	0.933333	0.880342	0.560486
395	0.641026	0.933333	0.884615	0.580381
396	0.589744	0.933333	0.876068	0.540295
397	0.615385	0.933333	0.880342	0.560486
398	0.615385	0.933333	0.880342	0.560486
399	0.641026	0.933333	0.884615	0.580381
400	0.641026	0.933333	0.884615	0.580381
401	0.641026	0.933333	0.884615	0.580381
402	0.641026	0.933333	0.884615	0.580381
403	0.641026	0.933333	0.884615	0.580381
404	0.641026	0.933333	0.884615	0.580381
405	0.615385	0.933333	0.880342	0.560486
406	0.615385	0.933333	0.880342	0.560486
407	0.615385	0.933333	0.880342	0.560486
408	0.615385	0.933333	0.880342	0.560486
409	0.615385	0.938462	0.884615	0.572078
410	0.615385	0.938462	0.884615	0.572078
411	0.615385	0.938462	0.884615	0.572078
412	0.615385	0.938462	0.884615	0.572078
413	0.615385	0.938462	0.884615	0.572078
414	0.615385	0.938462	0.884615	0.572078
415	0.589744	0.938462	0.880342	0.55194
416	0.615385	0.938462	0.884615	0.572078
417	0.615385	0.938462	0.884615	0.572078
418	0.589744	0.938462	0.880342	0.55194
419	0.615385	0.938462	0.884615	0.572078
420	0.615385	0.938462	0.884615	0.572078
421	0.615385	0.938462	0.884615	0.572078
422	0.615385	0.938462	0.884615	0.572078
423	0.615385	0.938462	0.884615	0.572078
424	0.615385	0.938462	0.884615	0.572078
425	0.589744	0.938462	0.880342	0.55194
426	0.615385	0.938462	0.884615	0.572078
427	0.615385	0.938462	0.884615	0.572078
428	0.615385	0.938462	0.884615	0.572078
429	0.615385	0.938462	0.884615	0.572078
430	0.615385	0.938462	0.884615	0.572078

431	0.615385	0.94359	0.888889	0.584092
432	0.615385	0.94359	0.888889	0.584092
433	0.615385	0.94359	0.888889	0.584092
434	0.615385	0.94359	0.888889	0.584092
435	0.615385	0.94359	0.888889	0.584092
436	0.615385	0.94359	0.888889	0.584092
437	0.641026	0.94359	0.893162	0.60386
438	0.641026	0.94359	0.893162	0.60386
439	0.641026	0.94359	0.893162	0.60386
440	0.641026	0.94359	0.893162	0.60386
441	0.641026	0.94359	0.893162	0.60386
442	0.641026	0.938462	0.888889	0.591915
443	0.641026	0.938462	0.888889	0.591915
444	0.615385	0.938462	0.884615	0.572078
445	0.615385	0.94359	0.888889	0.584092
446	0.615385	0.94359	0.888889	0.584092
447	0.615385	0.94359	0.888889	0.584092
448	0.615385	0.938462	0.884615	0.572078
449	0.589744	0.94359	0.884615	0.564019
450	0.615385	0.94359	0.888889	0.584092
451	0.641026	0.94359	0.893162	0.60386
452	0.641026	0.94359	0.893162	0.60386
453	0.641026	0.94359	0.893162	0.60386
454	0.641026	0.948718	0.897436	0.616243
455	0.641026	0.948718	0.897436	0.616243
456	0.641026	0.948718	0.897436	0.616243
457	0.641026	0.948718	0.897436	0.616243
458	0.641026	0.948718	0.897436	0.616243
459	0.641026	0.948718	0.897436	0.616243
460	0.641026	0.948718	0.897436	0.616243
461	0.641026	0.948718	0.897436	0.616243
462	0.641026	0.948718	0.897436	0.616243
463	0.641026	0.948718	0.897436	0.616243
464	0.641026	0.948718	0.897436	0.616243
465	0.641026	0.948718	0.897436	0.616243
466	0.641026	0.948718	0.897436	0.616243
467	0.641026	0.948718	0.897436	0.616243
468	0.641026	0.948718	0.897436	0.616243
469	0.641026	0.948718	0.897436	0.616243
470	0.641026	0.948718	0.897436	0.616243
471	0.641026	0.953846	0.901709	0.629098
472	0.641026	0.953846	0.901709	0.629098
473	0.641026	0.953846	0.901709	0.629098

474	0.641026	0.953846	0.901709	0.629098
475	0.641026	0.958974	0.905983	0.64246
476	0.641026	0.958974	0.905983	0.64246
477	0.641026	0.958974	0.905983	0.64246
478	0.641026	0.958974	0.905983	0.64246
479	0.641026	0.958974	0.905983	0.64246
480	0.641026	0.958974	0.905983	0.64246
481	0.641026	0.958974	0.905983	0.64246
482	0.641026	0.958974	0.905983	0.64246
483	0.641026	0.958974	0.905983	0.64246
484	0.641026	0.958974	0.905983	0.64246
485	0.641026	0.958974	0.905983	0.64246
486	0.641026	0.958974	0.905983	0.64246
487	0.641026	0.958974	0.905983	0.64246
488	0.641026	0.964103	0.910256	0.656366
489	0.666667	0.964103	0.91453	0.675406
490	0.641026	0.964103	0.910256	0.656366
491	0.641026	0.964103	0.910256	0.656366
492	0.641026	0.964103	0.910256	0.656366
493	0.641026	0.964103	0.910256	0.656366
494	0.641026	0.964103	0.910256	0.656366
495	0.641026	0.964103	0.910256	0.656366
496	0.641026	0.964103	0.910256	0.656366
497	0.641026	0.964103	0.910256	0.656366
498	0.641026	0.964103	0.910256	0.656366
499	0.641026	0.964103	0.910256	0.656366
500	0.641026	0.964103	0.910256	0.656366

(5) Dataset D_5

Number of features	SN	SP	ACC	MCC
4	0.487179	0.964103	0.884615	0.535155
5	0.358974	0.974359	0.871795	0.454812
6	0.410256	0.969231	0.876068	0.484582
7	0.435897	0.979487	0.888889	0.541627
8	0.461538	0.974359	0.888889	0.545668
9	0.487179	0.984615	0.901709	0.602454
10	0.487179	0.979487	0.897436	0.584186
11	0.538462	0.979487	0.905983	0.624875
12	0.487179	0.979487	0.897436	0.584186
13	0.589744	0.984615	0.918803	0.681106
14	0.564103	0.994872	0.923077	0.699739
15	0.589744	0.989744	0.923077	0.699118

16	0.615385	0.984615	0.923077	0.699896
17	0.589744	0.984615	0.918803	0.681106
18	0.589744	0.984615	0.918803	0.681106
19	0.589744	0.984615	0.918803	0.681106
20	0.589744	0.979487	0.91453	0.664004
21	0.615385	0.979487	0.918803	0.683062
22	0.615385	0.979487	0.918803	0.683062
23	0.589744	0.979487	0.91453	0.664004
24	0.589744	0.979487	0.91453	0.664004
25	0.589744	0.974359	0.910256	0.647732
26	0.589744	0.974359	0.910256	0.647732
27	0.589744	0.974359	0.910256	0.647732
28	0.589744	0.969231	0.905983	0.632216
29	0.641026	0.974359	0.918803	0.685994
30	0.666667	0.974359	0.923077	0.704687
31	0.666667	0.974359	0.923077	0.704687
32	0.666667	0.974359	0.923077	0.704687
33	0.666667	0.979487	0.92735	0.720294
34	0.666667	0.974359	0.923077	0.704687
35	0.641026	0.974359	0.918803	0.685994
36	0.589744	0.974359	0.910256	0.647732
37	0.589744	0.969231	0.905983	0.632216
38	0.589744	0.969231	0.905983	0.632216
39	0.589744	0.969231	0.905983	0.632216
40	0.615385	0.964103	0.905983	0.637037
41	0.615385	0.964103	0.905983	0.637037
42	0.615385	0.964103	0.905983	0.637037
43	0.615385	0.964103	0.905983	0.637037
44	0.589744	0.964103	0.901709	0.617395
45	0.615385	0.964103	0.905983	0.637037
46	0.615385	0.964103	0.905983	0.637037
47	0.615385	0.964103	0.905983	0.637037
48	0.615385	0.964103	0.905983	0.637037
49	0.615385	0.964103	0.905983	0.637037
50	0.615385	0.964103	0.905983	0.637037
51	0.641026	0.969231	0.91453	0.670862
52	0.641026	0.969231	0.91453	0.670862
53	0.641026	0.964103	0.910256	0.656366
54	0.641026	0.964103	0.910256	0.656366
55	0.615385	0.964103	0.905983	0.637037
56	0.589744	0.964103	0.901709	0.617395
57	0.589744	0.964103	0.901709	0.617395
58	0.589744	0.964103	0.901709	0.617395

59	0.564103	0.964103	0.897436	0.597415
60	0.564103	0.958974	0.893162	0.583095
61	0.564103	0.953846	0.888889	0.569387
62	0.564103	0.958974	0.893162	0.583095
63	0.564103	0.964103	0.897436	0.597415
64	0.589744	0.958974	0.897436	0.603212
65	0.589744	0.964103	0.901709	0.617395
66	0.538462	0.964103	0.893162	0.57707
67	0.589744	0.964103	0.901709	0.617395
68	0.589744	0.964103	0.901709	0.617395
69	0.564103	0.964103	0.897436	0.597415
70	0.589744	0.969231	0.905983	0.632216
71	0.589744	0.969231	0.905983	0.632216
72	0.589744	0.964103	0.901709	0.617395
73	0.589744	0.958974	0.897436	0.603212
74	0.589744	0.964103	0.901709	0.617395
75	0.589744	0.958974	0.897436	0.603212
76	0.589744	0.964103	0.901709	0.617395
77	0.589744	0.958974	0.897436	0.603212
78	0.589744	0.964103	0.901709	0.617395
79	0.589744	0.964103	0.901709	0.617395
80	0.589744	0.964103	0.901709	0.617395
81	0.589744	0.953846	0.893162	0.589617
82	0.589744	0.948718	0.888889	0.576566
83	0.564103	0.948718	0.884615	0.556243
84	0.564103	0.948718	0.884615	0.556243
85	0.564103	0.948718	0.884615	0.556243
86	0.589744	0.94359	0.884615	0.564019
87	0.589744	0.964103	0.901709	0.617395
88	0.615385	0.953846	0.897436	0.609513
89	0.589744	0.969231	0.905983	0.632216
90	0.615385	0.969231	0.910256	0.651695
91	0.615385	0.969231	0.910256	0.651695
92	0.615385	0.969231	0.910256	0.651695
93	0.717949	0.969231	0.92735	0.726717
94	0.666667	0.969231	0.918803	0.689741
95	0.692308	0.964103	0.918803	0.694177
96	0.692308	0.948718	0.905983	0.654773
97	0.74359	0.958974	0.923077	0.717632
98	0.74359	0.958974	0.923077	0.717632
99	0.74359	0.958974	0.923077	0.717632
100	0.74359	0.958974	0.923077	0.717632
101	0.717949	0.953846	0.91453	0.686203

102	0.74359	0.953846	0.918803	0.704748
103	0.692308	0.953846	0.910256	0.667424
104	0.666667	0.953846	0.905983	0.648395
105	0.666667	0.948718	0.901709	0.635642
106	0.666667	0.948718	0.901709	0.635642
107	0.666667	0.964103	0.91453	0.675406
108	0.666667	0.964103	0.91453	0.675406
109	0.666667	0.958974	0.910256	0.661638
110	0.666667	0.958974	0.910256	0.661638
111	0.666667	0.958974	0.910256	0.661638
112	0.641026	0.958974	0.905983	0.64246
113	0.666667	0.958974	0.910256	0.661638
114	0.641026	0.958974	0.905983	0.64246
115	0.666667	0.958974	0.910256	0.661638
116	0.666667	0.958974	0.910256	0.661638
117	0.641026	0.958974	0.905983	0.64246
118	0.641026	0.958974	0.905983	0.64246
119	0.615385	0.953846	0.897436	0.609513
120	0.666667	0.958974	0.910256	0.661638
121	0.666667	0.958974	0.910256	0.661638
122	0.692308	0.958974	0.91453	0.680547
123	0.692308	0.953846	0.910256	0.667424
124	0.692308	0.964103	0.918803	0.694177
125	0.692308	0.964103	0.918803	0.694177
126	0.692308	0.964103	0.918803	0.694177
127	0.692308	0.964103	0.918803	0.694177
128	0.692308	0.964103	0.918803	0.694177
129	0.692308	0.969231	0.923077	0.708353
130	0.717949	0.969231	0.92735	0.726717
131	0.717949	0.969231	0.92735	0.726717
132	0.717949	0.969231	0.92735	0.726717
133	0.717949	0.969231	0.92735	0.726717
134	0.717949	0.969231	0.92735	0.726717
135	0.717949	0.964103	0.923077	0.712699
136	0.717949	0.964103	0.923077	0.712699
137	0.717949	0.964103	0.923077	0.712699
138	0.717949	0.964103	0.923077	0.712699
139	0.717949	0.964103	0.923077	0.712699
140	0.692308	0.964103	0.918803	0.694177
141	0.74359	0.964103	0.92735	0.730988
142	0.717949	0.964103	0.923077	0.712699
143	0.717949	0.964103	0.923077	0.712699
144	0.717949	0.964103	0.923077	0.712699

145	0.692308	0.969231	0.923077	0.708353
146	0.74359	0.969231	0.931624	0.744851
147	0.74359	0.969231	0.931624	0.744851
148	0.74359	0.969231	0.931624	0.744851
149	0.74359	0.969231	0.931624	0.744851
150	0.74359	0.958974	0.923077	0.717632
151	0.692308	0.958974	0.91453	0.680547
152	0.692308	0.958974	0.91453	0.680547
153	0.717949	0.964103	0.923077	0.712699
154	0.666667	0.969231	0.918803	0.689741
155	0.666667	0.969231	0.918803	0.689741
156	0.666667	0.969231	0.918803	0.689741
157	0.666667	0.969231	0.918803	0.689741
158	0.666667	0.964103	0.91453	0.675406
159	0.666667	0.964103	0.91453	0.675406
160	0.666667	0.964103	0.91453	0.675406
161	0.666667	0.969231	0.918803	0.689741
162	0.692308	0.964103	0.918803	0.694177
163	0.692308	0.964103	0.918803	0.694177
164	0.692308	0.964103	0.918803	0.694177
165	0.692308	0.964103	0.918803	0.694177
166	0.692308	0.964103	0.918803	0.694177
167	0.692308	0.964103	0.918803	0.694177
168	0.692308	0.964103	0.918803	0.694177
169	0.692308	0.964103	0.918803	0.694177
170	0.692308	0.964103	0.918803	0.694177
171	0.692308	0.964103	0.918803	0.694177
172	0.717949	0.964103	0.923077	0.712699
173	0.717949	0.969231	0.92735	0.726717
174	0.717949	0.974359	0.931624	0.7413
175	0.717949	0.969231	0.92735	0.726717
176	0.717949	0.969231	0.92735	0.726717
177	0.717949	0.969231	0.92735	0.726717
178	0.717949	0.974359	0.931624	0.7413
179	0.717949	0.974359	0.931624	0.7413
180	0.717949	0.974359	0.931624	0.7413
181	0.692308	0.969231	0.923077	0.708353
182	0.692308	0.969231	0.923077	0.708353
183	0.692308	0.969231	0.923077	0.708353
184	0.692308	0.969231	0.923077	0.708353
185	0.717949	0.969231	0.92735	0.726717
186	0.717949	0.969231	0.92735	0.726717
187	0.717949	0.964103	0.923077	0.712699

188	0.717949	0.964103	0.923077	0.712699
189	0.717949	0.964103	0.923077	0.712699
190	0.717949	0.964103	0.923077	0.712699
191	0.717949	0.964103	0.923077	0.712699
192	0.717949	0.964103	0.923077	0.712699
193	0.717949	0.974359	0.931624	0.7413
194	0.717949	0.974359	0.931624	0.7413
195	0.717949	0.969231	0.92735	0.726717
196	0.692308	0.964103	0.918803	0.694177
197	0.692308	0.969231	0.923077	0.708353
198	0.692308	0.969231	0.923077	0.708353
199	0.692308	0.969231	0.923077	0.708353
200	0.692308	0.969231	0.923077	0.708353
201	0.692308	0.969231	0.923077	0.708353
202	0.692308	0.969231	0.923077	0.708353
203	0.717949	0.964103	0.923077	0.712699
204	0.717949	0.964103	0.923077	0.712699
205	0.717949	0.964103	0.923077	0.712699
206	0.717949	0.964103	0.923077	0.712699
207	0.717949	0.969231	0.92735	0.726717
208	0.717949	0.969231	0.92735	0.726717
209	0.717949	0.969231	0.92735	0.726717
210	0.717949	0.964103	0.923077	0.712699
211	0.717949	0.964103	0.923077	0.712699
212	0.717949	0.964103	0.923077	0.712699
213	0.717949	0.964103	0.923077	0.712699
214	0.717949	0.964103	0.923077	0.712699
215	0.717949	0.964103	0.923077	0.712699
216	0.717949	0.964103	0.923077	0.712699
217	0.717949	0.964103	0.923077	0.712699
218	0.692308	0.969231	0.923077	0.708353
219	0.692308	0.969231	0.923077	0.708353
220	0.692308	0.964103	0.918803	0.694177
221	0.692308	0.964103	0.918803	0.694177
222	0.692308	0.964103	0.918803	0.694177
223	0.692308	0.964103	0.918803	0.694177
224	0.692308	0.964103	0.918803	0.694177
225	0.692308	0.964103	0.918803	0.694177
226	0.717949	0.964103	0.923077	0.712699
227	0.717949	0.964103	0.923077	0.712699
228	0.717949	0.964103	0.923077	0.712699
229	0.74359	0.964103	0.92735	0.730988
230	0.74359	0.964103	0.92735	0.730988

231	0.692308	0.964103	0.918803	0.694177
232	0.692308	0.964103	0.918803	0.694177
233	0.717949	0.964103	0.923077	0.712699
234	0.74359	0.964103	0.92735	0.730988
235	0.717949	0.964103	0.923077	0.712699
236	0.692308	0.964103	0.918803	0.694177
237	0.666667	0.964103	0.91453	0.675406
238	0.666667	0.964103	0.91453	0.675406
239	0.692308	0.964103	0.918803	0.694177
240	0.666667	0.964103	0.91453	0.675406
241	0.666667	0.958974	0.910256	0.661638
242	0.666667	0.958974	0.910256	0.661638
243	0.666667	0.958974	0.910256	0.661638
244	0.692308	0.958974	0.91453	0.680547
245	0.692308	0.958974	0.91453	0.680547
246	0.692308	0.958974	0.91453	0.680547
247	0.692308	0.964103	0.918803	0.694177
248	0.692308	0.964103	0.918803	0.694177
249	0.692308	0.964103	0.918803	0.694177
250	0.692308	0.964103	0.918803	0.694177
251	0.692308	0.964103	0.918803	0.694177
252	0.692308	0.964103	0.918803	0.694177
253	0.692308	0.969231	0.923077	0.708353
254	0.692308	0.969231	0.923077	0.708353
255	0.692308	0.969231	0.923077	0.708353
256	0.717949	0.964103	0.923077	0.712699
257	0.717949	0.964103	0.923077	0.712699
258	0.717949	0.964103	0.923077	0.712699
259	0.717949	0.964103	0.923077	0.712699
260	0.717949	0.964103	0.923077	0.712699
261	0.717949	0.964103	0.923077	0.712699
262	0.717949	0.964103	0.923077	0.712699
263	0.717949	0.964103	0.923077	0.712699
264	0.717949	0.964103	0.923077	0.712699
265	0.717949	0.964103	0.923077	0.712699
266	0.717949	0.964103	0.923077	0.712699
267	0.717949	0.964103	0.923077	0.712699
268	0.717949	0.964103	0.923077	0.712699
269	0.717949	0.964103	0.923077	0.712699
270	0.717949	0.964103	0.923077	0.712699
271	0.717949	0.964103	0.923077	0.712699
272	0.692308	0.964103	0.918803	0.694177
273	0.692308	0.964103	0.918803	0.694177

274	0.692308	0.964103	0.918803	0.694177
275	0.692308	0.964103	0.918803	0.694177
276	0.692308	0.964103	0.918803	0.694177
277	0.692308	0.969231	0.923077	0.708353
278	0.692308	0.969231	0.923077	0.708353
279	0.692308	0.969231	0.923077	0.708353
280	0.692308	0.969231	0.923077	0.708353
281	0.692308	0.969231	0.923077	0.708353
282	0.692308	0.969231	0.923077	0.708353
283	0.692308	0.964103	0.918803	0.694177
284	0.692308	0.964103	0.918803	0.694177
285	0.692308	0.964103	0.918803	0.694177
286	0.692308	0.964103	0.918803	0.694177
287	0.692308	0.964103	0.918803	0.694177
288	0.717949	0.969231	0.92735	0.726717
289	0.692308	0.964103	0.918803	0.694177
290	0.692308	0.964103	0.918803	0.694177
291	0.717949	0.969231	0.92735	0.726717
292	0.717949	0.969231	0.92735	0.726717
293	0.717949	0.969231	0.92735	0.726717
294	0.717949	0.969231	0.92735	0.726717
295	0.717949	0.969231	0.92735	0.726717
296	0.692308	0.969231	0.923077	0.708353
297	0.717949	0.969231	0.92735	0.726717
298	0.717949	0.969231	0.92735	0.726717
299	0.717949	0.964103	0.923077	0.712699
300	0.717949	0.964103	0.923077	0.712699
301	0.717949	0.964103	0.923077	0.712699
302	0.717949	0.964103	0.923077	0.712699
303	0.717949	0.964103	0.923077	0.712699
304	0.717949	0.964103	0.923077	0.712699
305	0.717949	0.964103	0.923077	0.712699
306	0.717949	0.964103	0.923077	0.712699
307	0.692308	0.964103	0.918803	0.694177
308	0.692308	0.964103	0.918803	0.694177
309	0.692308	0.964103	0.918803	0.694177
310	0.692308	0.964103	0.918803	0.694177
311	0.692308	0.964103	0.918803	0.694177
312	0.692308	0.964103	0.918803	0.694177
313	0.74359	0.964103	0.92735	0.730988
314	0.74359	0.964103	0.92735	0.730988
315	0.74359	0.964103	0.92735	0.730988
316	0.74359	0.964103	0.92735	0.730988

317	0.717949	0.964103	0.923077	0.712699
318	0.692308	0.964103	0.918803	0.694177
319	0.692308	0.964103	0.918803	0.694177
320	0.692308	0.964103	0.918803	0.694177
321	0.717949	0.964103	0.923077	0.712699
322	0.717949	0.964103	0.923077	0.712699
323	0.717949	0.964103	0.923077	0.712699
324	0.692308	0.964103	0.918803	0.694177
325	0.717949	0.964103	0.923077	0.712699
326	0.717949	0.964103	0.923077	0.712699
327	0.717949	0.964103	0.923077	0.712699
328	0.717949	0.958974	0.918803	0.699206
329	0.717949	0.958974	0.918803	0.699206
330	0.717949	0.964103	0.923077	0.712699
331	0.717949	0.958974	0.918803	0.699206
332	0.717949	0.953846	0.91453	0.686203
333	0.717949	0.953846	0.91453	0.686203
334	0.717949	0.953846	0.91453	0.686203
335	0.717949	0.953846	0.91453	0.686203
336	0.717949	0.953846	0.91453	0.686203
337	0.717949	0.953846	0.91453	0.686203
338	0.717949	0.953846	0.91453	0.686203
339	0.717949	0.953846	0.91453	0.686203
340	0.717949	0.953846	0.91453	0.686203
341	0.717949	0.953846	0.91453	0.686203
342	0.717949	0.953846	0.91453	0.686203
343	0.717949	0.953846	0.91453	0.686203
344	0.717949	0.953846	0.91453	0.686203
345	0.717949	0.953846	0.91453	0.686203
346	0.717949	0.953846	0.91453	0.686203
347	0.717949	0.953846	0.91453	0.686203
348	0.717949	0.953846	0.91453	0.686203
349	0.717949	0.953846	0.91453	0.686203
350	0.692308	0.964103	0.918803	0.694177
351	0.692308	0.964103	0.918803	0.694177
352	0.692308	0.964103	0.918803	0.694177
353	0.717949	0.964103	0.923077	0.712699
354	0.717949	0.964103	0.923077	0.712699
355	0.717949	0.969231	0.92735	0.726717
356	0.717949	0.969231	0.92735	0.726717
357	0.717949	0.969231	0.92735	0.726717
358	0.717949	0.969231	0.92735	0.726717
359	0.717949	0.969231	0.92735	0.726717

360	0.717949	0.969231	0.92735	0.726717
361	0.717949	0.969231	0.92735	0.726717
362	0.717949	0.969231	0.92735	0.726717
363	0.717949	0.969231	0.92735	0.726717
364	0.717949	0.969231	0.92735	0.726717
365	0.717949	0.969231	0.92735	0.726717
366	0.717949	0.958974	0.918803	0.699206
367	0.717949	0.958974	0.918803	0.699206
368	0.717949	0.958974	0.918803	0.699206
369	0.717949	0.958974	0.918803	0.699206
370	0.717949	0.958974	0.918803	0.699206
371	0.717949	0.958974	0.918803	0.699206
372	0.717949	0.964103	0.923077	0.712699
373	0.717949	0.964103	0.923077	0.712699
374	0.717949	0.958974	0.918803	0.699206
375	0.717949	0.964103	0.923077	0.712699
376	0.717949	0.964103	0.923077	0.712699
377	0.717949	0.964103	0.923077	0.712699
378	0.717949	0.964103	0.923077	0.712699
379	0.717949	0.964103	0.923077	0.712699
380	0.717949	0.964103	0.923077	0.712699
381	0.717949	0.964103	0.923077	0.712699
382	0.717949	0.964103	0.923077	0.712699
383	0.717949	0.964103	0.923077	0.712699
384	0.717949	0.964103	0.923077	0.712699
385	0.717949	0.964103	0.923077	0.712699
386	0.717949	0.964103	0.923077	0.712699
387	0.717949	0.964103	0.923077	0.712699
388	0.717949	0.964103	0.923077	0.712699
389	0.717949	0.958974	0.918803	0.699206
390	0.717949	0.958974	0.918803	0.699206
391	0.717949	0.958974	0.918803	0.699206
392	0.717949	0.958974	0.918803	0.699206
393	0.717949	0.958974	0.918803	0.699206
394	0.717949	0.958974	0.918803	0.699206
395	0.717949	0.958974	0.918803	0.699206
396	0.717949	0.958974	0.918803	0.699206
397	0.717949	0.964103	0.923077	0.712699
398	0.717949	0.964103	0.923077	0.712699
399	0.717949	0.964103	0.923077	0.712699
400	0.717949	0.964103	0.923077	0.712699
401	0.717949	0.969231	0.92735	0.726717
402	0.717949	0.969231	0.92735	0.726717

403	0.717949	0.969231	0.92735	0.726717
404	0.717949	0.969231	0.92735	0.726717
405	0.717949	0.969231	0.92735	0.726717
406	0.717949	0.969231	0.92735	0.726717
407	0.717949	0.969231	0.92735	0.726717
408	0.717949	0.969231	0.92735	0.726717
409	0.717949	0.969231	0.92735	0.726717
410	0.717949	0.969231	0.92735	0.726717
411	0.717949	0.969231	0.92735	0.726717
412	0.717949	0.969231	0.92735	0.726717
413	0.74359	0.969231	0.931624	0.744851
414	0.74359	0.969231	0.931624	0.744851
415	0.74359	0.969231	0.931624	0.744851
416	0.74359	0.969231	0.931624	0.744851
417	0.74359	0.969231	0.931624	0.744851
418	0.74359	0.969231	0.931624	0.744851
419	0.74359	0.969231	0.931624	0.744851
420	0.74359	0.969231	0.931624	0.744851
421	0.74359	0.969231	0.931624	0.744851
422	0.74359	0.969231	0.931624	0.744851
423	0.74359	0.969231	0.931624	0.744851
424	0.74359	0.969231	0.931624	0.744851
425	0.74359	0.969231	0.931624	0.744851
426	0.74359	0.969231	0.931624	0.744851
427	0.74359	0.969231	0.931624	0.744851
428	0.74359	0.969231	0.931624	0.744851
429	0.74359	0.969231	0.931624	0.744851
430	0.74359	0.969231	0.931624	0.744851
431	0.74359	0.969231	0.931624	0.744851
432	0.74359	0.969231	0.931624	0.744851
433	0.74359	0.969231	0.931624	0.744851
434	0.74359	0.969231	0.931624	0.744851
435	0.74359	0.969231	0.931624	0.744851
436	0.74359	0.969231	0.931624	0.744851
437	0.74359	0.969231	0.931624	0.744851
438	0.74359	0.969231	0.931624	0.744851
439	0.74359	0.969231	0.931624	0.744851
440	0.74359	0.969231	0.931624	0.744851
441	0.74359	0.969231	0.931624	0.744851
442	0.74359	0.969231	0.931624	0.744851
443	0.74359	0.969231	0.931624	0.744851
444	0.74359	0.969231	0.931624	0.744851
445	0.74359	0.969231	0.931624	0.744851

446	0.74359	0.969231	0.931624	0.744851
447	0.74359	0.969231	0.931624	0.744851
448	0.74359	0.969231	0.931624	0.744851
449	0.74359	0.969231	0.931624	0.744851
450	0.74359	0.969231	0.931624	0.744851
451	0.74359	0.969231	0.931624	0.744851
452	0.74359	0.969231	0.931624	0.744851
453	0.74359	0.969231	0.931624	0.744851
454	0.74359	0.969231	0.931624	0.744851
455	0.717949	0.969231	0.92735	0.726717
456	0.717949	0.969231	0.92735	0.726717
457	0.717949	0.969231	0.92735	0.726717
458	0.717949	0.969231	0.92735	0.726717
459	0.717949	0.969231	0.92735	0.726717
460	0.717949	0.969231	0.92735	0.726717
461	0.717949	0.964103	0.923077	0.712699
462	0.717949	0.964103	0.923077	0.712699
463	0.717949	0.964103	0.923077	0.712699
464	0.717949	0.964103	0.923077	0.712699
465	0.717949	0.964103	0.923077	0.712699
466	0.717949	0.964103	0.923077	0.712699
467	0.717949	0.964103	0.923077	0.712699
468	0.717949	0.964103	0.923077	0.712699
469	0.692308	0.964103	0.918803	0.694177
470	0.692308	0.964103	0.918803	0.694177
471	0.692308	0.964103	0.918803	0.694177
472	0.692308	0.964103	0.918803	0.694177
473	0.692308	0.964103	0.918803	0.694177
474	0.692308	0.964103	0.918803	0.694177
475	0.692308	0.964103	0.918803	0.694177
476	0.692308	0.964103	0.918803	0.694177
477	0.692308	0.964103	0.918803	0.694177
478	0.692308	0.964103	0.918803	0.694177
479	0.692308	0.964103	0.918803	0.694177
480	0.666667	0.969231	0.918803	0.689741
481	0.666667	0.969231	0.918803	0.689741
482	0.666667	0.969231	0.918803	0.689741
483	0.666667	0.969231	0.918803	0.689741
484	0.666667	0.969231	0.918803	0.689741
485	0.666667	0.969231	0.918803	0.689741
486	0.666667	0.969231	0.918803	0.689741
487	0.692308	0.969231	0.923077	0.708353
488	0.692308	0.969231	0.923077	0.708353

489	0.666667	0.969231	0.918803	0.689741
490	0.717949	0.969231	0.92735	0.726717
491	0.717949	0.969231	0.92735	0.726717
492	0.717949	0.969231	0.92735	0.726717
493	0.692308	0.964103	0.918803	0.694177
494	0.717949	0.969231	0.92735	0.726717
495	0.717949	0.969231	0.92735	0.726717
496	0.717949	0.964103	0.923077	0.712699
497	0.717949	0.964103	0.923077	0.712699
498	0.717949	0.964103	0.923077	0.712699
499	0.717949	0.964103	0.923077	0.712699
500	0.717949	0.964103	0.923077	0.712699

(6) Dataset D_6

Number of features	SN	SP	ACC	MCC
4	0.512821	0.979487	0.901709	0.604743
5	0.512821	0.984615	0.905983	0.622704
6	0.512821	0.984615	0.905983	0.622704
7	0.512821	0.969231	0.893162	0.571643
8	0.512821	0.969231	0.893162	0.571643
9	0.512821	0.974359	0.897436	0.587754
10	0.512821	0.974359	0.897436	0.587754
11	0.512821	0.969231	0.893162	0.571643
12	0.538462	0.979487	0.905983	0.624875
13	0.538462	0.979487	0.905983	0.624875
14	0.538462	0.979487	0.905983	0.624875
15	0.512821	0.979487	0.901709	0.604743
16	0.512821	0.974359	0.897436	0.587754
17	0.538462	0.984615	0.910256	0.64254
18	0.538462	0.984615	0.910256	0.64254
19	0.538462	0.984615	0.910256	0.64254
20	0.512821	0.984615	0.905983	0.622704
21	0.512821	0.994872	0.91453	0.661989
22	0.512821	0.984615	0.905983	0.622704
23	0.538462	0.984615	0.910256	0.64254
24	0.564103	0.989744	0.918803	0.680336
25	0.564103	0.989744	0.918803	0.680336
26	0.589744	0.989744	0.923077	0.699118
27	0.564103	0.989744	0.918803	0.680336
28	0.564103	0.989744	0.918803	0.680336
29	0.564103	0.984615	0.91453	0.661996
30	0.564103	0.984615	0.91453	0.661996

31	0.564103	0.984615	0.91453	0.661996
32	0.564103	0.984615	0.91453	0.661996
33	0.564103	0.979487	0.910256	0.644618
34	0.564103	0.984615	0.91453	0.661996
35	0.589744	0.979487	0.91453	0.664004
36	0.564103	0.979487	0.910256	0.644618
37	0.564103	0.979487	0.910256	0.644618
38	0.564103	0.979487	0.910256	0.644618
39	0.564103	0.979487	0.910256	0.644618
40	0.564103	0.979487	0.910256	0.644618
41	0.564103	0.979487	0.910256	0.644618
42	0.564103	0.979487	0.910256	0.644618
43	0.564103	0.979487	0.910256	0.644618
44	0.564103	0.979487	0.910256	0.644618
45	0.564103	0.979487	0.910256	0.644618
46	0.564103	0.979487	0.910256	0.644618
47	0.564103	0.979487	0.910256	0.644618
48	0.564103	0.979487	0.910256	0.644618
49	0.564103	0.979487	0.910256	0.644618
50	0.589744	0.974359	0.910256	0.647732
51	0.564103	0.974359	0.905983	0.628112
52	0.564103	0.969231	0.901709	0.612401
53	0.564103	0.969231	0.901709	0.612401
54	0.564103	0.969231	0.901709	0.612401
55	0.564103	0.969231	0.901709	0.612401
56	0.564103	0.969231	0.901709	0.612401
57	0.538462	0.964103	0.893162	0.57707
58	0.538462	0.969231	0.897436	0.59222
59	0.538462	0.969231	0.897436	0.59222
60	0.538462	0.969231	0.897436	0.59222
61	0.538462	0.969231	0.897436	0.59222
62	0.564103	0.969231	0.901709	0.612401
63	0.564103	0.969231	0.901709	0.612401
64	0.564103	0.969231	0.901709	0.612401
65	0.564103	0.974359	0.905983	0.628112
66	0.564103	0.974359	0.905983	0.628112
67	0.564103	0.974359	0.905983	0.628112
68	0.564103	0.969231	0.901709	0.612401
69	0.564103	0.969231	0.901709	0.612401
70	0.564103	0.969231	0.901709	0.612401
71	0.564103	0.969231	0.901709	0.612401
72	0.564103	0.969231	0.901709	0.612401
73	0.564103	0.969231	0.901709	0.612401

74	0.564103	0.969231	0.901709	0.612401
75	0.564103	0.969231	0.901709	0.612401
76	0.564103	0.964103	0.897436	0.597415
77	0.564103	0.964103	0.897436	0.597415
78	0.564103	0.964103	0.897436	0.597415
79	0.564103	0.964103	0.897436	0.597415
80	0.564103	0.969231	0.901709	0.612401
81	0.564103	0.969231	0.901709	0.612401
82	0.564103	0.964103	0.897436	0.597415
83	0.564103	0.964103	0.897436	0.597415
84	0.564103	0.969231	0.901709	0.612401
85	0.564103	0.969231	0.901709	0.612401
86	0.564103	0.969231	0.901709	0.612401
87	0.564103	0.964103	0.897436	0.597415
88	0.564103	0.964103	0.897436	0.597415
89	0.564103	0.964103	0.897436	0.597415
90	0.564103	0.964103	0.897436	0.597415
91	0.564103	0.958974	0.893162	0.583095
92	0.564103	0.964103	0.897436	0.597415
93	0.564103	0.958974	0.893162	0.583095
94	0.589744	0.964103	0.901709	0.617395
95	0.589744	0.964103	0.901709	0.617395
96	0.589744	0.964103	0.901709	0.617395
97	0.564103	0.964103	0.897436	0.597415
98	0.589744	0.964103	0.901709	0.617395
99	0.589744	0.964103	0.901709	0.617395
100	0.589744	0.964103	0.901709	0.617395
101	0.589744	0.958974	0.897436	0.603212
102	0.589744	0.958974	0.897436	0.603212
103	0.589744	0.964103	0.901709	0.617395
104	0.589744	0.964103	0.901709	0.617395
105	0.589744	0.958974	0.897436	0.603212
106	0.589744	0.964103	0.901709	0.617395
107	0.589744	0.964103	0.901709	0.617395
108	0.589744	0.964103	0.901709	0.617395
109	0.564103	0.964103	0.897436	0.597415
110	0.564103	0.964103	0.897436	0.597415
111	0.564103	0.964103	0.897436	0.597415
112	0.589744	0.964103	0.901709	0.617395
113	0.589744	0.964103	0.901709	0.617395
114	0.615385	0.964103	0.905983	0.637037
115	0.615385	0.964103	0.905983	0.637037
116	0.615385	0.964103	0.905983	0.637037

117	0.615385	0.964103	0.905983	0.637037
118	0.641026	0.964103	0.910256	0.656366
119	0.641026	0.964103	0.910256	0.656366
120	0.615385	0.964103	0.905983	0.637037
121	0.615385	0.964103	0.905983	0.637037
122	0.615385	0.964103	0.905983	0.637037
123	0.615385	0.964103	0.905983	0.637037
124	0.615385	0.964103	0.905983	0.637037
125	0.589744	0.964103	0.901709	0.617395
126	0.564103	0.964103	0.897436	0.597415
127	0.564103	0.964103	0.897436	0.597415
128	0.589744	0.969231	0.905983	0.632216
129	0.589744	0.958974	0.897436	0.603212
130	0.589744	0.958974	0.897436	0.603212
131	0.564103	0.958974	0.893162	0.583095
132	0.564103	0.958974	0.893162	0.583095
133	0.564103	0.958974	0.893162	0.583095
134	0.589744	0.958974	0.897436	0.603212
135	0.589744	0.958974	0.897436	0.603212
136	0.589744	0.958974	0.897436	0.603212
137	0.589744	0.958974	0.897436	0.603212
138	0.589744	0.958974	0.897436	0.603212
139	0.589744	0.964103	0.901709	0.617395
140	0.589744	0.964103	0.901709	0.617395
141	0.589744	0.969231	0.905983	0.632216
142	0.589744	0.974359	0.910256	0.647732
143	0.615385	0.969231	0.910256	0.651695
144	0.615385	0.969231	0.910256	0.651695
145	0.615385	0.974359	0.91453	0.667017
146	0.641026	0.974359	0.918803	0.685994
147	0.641026	0.974359	0.918803	0.685994
148	0.641026	0.974359	0.918803	0.685994
149	0.615385	0.974359	0.91453	0.667017
150	0.641026	0.974359	0.918803	0.685994
151	0.641026	0.974359	0.918803	0.685994
152	0.615385	0.974359	0.91453	0.667017
153	0.641026	0.974359	0.918803	0.685994
154	0.641026	0.974359	0.918803	0.685994
155	0.641026	0.969231	0.91453	0.670862
156	0.615385	0.974359	0.91453	0.667017
157	0.615385	0.974359	0.91453	0.667017
158	0.615385	0.974359	0.91453	0.667017
159	0.641026	0.969231	0.91453	0.670862

160	0.641026	0.969231	0.91453	0.670862
161	0.641026	0.964103	0.910256	0.656366
162	0.641026	0.964103	0.910256	0.656366
163	0.641026	0.964103	0.910256	0.656366
164	0.615385	0.964103	0.905983	0.637037
165	0.641026	0.969231	0.91453	0.670862
166	0.641026	0.964103	0.910256	0.656366
167	0.641026	0.964103	0.910256	0.656366
168	0.641026	0.964103	0.910256	0.656366
169	0.641026	0.964103	0.910256	0.656366
170	0.641026	0.969231	0.91453	0.670862
171	0.641026	0.969231	0.91453	0.670862
172	0.641026	0.969231	0.91453	0.670862
173	0.615385	0.969231	0.910256	0.651695
174	0.615385	0.969231	0.910256	0.651695
175	0.615385	0.969231	0.910256	0.651695
176	0.615385	0.969231	0.910256	0.651695
177	0.641026	0.969231	0.91453	0.670862
178	0.641026	0.969231	0.91453	0.670862
179	0.615385	0.969231	0.910256	0.651695
180	0.666667	0.964103	0.91453	0.675406
181	0.641026	0.964103	0.910256	0.656366
182	0.641026	0.964103	0.910256	0.656366
183	0.641026	0.964103	0.910256	0.656366
184	0.641026	0.964103	0.910256	0.656366
185	0.641026	0.964103	0.910256	0.656366
186	0.666667	0.964103	0.91453	0.675406
187	0.641026	0.964103	0.910256	0.656366
188	0.641026	0.964103	0.910256	0.656366
189	0.641026	0.964103	0.910256	0.656366
190	0.641026	0.964103	0.910256	0.656366
191	0.641026	0.964103	0.910256	0.656366
192	0.641026	0.964103	0.910256	0.656366
193	0.641026	0.964103	0.910256	0.656366
194	0.641026	0.964103	0.910256	0.656366
195	0.641026	0.964103	0.910256	0.656366
196	0.641026	0.964103	0.910256	0.656366
197	0.615385	0.964103	0.905983	0.637037
198	0.615385	0.964103	0.905983	0.637037
199	0.641026	0.964103	0.910256	0.656366
200	0.641026	0.964103	0.910256	0.656366
201	0.641026	0.964103	0.910256	0.656366
202	0.641026	0.964103	0.910256	0.656366

203	0.641026	0.964103	0.910256	0.656366
204	0.641026	0.964103	0.910256	0.656366
205	0.641026	0.964103	0.910256	0.656366
206	0.641026	0.964103	0.910256	0.656366
207	0.641026	0.964103	0.910256	0.656366
208	0.666667	0.964103	0.91453	0.675406
209	0.666667	0.964103	0.91453	0.675406
210	0.666667	0.964103	0.91453	0.675406
211	0.666667	0.964103	0.91453	0.675406
212	0.666667	0.964103	0.91453	0.675406
213	0.666667	0.964103	0.91453	0.675406
214	0.666667	0.964103	0.91453	0.675406
215	0.666667	0.964103	0.91453	0.675406
216	0.666667	0.964103	0.91453	0.675406
217	0.666667	0.964103	0.91453	0.675406
218	0.666667	0.964103	0.91453	0.675406
219	0.666667	0.958974	0.910256	0.661638
220	0.666667	0.958974	0.910256	0.661638
221	0.666667	0.958974	0.910256	0.661638
222	0.666667	0.958974	0.910256	0.661638
223	0.666667	0.958974	0.910256	0.661638
224	0.666667	0.958974	0.910256	0.661638
225	0.641026	0.958974	0.905983	0.64246
226	0.641026	0.958974	0.905983	0.64246
227	0.641026	0.958974	0.905983	0.64246
228	0.641026	0.958974	0.905983	0.64246
229	0.666667	0.958974	0.910256	0.661638
230	0.692308	0.958974	0.91453	0.680547
231	0.692308	0.958974	0.91453	0.680547
232	0.641026	0.958974	0.905983	0.64246
233	0.641026	0.958974	0.905983	0.64246
234	0.641026	0.958974	0.905983	0.64246
235	0.641026	0.958974	0.905983	0.64246
236	0.641026	0.958974	0.905983	0.64246
237	0.641026	0.958974	0.905983	0.64246
238	0.666667	0.958974	0.910256	0.661638
239	0.666667	0.958974	0.910256	0.661638
240	0.666667	0.958974	0.910256	0.661638
241	0.666667	0.958974	0.910256	0.661638
242	0.692308	0.958974	0.91453	0.680547
243	0.666667	0.964103	0.91453	0.675406
244	0.641026	0.958974	0.905983	0.64246
245	0.641026	0.958974	0.905983	0.64246

246	0.615385	0.953846	0.897436	0.609513
247	0.641026	0.953846	0.901709	0.629098
248	0.615385	0.953846	0.897436	0.609513
249	0.615385	0.948718	0.893162	0.596559
250	0.641026	0.953846	0.901709	0.629098
251	0.641026	0.953846	0.901709	0.629098
252	0.641026	0.953846	0.901709	0.629098
253	0.641026	0.948718	0.897436	0.616243
254	0.641026	0.948718	0.897436	0.616243
255	0.641026	0.948718	0.897436	0.616243
256	0.641026	0.948718	0.897436	0.616243
257	0.641026	0.948718	0.897436	0.616243
258	0.641026	0.958974	0.905983	0.64246
259	0.641026	0.958974	0.905983	0.64246
260	0.641026	0.958974	0.905983	0.64246
261	0.641026	0.958974	0.905983	0.64246
262	0.641026	0.953846	0.901709	0.629098
263	0.641026	0.953846	0.901709	0.629098
264	0.641026	0.953846	0.901709	0.629098
265	0.641026	0.958974	0.905983	0.64246
266	0.641026	0.958974	0.905983	0.64246
267	0.641026	0.958974	0.905983	0.64246
268	0.641026	0.958974	0.905983	0.64246
269	0.641026	0.958974	0.905983	0.64246
270	0.641026	0.958974	0.905983	0.64246
271	0.615385	0.958974	0.901709	0.622992
272	0.615385	0.958974	0.901709	0.622992
273	0.615385	0.948718	0.893162	0.596559
274	0.615385	0.953846	0.897436	0.609513
275	0.615385	0.953846	0.897436	0.609513
276	0.615385	0.953846	0.897436	0.609513
277	0.615385	0.953846	0.897436	0.609513
278	0.615385	0.948718	0.893162	0.596559
279	0.615385	0.948718	0.893162	0.596559
280	0.615385	0.948718	0.893162	0.596559
281	0.615385	0.948718	0.893162	0.596559
282	0.615385	0.948718	0.893162	0.596559
283	0.615385	0.948718	0.893162	0.596559
284	0.615385	0.948718	0.893162	0.596559
285	0.615385	0.948718	0.893162	0.596559
286	0.641026	0.948718	0.897436	0.616243
287	0.641026	0.948718	0.897436	0.616243
288	0.641026	0.948718	0.897436	0.616243

289	0.641026	0.948718	0.897436	0.616243
290	0.615385	0.948718	0.893162	0.596559
291	0.615385	0.948718	0.893162	0.596559
292	0.615385	0.948718	0.893162	0.596559
293	0.615385	0.948718	0.893162	0.596559
294	0.615385	0.948718	0.893162	0.596559
295	0.615385	0.94359	0.888889	0.584092
296	0.615385	0.94359	0.888889	0.584092
297	0.615385	0.94359	0.888889	0.584092
298	0.641026	0.94359	0.893162	0.60386
299	0.641026	0.94359	0.893162	0.60386
300	0.641026	0.94359	0.893162	0.60386
301	0.641026	0.94359	0.893162	0.60386
302	0.641026	0.948718	0.897436	0.616243
303	0.641026	0.948718	0.897436	0.616243
304	0.641026	0.948718	0.897436	0.616243
305	0.641026	0.948718	0.897436	0.616243
306	0.641026	0.948718	0.897436	0.616243
307	0.641026	0.948718	0.897436	0.616243
308	0.641026	0.948718	0.897436	0.616243
309	0.641026	0.948718	0.897436	0.616243
310	0.641026	0.948718	0.897436	0.616243
311	0.641026	0.948718	0.897436	0.616243
312	0.641026	0.948718	0.897436	0.616243
313	0.641026	0.948718	0.897436	0.616243
314	0.641026	0.948718	0.897436	0.616243
315	0.641026	0.948718	0.897436	0.616243
316	0.641026	0.948718	0.897436	0.616243
317	0.641026	0.948718	0.897436	0.616243
318	0.641026	0.948718	0.897436	0.616243
319	0.641026	0.948718	0.897436	0.616243
320	0.641026	0.94359	0.893162	0.60386
321	0.641026	0.948718	0.897436	0.616243
322	0.641026	0.94359	0.893162	0.60386
323	0.641026	0.948718	0.897436	0.616243
324	0.641026	0.948718	0.897436	0.616243
325	0.641026	0.948718	0.897436	0.616243
326	0.641026	0.948718	0.897436	0.616243
327	0.641026	0.948718	0.897436	0.616243
328	0.641026	0.948718	0.897436	0.616243
329	0.641026	0.948718	0.897436	0.616243
330	0.641026	0.948718	0.897436	0.616243
331	0.641026	0.948718	0.897436	0.616243

332	0.641026	0.94359	0.893162	0.60386
333	0.641026	0.94359	0.893162	0.60386
334	0.641026	0.94359	0.893162	0.60386
335	0.641026	0.94359	0.893162	0.60386
336	0.641026	0.94359	0.893162	0.60386
337	0.641026	0.94359	0.893162	0.60386
338	0.641026	0.94359	0.893162	0.60386
339	0.641026	0.938462	0.888889	0.591915
340	0.641026	0.938462	0.888889	0.591915
341	0.641026	0.938462	0.888889	0.591915
342	0.641026	0.938462	0.888889	0.591915
343	0.641026	0.938462	0.888889	0.591915
344	0.641026	0.933333	0.884615	0.580381
345	0.666667	0.933333	0.888889	0.6
346	0.666667	0.933333	0.888889	0.6
347	0.666667	0.933333	0.888889	0.6
348	0.666667	0.933333	0.888889	0.6
349	0.666667	0.933333	0.888889	0.6
350	0.641026	0.933333	0.884615	0.580381
351	0.641026	0.933333	0.884615	0.580381
352	0.641026	0.933333	0.884615	0.580381
353	0.641026	0.933333	0.884615	0.580381
354	0.641026	0.933333	0.884615	0.580381
355	0.641026	0.933333	0.884615	0.580381
356	0.641026	0.933333	0.884615	0.580381
357	0.641026	0.933333	0.884615	0.580381
358	0.666667	0.948718	0.901709	0.635642
359	0.666667	0.948718	0.901709	0.635642
360	0.666667	0.948718	0.901709	0.635642
361	0.666667	0.948718	0.901709	0.635642
362	0.666667	0.953846	0.905983	0.648395
363	0.666667	0.953846	0.905983	0.648395
364	0.666667	0.953846	0.905983	0.648395
365	0.666667	0.948718	0.901709	0.635642
366	0.666667	0.953846	0.905983	0.648395
367	0.666667	0.948718	0.901709	0.635642
368	0.666667	0.948718	0.901709	0.635642
369	0.666667	0.948718	0.901709	0.635642
370	0.666667	0.953846	0.905983	0.648395
371	0.666667	0.953846	0.905983	0.648395
372	0.666667	0.953846	0.905983	0.648395
373	0.666667	0.953846	0.905983	0.648395
374	0.666667	0.938462	0.893162	0.611473

375	0.666667	0.938462	0.893162	0.611473
376	0.666667	0.938462	0.893162	0.611473
377	0.666667	0.938462	0.893162	0.611473
378	0.666667	0.938462	0.893162	0.611473
379	0.666667	0.938462	0.893162	0.611473
380	0.666667	0.938462	0.893162	0.611473
381	0.666667	0.938462	0.893162	0.611473
382	0.666667	0.938462	0.893162	0.611473
383	0.666667	0.938462	0.893162	0.611473
384	0.666667	0.933333	0.888889	0.6
385	0.666667	0.94359	0.897436	0.623344
386	0.666667	0.94359	0.897436	0.623344
387	0.666667	0.94359	0.897436	0.623344
388	0.666667	0.948718	0.901709	0.635642
389	0.666667	0.948718	0.901709	0.635642
390	0.666667	0.948718	0.901709	0.635642
391	0.666667	0.948718	0.901709	0.635642
392	0.666667	0.948718	0.901709	0.635642
393	0.666667	0.94359	0.897436	0.623344
394	0.666667	0.94359	0.897436	0.623344
395	0.666667	0.94359	0.897436	0.623344
396	0.666667	0.938462	0.893162	0.611473
397	0.666667	0.938462	0.893162	0.611473
398	0.666667	0.938462	0.893162	0.611473
399	0.666667	0.938462	0.893162	0.611473
400	0.666667	0.938462	0.893162	0.611473
401	0.666667	0.94359	0.897436	0.623344
402	0.666667	0.938462	0.893162	0.611473
403	0.666667	0.94359	0.897436	0.623344
404	0.666667	0.94359	0.897436	0.623344
405	0.666667	0.94359	0.897436	0.623344
406	0.666667	0.94359	0.897436	0.623344
407	0.666667	0.94359	0.897436	0.623344
408	0.666667	0.94359	0.897436	0.623344
409	0.666667	0.94359	0.897436	0.623344
410	0.666667	0.94359	0.897436	0.623344
411	0.666667	0.94359	0.897436	0.623344
412	0.666667	0.94359	0.897436	0.623344
413	0.666667	0.94359	0.897436	0.623344
414	0.666667	0.94359	0.897436	0.623344
415	0.666667	0.94359	0.897436	0.623344
416	0.666667	0.94359	0.897436	0.623344
417	0.666667	0.94359	0.897436	0.623344

418	0.666667	0.94359	0.897436	0.623344
419	0.666667	0.94359	0.897436	0.623344
420	0.666667	0.94359	0.897436	0.623344
421	0.666667	0.94359	0.897436	0.623344
422	0.666667	0.94359	0.897436	0.623344
423	0.666667	0.94359	0.897436	0.623344
424	0.666667	0.94359	0.897436	0.623344
425	0.666667	0.94359	0.897436	0.623344
426	0.666667	0.94359	0.897436	0.623344
427	0.666667	0.94359	0.897436	0.623344
428	0.666667	0.938462	0.893162	0.611473
429	0.666667	0.938462	0.893162	0.611473
430	0.666667	0.938462	0.893162	0.611473
431	0.666667	0.938462	0.893162	0.611473
432	0.666667	0.938462	0.893162	0.611473
433	0.666667	0.938462	0.893162	0.611473
434	0.666667	0.94359	0.897436	0.623344
435	0.666667	0.94359	0.897436	0.623344
436	0.666667	0.94359	0.897436	0.623344
437	0.666667	0.94359	0.897436	0.623344
438	0.666667	0.94359	0.897436	0.623344
439	0.666667	0.94359	0.897436	0.623344
440	0.666667	0.938462	0.893162	0.611473
441	0.666667	0.938462	0.893162	0.611473
442	0.666667	0.938462	0.893162	0.611473
443	0.666667	0.938462	0.893162	0.611473
444	0.666667	0.938462	0.893162	0.611473
445	0.666667	0.938462	0.893162	0.611473
446	0.666667	0.938462	0.893162	0.611473
447	0.666667	0.938462	0.893162	0.611473
448	0.666667	0.938462	0.893162	0.611473
449	0.666667	0.938462	0.893162	0.611473
450	0.666667	0.938462	0.893162	0.611473
451	0.666667	0.938462	0.893162	0.611473
452	0.666667	0.938462	0.893162	0.611473
453	0.666667	0.938462	0.893162	0.611473
454	0.666667	0.938462	0.893162	0.611473
455	0.666667	0.938462	0.893162	0.611473
456	0.666667	0.948718	0.901709	0.635642
457	0.666667	0.948718	0.901709	0.635642
458	0.666667	0.948718	0.901709	0.635642
459	0.666667	0.948718	0.901709	0.635642
460	0.666667	0.948718	0.901709	0.635642

461	0.666667	0.94359	0.897436	0.623344
462	0.666667	0.94359	0.897436	0.623344
463	0.666667	0.948718	0.901709	0.635642
464	0.666667	0.948718	0.901709	0.635642
465	0.666667	0.948718	0.901709	0.635642
466	0.666667	0.948718	0.901709	0.635642
467	0.666667	0.948718	0.901709	0.635642
468	0.666667	0.948718	0.901709	0.635642
469	0.666667	0.948718	0.901709	0.635642
470	0.666667	0.948718	0.901709	0.635642
471	0.666667	0.948718	0.901709	0.635642
472	0.666667	0.948718	0.901709	0.635642
473	0.666667	0.94359	0.897436	0.623344
474	0.666667	0.94359	0.897436	0.623344
475	0.666667	0.94359	0.897436	0.623344
476	0.666667	0.94359	0.897436	0.623344
477	0.666667	0.94359	0.897436	0.623344
478	0.666667	0.94359	0.897436	0.623344
479	0.666667	0.938462	0.893162	0.611473
480	0.666667	0.938462	0.893162	0.611473
481	0.666667	0.938462	0.893162	0.611473
482	0.666667	0.938462	0.893162	0.611473
483	0.666667	0.938462	0.893162	0.611473
484	0.666667	0.938462	0.893162	0.611473
485	0.666667	0.938462	0.893162	0.611473
486	0.666667	0.938462	0.893162	0.611473
487	0.666667	0.938462	0.893162	0.611473
488	0.666667	0.938462	0.893162	0.611473
489	0.666667	0.938462	0.893162	0.611473
490	0.666667	0.938462	0.893162	0.611473
491	0.666667	0.938462	0.893162	0.611473
492	0.666667	0.938462	0.893162	0.611473
493	0.666667	0.938462	0.893162	0.611473
494	0.666667	0.938462	0.893162	0.611473
495	0.666667	0.938462	0.893162	0.611473
496	0.666667	0.938462	0.893162	0.611473
497	0.666667	0.938462	0.893162	0.611473
498	0.666667	0.938462	0.893162	0.611473
499	0.666667	0.938462	0.893162	0.611473
500	0.666667	0.938462	0.893162	0.611473

(7) Dataset D_7

Number of	SN	SP	ACC	MCC

features				
4	0.487179	1	0.91453	0.664726
5	0.512821	1	0.918803	0.683586
6	0.564103	1	0.92735	0.720325
7	0.615385	1	0.935897	0.755929
8	0.615385	1	0.935897	0.755929
9	0.615385	0.989744	0.92735	0.717594
10	0.641026	0.984615	0.92735	0.718393
11	0.666667	0.984615	0.931624	0.736619
12	0.666667	0.979487	0.92735	0.720294
13	0.641026	0.984615	0.92735	0.718393
14	0.641026	0.984615	0.92735	0.718393
15	0.589744	0.989744	0.923077	0.699118
16	0.615385	0.984615	0.923077	0.699896
17	0.666667	0.979487	0.92735	0.720294
18	0.666667	0.979487	0.92735	0.720294
19	0.692308	0.984615	0.935897	0.754594
20	0.692308	0.979487	0.931624	0.738512
21	0.692308	0.984615	0.935897	0.754594
22	0.692308	0.984615	0.935897	0.754594
23	0.692308	0.984615	0.935897	0.754594
24	0.692308	0.984615	0.935897	0.754594
25	0.692308	0.984615	0.935897	0.754594
26	0.666667	0.984615	0.931624	0.736619
27	0.666667	0.984615	0.931624	0.736619
28	0.666667	0.984615	0.931624	0.736619
29	0.666667	0.979487	0.92735	0.720294
30	0.666667	0.979487	0.92735	0.720294
31	0.692308	0.989744	0.940171	0.77142
32	0.692308	0.989744	0.940171	0.77142
33	0.692308	0.984615	0.935897	0.754594
34	0.692308	0.989744	0.940171	0.77142
35	0.692308	0.989744	0.940171	0.77142
36	0.692308	0.984615	0.935897	0.754594
37	0.692308	0.984615	0.935897	0.754594
38	0.692308	0.984615	0.935897	0.754594
39	0.692308	0.984615	0.935897	0.754594
40	0.692308	0.984615	0.935897	0.754594
41	0.692308	0.984615	0.935897	0.754594
42	0.692308	0.984615	0.935897	0.754594
43	0.692308	0.984615	0.935897	0.754594
44	0.666667	0.984615	0.931624	0.736619
45	0.666667	0.984615	0.931624	0.736619

46	0.666667	0.984615	0.931624	0.736619
47	0.666667	0.984615	0.931624	0.736619
48	0.666667	0.984615	0.931624	0.736619
49	0.666667	0.979487	0.92735	0.720294
50	0.666667	0.979487	0.92735	0.720294
51	0.666667	0.979487	0.92735	0.720294
52	0.615385	0.979487	0.918803	0.683062
53	0.641026	0.979487	0.923077	0.701818
54	0.641026	0.979487	0.923077	0.701818
55	0.641026	0.979487	0.923077	0.701818
56	0.641026	0.979487	0.923077	0.701818
57	0.641026	0.979487	0.923077	0.701818
58	0.641026	0.979487	0.923077	0.701818
59	0.641026	0.979487	0.923077	0.701818
60	0.641026	0.979487	0.923077	0.701818
61	0.641026	0.979487	0.923077	0.701818
62	0.641026	0.979487	0.923077	0.701818
63	0.641026	0.979487	0.923077	0.701818
64	0.641026	0.979487	0.923077	0.701818
65	0.615385	0.974359	0.91453	0.667017
66	0.641026	0.974359	0.918803	0.685994
67	0.615385	0.974359	0.91453	0.667017
68	0.615385	0.969231	0.910256	0.651695
69	0.641026	0.969231	0.91453	0.670862
70	0.615385	0.969231	0.910256	0.651695
71	0.615385	0.969231	0.910256	0.651695
72	0.615385	0.969231	0.910256	0.651695
73	0.615385	0.969231	0.910256	0.651695
74	0.641026	0.969231	0.91453	0.670862
75	0.641026	0.974359	0.918803	0.685994
76	0.641026	0.974359	0.918803	0.685994
77	0.615385	0.979487	0.918803	0.683062
78	0.615385	0.974359	0.91453	0.667017
79	0.666667	0.974359	0.923077	0.704687
80	0.641026	0.974359	0.918803	0.685994
81	0.666667	0.979487	0.92735	0.720294
82	0.692308	0.979487	0.931624	0.738512
83	0.717949	0.974359	0.931624	0.7413
84	0.717949	0.979487	0.935897	0.75649
85	0.717949	0.979487	0.935897	0.75649
86	0.717949	0.979487	0.935897	0.75649
87	0.717949	0.979487	0.935897	0.75649
88	0.692308	0.979487	0.931624	0.738512

89	0.692308	0.974359	0.92735	0.723116
90	0.666667	0.974359	0.923077	0.704687
91	0.666667	0.969231	0.918803	0.689741
92	0.666667	0.969231	0.918803	0.689741
93	0.666667	0.964103	0.91453	0.675406
94	0.666667	0.969231	0.918803	0.689741
95	0.692308	0.974359	0.92735	0.723116
96	0.666667	0.969231	0.918803	0.689741
97	0.666667	0.969231	0.918803	0.689741
98	0.692308	0.969231	0.923077	0.708353
99	0.692308	0.974359	0.92735	0.723116
100	0.692308	0.974359	0.92735	0.723116
101	0.692308	0.979487	0.931624	0.738512
102	0.692308	0.979487	0.931624	0.738512
103	0.692308	0.979487	0.931624	0.738512
104	0.692308	0.979487	0.931624	0.738512
105	0.692308	0.974359	0.92735	0.723116
106	0.692308	0.979487	0.931624	0.738512
107	0.666667	0.974359	0.923077	0.704687
108	0.666667	0.969231	0.918803	0.689741
109	0.666667	0.969231	0.918803	0.689741
110	0.666667	0.969231	0.918803	0.689741
111	0.666667	0.969231	0.918803	0.689741
112	0.717949	0.969231	0.92735	0.726717
113	0.717949	0.969231	0.92735	0.726717
114	0.717949	0.969231	0.92735	0.726717
115	0.717949	0.969231	0.92735	0.726717
116	0.717949	0.974359	0.931624	0.7413
117	0.717949	0.974359	0.931624	0.7413
118	0.717949	0.974359	0.931624	0.7413
119	0.717949	0.974359	0.931624	0.7413
120	0.692308	0.974359	0.92735	0.723116
121	0.717949	0.974359	0.931624	0.7413
122	0.717949	0.974359	0.931624	0.7413
123	0.717949	0.974359	0.931624	0.7413
124	0.717949	0.974359	0.931624	0.7413
125	0.717949	0.974359	0.931624	0.7413
126	0.717949	0.979487	0.935897	0.75649
127	0.717949	0.984615	0.940171	0.772337
128	0.717949	0.984615	0.940171	0.772337
129	0.717949	0.984615	0.940171	0.772337
130	0.717949	0.984615	0.940171	0.772337
131	0.717949	0.984615	0.940171	0.772337

132	0.717949	0.984615	0.940171	0.772337
133	0.717949	0.984615	0.940171	0.772337
134	0.717949	0.984615	0.940171	0.772337
135	0.717949	0.984615	0.940171	0.772337
136	0.717949	0.989744	0.944444	0.788893
137	0.717949	0.989744	0.944444	0.788893
138	0.717949	0.989744	0.944444	0.788893
139	0.717949	0.989744	0.944444	0.788893
140	0.717949	0.989744	0.944444	0.788893
141	0.717949	0.989744	0.944444	0.788893
142	0.717949	0.989744	0.944444	0.788893
143	0.717949	0.989744	0.944444	0.788893
144	0.717949	0.989744	0.944444	0.788893
145	0.717949	0.989744	0.944444	0.788893
146	0.717949	0.989744	0.944444	0.788893
147	0.717949	0.989744	0.944444	0.788893
148	0.717949	0.989744	0.944444	0.788893
149	0.717949	0.989744	0.944444	0.788893
150	0.717949	0.989744	0.944444	0.788893
151	0.717949	0.989744	0.944444	0.788893
152	0.717949	0.989744	0.944444	0.788893
153	0.717949	0.989744	0.944444	0.788893
154	0.717949	0.989744	0.944444	0.788893
155	0.717949	0.984615	0.940171	0.772337
156	0.717949	0.979487	0.935897	0.75649
157	0.717949	0.979487	0.935897	0.75649
158	0.717949	0.979487	0.935897	0.75649
159	0.692308	0.979487	0.931624	0.738512
160	0.692308	0.979487	0.931624	0.738512
161	0.692308	0.979487	0.931624	0.738512
162	0.717949	0.979487	0.935897	0.75649
163	0.717949	0.984615	0.940171	0.772337
164	0.717949	0.984615	0.940171	0.772337
165	0.717949	0.979487	0.935897	0.75649
166	0.717949	0.979487	0.935897	0.75649
167	0.717949	0.984615	0.940171	0.772337
168	0.717949	0.984615	0.940171	0.772337
169	0.717949	0.984615	0.940171	0.772337
170	0.717949	0.984615	0.940171	0.772337
171	0.717949	0.984615	0.940171	0.772337
172	0.717949	0.984615	0.940171	0.772337
173	0.717949	0.984615	0.940171	0.772337
174	0.717949	0.984615	0.940171	0.772337

175	0.717949	0.984615	0.940171	0.772337
176	0.717949	0.984615	0.940171	0.772337
177	0.717949	0.984615	0.940171	0.772337
178	0.717949	0.989744	0.944444	0.788893
179	0.717949	0.984615	0.940171	0.772337
180	0.717949	0.984615	0.940171	0.772337
181	0.717949	0.984615	0.940171	0.772337
182	0.717949	0.989744	0.944444	0.788893
183	0.717949	0.989744	0.944444	0.788893
184	0.717949	0.989744	0.944444	0.788893
185	0.717949	0.984615	0.940171	0.772337
186	0.717949	0.984615	0.940171	0.772337
187	0.717949	0.979487	0.935897	0.75649
188	0.717949	0.984615	0.940171	0.772337
189	0.717949	0.974359	0.931624	0.7413
190	0.717949	0.974359	0.931624	0.7413
191	0.717949	0.979487	0.935897	0.75649
192	0.717949	0.984615	0.940171	0.772337
193	0.717949	0.984615	0.940171	0.772337
194	0.692308	0.974359	0.92735	0.723116
195	0.692308	0.974359	0.92735	0.723116
196	0.692308	0.974359	0.92735	0.723116
197	0.692308	0.974359	0.92735	0.723116
198	0.666667	0.974359	0.923077	0.704687
199	0.666667	0.974359	0.923077	0.704687
200	0.666667	0.974359	0.923077	0.704687
201	0.666667	0.974359	0.923077	0.704687
202	0.692308	0.969231	0.923077	0.708353
203	0.692308	0.969231	0.923077	0.708353
204	0.692308	0.969231	0.923077	0.708353
205	0.692308	0.969231	0.923077	0.708353
206	0.692308	0.969231	0.923077	0.708353
207	0.692308	0.969231	0.923077	0.708353
208	0.692308	0.969231	0.923077	0.708353
209	0.692308	0.969231	0.923077	0.708353
210	0.692308	0.969231	0.923077	0.708353
211	0.692308	0.969231	0.923077	0.708353
212	0.692308	0.974359	0.92735	0.723116
213	0.692308	0.974359	0.92735	0.723116
214	0.692308	0.974359	0.92735	0.723116
215	0.692308	0.974359	0.92735	0.723116
216	0.692308	0.974359	0.92735	0.723116
217	0.692308	0.974359	0.92735	0.723116

218	0.692308	0.974359	0.92735	0.723116
219	0.692308	0.974359	0.92735	0.723116
220	0.692308	0.974359	0.92735	0.723116
221	0.717949	0.974359	0.931624	0.7413
222	0.717949	0.974359	0.931624	0.7413
223	0.717949	0.974359	0.931624	0.7413
224	0.717949	0.974359	0.931624	0.7413
225	0.692308	0.974359	0.92735	0.723116
226	0.692308	0.974359	0.92735	0.723116
227	0.692308	0.979487	0.931624	0.738512
228	0.692308	0.979487	0.931624	0.738512
229	0.692308	0.984615	0.935897	0.754594
230	0.692308	0.979487	0.931624	0.738512
231	0.692308	0.979487	0.931624	0.738512
232	0.666667	0.984615	0.931624	0.736619
233	0.666667	0.984615	0.931624	0.736619
234	0.666667	0.979487	0.92735	0.720294
235	0.692308	0.979487	0.931624	0.738512
236	0.692308	0.979487	0.931624	0.738512
237	0.692308	0.979487	0.931624	0.738512
238	0.692308	0.984615	0.935897	0.754594
239	0.692308	0.984615	0.935897	0.754594
240	0.717949	0.984615	0.940171	0.772337
241	0.717949	0.984615	0.940171	0.772337
242	0.717949	0.984615	0.940171	0.772337
243	0.692308	0.984615	0.935897	0.754594
244	0.692308	0.984615	0.935897	0.754594
245	0.666667	0.974359	0.923077	0.704687
246	0.666667	0.974359	0.923077	0.704687
247	0.666667	0.974359	0.923077	0.704687
248	0.666667	0.974359	0.923077	0.704687
249	0.666667	0.974359	0.923077	0.704687
250	0.666667	0.969231	0.918803	0.689741
251	0.666667	0.974359	0.923077	0.704687
252	0.666667	0.974359	0.923077	0.704687
253	0.666667	0.974359	0.923077	0.704687
254	0.666667	0.974359	0.923077	0.704687
255	0.666667	0.974359	0.923077	0.704687
256	0.666667	0.974359	0.923077	0.704687
257	0.666667	0.974359	0.923077	0.704687
258	0.666667	0.974359	0.923077	0.704687
259	0.666667	0.974359	0.923077	0.704687
260	0.666667	0.974359	0.923077	0.704687

261	0.666667	0.974359	0.923077	0.704687
262	0.666667	0.974359	0.923077	0.704687
263	0.666667	0.974359	0.923077	0.704687
264	0.666667	0.974359	0.923077	0.704687
265	0.666667	0.974359	0.923077	0.704687
266	0.666667	0.974359	0.923077	0.704687
267	0.666667	0.974359	0.923077	0.704687
268	0.666667	0.974359	0.923077	0.704687
269	0.666667	0.974359	0.923077	0.704687
270	0.666667	0.974359	0.923077	0.704687
271	0.692308	0.974359	0.92735	0.723116
272	0.692308	0.974359	0.92735	0.723116
273	0.692308	0.979487	0.931624	0.738512
274	0.692308	0.979487	0.931624	0.738512
275	0.692308	0.979487	0.931624	0.738512
276	0.666667	0.979487	0.92735	0.720294
277	0.666667	0.979487	0.92735	0.720294
278	0.666667	0.979487	0.92735	0.720294
279	0.666667	0.984615	0.931624	0.736619
280	0.666667	0.984615	0.931624	0.736619
281	0.666667	0.984615	0.931624	0.736619
282	0.666667	0.984615	0.931624	0.736619
283	0.666667	0.984615	0.931624	0.736619
284	0.666667	0.984615	0.931624	0.736619
285	0.666667	0.979487	0.92735	0.720294
286	0.666667	0.979487	0.92735	0.720294
287	0.692308	0.979487	0.931624	0.738512
288	0.692308	0.979487	0.931624	0.738512
289	0.692308	0.979487	0.931624	0.738512
290	0.692308	0.979487	0.931624	0.738512
291	0.692308	0.979487	0.931624	0.738512
292	0.692308	0.984615	0.935897	0.754594
293	0.692308	0.984615	0.935897	0.754594
294	0.692308	0.984615	0.935897	0.754594
295	0.692308	0.984615	0.935897	0.754594
296	0.692308	0.984615	0.935897	0.754594
297	0.692308	0.979487	0.931624	0.738512
298	0.692308	0.979487	0.931624	0.738512
299	0.692308	0.979487	0.931624	0.738512
300	0.692308	0.979487	0.931624	0.738512
301	0.692308	0.979487	0.931624	0.738512
302	0.692308	0.979487	0.931624	0.738512
303	0.692308	0.979487	0.931624	0.738512

304	0.692308	0.974359	0.92735	0.723116
305	0.692308	0.974359	0.92735	0.723116
306	0.692308	0.974359	0.92735	0.723116
307	0.692308	0.974359	0.92735	0.723116
308	0.692308	0.974359	0.92735	0.723116
309	0.692308	0.974359	0.92735	0.723116
310	0.692308	0.974359	0.92735	0.723116
311	0.692308	0.974359	0.92735	0.723116
312	0.692308	0.979487	0.931624	0.738512
313	0.692308	0.979487	0.931624	0.738512
314	0.692308	0.979487	0.931624	0.738512
315	0.692308	0.979487	0.931624	0.738512
316	0.692308	0.979487	0.931624	0.738512
317	0.692308	0.979487	0.931624	0.738512
318	0.666667	0.969231	0.918803	0.689741
319	0.692308	0.984615	0.935897	0.754594
320	0.692308	0.979487	0.931624	0.738512
321	0.692308	0.979487	0.931624	0.738512
322	0.692308	0.979487	0.931624	0.738512
323	0.692308	0.979487	0.931624	0.738512
324	0.692308	0.984615	0.935897	0.754594
325	0.692308	0.984615	0.935897	0.754594
326	0.692308	0.984615	0.935897	0.754594
327	0.692308	0.984615	0.935897	0.754594
328	0.692308	0.984615	0.935897	0.754594
329	0.692308	0.984615	0.935897	0.754594
330	0.692308	0.984615	0.935897	0.754594
331	0.692308	0.984615	0.935897	0.754594
332	0.692308	0.984615	0.935897	0.754594
333	0.692308	0.984615	0.935897	0.754594
334	0.692308	0.979487	0.931624	0.738512
335	0.692308	0.979487	0.931624	0.738512
336	0.692308	0.979487	0.931624	0.738512
337	0.692308	0.979487	0.931624	0.738512
338	0.692308	0.979487	0.931624	0.738512
339	0.692308	0.979487	0.931624	0.738512
340	0.692308	0.979487	0.931624	0.738512
341	0.692308	0.979487	0.931624	0.738512
342	0.692308	0.979487	0.931624	0.738512
343	0.692308	0.979487	0.931624	0.738512
344	0.692308	0.979487	0.931624	0.738512
345	0.692308	0.979487	0.931624	0.738512
346	0.692308	0.979487	0.931624	0.738512

347	0.692308	0.979487	0.931624	0.738512
348	0.692308	0.979487	0.931624	0.738512
349	0.692308	0.979487	0.931624	0.738512
350	0.692308	0.979487	0.931624	0.738512
351	0.692308	0.979487	0.931624	0.738512
352	0.692308	0.979487	0.931624	0.738512
353	0.692308	0.979487	0.931624	0.738512
354	0.692308	0.979487	0.931624	0.738512
355	0.692308	0.979487	0.931624	0.738512
356	0.692308	0.979487	0.931624	0.738512
357	0.692308	0.979487	0.931624	0.738512
358	0.692308	0.979487	0.931624	0.738512
359	0.692308	0.984615	0.935897	0.754594
360	0.692308	0.984615	0.935897	0.754594
361	0.692308	0.984615	0.935897	0.754594
362	0.692308	0.984615	0.935897	0.754594
363	0.692308	0.984615	0.935897	0.754594
364	0.692308	0.989744	0.940171	0.77142
365	0.692308	0.989744	0.940171	0.77142
366	0.717949	0.989744	0.944444	0.788893
367	0.692308	0.989744	0.940171	0.77142
368	0.717949	0.989744	0.944444	0.788893
369	0.717949	0.989744	0.944444	0.788893
370	0.717949	0.989744	0.944444	0.788893
371	0.692308	0.989744	0.940171	0.77142
372	0.717949	0.989744	0.944444	0.788893
373	0.692308	0.989744	0.940171	0.77142
374	0.692308	0.989744	0.940171	0.77142
375	0.692308	0.984615	0.935897	0.754594
376	0.692308	0.984615	0.935897	0.754594
377	0.692308	0.984615	0.935897	0.754594
378	0.692308	0.984615	0.935897	0.754594
379	0.692308	0.984615	0.935897	0.754594
380	0.692308	0.984615	0.935897	0.754594
381	0.692308	0.984615	0.935897	0.754594
382	0.692308	0.979487	0.931624	0.738512
383	0.692308	0.979487	0.931624	0.738512
384	0.692308	0.979487	0.931624	0.738512
385	0.692308	0.984615	0.935897	0.754594
386	0.692308	0.984615	0.935897	0.754594
387	0.717949	0.989744	0.944444	0.788893
388	0.717949	0.989744	0.944444	0.788893
389	0.717949	0.989744	0.944444	0.788893

390	0.717949	0.989744	0.944444	0.788893
391	0.717949	0.989744	0.944444	0.788893
392	0.692308	0.989744	0.940171	0.77142
393	0.717949	0.989744	0.944444	0.788893
394	0.717949	0.989744	0.944444	0.788893
395	0.717949	0.989744	0.944444	0.788893
396	0.717949	0.984615	0.940171	0.772337
397	0.717949	0.984615	0.940171	0.772337
398	0.717949	0.984615	0.940171	0.772337
399	0.717949	0.984615	0.940171	0.772337
400	0.692308	0.974359	0.92735	0.723116
401	0.692308	0.974359	0.92735	0.723116
402	0.692308	0.974359	0.92735	0.723116
403	0.717949	0.974359	0.931624	0.7413
404	0.717949	0.974359	0.931624	0.7413
405	0.717949	0.974359	0.931624	0.7413
406	0.717949	0.974359	0.931624	0.7413
407	0.717949	0.974359	0.931624	0.7413
408	0.717949	0.974359	0.931624	0.7413
409	0.717949	0.974359	0.931624	0.7413
410	0.717949	0.974359	0.931624	0.7413
411	0.717949	0.974359	0.931624	0.7413
412	0.692308	0.974359	0.92735	0.723116
413	0.692308	0.974359	0.92735	0.723116
414	0.717949	0.974359	0.931624	0.7413
415	0.717949	0.974359	0.931624	0.7413
416	0.717949	0.974359	0.931624	0.7413
417	0.717949	0.974359	0.931624	0.7413
418	0.717949	0.974359	0.931624	0.7413
419	0.717949	0.974359	0.931624	0.7413
420	0.692308	0.974359	0.92735	0.723116
421	0.692308	0.974359	0.92735	0.723116
422	0.692308	0.974359	0.92735	0.723116
423	0.692308	0.974359	0.92735	0.723116
424	0.717949	0.974359	0.931624	0.7413
425	0.717949	0.974359	0.931624	0.7413
426	0.717949	0.969231	0.92735	0.726717
427	0.717949	0.969231	0.92735	0.726717
428	0.717949	0.969231	0.92735	0.726717
429	0.717949	0.969231	0.92735	0.726717
430	0.717949	0.969231	0.92735	0.726717
431	0.717949	0.969231	0.92735	0.726717
432	0.717949	0.969231	0.92735	0.726717

433	0.717949	0.969231	0.92735	0.726717
434	0.717949	0.969231	0.92735	0.726717
435	0.717949	0.969231	0.92735	0.726717
436	0.717949	0.969231	0.92735	0.726717
437	0.717949	0.969231	0.92735	0.726717
438	0.717949	0.969231	0.92735	0.726717
439	0.717949	0.969231	0.92735	0.726717
440	0.717949	0.969231	0.92735	0.726717
441	0.717949	0.969231	0.92735	0.726717
442	0.717949	0.969231	0.92735	0.726717
443	0.717949	0.969231	0.92735	0.726717
444	0.717949	0.974359	0.931624	0.7413
445	0.717949	0.974359	0.931624	0.7413
446	0.717949	0.969231	0.92735	0.726717
447	0.717949	0.969231	0.92735	0.726717
448	0.717949	0.969231	0.92735	0.726717
449	0.717949	0.974359	0.931624	0.7413
450	0.717949	0.974359	0.931624	0.7413
451	0.717949	0.969231	0.92735	0.726717
452	0.717949	0.969231	0.92735	0.726717
453	0.717949	0.969231	0.92735	0.726717
454	0.717949	0.969231	0.92735	0.726717
455	0.717949	0.969231	0.92735	0.726717
456	0.717949	0.969231	0.92735	0.726717
457	0.717949	0.969231	0.92735	0.726717
458	0.717949	0.969231	0.92735	0.726717
459	0.717949	0.969231	0.92735	0.726717
460	0.74359	0.974359	0.935897	0.759257
461	0.74359	0.974359	0.935897	0.759257
462	0.74359	0.974359	0.935897	0.759257
463	0.74359	0.979487	0.940171	0.774246
464	0.74359	0.979487	0.940171	0.774246
465	0.74359	0.979487	0.940171	0.774246
466	0.74359	0.979487	0.940171	0.774246
467	0.74359	0.979487	0.940171	0.774246
468	0.74359	0.979487	0.940171	0.774246
469	0.74359	0.979487	0.940171	0.774246
470	0.74359	0.974359	0.935897	0.759257
471	0.74359	0.979487	0.940171	0.774246
472	0.74359	0.979487	0.940171	0.774246
473	0.74359	0.974359	0.935897	0.759257
474	0.74359	0.974359	0.935897	0.759257
475	0.74359	0.974359	0.935897	0.759257

476	0.74359	0.974359	0.935897	0.759257
477	0.74359	0.974359	0.935897	0.759257
478	0.74359	0.974359	0.935897	0.759257
479	0.74359	0.974359	0.935897	0.759257
480	0.74359	0.974359	0.935897	0.759257
481	0.74359	0.974359	0.935897	0.759257
482	0.74359	0.974359	0.935897	0.759257
483	0.74359	0.974359	0.935897	0.759257
484	0.74359	0.974359	0.935897	0.759257
485	0.74359	0.974359	0.935897	0.759257
486	0.74359	0.974359	0.935897	0.759257
487	0.74359	0.974359	0.935897	0.759257
488	0.74359	0.974359	0.935897	0.759257
489	0.74359	0.974359	0.935897	0.759257
490	0.74359	0.974359	0.935897	0.759257
491	0.74359	0.974359	0.935897	0.759257
492	0.74359	0.974359	0.935897	0.759257
493	0.74359	0.974359	0.935897	0.759257
494	0.74359	0.974359	0.935897	0.759257
495	0.74359	0.974359	0.935897	0.759257
496	0.74359	0.979487	0.940171	0.774246
497	0.74359	0.974359	0.935897	0.759257
498	0.74359	0.974359	0.935897	0.759257
499	0.74359	0.974359	0.935897	0.759257
500	0.74359	0.974359	0.935897	0.759257

(8) Dataset D_8

Number of features	SN	SP	ACC	MCC
4	0.333333	0.974359	0.867521	0.430331
5	0.435897	0.974359	0.884615	0.523873
6	0.487179	0.969231	0.888889	0.550633
7	0.512821	0.964103	0.888889	0.556328
8	0.512821	0.964103	0.888889	0.556328
9	0.487179	0.969231	0.888889	0.550633
10	0.538462	0.974359	0.901709	0.60813
11	0.615385	0.979487	0.918803	0.683062
12	0.615385	0.979487	0.918803	0.683062
13	0.589744	0.979487	0.91453	0.664004
14	0.589744	0.979487	0.91453	0.664004
15	0.589744	0.979487	0.91453	0.664004
16	0.589744	0.979487	0.91453	0.664004
17	0.589744	0.979487	0.91453	0.664004

18	0.589744	0.979487	0.91453	0.664004
19	0.589744	0.974359	0.910256	0.647732
20	0.589744	0.969231	0.905983	0.632216
21	0.589744	0.969231	0.905983	0.632216
22	0.564103	0.969231	0.901709	0.612401
23	0.564103	0.969231	0.901709	0.612401
24	0.564103	0.974359	0.905983	0.628112
25	0.538462	0.974359	0.901709	0.60813
26	0.538462	0.969231	0.897436	0.59222
27	0.538462	0.969231	0.897436	0.59222
28	0.564103	0.969231	0.901709	0.612401
29	0.538462	0.969231	0.897436	0.59222
30	0.564103	0.969231	0.901709	0.612401
31	0.564103	0.969231	0.901709	0.612401
32	0.538462	0.964103	0.893162	0.57707
33	0.538462	0.964103	0.893162	0.57707
34	0.538462	0.964103	0.893162	0.57707
35	0.564103	0.964103	0.897436	0.597415
36	0.589744	0.964103	0.901709	0.617395
37	0.589744	0.964103	0.901709	0.617395
38	0.564103	0.964103	0.897436	0.597415
39	0.589744	0.964103	0.901709	0.617395
40	0.589744	0.964103	0.901709	0.617395
41	0.564103	0.964103	0.897436	0.597415
42	0.589744	0.969231	0.905983	0.632216
43	0.589744	0.969231	0.905983	0.632216
44	0.615385	0.974359	0.91453	0.667017
45	0.615385	0.974359	0.91453	0.667017
46	0.615385	0.974359	0.91453	0.667017
47	0.615385	0.974359	0.91453	0.667017
48	0.615385	0.969231	0.910256	0.651695
49	0.615385	0.969231	0.910256	0.651695
50	0.615385	0.974359	0.91453	0.667017
51	0.589744	0.974359	0.910256	0.647732
52	0.589744	0.974359	0.910256	0.647732
53	0.589744	0.974359	0.910256	0.647732
54	0.589744	0.974359	0.910256	0.647732
55	0.589744	0.974359	0.910256	0.647732
56	0.589744	0.974359	0.910256	0.647732
57	0.589744	0.974359	0.910256	0.647732
58	0.589744	0.974359	0.910256	0.647732
59	0.589744	0.969231	0.905983	0.632216
60	0.589744	0.974359	0.910256	0.647732

61	0.589744	0.969231	0.905983	0.632216
62	0.589744	0.979487	0.91453	0.664004
63	0.589744	0.974359	0.910256	0.647732
64	0.589744	0.974359	0.910256	0.647732
65	0.564103	0.979487	0.910256	0.644618
66	0.564103	0.979487	0.910256	0.644618
67	0.564103	0.979487	0.910256	0.644618
68	0.564103	0.979487	0.910256	0.644618
69	0.564103	0.974359	0.905983	0.628112
70	0.564103	0.974359	0.905983	0.628112
71	0.589744	0.984615	0.918803	0.681106
72	0.589744	0.984615	0.918803	0.681106
73	0.615385	0.984615	0.923077	0.699896
74	0.641026	0.984615	0.92735	0.718393
75	0.615385	0.984615	0.923077	0.699896
76	0.615385	0.984615	0.923077	0.699896
77	0.615385	0.984615	0.923077	0.699896
78	0.615385	0.984615	0.923077	0.699896
79	0.641026	0.984615	0.92735	0.718393
80	0.615385	0.984615	0.923077	0.699896
81	0.666667	0.979487	0.92735	0.720294
82	0.641026	0.979487	0.923077	0.701818
83	0.641026	0.979487	0.923077	0.701818
84	0.641026	0.974359	0.918803	0.685994
85	0.641026	0.974359	0.918803	0.685994
86	0.641026	0.974359	0.918803	0.685994
87	0.641026	0.979487	0.923077	0.701818
88	0.692308	0.979487	0.931624	0.738512
89	0.692308	0.979487	0.931624	0.738512
90	0.692308	0.979487	0.931624	0.738512
91	0.692308	0.979487	0.931624	0.738512
92	0.666667	0.979487	0.92735	0.720294
93	0.666667	0.974359	0.923077	0.704687
94	0.641026	0.979487	0.923077	0.701818
95	0.641026	0.979487	0.923077	0.701818
96	0.641026	0.979487	0.923077	0.701818
97	0.641026	0.979487	0.923077	0.701818
98	0.641026	0.979487	0.923077	0.701818
99	0.641026	0.979487	0.923077	0.701818
100	0.641026	0.979487	0.923077	0.701818
101	0.641026	0.979487	0.923077	0.701818
102	0.666667	0.989744	0.935897	0.753724
103	0.666667	0.989744	0.935897	0.753724

104	0.666667	0.994872	0.940171	0.771681
105	0.666667	0.994872	0.940171	0.771681
106	0.692308	0.994872	0.944444	0.789055
107	0.666667	0.989744	0.935897	0.753724
108	0.692308	0.984615	0.935897	0.754594
109	0.692308	0.984615	0.935897	0.754594
110	0.692308	0.979487	0.931624	0.738512
111	0.692308	0.974359	0.92735	0.723116
112	0.692308	0.979487	0.931624	0.738512
113	0.692308	0.984615	0.935897	0.754594
114	0.692308	0.979487	0.931624	0.738512
115	0.666667	0.979487	0.92735	0.720294
116	0.666667	0.979487	0.92735	0.720294
117	0.641026	0.979487	0.923077	0.701818
118	0.641026	0.979487	0.923077	0.701818
119	0.641026	0.979487	0.923077	0.701818
120	0.641026	0.979487	0.923077	0.701818
121	0.615385	0.974359	0.91453	0.667017
122	0.615385	0.969231	0.910256	0.651695
123	0.641026	0.964103	0.910256	0.656366
124	0.641026	0.964103	0.910256	0.656366
125	0.641026	0.964103	0.910256	0.656366
126	0.641026	0.964103	0.910256	0.656366
127	0.641026	0.969231	0.91453	0.670862
128	0.641026	0.969231	0.91453	0.670862
129	0.666667	0.969231	0.918803	0.689741
130	0.666667	0.969231	0.918803	0.689741
131	0.615385	0.969231	0.910256	0.651695
132	0.641026	0.969231	0.91453	0.670862
133	0.615385	0.964103	0.905983	0.637037
134	0.641026	0.964103	0.910256	0.656366
135	0.641026	0.964103	0.910256	0.656366
136	0.666667	0.979487	0.92735	0.720294
137	0.666667	0.979487	0.92735	0.720294
138	0.666667	0.974359	0.923077	0.704687
139	0.666667	0.969231	0.918803	0.689741
140	0.641026	0.964103	0.910256	0.656366
141	0.641026	0.964103	0.910256	0.656366
142	0.641026	0.964103	0.910256	0.656366
143	0.641026	0.964103	0.910256	0.656366
144	0.641026	0.964103	0.910256	0.656366
145	0.641026	0.964103	0.910256	0.656366
146	0.641026	0.974359	0.918803	0.685994

147	0.641026	0.969231	0.91453	0.670862
148	0.641026	0.964103	0.910256	0.656366
149	0.641026	0.969231	0.91453	0.670862
150	0.641026	0.964103	0.910256	0.656366
151	0.641026	0.964103	0.910256	0.656366
152	0.641026	0.964103	0.910256	0.656366
153	0.641026	0.964103	0.910256	0.656366
154	0.641026	0.964103	0.910256	0.656366
155	0.615385	0.984615	0.923077	0.699896
156	0.615385	0.979487	0.918803	0.683062
157	0.615385	0.984615	0.923077	0.699896
158	0.615385	0.984615	0.923077	0.699896
159	0.615385	0.984615	0.923077	0.699896
160	0.641026	0.984615	0.92735	0.718393
161	0.615385	0.984615	0.923077	0.699896
162	0.615385	0.984615	0.923077	0.699896
163	0.641026	0.979487	0.923077	0.701818
164	0.641026	0.979487	0.923077	0.701818
165	0.641026	0.979487	0.923077	0.701818
166	0.641026	0.974359	0.918803	0.685994
167	0.641026	0.979487	0.923077	0.701818
168	0.641026	0.979487	0.923077	0.701818
169	0.641026	0.979487	0.923077	0.701818
170	0.692308	0.979487	0.931624	0.738512
171	0.692308	0.979487	0.931624	0.738512
172	0.692308	0.979487	0.931624	0.738512
173	0.692308	0.974359	0.92735	0.723116
174	0.692308	0.974359	0.92735	0.723116
175	0.692308	0.974359	0.92735	0.723116
176	0.692308	0.979487	0.931624	0.738512
177	0.692308	0.979487	0.931624	0.738512
178	0.692308	0.969231	0.923077	0.708353
179	0.692308	0.979487	0.931624	0.738512
180	0.692308	0.979487	0.931624	0.738512
181	0.692308	0.979487	0.931624	0.738512
182	0.641026	0.979487	0.923077	0.701818
183	0.641026	0.979487	0.923077	0.701818
184	0.641026	0.984615	0.92735	0.718393
185	0.641026	0.979487	0.923077	0.701818
186	0.641026	0.979487	0.923077	0.701818
187	0.641026	0.974359	0.918803	0.685994
188	0.641026	0.979487	0.923077	0.701818
189	0.641026	0.974359	0.918803	0.685994

190	0.641026	0.974359	0.918803	0.685994
191	0.641026	0.974359	0.918803	0.685994
192	0.641026	0.974359	0.918803	0.685994
193	0.641026	0.974359	0.918803	0.685994
194	0.641026	0.974359	0.918803	0.685994
195	0.641026	0.974359	0.918803	0.685994
196	0.641026	0.974359	0.918803	0.685994
197	0.641026	0.974359	0.918803	0.685994
198	0.641026	0.974359	0.918803	0.685994
199	0.641026	0.974359	0.918803	0.685994
200	0.641026	0.974359	0.918803	0.685994
201	0.641026	0.974359	0.918803	0.685994
202	0.641026	0.969231	0.91453	0.670862
203	0.641026	0.969231	0.91453	0.670862
204	0.641026	0.969231	0.91453	0.670862
205	0.641026	0.969231	0.91453	0.670862
206	0.641026	0.969231	0.91453	0.670862
207	0.641026	0.969231	0.91453	0.670862
208	0.666667	0.969231	0.918803	0.689741
209	0.666667	0.969231	0.918803	0.689741
210	0.666667	0.969231	0.918803	0.689741
211	0.666667	0.969231	0.918803	0.689741
212	0.666667	0.969231	0.918803	0.689741
213	0.666667	0.969231	0.918803	0.689741
214	0.666667	0.969231	0.918803	0.689741
215	0.666667	0.969231	0.918803	0.689741
216	0.666667	0.969231	0.918803	0.689741
217	0.666667	0.969231	0.918803	0.689741
218	0.666667	0.969231	0.918803	0.689741
219	0.666667	0.969231	0.918803	0.689741
220	0.666667	0.969231	0.918803	0.689741
221	0.666667	0.969231	0.918803	0.689741
222	0.666667	0.969231	0.918803	0.689741
223	0.666667	0.969231	0.918803	0.689741
224	0.666667	0.969231	0.918803	0.689741
225	0.666667	0.969231	0.918803	0.689741
226	0.666667	0.969231	0.918803	0.689741
227	0.641026	0.969231	0.91453	0.670862
228	0.641026	0.969231	0.91453	0.670862
229	0.641026	0.969231	0.91453	0.670862
230	0.641026	0.969231	0.91453	0.670862
231	0.641026	0.969231	0.91453	0.670862
232	0.641026	0.969231	0.91453	0.670862

233	0.641026	0.969231	0.91453	0.670862
234	0.641026	0.969231	0.91453	0.670862
235	0.641026	0.969231	0.91453	0.670862
236	0.641026	0.969231	0.91453	0.670862
237	0.641026	0.969231	0.91453	0.670862
238	0.641026	0.969231	0.91453	0.670862
239	0.641026	0.969231	0.91453	0.670862
240	0.641026	0.969231	0.91453	0.670862
241	0.641026	0.969231	0.91453	0.670862
242	0.641026	0.969231	0.91453	0.670862
243	0.641026	0.969231	0.91453	0.670862
244	0.641026	0.969231	0.91453	0.670862
245	0.641026	0.969231	0.91453	0.670862
246	0.641026	0.969231	0.91453	0.670862
247	0.641026	0.964103	0.910256	0.656366
248	0.641026	0.964103	0.910256	0.656366
249	0.641026	0.964103	0.910256	0.656366
250	0.641026	0.964103	0.910256	0.656366
251	0.692308	0.969231	0.923077	0.708353
252	0.692308	0.969231	0.923077	0.708353
253	0.692308	0.969231	0.923077	0.708353
254	0.692308	0.974359	0.92735	0.723116
255	0.692308	0.974359	0.92735	0.723116
256	0.692308	0.974359	0.92735	0.723116
257	0.692308	0.974359	0.92735	0.723116
258	0.692308	0.974359	0.92735	0.723116
259	0.692308	0.974359	0.92735	0.723116
260	0.692308	0.974359	0.92735	0.723116
261	0.692308	0.974359	0.92735	0.723116
262	0.692308	0.974359	0.92735	0.723116
263	0.692308	0.974359	0.92735	0.723116
264	0.692308	0.974359	0.92735	0.723116
265	0.692308	0.974359	0.92735	0.723116
266	0.692308	0.974359	0.92735	0.723116
267	0.692308	0.974359	0.92735	0.723116
268	0.692308	0.974359	0.92735	0.723116
269	0.692308	0.979487	0.931624	0.738512
270	0.692308	0.974359	0.92735	0.723116
271	0.692308	0.974359	0.92735	0.723116
272	0.692308	0.974359	0.92735	0.723116
273	0.692308	0.974359	0.92735	0.723116
274	0.692308	0.974359	0.92735	0.723116
275	0.692308	0.974359	0.92735	0.723116

276	0.692308	0.974359	0.92735	0.723116
277	0.666667	0.974359	0.923077	0.704687
278	0.666667	0.974359	0.923077	0.704687
279	0.666667	0.974359	0.923077	0.704687
280	0.666667	0.974359	0.923077	0.704687
281	0.666667	0.974359	0.923077	0.704687
282	0.666667	0.974359	0.923077	0.704687
283	0.666667	0.974359	0.923077	0.704687
284	0.666667	0.974359	0.923077	0.704687
285	0.666667	0.974359	0.923077	0.704687
286	0.666667	0.974359	0.923077	0.704687
287	0.666667	0.974359	0.923077	0.704687
288	0.692308	0.979487	0.931624	0.738512
289	0.692308	0.979487	0.931624	0.738512
290	0.692308	0.979487	0.931624	0.738512
291	0.692308	0.979487	0.931624	0.738512
292	0.692308	0.979487	0.931624	0.738512
293	0.692308	0.979487	0.931624	0.738512
294	0.666667	0.979487	0.92735	0.720294
295	0.666667	0.979487	0.92735	0.720294
296	0.666667	0.979487	0.92735	0.720294
297	0.666667	0.979487	0.92735	0.720294
298	0.666667	0.979487	0.92735	0.720294
299	0.666667	0.979487	0.92735	0.720294
300	0.692308	0.979487	0.931624	0.738512
301	0.692308	0.979487	0.931624	0.738512
302	0.692308	0.979487	0.931624	0.738512
303	0.666667	0.979487	0.92735	0.720294
304	0.717949	0.974359	0.931624	0.7413
305	0.717949	0.974359	0.931624	0.7413
306	0.717949	0.974359	0.931624	0.7413
307	0.717949	0.974359	0.931624	0.7413
308	0.717949	0.974359	0.931624	0.7413
309	0.717949	0.974359	0.931624	0.7413
310	0.717949	0.974359	0.931624	0.7413
311	0.717949	0.974359	0.931624	0.7413
312	0.717949	0.974359	0.931624	0.7413
313	0.717949	0.974359	0.931624	0.7413
314	0.717949	0.974359	0.931624	0.7413
315	0.717949	0.974359	0.931624	0.7413
316	0.717949	0.979487	0.935897	0.75649
317	0.717949	0.979487	0.935897	0.75649
318	0.717949	0.979487	0.935897	0.75649

319	0.717949	0.974359	0.931624	0.7413
320	0.717949	0.974359	0.931624	0.7413
321	0.717949	0.974359	0.931624	0.7413
322	0.717949	0.974359	0.931624	0.7413
323	0.717949	0.974359	0.931624	0.7413
324	0.717949	0.974359	0.931624	0.7413
325	0.717949	0.974359	0.931624	0.7413
326	0.717949	0.974359	0.931624	0.7413
327	0.717949	0.974359	0.931624	0.7413
328	0.717949	0.974359	0.931624	0.7413
329	0.717949	0.969231	0.92735	0.726717
330	0.717949	0.969231	0.92735	0.726717
331	0.717949	0.974359	0.931624	0.7413
332	0.717949	0.974359	0.931624	0.7413
333	0.717949	0.974359	0.931624	0.7413
334	0.717949	0.974359	0.931624	0.7413
335	0.717949	0.969231	0.92735	0.726717
336	0.717949	0.974359	0.931624	0.7413
337	0.717949	0.974359	0.931624	0.7413
338	0.717949	0.974359	0.931624	0.7413
339	0.717949	0.974359	0.931624	0.7413
340	0.717949	0.974359	0.931624	0.7413
341	0.717949	0.974359	0.931624	0.7413
342	0.717949	0.974359	0.931624	0.7413
343	0.717949	0.974359	0.931624	0.7413
344	0.717949	0.974359	0.931624	0.7413
345	0.717949	0.979487	0.935897	0.75649
346	0.717949	0.979487	0.935897	0.75649
347	0.717949	0.979487	0.935897	0.75649
348	0.717949	0.979487	0.935897	0.75649
349	0.717949	0.979487	0.935897	0.75649
350	0.717949	0.979487	0.935897	0.75649
351	0.717949	0.979487	0.935897	0.75649
352	0.717949	0.979487	0.935897	0.75649
353	0.717949	0.984615	0.940171	0.772337
354	0.717949	0.979487	0.935897	0.75649
355	0.717949	0.974359	0.931624	0.7413
356	0.717949	0.974359	0.931624	0.7413
357	0.717949	0.974359	0.931624	0.7413
358	0.717949	0.974359	0.931624	0.7413
359	0.717949	0.979487	0.935897	0.75649
360	0.717949	0.979487	0.935897	0.75649
361	0.717949	0.979487	0.935897	0.75649

362	0.717949	0.979487	0.935897	0.75649
363	0.717949	0.979487	0.935897	0.75649
364	0.717949	0.979487	0.935897	0.75649
365	0.717949	0.979487	0.935897	0.75649
366	0.717949	0.979487	0.935897	0.75649
367	0.717949	0.979487	0.935897	0.75649
368	0.717949	0.979487	0.935897	0.75649
369	0.717949	0.979487	0.935897	0.75649
370	0.717949	0.979487	0.935897	0.75649
371	0.717949	0.979487	0.935897	0.75649
372	0.717949	0.979487	0.935897	0.75649
373	0.717949	0.979487	0.935897	0.75649
374	0.717949	0.979487	0.935897	0.75649
375	0.717949	0.979487	0.935897	0.75649
376	0.717949	0.979487	0.935897	0.75649
377	0.717949	0.979487	0.935897	0.75649
378	0.717949	0.979487	0.935897	0.75649
379	0.717949	0.979487	0.935897	0.75649
380	0.717949	0.979487	0.935897	0.75649
381	0.717949	0.979487	0.935897	0.75649
382	0.717949	0.979487	0.935897	0.75649
383	0.717949	0.979487	0.935897	0.75649
384	0.717949	0.979487	0.935897	0.75649
385	0.717949	0.979487	0.935897	0.75649
386	0.717949	0.974359	0.931624	0.7413
387	0.717949	0.979487	0.935897	0.75649
388	0.692308	0.979487	0.931624	0.738512
389	0.692308	0.979487	0.931624	0.738512
390	0.692308	0.979487	0.931624	0.738512
391	0.692308	0.969231	0.923077	0.708353
392	0.692308	0.969231	0.923077	0.708353
393	0.692308	0.969231	0.923077	0.708353
394	0.692308	0.969231	0.923077	0.708353
395	0.692308	0.969231	0.923077	0.708353
396	0.692308	0.969231	0.923077	0.708353
397	0.692308	0.969231	0.923077	0.708353
398	0.692308	0.969231	0.923077	0.708353
399	0.692308	0.969231	0.923077	0.708353
400	0.692308	0.969231	0.923077	0.708353
401	0.692308	0.969231	0.923077	0.708353
402	0.692308	0.969231	0.923077	0.708353
403	0.692308	0.969231	0.923077	0.708353
404	0.666667	0.969231	0.918803	0.689741

405	0.666667	0.969231	0.918803	0.689741
406	0.666667	0.969231	0.918803	0.689741
407	0.666667	0.969231	0.918803	0.689741
408	0.666667	0.969231	0.918803	0.689741
409	0.666667	0.969231	0.918803	0.689741
410	0.666667	0.969231	0.918803	0.689741
411	0.666667	0.969231	0.918803	0.689741
412	0.666667	0.974359	0.923077	0.704687
413	0.666667	0.974359	0.923077	0.704687
414	0.666667	0.974359	0.923077	0.704687
415	0.666667	0.974359	0.923077	0.704687
416	0.666667	0.974359	0.923077	0.704687
417	0.666667	0.974359	0.923077	0.704687
418	0.666667	0.974359	0.923077	0.704687
419	0.666667	0.974359	0.923077	0.704687
420	0.666667	0.974359	0.923077	0.704687
421	0.666667	0.974359	0.923077	0.704687
422	0.666667	0.974359	0.923077	0.704687
423	0.666667	0.979487	0.92735	0.720294
424	0.666667	0.979487	0.92735	0.720294
425	0.666667	0.979487	0.92735	0.720294
426	0.666667	0.974359	0.923077	0.704687
427	0.666667	0.974359	0.923077	0.704687
428	0.666667	0.974359	0.923077	0.704687
429	0.666667	0.979487	0.92735	0.720294
430	0.641026	0.969231	0.91453	0.670862
431	0.641026	0.969231	0.91453	0.670862
432	0.641026	0.969231	0.91453	0.670862
433	0.641026	0.974359	0.918803	0.685994
434	0.666667	0.974359	0.923077	0.704687
435	0.666667	0.974359	0.923077	0.704687
436	0.666667	0.974359	0.923077	0.704687
437	0.692308	0.974359	0.92735	0.723116
438	0.692308	0.974359	0.92735	0.723116
439	0.692308	0.974359	0.92735	0.723116
440	0.692308	0.974359	0.92735	0.723116
441	0.692308	0.974359	0.92735	0.723116
442	0.692308	0.974359	0.92735	0.723116
443	0.692308	0.974359	0.92735	0.723116
444	0.692308	0.974359	0.92735	0.723116
445	0.692308	0.974359	0.92735	0.723116
446	0.692308	0.974359	0.92735	0.723116
447	0.692308	0.974359	0.92735	0.723116

448	0.692308	0.974359	0.92735	0.723116
449	0.692308	0.974359	0.92735	0.723116
450	0.692308	0.974359	0.92735	0.723116
451	0.692308	0.974359	0.92735	0.723116
452	0.692308	0.974359	0.92735	0.723116
453	0.692308	0.974359	0.92735	0.723116
454	0.692308	0.974359	0.92735	0.723116
455	0.692308	0.974359	0.92735	0.723116
456	0.692308	0.974359	0.92735	0.723116
457	0.692308	0.974359	0.92735	0.723116
458	0.74359	0.979487	0.940171	0.774246
459	0.74359	0.979487	0.940171	0.774246
460	0.74359	0.979487	0.940171	0.774246
461	0.74359	0.979487	0.940171	0.774246
462	0.74359	0.984615	0.944444	0.789865
463	0.74359	0.984615	0.944444	0.789865
464	0.74359	0.984615	0.944444	0.789865
465	0.74359	0.984615	0.944444	0.789865
466	0.74359	0.984615	0.944444	0.789865
467	0.74359	0.984615	0.944444	0.789865
468	0.74359	0.984615	0.944444	0.789865
469	0.74359	0.984615	0.944444	0.789865
470	0.74359	0.984615	0.944444	0.789865
471	0.717949	0.984615	0.940171	0.772337
472	0.717949	0.984615	0.940171	0.772337
473	0.717949	0.984615	0.940171	0.772337
474	0.717949	0.984615	0.940171	0.772337
475	0.717949	0.984615	0.940171	0.772337
476	0.717949	0.984615	0.940171	0.772337
477	0.717949	0.984615	0.940171	0.772337
478	0.717949	0.984615	0.940171	0.772337
479	0.717949	0.984615	0.940171	0.772337
480	0.717949	0.984615	0.940171	0.772337
481	0.717949	0.984615	0.940171	0.772337
482	0.717949	0.984615	0.940171	0.772337
483	0.717949	0.984615	0.940171	0.772337
484	0.717949	0.984615	0.940171	0.772337
485	0.717949	0.984615	0.940171	0.772337
486	0.717949	0.984615	0.940171	0.772337
487	0.717949	0.984615	0.940171	0.772337
488	0.717949	0.984615	0.940171	0.772337
489	0.717949	0.984615	0.940171	0.772337
490	0.717949	0.984615	0.940171	0.772337

491	0.717949	0.984615	0.940171	0.772337
492	0.717949	0.984615	0.940171	0.772337
493	0.717949	0.984615	0.940171	0.772337
494	0.717949	0.984615	0.940171	0.772337
495	0.692308	0.984615	0.935897	0.754594
496	0.692308	0.984615	0.935897	0.754594
497	0.692308	0.984615	0.935897	0.754594
498	0.692308	0.984615	0.935897	0.754594
499	0.692308	0.984615	0.935897	0.754594
500	0.692308	0.984615	0.935897	0.754594

(9) Dataset D_9

Number of features	SN	SP	ACC	MCC
4	0.589744	0.964103	0.901709	0.617395
5	0.589744	0.958974	0.897436	0.603212
6	0.589744	0.964103	0.901709	0.617395
7	0.589744	0.974359	0.910256	0.647732
8	0.589744	0.964103	0.901709	0.617395
9	0.564103	0.979487	0.910256	0.644618
10	0.589744	0.974359	0.910256	0.647732
11	0.564103	0.969231	0.901709	0.612401
12	0.615385	0.974359	0.91453	0.667017
13	0.589744	0.974359	0.910256	0.647732
14	0.538462	0.969231	0.897436	0.59222
15	0.538462	0.974359	0.901709	0.60813
16	0.564103	0.979487	0.910256	0.644618
17	0.615385	0.974359	0.91453	0.667017
18	0.589744	0.979487	0.91453	0.664004
19	0.589744	0.979487	0.91453	0.664004
20	0.589744	0.974359	0.910256	0.647732
21	0.589744	0.974359	0.910256	0.647732
22	0.589744	0.964103	0.901709	0.617395
23	0.589744	0.964103	0.901709	0.617395
24	0.641026	0.969231	0.91453	0.670862
25	0.641026	0.969231	0.91453	0.670862
26	0.641026	0.974359	0.918803	0.685994
27	0.615385	0.974359	0.91453	0.667017
28	0.589744	0.979487	0.91453	0.664004
29	0.589744	0.979487	0.91453	0.664004
30	0.589744	0.974359	0.910256	0.647732
31	0.589744	0.964103	0.901709	0.617395
32	0.589744	0.969231	0.905983	0.632216

33	0.564103	0.974359	0.905983	0.628112
34	0.564103	0.979487	0.910256	0.644618
35	0.589744	0.964103	0.901709	0.617395
36	0.564103	0.964103	0.897436	0.597415
37	0.564103	0.958974	0.893162	0.583095
38	0.589744	0.964103	0.901709	0.617395
39	0.589744	0.964103	0.901709	0.617395
40	0.589744	0.964103	0.901709	0.617395
41	0.589744	0.958974	0.897436	0.603212
42	0.589744	0.958974	0.897436	0.603212
43	0.589744	0.958974	0.897436	0.603212
44	0.589744	0.958974	0.897436	0.603212
45	0.589744	0.964103	0.901709	0.617395
46	0.589744	0.969231	0.905983	0.632216
47	0.589744	0.974359	0.910256	0.647732
48	0.589744	0.974359	0.910256	0.647732
49	0.589744	0.974359	0.910256	0.647732
50	0.589744	0.974359	0.910256	0.647732
51	0.589744	0.974359	0.910256	0.647732
52	0.589744	0.974359	0.910256	0.647732
53	0.589744	0.974359	0.910256	0.647732
54	0.641026	0.974359	0.918803	0.685994
55	0.666667	0.974359	0.923077	0.704687
56	0.641026	0.979487	0.923077	0.701818
57	0.641026	0.979487	0.923077	0.701818
58	0.641026	0.979487	0.923077	0.701818
59	0.615385	0.979487	0.918803	0.683062
60	0.615385	0.979487	0.918803	0.683062
61	0.615385	0.974359	0.91453	0.667017
62	0.615385	0.974359	0.91453	0.667017
63	0.589744	0.974359	0.910256	0.647732
64	0.589744	0.969231	0.905983	0.632216
65	0.589744	0.974359	0.910256	0.647732
66	0.589744	0.969231	0.905983	0.632216
67	0.641026	0.969231	0.91453	0.670862
68	0.615385	0.969231	0.910256	0.651695
69	0.641026	0.969231	0.91453	0.670862
70	0.589744	0.964103	0.901709	0.617395
71	0.589744	0.964103	0.901709	0.617395
72	0.589744	0.964103	0.901709	0.617395
73	0.589744	0.964103	0.901709	0.617395
74	0.589744	0.964103	0.901709	0.617395
75	0.615385	0.964103	0.905983	0.637037

76	0.615385	0.958974	0.901709	0.622992
77	0.615385	0.958974	0.901709	0.622992
78	0.615385	0.958974	0.901709	0.622992
79	0.615385	0.958974	0.901709	0.622992
80	0.615385	0.958974	0.901709	0.622992
81	0.615385	0.958974	0.901709	0.622992
82	0.564103	0.958974	0.893162	0.583095
83	0.589744	0.958974	0.897436	0.603212
84	0.589744	0.958974	0.897436	0.603212
85	0.564103	0.964103	0.897436	0.597415
86	0.564103	0.964103	0.897436	0.597415
87	0.538462	0.964103	0.893162	0.57707
88	0.538462	0.958974	0.888889	0.562614
89	0.538462	0.958974	0.888889	0.562614
90	0.538462	0.958974	0.888889	0.562614
91	0.538462	0.958974	0.888889	0.562614
92	0.538462	0.964103	0.893162	0.57707
93	0.538462	0.958974	0.888889	0.562614
94	0.538462	0.958974	0.888889	0.562614
95	0.564103	0.958974	0.893162	0.583095
96	0.538462	0.958974	0.888889	0.562614
97	0.564103	0.958974	0.893162	0.583095
98	0.538462	0.958974	0.888889	0.562614
99	0.564103	0.958974	0.893162	0.583095
100	0.538462	0.958974	0.888889	0.562614
101	0.538462	0.958974	0.888889	0.562614
102	0.538462	0.958974	0.888889	0.562614
103	0.564103	0.958974	0.893162	0.583095
104	0.564103	0.958974	0.893162	0.583095
105	0.538462	0.958974	0.888889	0.562614
106	0.589744	0.958974	0.897436	0.603212
107	0.615385	0.964103	0.905983	0.637037
108	0.615385	0.964103	0.905983	0.637037
109	0.615385	0.964103	0.905983	0.637037
110	0.615385	0.964103	0.905983	0.637037
111	0.615385	0.964103	0.905983	0.637037
112	0.615385	0.958974	0.901709	0.622992
113	0.589744	0.958974	0.897436	0.603212
114	0.589744	0.958974	0.897436	0.603212
115	0.589744	0.958974	0.897436	0.603212
116	0.589744	0.958974	0.897436	0.603212
117	0.589744	0.958974	0.897436	0.603212
118	0.615385	0.958974	0.901709	0.622992

119	0.615385	0.958974	0.901709	0.622992
120	0.615385	0.958974	0.901709	0.622992
121	0.589744	0.958974	0.897436	0.603212
122	0.589744	0.958974	0.897436	0.603212
123	0.615385	0.958974	0.901709	0.622992
124	0.589744	0.958974	0.897436	0.603212
125	0.641026	0.958974	0.905983	0.64246
126	0.615385	0.958974	0.901709	0.622992
127	0.641026	0.953846	0.901709	0.629098
128	0.641026	0.958974	0.905983	0.64246
129	0.641026	0.958974	0.905983	0.64246
130	0.641026	0.958974	0.905983	0.64246
131	0.615385	0.958974	0.901709	0.622992
132	0.615385	0.958974	0.901709	0.622992
133	0.615385	0.958974	0.901709	0.622992
134	0.615385	0.958974	0.901709	0.622992
135	0.641026	0.958974	0.905983	0.64246
136	0.615385	0.964103	0.905983	0.637037
137	0.641026	0.964103	0.910256	0.656366
138	0.641026	0.964103	0.910256	0.656366
139	0.615385	0.958974	0.901709	0.622992
140	0.615385	0.958974	0.901709	0.622992
141	0.641026	0.958974	0.905983	0.64246
142	0.615385	0.958974	0.901709	0.622992
143	0.641026	0.958974	0.905983	0.64246
144	0.615385	0.964103	0.905983	0.637037
145	0.641026	0.964103	0.910256	0.656366
146	0.641026	0.964103	0.910256	0.656366
147	0.641026	0.964103	0.910256	0.656366
148	0.615385	0.964103	0.905983	0.637037
149	0.615385	0.953846	0.897436	0.609513
150	0.615385	0.953846	0.897436	0.609513
151	0.589744	0.958974	0.897436	0.603212
152	0.615385	0.948718	0.893162	0.596559
153	0.589744	0.958974	0.897436	0.603212
154	0.564103	0.964103	0.897436	0.597415
155	0.589744	0.964103	0.901709	0.617395
156	0.564103	0.964103	0.897436	0.597415
157	0.564103	0.964103	0.897436	0.597415
158	0.589744	0.964103	0.901709	0.617395
159	0.589744	0.964103	0.901709	0.617395
160	0.589744	0.964103	0.901709	0.617395
161	0.564103	0.964103	0.897436	0.597415

162	0.564103	0.964103	0.897436	0.597415
163	0.564103	0.964103	0.897436	0.597415
164	0.564103	0.964103	0.897436	0.597415
165	0.564103	0.964103	0.897436	0.597415
166	0.589744	0.964103	0.901709	0.617395
167	0.589744	0.958974	0.897436	0.603212
168	0.564103	0.964103	0.897436	0.597415
169	0.538462	0.964103	0.893162	0.57707
170	0.564103	0.958974	0.893162	0.583095
171	0.564103	0.958974	0.893162	0.583095
172	0.564103	0.958974	0.893162	0.583095
173	0.564103	0.958974	0.893162	0.583095
174	0.538462	0.953846	0.884615	0.548795
175	0.564103	0.953846	0.888889	0.569387
176	0.538462	0.953846	0.884615	0.548795
177	0.564103	0.953846	0.888889	0.569387
178	0.538462	0.948718	0.880342	0.535562
179	0.564103	0.948718	0.884615	0.556243
180	0.564103	0.948718	0.884615	0.556243
181	0.589744	0.964103	0.901709	0.617395
182	0.589744	0.964103	0.901709	0.617395
183	0.564103	0.964103	0.897436	0.597415
184	0.564103	0.958974	0.893162	0.583095
185	0.564103	0.953846	0.888889	0.569387
186	0.564103	0.953846	0.888889	0.569387
187	0.564103	0.953846	0.888889	0.569387
188	0.564103	0.953846	0.888889	0.569387
189	0.564103	0.964103	0.897436	0.597415
190	0.538462	0.964103	0.893162	0.57707
191	0.538462	0.964103	0.893162	0.57707
192	0.538462	0.964103	0.893162	0.57707
193	0.564103	0.964103	0.897436	0.597415
194	0.564103	0.964103	0.897436	0.597415
195	0.589744	0.958974	0.897436	0.603212
196	0.564103	0.958974	0.893162	0.583095
197	0.564103	0.958974	0.893162	0.583095
198	0.564103	0.964103	0.897436	0.597415
199	0.564103	0.964103	0.897436	0.597415
200	0.564103	0.958974	0.893162	0.583095
201	0.564103	0.958974	0.893162	0.583095
202	0.538462	0.958974	0.888889	0.562614
203	0.538462	0.964103	0.893162	0.57707
204	0.538462	0.958974	0.888889	0.562614

205	0.564103	0.958974	0.893162	0.583095
206	0.538462	0.958974	0.888889	0.562614
207	0.538462	0.958974	0.888889	0.562614
208	0.538462	0.958974	0.888889	0.562614
209	0.538462	0.958974	0.888889	0.562614
210	0.538462	0.958974	0.888889	0.562614
211	0.564103	0.958974	0.893162	0.583095
212	0.564103	0.958974	0.893162	0.583095
213	0.538462	0.958974	0.888889	0.562614
214	0.538462	0.958974	0.888889	0.562614
215	0.564103	0.958974	0.893162	0.583095
216	0.538462	0.958974	0.888889	0.562614
217	0.538462	0.958974	0.888889	0.562614
218	0.538462	0.958974	0.888889	0.562614
219	0.538462	0.953846	0.884615	0.548795
220	0.538462	0.953846	0.884615	0.548795
221	0.589744	0.953846	0.893162	0.589617
222	0.589744	0.953846	0.893162	0.589617
223	0.589744	0.953846	0.893162	0.589617
224	0.589744	0.953846	0.893162	0.589617
225	0.589744	0.953846	0.893162	0.589617
226	0.589744	0.953846	0.893162	0.589617
227	0.589744	0.953846	0.893162	0.589617
228	0.589744	0.953846	0.893162	0.589617
229	0.589744	0.953846	0.893162	0.589617
230	0.589744	0.953846	0.893162	0.589617
231	0.589744	0.953846	0.893162	0.589617
232	0.589744	0.953846	0.893162	0.589617
233	0.589744	0.953846	0.893162	0.589617
234	0.589744	0.953846	0.893162	0.589617
235	0.615385	0.948718	0.893162	0.596559
236	0.615385	0.948718	0.893162	0.596559
237	0.615385	0.948718	0.893162	0.596559
238	0.615385	0.953846	0.897436	0.609513
239	0.615385	0.953846	0.897436	0.609513
240	0.615385	0.948718	0.893162	0.596559
241	0.615385	0.953846	0.897436	0.609513
242	0.615385	0.953846	0.897436	0.609513
243	0.615385	0.948718	0.893162	0.596559
244	0.615385	0.948718	0.893162	0.596559
245	0.615385	0.948718	0.893162	0.596559
246	0.589744	0.948718	0.888889	0.576566
247	0.589744	0.94359	0.884615	0.564019

248	0.615385	0.94359	0.888889	0.584092
249	0.615385	0.94359	0.888889	0.584092
250	0.615385	0.94359	0.888889	0.584092
251	0.615385	0.94359	0.888889	0.584092
252	0.615385	0.938462	0.884615	0.572078
253	0.615385	0.938462	0.884615	0.572078
254	0.615385	0.94359	0.888889	0.584092
255	0.615385	0.94359	0.888889	0.584092
256	0.615385	0.94359	0.888889	0.584092
257	0.615385	0.94359	0.888889	0.584092
258	0.615385	0.94359	0.888889	0.584092
259	0.615385	0.94359	0.888889	0.584092
260	0.615385	0.94359	0.888889	0.584092
261	0.615385	0.94359	0.888889	0.584092
262	0.615385	0.94359	0.888889	0.584092
263	0.615385	0.94359	0.888889	0.584092
264	0.615385	0.94359	0.888889	0.584092
265	0.615385	0.94359	0.888889	0.584092
266	0.615385	0.94359	0.888889	0.584092
267	0.615385	0.94359	0.888889	0.584092
268	0.615385	0.94359	0.888889	0.584092
269	0.615385	0.94359	0.888889	0.584092
270	0.615385	0.948718	0.893162	0.596559
271	0.615385	0.948718	0.893162	0.596559
272	0.615385	0.948718	0.893162	0.596559
273	0.615385	0.948718	0.893162	0.596559
274	0.615385	0.948718	0.893162	0.596559
275	0.615385	0.948718	0.893162	0.596559
276	0.615385	0.948718	0.893162	0.596559
277	0.615385	0.948718	0.893162	0.596559
278	0.589744	0.953846	0.893162	0.589617
279	0.589744	0.953846	0.893162	0.589617
280	0.589744	0.953846	0.893162	0.589617
281	0.589744	0.953846	0.893162	0.589617
282	0.589744	0.953846	0.893162	0.589617
283	0.589744	0.953846	0.893162	0.589617
284	0.589744	0.953846	0.893162	0.589617
285	0.589744	0.958974	0.897436	0.603212
286	0.589744	0.953846	0.893162	0.589617
287	0.589744	0.953846	0.893162	0.589617
288	0.589744	0.958974	0.897436	0.603212
289	0.615385	0.953846	0.897436	0.609513
290	0.615385	0.948718	0.893162	0.596559

291	0.615385	0.948718	0.893162	0.596559
292	0.615385	0.948718	0.893162	0.596559
293	0.615385	0.948718	0.893162	0.596559
294	0.615385	0.953846	0.897436	0.609513
295	0.589744	0.953846	0.893162	0.589617
296	0.589744	0.953846	0.893162	0.589617
297	0.589744	0.953846	0.893162	0.589617
298	0.589744	0.958974	0.897436	0.603212
299	0.564103	0.958974	0.893162	0.583095
300	0.564103	0.958974	0.893162	0.583095
301	0.564103	0.958974	0.893162	0.583095
302	0.564103	0.958974	0.893162	0.583095
303	0.564103	0.958974	0.893162	0.583095
304	0.564103	0.958974	0.893162	0.583095
305	0.564103	0.958974	0.893162	0.583095
306	0.564103	0.958974	0.893162	0.583095
307	0.564103	0.958974	0.893162	0.583095
308	0.589744	0.953846	0.893162	0.589617
309	0.589744	0.953846	0.893162	0.589617
310	0.589744	0.953846	0.893162	0.589617
311	0.589744	0.953846	0.893162	0.589617
312	0.589744	0.953846	0.893162	0.589617
313	0.589744	0.953846	0.893162	0.589617
314	0.589744	0.953846	0.893162	0.589617
315	0.589744	0.953846	0.893162	0.589617
316	0.589744	0.953846	0.893162	0.589617
317	0.589744	0.953846	0.893162	0.589617
318	0.589744	0.953846	0.893162	0.589617
319	0.589744	0.953846	0.893162	0.589617
320	0.589744	0.953846	0.893162	0.589617
321	0.589744	0.953846	0.893162	0.589617
322	0.589744	0.953846	0.893162	0.589617
323	0.589744	0.953846	0.893162	0.589617
324	0.589744	0.953846	0.893162	0.589617
325	0.589744	0.953846	0.893162	0.589617
326	0.589744	0.953846	0.893162	0.589617
327	0.615385	0.953846	0.897436	0.609513
328	0.615385	0.953846	0.897436	0.609513
329	0.615385	0.953846	0.897436	0.609513
330	0.615385	0.953846	0.897436	0.609513
331	0.615385	0.953846	0.897436	0.609513
332	0.615385	0.953846	0.897436	0.609513
333	0.615385	0.953846	0.897436	0.609513

334	0.589744	0.953846	0.893162	0.589617
335	0.589744	0.953846	0.893162	0.589617
336	0.589744	0.953846	0.893162	0.589617
337	0.615385	0.953846	0.897436	0.609513
338	0.615385	0.953846	0.897436	0.609513
339	0.615385	0.953846	0.897436	0.609513
340	0.615385	0.953846	0.897436	0.609513
341	0.615385	0.948718	0.893162	0.596559
342	0.615385	0.948718	0.893162	0.596559
343	0.641026	0.948718	0.897436	0.616243
344	0.641026	0.953846	0.901709	0.629098
345	0.615385	0.948718	0.893162	0.596559
346	0.615385	0.948718	0.893162	0.596559
347	0.615385	0.948718	0.893162	0.596559
348	0.615385	0.948718	0.893162	0.596559
349	0.615385	0.948718	0.893162	0.596559
350	0.615385	0.948718	0.893162	0.596559
351	0.615385	0.948718	0.893162	0.596559
352	0.615385	0.948718	0.893162	0.596559
353	0.615385	0.948718	0.893162	0.596559
354	0.615385	0.948718	0.893162	0.596559
355	0.615385	0.948718	0.893162	0.596559
356	0.589744	0.948718	0.888889	0.576566
357	0.589744	0.948718	0.888889	0.576566
358	0.589744	0.953846	0.893162	0.589617
359	0.589744	0.953846	0.893162	0.589617
360	0.589744	0.953846	0.893162	0.589617
361	0.589744	0.953846	0.893162	0.589617
362	0.589744	0.958974	0.897436	0.603212
363	0.589744	0.958974	0.897436	0.603212
364	0.589744	0.958974	0.897436	0.603212
365	0.589744	0.958974	0.897436	0.603212
366	0.589744	0.958974	0.897436	0.603212
367	0.589744	0.958974	0.897436	0.603212
368	0.589744	0.958974	0.897436	0.603212
369	0.589744	0.958974	0.897436	0.603212
370	0.589744	0.958974	0.897436	0.603212
371	0.589744	0.958974	0.897436	0.603212
372	0.589744	0.958974	0.897436	0.603212
373	0.589744	0.953846	0.893162	0.589617
374	0.564103	0.958974	0.893162	0.583095
375	0.564103	0.958974	0.893162	0.583095
376	0.564103	0.958974	0.893162	0.583095

377	0.564103	0.958974	0.893162	0.583095
378	0.564103	0.953846	0.888889	0.569387
379	0.564103	0.953846	0.888889	0.569387
380	0.564103	0.958974	0.893162	0.583095
381	0.564103	0.958974	0.893162	0.583095
382	0.564103	0.958974	0.893162	0.583095
383	0.564103	0.958974	0.893162	0.583095
384	0.564103	0.948718	0.884615	0.556243
385	0.564103	0.948718	0.884615	0.556243
386	0.564103	0.948718	0.884615	0.556243
387	0.564103	0.948718	0.884615	0.556243
388	0.564103	0.948718	0.884615	0.556243
389	0.564103	0.948718	0.884615	0.556243
390	0.564103	0.948718	0.884615	0.556243
391	0.589744	0.958974	0.897436	0.603212
392	0.589744	0.958974	0.897436	0.603212
393	0.589744	0.953846	0.893162	0.589617
394	0.589744	0.958974	0.897436	0.603212
395	0.589744	0.948718	0.888889	0.576566
396	0.589744	0.948718	0.888889	0.576566
397	0.589744	0.948718	0.888889	0.576566
398	0.589744	0.948718	0.888889	0.576566
399	0.589744	0.94359	0.884615	0.564019
400	0.589744	0.94359	0.884615	0.564019
401	0.589744	0.953846	0.893162	0.589617
402	0.589744	0.953846	0.893162	0.589617
403	0.589744	0.953846	0.893162	0.589617
404	0.589744	0.948718	0.888889	0.576566
405	0.589744	0.948718	0.888889	0.576566
406	0.589744	0.948718	0.888889	0.576566
407	0.589744	0.948718	0.888889	0.576566
408	0.589744	0.948718	0.888889	0.576566
409	0.589744	0.948718	0.888889	0.576566
410	0.589744	0.948718	0.888889	0.576566
411	0.589744	0.948718	0.888889	0.576566
412	0.589744	0.948718	0.888889	0.576566
413	0.589744	0.948718	0.888889	0.576566
414	0.589744	0.953846	0.893162	0.589617
415	0.589744	0.953846	0.893162	0.589617
416	0.589744	0.953846	0.893162	0.589617
417	0.589744	0.953846	0.893162	0.589617
418	0.589744	0.953846	0.893162	0.589617
419	0.589744	0.953846	0.893162	0.589617

420	0.589744	0.953846	0.893162	0.589617
421	0.589744	0.953846	0.893162	0.589617
422	0.589744	0.953846	0.893162	0.589617
423	0.589744	0.953846	0.893162	0.589617
424	0.589744	0.953846	0.893162	0.589617
425	0.589744	0.953846	0.893162	0.589617
426	0.589744	0.953846	0.893162	0.589617
427	0.589744	0.953846	0.893162	0.589617
428	0.589744	0.953846	0.893162	0.589617
429	0.589744	0.953846	0.893162	0.589617
430	0.589744	0.953846	0.893162	0.589617
431	0.589744	0.953846	0.893162	0.589617
432	0.589744	0.953846	0.893162	0.589617
433	0.589744	0.958974	0.897436	0.603212
434	0.589744	0.958974	0.897436	0.603212
435	0.615385	0.958974	0.901709	0.622992
436	0.615385	0.953846	0.897436	0.609513
437	0.615385	0.953846	0.897436	0.609513
438	0.615385	0.953846	0.897436	0.609513
439	0.615385	0.953846	0.897436	0.609513
440	0.615385	0.953846	0.897436	0.609513
441	0.615385	0.953846	0.897436	0.609513
442	0.615385	0.953846	0.897436	0.609513
443	0.615385	0.953846	0.897436	0.609513
444	0.615385	0.953846	0.897436	0.609513
445	0.615385	0.953846	0.897436	0.609513
446	0.615385	0.953846	0.897436	0.609513
447	0.615385	0.953846	0.897436	0.609513
448	0.615385	0.953846	0.897436	0.609513
449	0.615385	0.953846	0.897436	0.609513
450	0.615385	0.953846	0.897436	0.609513
451	0.615385	0.953846	0.897436	0.609513
452	0.615385	0.953846	0.897436	0.609513
453	0.615385	0.953846	0.897436	0.609513
454	0.615385	0.953846	0.897436	0.609513
455	0.615385	0.953846	0.897436	0.609513
456	0.615385	0.953846	0.897436	0.609513
457	0.615385	0.953846	0.897436	0.609513
458	0.615385	0.953846	0.897436	0.609513
459	0.615385	0.953846	0.897436	0.609513
460	0.615385	0.953846	0.897436	0.609513
461	0.615385	0.953846	0.897436	0.609513
462	0.615385	0.953846	0.897436	0.609513

463	0.615385	0.953846	0.897436	0.609513
464	0.615385	0.953846	0.897436	0.609513
465	0.615385	0.953846	0.897436	0.609513
466	0.615385	0.953846	0.897436	0.609513
467	0.615385	0.953846	0.897436	0.609513
468	0.615385	0.953846	0.897436	0.609513
469	0.615385	0.953846	0.897436	0.609513
470	0.615385	0.953846	0.897436	0.609513
471	0.615385	0.953846	0.897436	0.609513
472	0.615385	0.953846	0.897436	0.609513
473	0.615385	0.948718	0.893162	0.596559
474	0.615385	0.948718	0.893162	0.596559
475	0.615385	0.948718	0.893162	0.596559
476	0.615385	0.948718	0.893162	0.596559
477	0.615385	0.948718	0.893162	0.596559
478	0.615385	0.948718	0.893162	0.596559
479	0.615385	0.948718	0.893162	0.596559
480	0.615385	0.948718	0.893162	0.596559
481	0.615385	0.948718	0.893162	0.596559
482	0.615385	0.948718	0.893162	0.596559
483	0.615385	0.948718	0.893162	0.596559
484	0.615385	0.948718	0.893162	0.596559
485	0.615385	0.948718	0.893162	0.596559
486	0.615385	0.948718	0.893162	0.596559
487	0.589744	0.948718	0.888889	0.576566
488	0.589744	0.948718	0.888889	0.576566
489	0.589744	0.948718	0.888889	0.576566
490	0.589744	0.948718	0.888889	0.576566
491	0.589744	0.948718	0.888889	0.576566
492	0.589744	0.948718	0.888889	0.576566
493	0.589744	0.948718	0.888889	0.576566
494	0.589744	0.948718	0.888889	0.576566
495	0.615385	0.948718	0.893162	0.596559
496	0.615385	0.948718	0.893162	0.596559
497	0.615385	0.948718	0.893162	0.596559
498	0.615385	0.948718	0.893162	0.596559
499	0.615385	0.948718	0.893162	0.596559
500	0.615385	0.948718	0.893162	0.596559

(10) Dataset D_{10}

Number of features	SN	SP	ACC	MCC
4	0.461538	0.989744	0.901709	0.601556

5	0.487179	0.989744	0.905983	0.621868
6	0.487179	0.989744	0.905983	0.621868
7	0.512821	0.989744	0.910256	0.641744
8	0.487179	0.989744	0.905983	0.621868
9	0.512821	0.989744	0.910256	0.641744
10	0.538462	0.989744	0.91453	0.661222
11	0.538462	0.989744	0.91453	0.661222
12	0.564103	0.989744	0.918803	0.680336
13	0.564103	0.989744	0.918803	0.680336
14	0.564103	0.989744	0.918803	0.680336
15	0.564103	0.989744	0.918803	0.680336
16	0.641026	0.989744	0.931624	0.735789
17	0.641026	0.989744	0.931624	0.735789
18	0.641026	0.989744	0.931624	0.735789
19	0.641026	0.989744	0.931624	0.735789
20	0.641026	0.994872	0.935897	0.754082
21	0.615385	0.994872	0.931624	0.736239
22	0.615385	0.989744	0.92735	0.717594
23	0.615385	0.989744	0.92735	0.717594
24	0.589744	0.989744	0.923077	0.699118
25	0.589744	0.989744	0.923077	0.699118
26	0.615385	0.994872	0.931624	0.736239
27	0.615385	0.989744	0.92735	0.717594
28	0.615385	0.989744	0.92735	0.717594
29	0.641026	0.989744	0.931624	0.735789
30	0.641026	0.984615	0.92735	0.718393
31	0.641026	0.979487	0.923077	0.701818
32	0.615385	0.984615	0.923077	0.699896
33	0.666667	0.984615	0.931624	0.736619
34	0.641026	0.979487	0.923077	0.701818
35	0.615385	0.979487	0.918803	0.683062
36	0.615385	0.984615	0.923077	0.699896
37	0.615385	0.984615	0.923077	0.699896
38	0.615385	0.979487	0.918803	0.683062
39	0.615385	0.984615	0.923077	0.699896
40	0.589744	0.974359	0.910256	0.647732
41	0.615385	0.979487	0.918803	0.683062
42	0.641026	0.979487	0.923077	0.701818
43	0.641026	0.979487	0.923077	0.701818
44	0.641026	0.984615	0.92735	0.718393
45	0.666667	0.979487	0.92735	0.720294
46	0.666667	0.979487	0.92735	0.720294
47	0.641026	0.974359	0.918803	0.685994

48	0.641026	0.984615	0.92735	0.718393
49	0.692308	0.984615	0.935897	0.754594
50	0.666667	0.984615	0.931624	0.736619
51	0.692308	0.984615	0.935897	0.754594
52	0.692308	0.984615	0.935897	0.754594
53	0.692308	0.984615	0.935897	0.754594
54	0.666667	0.984615	0.931624	0.736619
55	0.692308	0.984615	0.935897	0.754594
56	0.692308	0.984615	0.935897	0.754594
57	0.666667	0.989744	0.935897	0.753724
58	0.666667	0.989744	0.935897	0.753724
59	0.666667	0.989744	0.935897	0.753724
60	0.666667	0.989744	0.935897	0.753724
61	0.666667	0.989744	0.935897	0.753724
62	0.666667	0.989744	0.935897	0.753724
63	0.666667	0.984615	0.931624	0.736619
64	0.692308	0.989744	0.940171	0.77142
65	0.666667	0.989744	0.935897	0.753724
66	0.666667	0.989744	0.935897	0.753724
67	0.717949	0.989744	0.944444	0.788893
68	0.717949	0.989744	0.944444	0.788893
69	0.717949	0.989744	0.944444	0.788893
70	0.74359	0.989744	0.948718	0.806162
71	0.74359	0.989744	0.948718	0.806162
72	0.74359	0.989744	0.948718	0.806162
73	0.717949	0.989744	0.944444	0.788893
74	0.717949	0.989744	0.944444	0.788893
75	0.717949	0.989744	0.944444	0.788893
76	0.717949	0.989744	0.944444	0.788893
77	0.717949	0.989744	0.944444	0.788893
78	0.717949	0.989744	0.944444	0.788893
79	0.717949	0.989744	0.944444	0.788893
80	0.717949	0.989744	0.944444	0.788893
81	0.717949	0.989744	0.944444	0.788893
82	0.717949	0.989744	0.944444	0.788893
83	0.717949	0.989744	0.944444	0.788893
84	0.717949	0.989744	0.944444	0.788893
85	0.717949	0.989744	0.944444	0.788893
86	0.717949	0.984615	0.940171	0.772337
87	0.74359	0.989744	0.948718	0.806162
88	0.74359	0.979487	0.940171	0.774246
89	0.74359	0.979487	0.940171	0.774246
90	0.74359	0.984615	0.944444	0.789865

91	0.74359	0.984615	0.944444	0.789865
92	0.74359	0.984615	0.944444	0.789865
93	0.74359	0.984615	0.944444	0.789865
94	0.74359	0.984615	0.944444	0.789865
95	0.74359	0.984615	0.944444	0.789865
96	0.74359	0.984615	0.944444	0.789865
97	0.717949	0.984615	0.940171	0.772337
98	0.74359	0.984615	0.944444	0.789865
99	0.692308	0.979487	0.931624	0.738512
100	0.692308	0.979487	0.931624	0.738512
101	0.692308	0.979487	0.931624	0.738512
102	0.692308	0.979487	0.931624	0.738512
103	0.717949	0.979487	0.935897	0.75649
104	0.717949	0.979487	0.935897	0.75649
105	0.717949	0.979487	0.935897	0.75649
106	0.717949	0.979487	0.935897	0.75649
107	0.717949	0.979487	0.935897	0.75649
108	0.717949	0.979487	0.935897	0.75649
109	0.692308	0.979487	0.931624	0.738512
110	0.717949	0.979487	0.935897	0.75649
111	0.717949	0.979487	0.935897	0.75649
112	0.717949	0.979487	0.935897	0.75649
113	0.717949	0.979487	0.935897	0.75649
114	0.717949	0.979487	0.935897	0.75649
115	0.717949	0.979487	0.935897	0.75649
116	0.717949	0.979487	0.935897	0.75649
117	0.717949	0.979487	0.935897	0.75649
118	0.717949	0.979487	0.935897	0.75649
119	0.717949	0.979487	0.935897	0.75649
120	0.717949	0.979487	0.935897	0.75649
121	0.717949	0.979487	0.935897	0.75649
122	0.717949	0.979487	0.935897	0.75649
123	0.717949	0.979487	0.935897	0.75649
124	0.717949	0.979487	0.935897	0.75649
125	0.717949	0.979487	0.935897	0.75649
126	0.717949	0.979487	0.935897	0.75649
127	0.717949	0.979487	0.935897	0.75649
128	0.717949	0.974359	0.931624	0.7413
129	0.692308	0.979487	0.931624	0.738512
130	0.692308	0.979487	0.931624	0.738512
131	0.692308	0.979487	0.931624	0.738512
132	0.666667	0.979487	0.92735	0.720294
133	0.692308	0.979487	0.931624	0.738512

134	0.692308	0.984615	0.935897	0.754594
135	0.666667	0.984615	0.931624	0.736619
136	0.666667	0.984615	0.931624	0.736619
137	0.666667	0.979487	0.92735	0.720294
138	0.641026	0.984615	0.92735	0.718393
139	0.615385	0.979487	0.918803	0.683062
140	0.641026	0.979487	0.923077	0.701818
141	0.641026	0.979487	0.923077	0.701818
142	0.641026	0.979487	0.923077	0.701818
143	0.666667	0.979487	0.92735	0.720294
144	0.641026	0.979487	0.923077	0.701818
145	0.641026	0.979487	0.923077	0.701818
146	0.666667	0.979487	0.92735	0.720294
147	0.666667	0.979487	0.92735	0.720294
148	0.641026	0.979487	0.923077	0.701818
149	0.641026	0.979487	0.923077	0.701818
150	0.641026	0.979487	0.923077	0.701818
151	0.641026	0.979487	0.923077	0.701818
152	0.641026	0.979487	0.923077	0.701818
153	0.641026	0.979487	0.923077	0.701818
154	0.641026	0.979487	0.923077	0.701818
155	0.641026	0.979487	0.923077	0.701818
156	0.641026	0.979487	0.923077	0.701818
157	0.641026	0.979487	0.923077	0.701818
158	0.641026	0.979487	0.923077	0.701818
159	0.641026	0.979487	0.923077	0.701818
160	0.641026	0.979487	0.923077	0.701818
161	0.641026	0.974359	0.918803	0.685994
162	0.641026	0.974359	0.918803	0.685994
163	0.641026	0.974359	0.918803	0.685994
164	0.641026	0.974359	0.918803	0.685994
165	0.641026	0.974359	0.918803	0.685994
166	0.641026	0.979487	0.923077	0.701818
167	0.641026	0.979487	0.923077	0.701818
168	0.641026	0.984615	0.92735	0.718393
169	0.641026	0.984615	0.92735	0.718393
170	0.641026	0.984615	0.92735	0.718393
171	0.641026	0.984615	0.92735	0.718393
172	0.641026	0.979487	0.923077	0.701818
173	0.641026	0.979487	0.923077	0.701818
174	0.641026	0.984615	0.92735	0.718393
175	0.641026	0.984615	0.92735	0.718393
176	0.641026	0.984615	0.92735	0.718393

177	0.641026	0.984615	0.92735	0.718393
178	0.615385	0.979487	0.918803	0.683062
179	0.615385	0.979487	0.918803	0.683062
180	0.615385	0.979487	0.918803	0.683062
181	0.589744	0.979487	0.91453	0.664004
182	0.589744	0.979487	0.91453	0.664004
183	0.589744	0.979487	0.91453	0.664004
184	0.589744	0.979487	0.91453	0.664004
185	0.589744	0.974359	0.910256	0.647732
186	0.589744	0.979487	0.91453	0.664004
187	0.589744	0.979487	0.91453	0.664004
188	0.589744	0.974359	0.910256	0.647732
189	0.589744	0.974359	0.910256	0.647732
190	0.589744	0.974359	0.910256	0.647732
191	0.589744	0.974359	0.910256	0.647732
192	0.589744	0.974359	0.910256	0.647732
193	0.589744	0.974359	0.910256	0.647732
194	0.589744	0.974359	0.910256	0.647732
195	0.589744	0.974359	0.910256	0.647732
196	0.589744	0.974359	0.910256	0.647732
197	0.589744	0.974359	0.910256	0.647732
198	0.589744	0.974359	0.910256	0.647732
199	0.589744	0.974359	0.910256	0.647732
200	0.589744	0.974359	0.910256	0.647732
201	0.589744	0.974359	0.910256	0.647732
202	0.589744	0.974359	0.910256	0.647732
203	0.589744	0.979487	0.91453	0.664004
204	0.589744	0.979487	0.91453	0.664004
205	0.589744	0.979487	0.91453	0.664004
206	0.589744	0.974359	0.910256	0.647732
207	0.589744	0.974359	0.910256	0.647732
208	0.589744	0.974359	0.910256	0.647732
209	0.589744	0.974359	0.910256	0.647732
210	0.589744	0.974359	0.910256	0.647732
211	0.589744	0.974359	0.910256	0.647732
212	0.589744	0.974359	0.910256	0.647732
213	0.589744	0.974359	0.910256	0.647732
214	0.589744	0.974359	0.910256	0.647732
215	0.589744	0.974359	0.910256	0.647732
216	0.589744	0.974359	0.910256	0.647732
217	0.589744	0.974359	0.910256	0.647732
218	0.589744	0.974359	0.910256	0.647732
219	0.589744	0.974359	0.910256	0.647732

220	0.589744	0.974359	0.910256	0.647732
221	0.589744	0.969231	0.905983	0.632216
222	0.589744	0.974359	0.910256	0.647732
223	0.589744	0.969231	0.905983	0.632216
224	0.589744	0.969231	0.905983	0.632216
225	0.615385	0.974359	0.91453	0.667017
226	0.615385	0.974359	0.91453	0.667017
227	0.615385	0.974359	0.91453	0.667017
228	0.615385	0.969231	0.910256	0.651695
229	0.615385	0.969231	0.910256	0.651695
230	0.615385	0.969231	0.910256	0.651695
231	0.615385	0.969231	0.910256	0.651695
232	0.615385	0.969231	0.910256	0.651695
233	0.615385	0.969231	0.910256	0.651695
234	0.641026	0.969231	0.91453	0.670862
235	0.615385	0.969231	0.910256	0.651695
236	0.615385	0.969231	0.910256	0.651695
237	0.564103	0.969231	0.901709	0.612401
238	0.589744	0.969231	0.905983	0.632216
239	0.589744	0.969231	0.905983	0.632216
240	0.589744	0.974359	0.910256	0.647732
241	0.589744	0.969231	0.905983	0.632216
242	0.589744	0.969231	0.905983	0.632216
243	0.589744	0.969231	0.905983	0.632216
244	0.564103	0.964103	0.897436	0.597415
245	0.564103	0.964103	0.897436	0.597415
246	0.564103	0.964103	0.897436	0.597415
247	0.564103	0.974359	0.905983	0.628112
248	0.564103	0.974359	0.905983	0.628112
249	0.564103	0.974359	0.905983	0.628112
250	0.589744	0.974359	0.910256	0.647732
251	0.589744	0.974359	0.910256	0.647732
252	0.564103	0.974359	0.905983	0.628112
253	0.589744	0.974359	0.910256	0.647732
254	0.589744	0.974359	0.910256	0.647732
255	0.589744	0.974359	0.910256	0.647732
256	0.538462	0.969231	0.897436	0.59222
257	0.538462	0.969231	0.897436	0.59222
258	0.538462	0.969231	0.897436	0.59222
259	0.538462	0.969231	0.897436	0.59222
260	0.564103	0.969231	0.901709	0.612401
261	0.564103	0.969231	0.901709	0.612401
262	0.564103	0.974359	0.905983	0.628112

263	0.589744	0.969231	0.905983	0.632216
264	0.589744	0.964103	0.901709	0.617395
265	0.564103	0.964103	0.897436	0.597415
266	0.564103	0.964103	0.897436	0.597415
267	0.564103	0.964103	0.897436	0.597415
268	0.589744	0.964103	0.901709	0.617395
269	0.564103	0.964103	0.897436	0.597415
270	0.564103	0.964103	0.897436	0.597415
271	0.589744	0.964103	0.901709	0.617395
272	0.589744	0.964103	0.901709	0.617395
273	0.589744	0.964103	0.901709	0.617395
274	0.589744	0.964103	0.901709	0.617395
275	0.564103	0.964103	0.897436	0.597415
276	0.589744	0.969231	0.905983	0.632216
277	0.589744	0.969231	0.905983	0.632216
278	0.615385	0.969231	0.910256	0.651695
279	0.615385	0.969231	0.910256	0.651695
280	0.615385	0.969231	0.910256	0.651695
281	0.615385	0.969231	0.910256	0.651695
282	0.615385	0.969231	0.910256	0.651695
283	0.615385	0.969231	0.910256	0.651695
284	0.615385	0.969231	0.910256	0.651695
285	0.615385	0.969231	0.910256	0.651695
286	0.615385	0.969231	0.910256	0.651695
287	0.615385	0.969231	0.910256	0.651695
288	0.615385	0.969231	0.910256	0.651695
289	0.615385	0.969231	0.910256	0.651695
290	0.615385	0.969231	0.910256	0.651695
291	0.615385	0.969231	0.910256	0.651695
292	0.615385	0.969231	0.910256	0.651695
293	0.615385	0.969231	0.910256	0.651695
294	0.615385	0.969231	0.910256	0.651695
295	0.615385	0.969231	0.910256	0.651695
296	0.589744	0.969231	0.905983	0.632216
297	0.589744	0.969231	0.905983	0.632216
298	0.589744	0.969231	0.905983	0.632216
299	0.589744	0.969231	0.905983	0.632216
300	0.615385	0.969231	0.910256	0.651695
301	0.589744	0.969231	0.905983	0.632216
302	0.589744	0.969231	0.905983	0.632216
303	0.589744	0.969231	0.905983	0.632216
304	0.589744	0.969231	0.905983	0.632216
305	0.589744	0.969231	0.905983	0.632216

306	0.589744	0.969231	0.905983	0.632216
307	0.589744	0.969231	0.905983	0.632216
308	0.589744	0.969231	0.905983	0.632216
309	0.589744	0.969231	0.905983	0.632216
310	0.589744	0.969231	0.905983	0.632216
311	0.589744	0.969231	0.905983	0.632216
312	0.589744	0.969231	0.905983	0.632216
313	0.589744	0.969231	0.905983	0.632216
314	0.589744	0.969231	0.905983	0.632216
315	0.589744	0.969231	0.905983	0.632216
316	0.589744	0.969231	0.905983	0.632216
317	0.589744	0.969231	0.905983	0.632216
318	0.589744	0.969231	0.905983	0.632216
319	0.589744	0.969231	0.905983	0.632216
320	0.615385	0.969231	0.910256	0.651695
321	0.615385	0.969231	0.910256	0.651695
322	0.615385	0.969231	0.910256	0.651695
323	0.615385	0.969231	0.910256	0.651695
324	0.615385	0.969231	0.910256	0.651695
325	0.615385	0.969231	0.910256	0.651695
326	0.615385	0.969231	0.910256	0.651695
327	0.615385	0.969231	0.910256	0.651695
328	0.641026	0.969231	0.91453	0.670862
329	0.641026	0.969231	0.91453	0.670862
330	0.641026	0.969231	0.91453	0.670862
331	0.666667	0.969231	0.918803	0.689741
332	0.666667	0.969231	0.918803	0.689741
333	0.666667	0.969231	0.918803	0.689741
334	0.666667	0.969231	0.918803	0.689741
335	0.666667	0.969231	0.918803	0.689741
336	0.666667	0.969231	0.918803	0.689741
337	0.666667	0.969231	0.918803	0.689741
338	0.666667	0.969231	0.918803	0.689741
339	0.666667	0.969231	0.918803	0.689741
340	0.666667	0.969231	0.918803	0.689741
341	0.666667	0.969231	0.918803	0.689741
342	0.666667	0.969231	0.918803	0.689741
343	0.641026	0.969231	0.91453	0.670862
344	0.666667	0.969231	0.918803	0.689741
345	0.666667	0.969231	0.918803	0.689741
346	0.666667	0.969231	0.918803	0.689741
347	0.666667	0.969231	0.918803	0.689741
348	0.666667	0.969231	0.918803	0.689741

349	0.666667	0.969231	0.918803	0.689741
350	0.666667	0.969231	0.918803	0.689741
351	0.666667	0.969231	0.918803	0.689741
352	0.666667	0.969231	0.918803	0.689741
353	0.666667	0.969231	0.918803	0.689741
354	0.666667	0.969231	0.918803	0.689741
355	0.666667	0.969231	0.918803	0.689741
356	0.666667	0.964103	0.91453	0.675406
357	0.666667	0.964103	0.91453	0.675406
358	0.666667	0.964103	0.91453	0.675406
359	0.666667	0.969231	0.918803	0.689741
360	0.666667	0.969231	0.918803	0.689741
361	0.666667	0.964103	0.91453	0.675406
362	0.666667	0.964103	0.91453	0.675406
363	0.666667	0.969231	0.918803	0.689741
364	0.666667	0.964103	0.91453	0.675406
365	0.641026	0.964103	0.910256	0.656366
366	0.641026	0.964103	0.910256	0.656366
367	0.641026	0.964103	0.910256	0.656366
368	0.641026	0.964103	0.910256	0.656366
369	0.641026	0.964103	0.910256	0.656366
370	0.641026	0.964103	0.910256	0.656366
371	0.641026	0.964103	0.910256	0.656366
372	0.641026	0.964103	0.910256	0.656366
373	0.641026	0.964103	0.910256	0.656366
374	0.641026	0.964103	0.910256	0.656366
375	0.666667	0.964103	0.91453	0.675406
376	0.666667	0.958974	0.910256	0.661638
377	0.641026	0.964103	0.910256	0.656366
378	0.641026	0.958974	0.905983	0.64246
379	0.641026	0.969231	0.91453	0.670862
380	0.641026	0.964103	0.910256	0.656366
381	0.641026	0.964103	0.910256	0.656366
382	0.641026	0.964103	0.910256	0.656366
383	0.641026	0.964103	0.910256	0.656366
384	0.641026	0.964103	0.910256	0.656366
385	0.641026	0.964103	0.910256	0.656366
386	0.666667	0.974359	0.923077	0.704687
387	0.666667	0.974359	0.923077	0.704687
388	0.666667	0.964103	0.91453	0.675406
389	0.666667	0.964103	0.91453	0.675406
390	0.666667	0.964103	0.91453	0.675406
391	0.666667	0.969231	0.918803	0.689741

392	0.666667	0.969231	0.918803	0.689741
393	0.666667	0.969231	0.918803	0.689741
394	0.666667	0.969231	0.918803	0.689741
395	0.666667	0.969231	0.918803	0.689741
396	0.666667	0.964103	0.91453	0.675406
397	0.666667	0.964103	0.91453	0.675406
398	0.666667	0.964103	0.91453	0.675406
399	0.666667	0.969231	0.918803	0.689741
400	0.666667	0.969231	0.918803	0.689741
401	0.666667	0.969231	0.918803	0.689741
402	0.666667	0.969231	0.918803	0.689741
403	0.666667	0.969231	0.918803	0.689741
404	0.666667	0.969231	0.918803	0.689741
405	0.666667	0.969231	0.918803	0.689741
406	0.666667	0.969231	0.918803	0.689741
407	0.666667	0.969231	0.918803	0.689741
408	0.666667	0.969231	0.918803	0.689741
409	0.666667	0.974359	0.923077	0.704687
410	0.666667	0.974359	0.923077	0.704687
411	0.666667	0.974359	0.923077	0.704687
412	0.666667	0.974359	0.923077	0.704687
413	0.666667	0.974359	0.923077	0.704687
414	0.666667	0.974359	0.923077	0.704687
415	0.666667	0.974359	0.923077	0.704687
416	0.666667	0.974359	0.923077	0.704687
417	0.666667	0.974359	0.923077	0.704687
418	0.666667	0.974359	0.923077	0.704687
419	0.666667	0.974359	0.923077	0.704687
420	0.666667	0.974359	0.923077	0.704687
421	0.666667	0.974359	0.923077	0.704687
422	0.666667	0.969231	0.918803	0.689741
423	0.666667	0.969231	0.918803	0.689741
424	0.666667	0.969231	0.918803	0.689741
425	0.666667	0.969231	0.918803	0.689741
426	0.666667	0.969231	0.918803	0.689741
427	0.666667	0.969231	0.918803	0.689741
428	0.666667	0.969231	0.918803	0.689741
429	0.666667	0.969231	0.918803	0.689741
430	0.666667	0.969231	0.918803	0.689741
431	0.666667	0.969231	0.918803	0.689741
432	0.666667	0.969231	0.918803	0.689741
433	0.666667	0.974359	0.923077	0.704687
434	0.666667	0.969231	0.918803	0.689741

435	0.666667	0.969231	0.918803	0.689741
436	0.666667	0.969231	0.918803	0.689741
437	0.666667	0.969231	0.918803	0.689741
438	0.666667	0.969231	0.918803	0.689741
439	0.666667	0.969231	0.918803	0.689741
440	0.666667	0.969231	0.918803	0.689741
441	0.666667	0.969231	0.918803	0.689741
442	0.666667	0.969231	0.918803	0.689741
443	0.666667	0.969231	0.918803	0.689741
444	0.666667	0.969231	0.918803	0.689741
445	0.666667	0.969231	0.918803	0.689741
446	0.666667	0.969231	0.918803	0.689741
447	0.666667	0.969231	0.918803	0.689741
448	0.666667	0.969231	0.918803	0.689741
449	0.666667	0.969231	0.918803	0.689741
450	0.666667	0.969231	0.918803	0.689741
451	0.666667	0.969231	0.918803	0.689741
452	0.666667	0.969231	0.918803	0.689741
453	0.641026	0.969231	0.91453	0.670862
454	0.641026	0.969231	0.91453	0.670862
455	0.641026	0.969231	0.91453	0.670862
456	0.641026	0.969231	0.91453	0.670862
457	0.641026	0.969231	0.91453	0.670862
458	0.641026	0.969231	0.91453	0.670862
459	0.666667	0.969231	0.918803	0.689741
460	0.666667	0.969231	0.918803	0.689741
461	0.666667	0.969231	0.918803	0.689741
462	0.641026	0.969231	0.91453	0.670862
463	0.666667	0.969231	0.918803	0.689741
464	0.666667	0.969231	0.918803	0.689741
465	0.666667	0.969231	0.918803	0.689741
466	0.666667	0.969231	0.918803	0.689741
467	0.666667	0.969231	0.918803	0.689741
468	0.666667	0.969231	0.918803	0.689741
469	0.666667	0.969231	0.918803	0.689741
470	0.666667	0.969231	0.918803	0.689741
471	0.641026	0.969231	0.91453	0.670862
472	0.641026	0.969231	0.91453	0.670862
473	0.641026	0.969231	0.91453	0.670862
474	0.641026	0.969231	0.91453	0.670862
475	0.641026	0.969231	0.91453	0.670862
476	0.641026	0.969231	0.91453	0.670862
477	0.641026	0.969231	0.91453	0.670862

478	0.641026	0.969231	0.91453	0.670862
479	0.641026	0.969231	0.91453	0.670862
480	0.641026	0.969231	0.91453	0.670862
481	0.641026	0.969231	0.91453	0.670862
482	0.641026	0.969231	0.91453	0.670862
483	0.641026	0.969231	0.91453	0.670862
484	0.641026	0.969231	0.91453	0.670862
485	0.615385	0.969231	0.910256	0.651695
486	0.615385	0.969231	0.910256	0.651695
487	0.615385	0.969231	0.910256	0.651695
488	0.641026	0.969231	0.91453	0.670862
489	0.641026	0.969231	0.91453	0.670862
490	0.641026	0.969231	0.91453	0.670862
491	0.641026	0.964103	0.910256	0.656366
492	0.641026	0.969231	0.91453	0.670862
493	0.641026	0.969231	0.91453	0.670862
494	0.641026	0.969231	0.91453	0.670862
495	0.641026	0.969231	0.91453	0.670862
496	0.641026	0.969231	0.91453	0.670862
497	0.641026	0.969231	0.91453	0.670862
498	0.641026	0.969231	0.91453	0.670862
499	0.641026	0.958974	0.905983	0.64246
500	0.641026	0.958974	0.905983	0.64246
