# Supplementary material for: Gene Ontology and KEGG Enrichment Analyses of Genes Related to Age-Related Macular Degeneration
Source: Biomed Res Int. 2014 Aug 6;2014:450386. doi: 10.1155/2014/450386 (PMC4140130; doi:10.1155/2014/450386)
Supplement: Supplementary file 1 — The Supplementary Material contains five files. In detail, Supplementary Material I lists 39 known AMD related genes and 1,950 randomly selected genes; Supplementary Material II lists the output of mRMR program on each dataset; Supplementary Material III lists the accuracies obtained by IFS and SMO on each dataset; Supplementary Material IV lists the IFS curve on each dataset; Supplementary Material V lists the features in the final optimal feature set. [file 450386.f1.zip › Supp-V.pdf]

**Supplementary Material V.** Features in the final optimal feature set

(1) Features of GO terms

| | | | |
|---|---|---|---|
| GO:0000003 | GO:0000152 | GO:0000254 | GO:0000267 |
| GO:0000268 | GO:0000302 | GO:0000303 | GO:0000406 |
| GO:0000506 | GO:0000710 | GO:0000832 | GO:0000910 |
| GO:0000930 | GO:0000932 | GO:0000956 | GO:0001300 |
| GO:0001315 | GO:0001502 | GO:0001540 | GO:0001552 |
| GO:0001567 | GO:0001569 | GO:0001574 | GO:0001763 |
| GO:0001798 | GO:0001848 | GO:0001850 | GO:0001867 |
| GO:0001910 | GO:0001917 | GO:0001919 | GO:0001922 |
| GO:0001923 | GO:0001937 | GO:0001938 | GO:0001957 |
| GO:0001968 | GO:0001970 | GO:0001974 | GO:0001991 |
| GO:0002028 | GO:0002032 | GO:0002033 | GO:0002042 |
| GO:0002055 | GO:0002204 | GO:0002232 | GO:0002262 |
| GO:0002282 | GO:0002429 | GO:0002446 | GO:0002457 |
| GO:0002462 | GO:0002480 | GO:0002504 | GO:0002523 |
| GO:0002537 | GO:0002544 | GO:0002551 | GO:0002575 |
| GO:0002666 | GO:0002767 | GO:0002820 | GO:0002891 |
| GO:0003009 | GO:0003032 | GO:0003069 | GO:0003084 |
| GO:0003085 | GO:0003100 | GO:0003273 | GO:0003416 |
| GO:0003729 | GO:0003743 | GO:0003746 | GO:0003747 |
| GO:0003823 | GO:0003828 | GO:0003948 | GO:0003997 |
| GO:0003999 | GO:0004012 | GO:0004175 | GO:0004181 |
| GO:0004221 | GO:0004252 | GO:0004301 | GO:0004441 |
| GO:0004473 | GO:0004489 | GO:0004496 | GO:0004509 |
| GO:0004517 | GO:0004631 | GO:0004653 | GO:0004784 |
| GO:0004832 | GO:0004843 | GO:0004864 | GO:0004866 |
| GO:0004876 | GO:0004903 | GO:0004906 | GO:0004943 |
| GO:0004944 | GO:0004962 | GO:0005010 | GO:0005041 |
| GO:0005044 | GO:0005072 | GO:0005105 | GO:0005114 |
| GO:0005124 | GO:0005161 | GO:0005172 | GO:0005198 |
| GO:0005201 | GO:0005367 | GO:0005462 | GO:0005488 |
| GO:0005506 | GO:0005520 | GO:0005534 | GO:0005537 |
| GO:0005540 | GO:0005548 | GO:0005576 | GO:0005577 |
| GO:0005578 | GO:0005579 | GO:0005581 | GO:0005582 |
| GO:0005585 | GO:0005586 | GO:0005588 | GO:0005592 |
| GO:0005597 | GO:0005602 | GO:0005604 | GO:0005608 |
| GO:0005610 | GO:0005615 | GO:0005688 | GO:0005876 |
| GO:0005922 | GO:0005930 | GO:0006029 | GO:0006086 |
| GO:0006108 | GO:0006122 | GO:0006165 | GO:0006168 |
| GO:0006183 | GO:0006290 | GO:0006311 | GO:0006323 |
| GO:0006351 | GO:0006438 | GO:0006491 | GO:0006527 |
| GO:0006537 | GO:0006562 | GO:0006576 | GO:0006649 |

| | | | |
|---|---|---|---|
| GO:0006657 | GO:0006700 | GO:0006704 | GO:0006705 |
| GO:0006707 | GO:0006741 | GO:0006749 | GO:0006801 |
| GO:0006809 | GO:0006869 | GO:0006930 | GO:0006940 |
| GO:0006956 | GO:0006957 | GO:0006958 | GO:0007063 |
| GO:0007080 | GO:0007091 | GO:0007179 | GO:0007181 |
| GO:0007183 | GO:0007184 | GO:0007256 | GO:0007398 |
| GO:0007497 | GO:0007567 | GO:0007603 | GO:0007606 |
| GO:0007632 | GO:0008094 | GO:0008106 | GO:0008187 |
| GO:0008200 | GO:0008209 | GO:0008228 | GO:0008236 |
| GO:0008253 | GO:0008271 | GO:0008272 | GO:0008347 |
| GO:0008360 | GO:0008390 | GO:0008395 | GO:0008397 |
| GO:0008428 | GO:0008440 | GO:0008513 | GO:0008554 |
| GO:0008574 | GO:0008753 | GO:0008817 | GO:0009100 |
| GO:0009236 | GO:0009258 | GO:0009409 | GO:0009432 |
| GO:0009449 | GO:0009583 | GO:0009597 | GO:0009749 |
| GO:0009881 | GO:0010001 | GO:0010165 | GO:0010189 |
| GO:0010369 | GO:0010388 | GO:0010466 | GO:0010501 |
| GO:0010510 | GO:0010519 | GO:0010625 | GO:0010700 |
| GO:0010703 | GO:0010711 | GO:0010716 | GO:0010738 |
| GO:0010740 | GO:0010747 | GO:0010759 | GO:0010760 |
| GO:0010763 | GO:0010764 | GO:0010804 | GO:0010814 |
| GO:0010815 | GO:0010821 | GO:0010826 | GO:0010863 |
| GO:0010873 | GO:0010875 | GO:0010900 | GO:0010902 |
| GO:0010916 | GO:0010923 | GO:0010934 | GO:0010987 |
| GO:0010991 | GO:0012501 | GO:0014043 | GO:0014056 |
| GO:0014826 | GO:0014909 | GO:0015030 | GO:0015074 |
| GO:0015079 | GO:0015116 | GO:0015126 | GO:0015129 |
| GO:0015184 | GO:0015301 | GO:0015432 | GO:0015485 |
| GO:0015727 | GO:0015788 | GO:0015811 | GO:0015920 |
| GO:0016046 | GO:0016056 | GO:0016209 | GO:0016254 |
| GO:0016422 | GO:0016495 | GO:0016554 | GO:0016578 |
| GO:0016603 | GO:0016619 | GO:0016805 | GO:0017038 |
| GO:0017046 | GO:0017158 | GO:0017176 | GO:0017186 |
| GO:0018242 | GO:0018243 | GO:0018894 | GO:0018963 |
| GO:0019049 | GO:0019064 | GO:0019166 | GO:0019430 |
| GO:0019439 | GO:0019509 | GO:0019543 | GO:0019730 |
| GO:0019799 | GO:0019825 | GO:0019826 | GO:0019835 |
| GO:0019836 | GO:0019882 | GO:0021517 | GO:0021678 |
| GO:0021681 | GO:0021762 | GO:0021858 | GO:0021885 |
| GO:0030020 | GO:0030023 | GO:0030145 | GO:0030176 |
| GO:0030185 | GO:0030199 | GO:0030214 | GO:0030229 |
| GO:0030235 | GO:0030247 | GO:0030279 | GO:0030346 |
| GO:0030382 | GO:0030449 | GO:0030492 | GO:0030497 |
| GO:0030593 | GO:0030621 | GO:0030704 | GO:0030895 |

| | | | |
|---|---|---|---|
| GO:0030903 | GO:0030949 | GO:0030950 | GO:0031012 |
| GO:0031061 | GO:0031077 | GO:0031109 | GO:0031134 |
| GO:0031232 | GO:0031302 | GO:0031362 | GO:0031491 |
| GO:0031573 | GO:0031575 | GO:0031593 | GO:0031616 |
| GO:0031701 | GO:0031714 | GO:0031798 | GO:0031954 |
| GO:0032003 | GO:0032089 | GO:0032139 | GO:0032181 |
| GO:0032302 | GO:0032364 | GO:0032368 | GO:0032387 |
| GO:0032393 | GO:0032395 | GO:0032417 | GO:0032432 |
| GO:0032449 | GO:0032463 | GO:0032467 | GO:0032488 |
| GO:0032490 | GO:0032510 | GO:0032691 | GO:0032757 |
| GO:0032784 | GO:0032793 | GO:0032805 | GO:0032809 |
| GO:0032816 | GO:0032855 | GO:0032930 | GO:0032958 |
| GO:0032964 | GO:0032990 | GO:0032994 | GO:0032996 |
| GO:0033005 | GO:0033081 | GO:0033093 | GO:0033141 |
| GO:0033145 | GO:0033192 | GO:0033269 | GO:0033299 |
| GO:0033364 | GO:0033602 | GO:0033627 | GO:0033691 |
| GO:0033700 | GO:0033778 | GO:0033781 | GO:0033961 |
| GO:0033962 | GO:0034021 | GO:0034056 | GO:0034103 |
| GO:0034122 | GO:0034148 | GO:0034185 | GO:0034189 |
| GO:0034346 | GO:0034358 | GO:0034365 | GO:0034369 |
| GO:0034378 | GO:0034405 | GO:0034436 | GO:0034437 |
| GO:0034445 | GO:0034447 | GO:0034450 | GO:0034605 |
| GO:0034616 | GO:0034701 | GO:0034755 | GO:0034767 |
| GO:0034875 | GO:0035082 | GO:0035092 | GO:0035254 |
| GO:0035257 | GO:0035270 | GO:0035417 | GO:0035473 |
| GO:0035478 | GO:0035545 | GO:0035624 | GO:0035630 |
| GO:0035645 | GO:0035767 | GO:0035845 | GO:0035873 |
| GO:0035924 | GO:0035988 | GO:0036020 | GO:0038025 |
| GO:0038026 | GO:0038052 | GO:0038062 | GO:0038063 |
| GO:0038084 | GO:0038091 | GO:0042033 | GO:0042088 |
| GO:0042159 | GO:0042231 | GO:0042253 | GO:0042310 |
| GO:0042325 | GO:0042461 | GO:0042462 | GO:0042490 |
| GO:0042554 | GO:0042583 | GO:0042612 | GO:0042613 |
| GO:0042622 | GO:0042637 | GO:0042660 | GO:0042753 |
| GO:0042801 | GO:0042832 | GO:0043022 | GO:0043024 |
| GO:0043064 | GO:0043117 | GO:0043142 | GO:0043153 |
| GO:0043171 | GO:0043178 | GO:0043183 | GO:0043184 |
| GO:0043196 | GO:0043206 | GO:0043229 | GO:0043248 |
| GO:0043277 | GO:0043331 | GO:0043381 | GO:0043405 |
| GO:0043490 | GO:0043498 | GO:0043536 | GO:0043537 |
| GO:0043570 | GO:0043654 | GO:0044241 | GO:0044464 |
| GO:0045039 | GO:0045103 | GO:0045116 | GO:0045128 |
| GO:0045174 | GO:0045179 | GO:0045254 | GO:0045263 |
| GO:0045309 | GO:0045343 | GO:0045356 | GO:0045359 |

| | | | |
|---|---|---|---|
| GO:0045414 | GO:0045423 | GO:0045494 | GO:0045541 |
| GO:0045599 | GO:0045618 | GO:0045824 | GO:0045869 |
| GO:0045953 | GO:0046314 | GO:0046434 | GO:0046548 |
| GO:0046599 | GO:0046629 | GO:0046649 | GO:0046813 |
| GO:0046814 | GO:0046907 | GO:0046911 | GO:0047408 |
| GO:0047409 | GO:0047497 | GO:0047547 | GO:0047696 |
| GO:0047915 | GO:0048009 | GO:0048018 | GO:0048074 |
| GO:0048242 | GO:0048243 | GO:0048245 | GO:0048246 |
| GO:0048251 | GO:0048255 | GO:0048306 | GO:0048386 |
| GO:0048407 | GO:0048525 | GO:0048535 | GO:0048549 |
| GO:0048593 | GO:0048598 | GO:0048640 | GO:0048666 |
| GO:0048678 | GO:0048739 | GO:0048773 | GO:0048844 |
| GO:0048845 | GO:0048878 | GO:0050262 | GO:0050610 |
| GO:0050655 | GO:0050665 | GO:0050689 | GO:0050711 |
| GO:0050766 | GO:0050777 | GO:0050790 | GO:0050840 |
| GO:0050868 | GO:0050884 | GO:0050921 | GO:0051000 |
| GO:0051028 | GO:0051106 | GO:0051148 | GO:0051224 |
| GO:0051262 | GO:0051264 | GO:0051265 | GO:0051271 |
| GO:0051272 | GO:0051280 | GO:0051297 | GO:0051301 |
| GO:0051346 | GO:0051389 | GO:0051400 | GO:0051537 |
| GO:0051580 | GO:0051607 | GO:0051635 | GO:0051651 |
| GO:0051654 | GO:0051683 | GO:0051782 | GO:0051788 |
| GO:0051856 | GO:0051873 | GO:0051881 | GO:0051894 |
| GO:0051898 | GO:0051918 | GO:0051926 | GO:0052033 |
| GO:0052723 | GO:0052724 | GO:0052871 | GO:0055087 |
| GO:0055090 | GO:0055093 | GO:0055094 | GO:0055106 |
| GO:0060011 | GO:0060042 | GO:0060052 | GO:0060068 |
| GO:0060090 | GO:0060123 | GO:0060128 | GO:0060129 |
| GO:0060220 | GO:0060228 | GO:0060229 | GO:0060265 |
| GO:0060266 | GO:0060272 | GO:0060311 | GO:0060312 |
| GO:0060313 | GO:0060319 | GO:0060342 | GO:0060346 |
| GO:0060364 | GO:0060394 | GO:0060613 | GO:0060697 |
| GO:0060706 | GO:0060744 | GO:0060745 | GO:0060750 |
| GO:0060754 | GO:0060841 | GO:0061035 | GO:0061043 |
| GO:0061045 | GO:0061077 | GO:0061153 | GO:0061302 |
| GO:0061338 | GO:0065005 | GO:0070050 | GO:0070052 |
| GO:0070173 | GO:0070195 | GO:0070206 | GO:0070247 |
| GO:0070287 | GO:0070326 | GO:0070328 | GO:0070383 |
| GO:0070429 | GO:0070430 | GO:0070433 | GO:0070483 |
| GO:0070506 | GO:0070524 | GO:0070530 | GO:0070541 |
| GO:0070584 | GO:0070633 | GO:0070722 | GO:0071028 |
| GO:0071062 | GO:0071230 | GO:0071253 | GO:0071472 |
| GO:0071482 | GO:0071503 | GO:0071529 | GO:0071593 |
| GO:0071622 | GO:0071777 | GO:0071813 | GO:0071953 |

| | | | |
|---|---|---|---|
| GO:0072321 | GO:0072332 | GO:0072384 | GO:0072563 |
| GO:0072573 | GO:0072606 | GO:0072686 | GO:0085029 |
| GO:0090031 | GO:0090037 | GO:0090050 | GO:0090281 |
| GO:0090305 | GO:0090321 | GO:0097186 | GO:1900006 |
| GO:1900086 | GO:1900142 | GO:1900165 | GO:2000256 |
| GO:2000349 | GO:2000402 | GO:2000504 | GO:2000533 |
| GO:2001027 | GO:2001028 | GO:2001141 | GO:2001199 |

(2) Features of KEGG pathways

| | | | |
|---|---|---|---|
| hsa00290 | hsa00450 | hsa00512 | hsa03013 |
| hsa04145 | hsa04610 | hsa04962 | hsa05133 |
| hsa05146 | hsa05150 | hsa05416 | |
