# Supplementary figures and images for: Gene Ontology and KEGG Enrichment Analyses of Genes Related to Age-Related Macular Degeneration
Source: Biomed Res Int. 2014 Aug 6;2014:450386. doi: 10.1155/2014/450386 (PMC4140130; doi:10.1155/2014/450386)

Supplementary Material IV. IFS-curve for each dataset

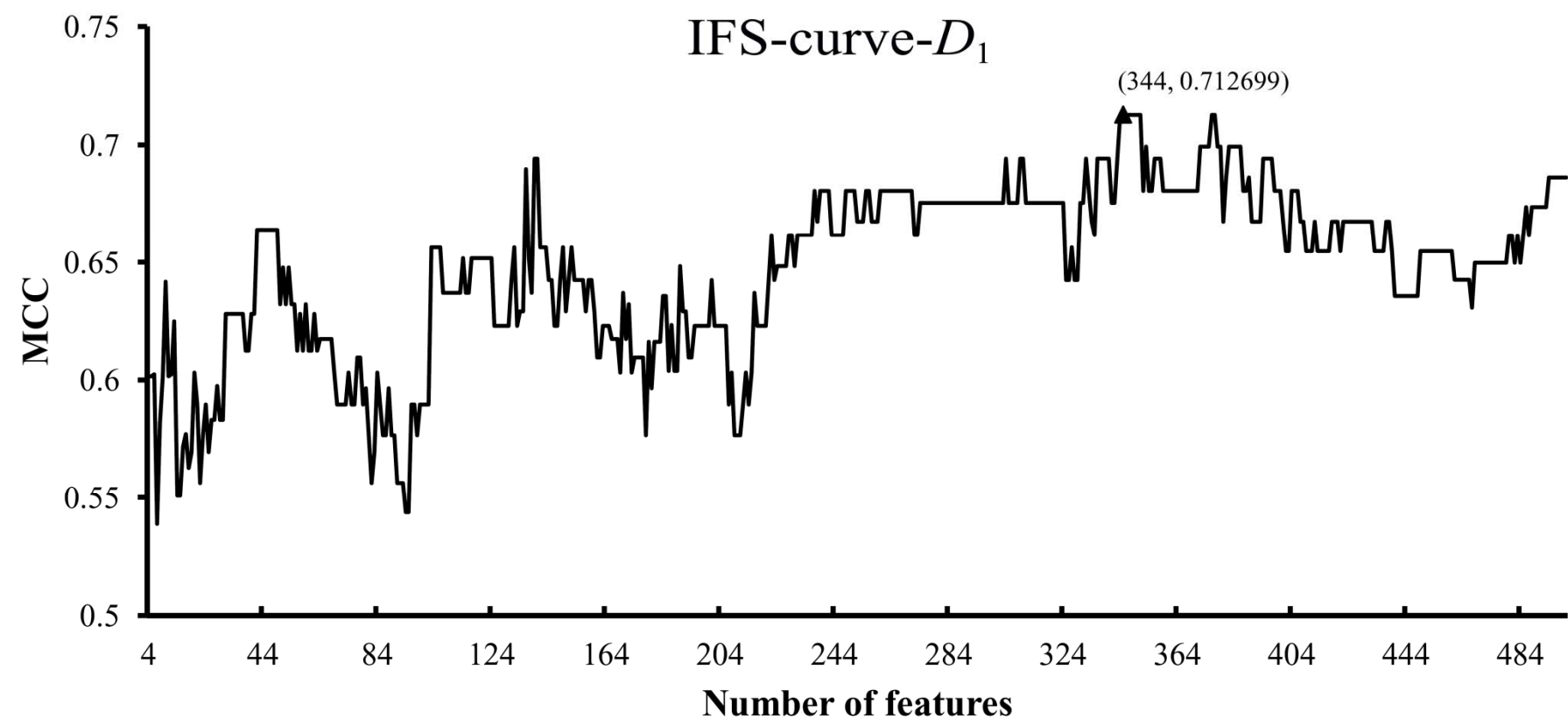

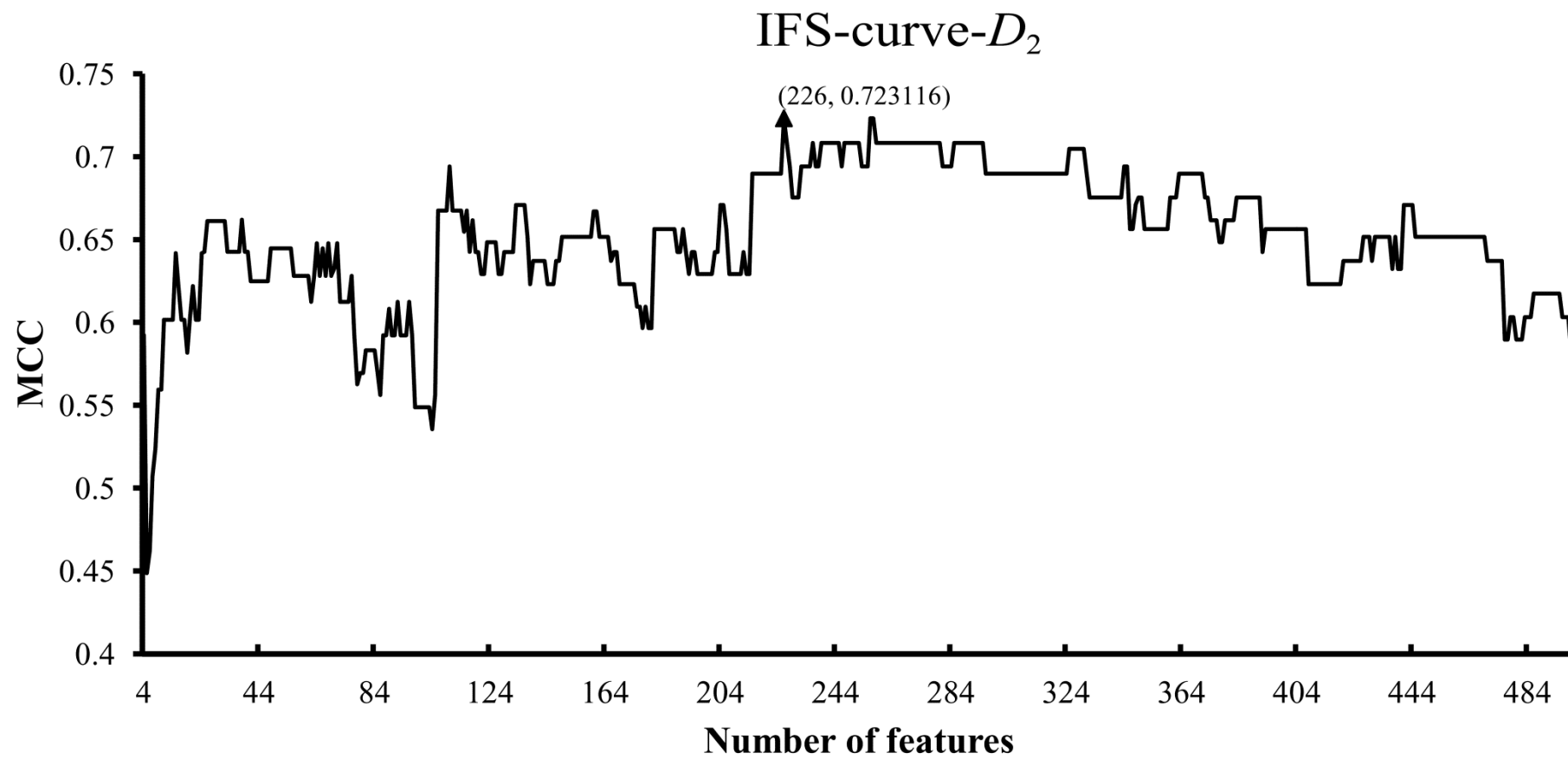

IFS-curve- $D_3$

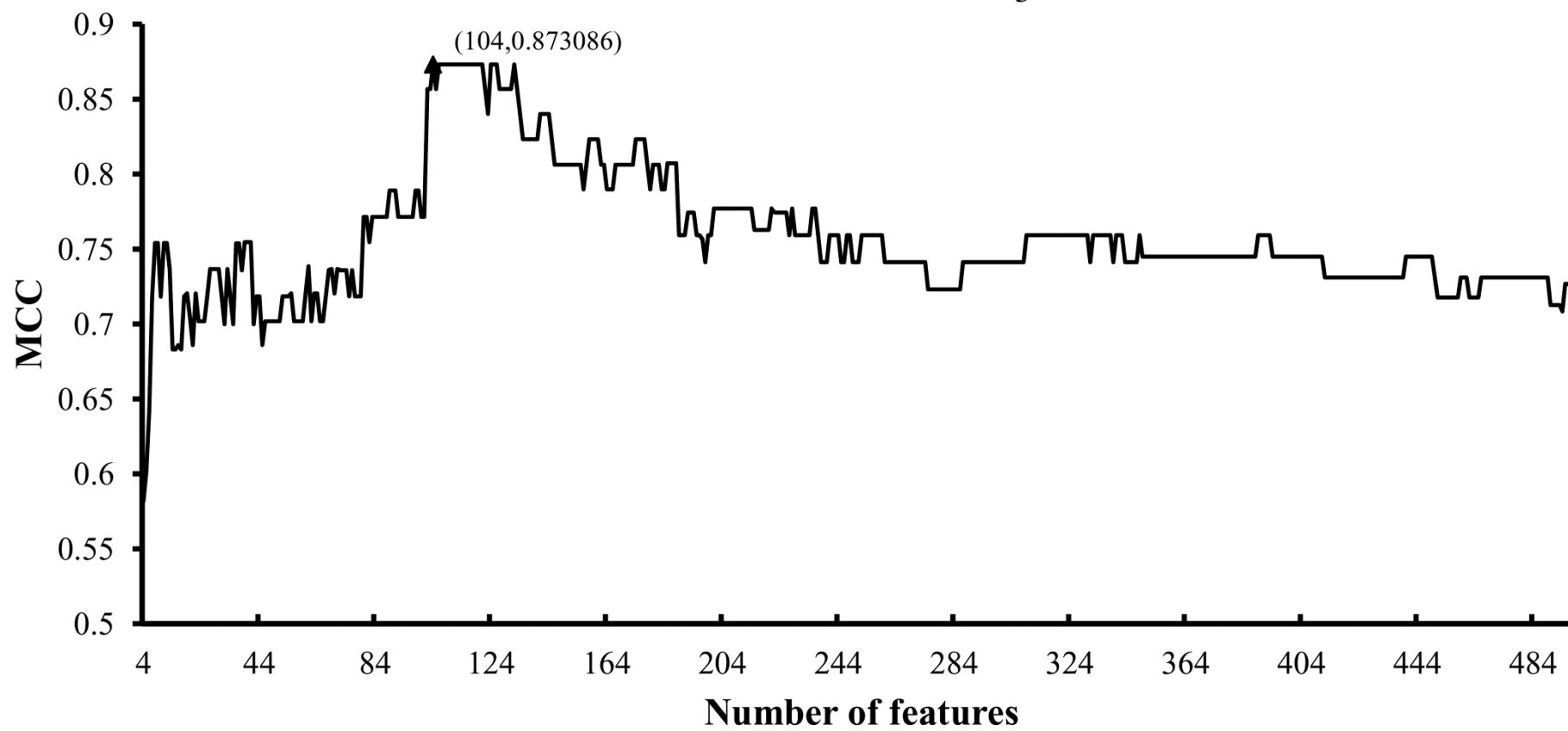

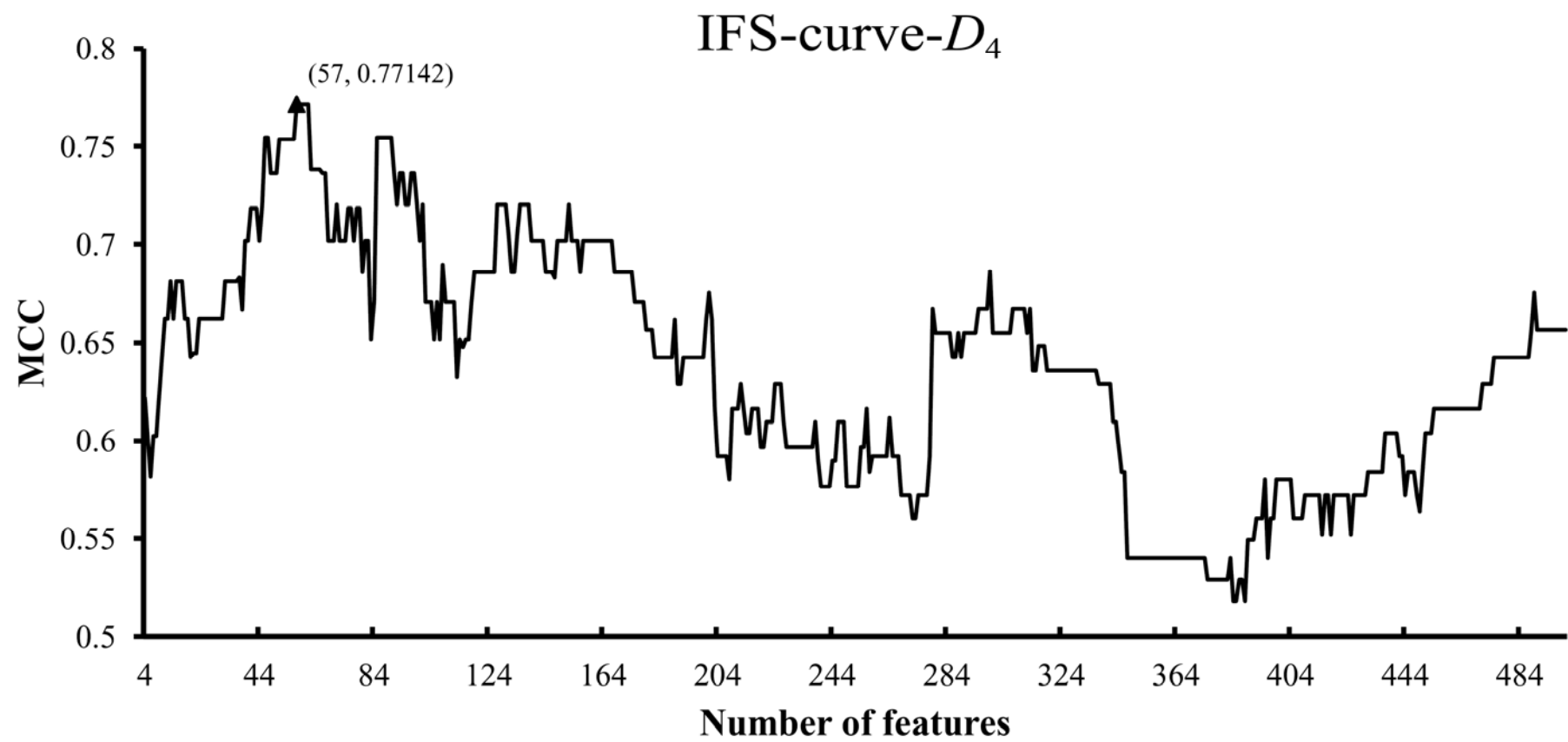

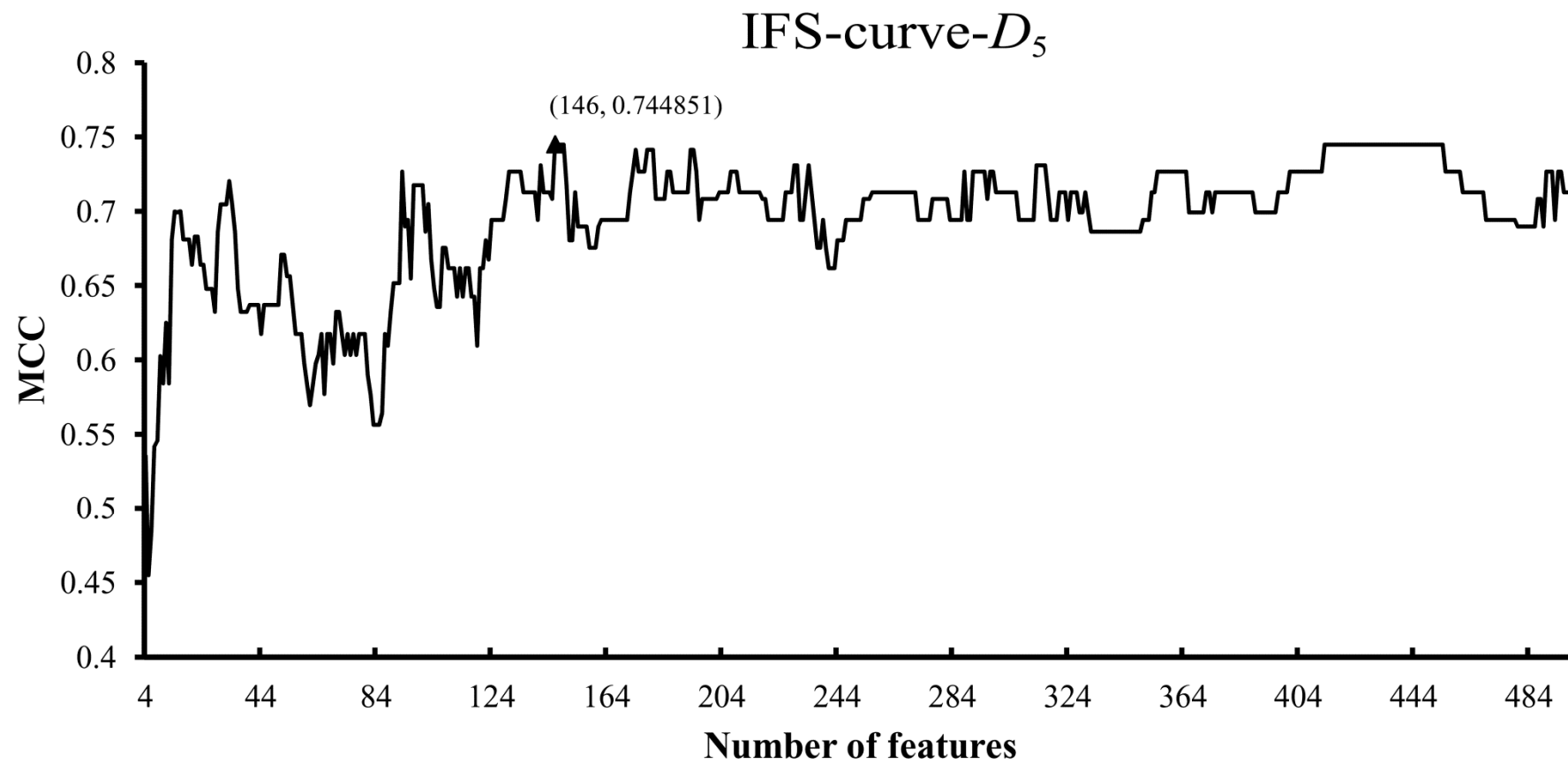

IFS-curve- $D_6$

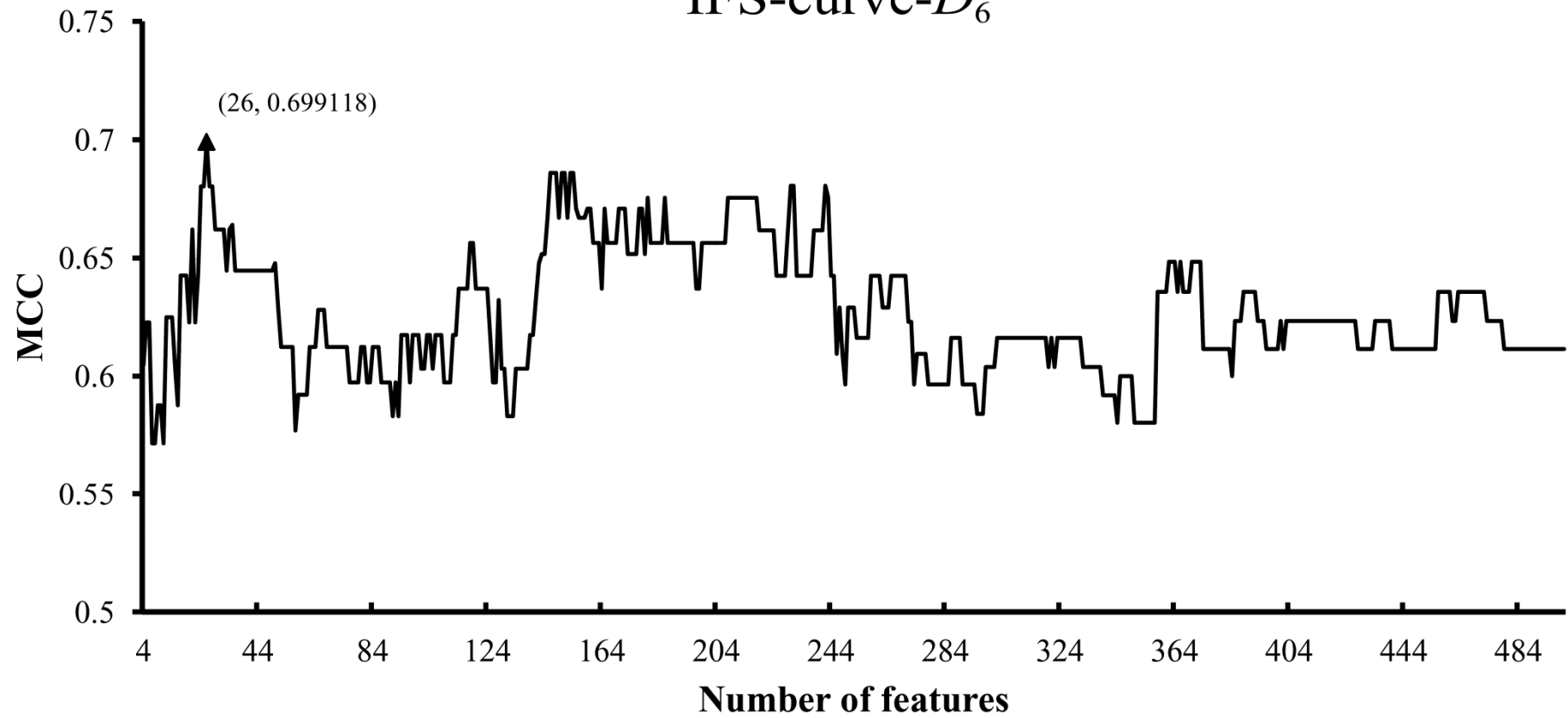

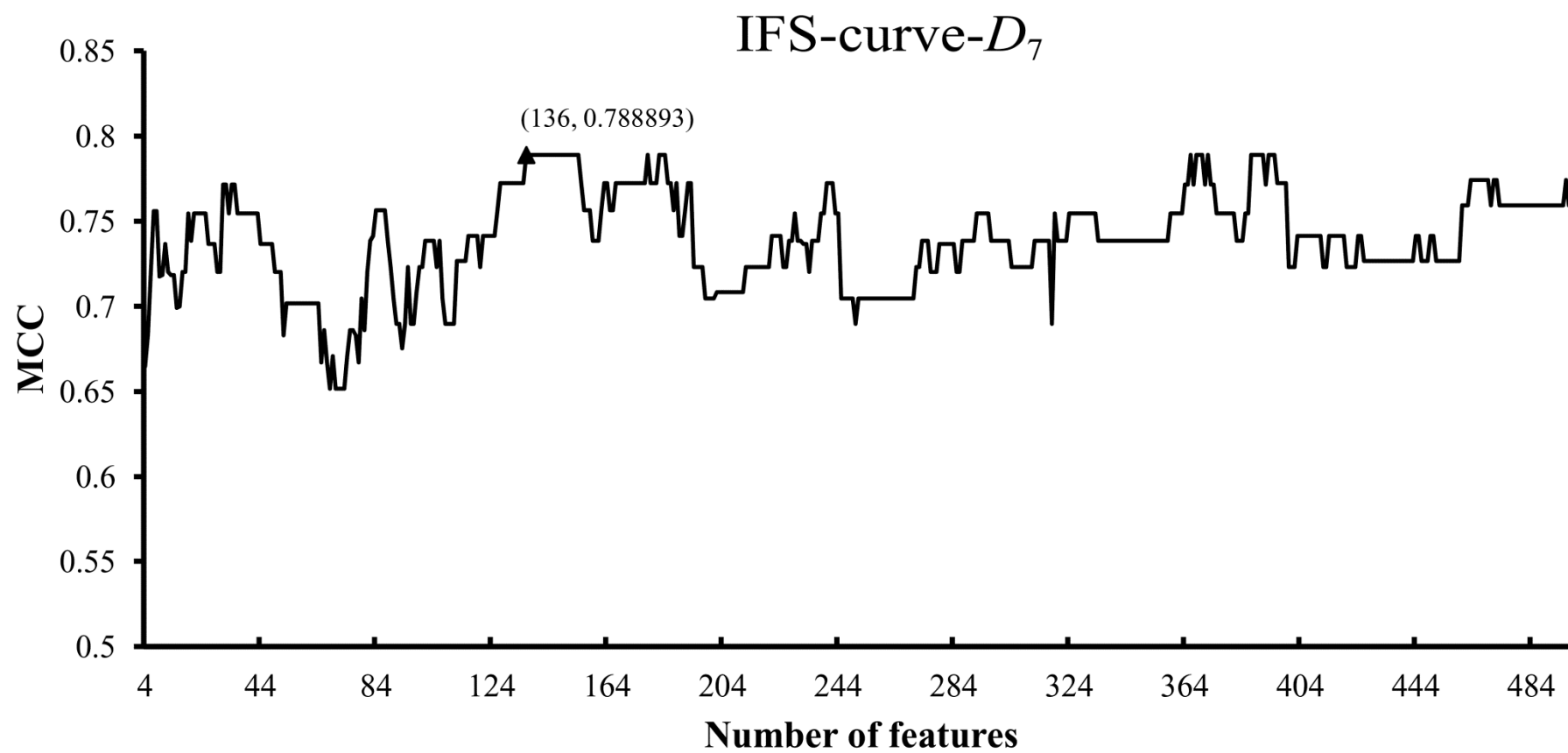

IFS-curve- $D_8$

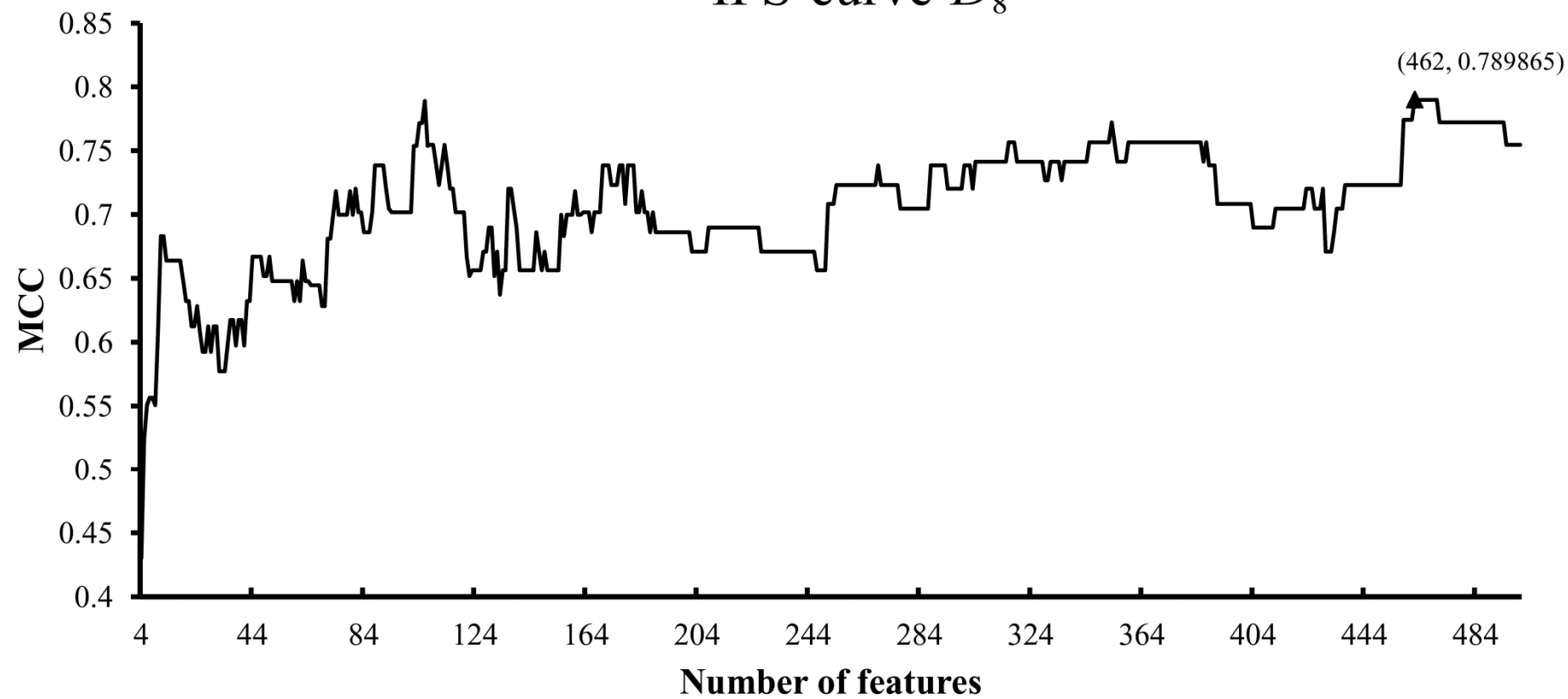

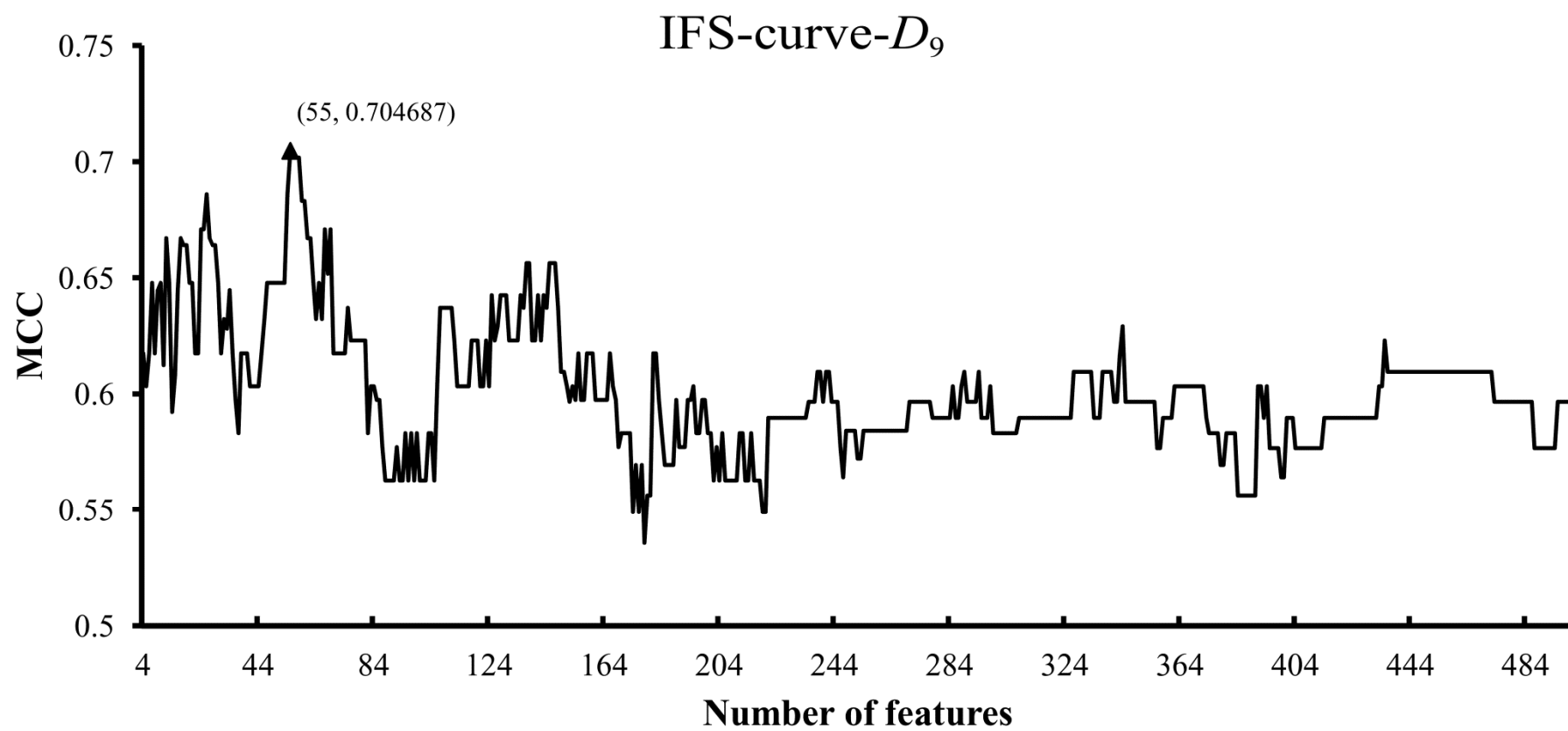

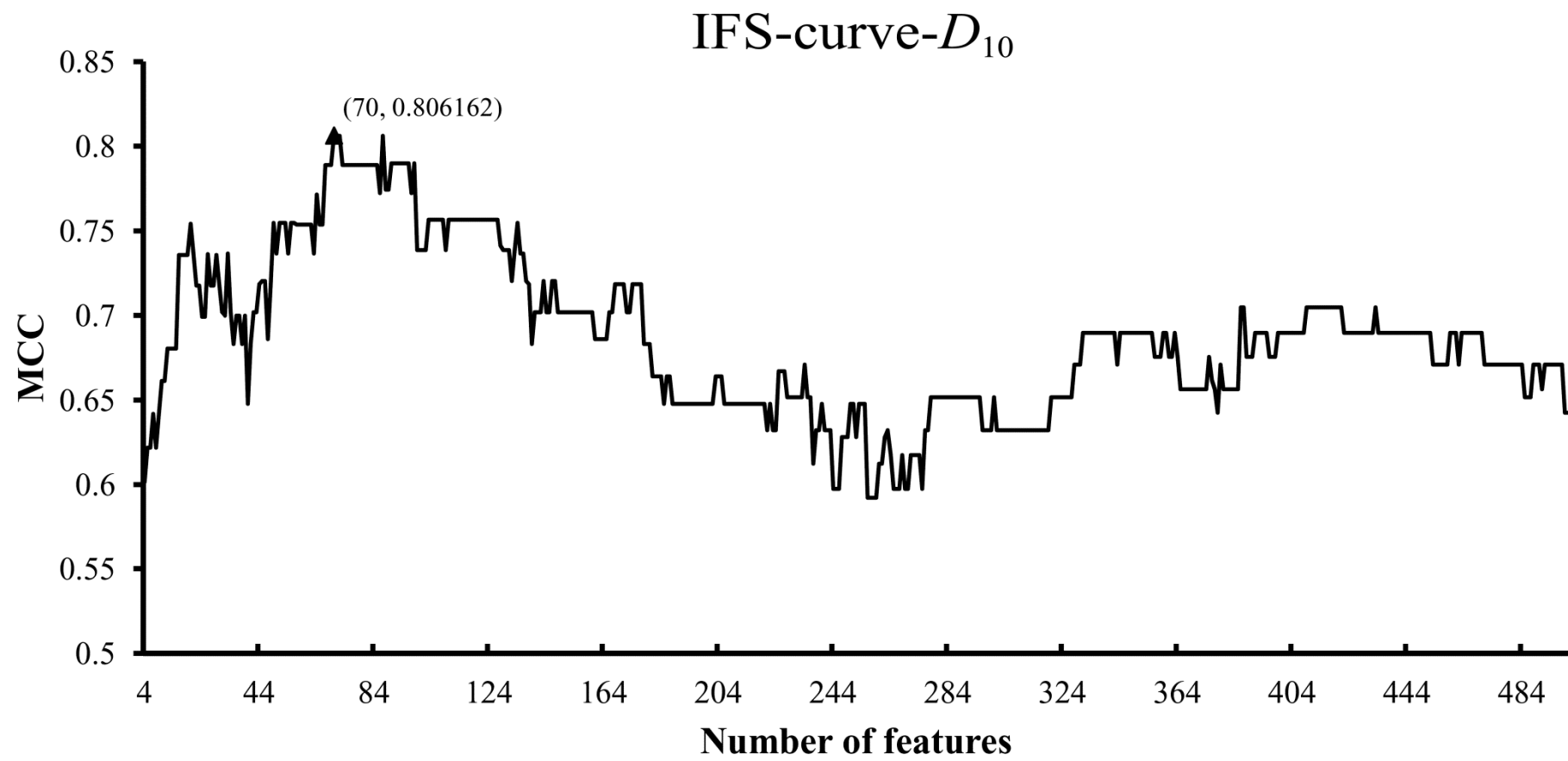

Supplement: Supplementary file 1 — The Supplementary Material contains five files. In detail, Supplementary Material I lists 39 known AMD related genes and 1,950 randomly selected genes; Supplementary Material II lists the output of mRMR program on each dataset; Supplementary Material III lists the accuracies obtained by IFS and SMO on each dataset; Supplementary Material IV lists the IFS curve on each dataset; Supplementary Material V lists the features in the final optimal feature set. [file 450386.f1.zip › Supp-IV.pdf]
